# Supplementary material for: Brain expression quantitative trait locus and network analyses reveal downstream effects and putative drivers for brain-related diseases
Source: Nat Genet. 2023 Feb 23;55(3):377–88. doi: 10.1038/s41588-023-01300-6 (PMC10011140; doi:10.1038/s41588-023-01300-6)

nature genetics

Article https://doi.org/10.1038/s41588-023-01300-6

Brain expression quantitative trait locus and network analyses reveal downstream effects and putative drivers for brain-related diseases

In the format provided by the authors and unedited

Table of contents

RNA-seq alignment and Quality control ... 4

PCA-based outlier identification ... 4

Covariate correction ... 4

Processing public samples from the European Nucleotide Archive ... 4

ENA Sample selection ... 4

Genotyping and imputation of ENA samples ... 5

Definition of eQTL datasets ... 7

Genotype QC ... 7

Linking genotyped individuals with RNA-seq samples ... 8

Sample mix-up and duplicate identification ... 8

eQTL analysis ... 9

Definition of *cis*- and *trans*-eQTL in this manuscript ... 9

Cis-eQTLs ... 9

Optimal number of PCs to regress out ... 10

Independent, non-primary *cis*-eQTLs ... 11

Trans-eQTLs ... 11

Removing cross-mapping artefacts from *trans*-eQTL results ... 12

Comparison of eQTL multiple testing correction methods based on permutations ... 13

MAPT and the influence of patch sequences in GRCh38 on RNA-seq quantification ... 15

Properties of primary, secondary, tertiary, and quaternary *cis*-eQTLs ... 15

Enrichment of primary vs non-primary *cis*-eQTL genes ... 17

Cortex eQTL concordance and heterogeneity between datasets ... 17

eQTL agreement ... 17

eQTL agreement calculations ... 17

Cis-eQTL agreement between tissues and ancestries ... 18

Cell type deconvolution and ieQTL analysis ... 20

Estimation of cell type proportions ... 20

Identification of cell type dependent eQTLs ... 21

Determining significance of ieQTLs through permutations ... 21

Replication of cell type dependent eQTL effects ... 22

Cis-eQTL LD overlap **23**

Mendelian randomization (MR) and colocalization **25**

Methods **25**
Annotating suggestive MR findings 25
Taylor expansion for the Wald ratio standard error 25

Results **26**
Alzheimer's disease 26
Attention deficit/hyperactivity disorder (ADHD) 28
Amyotrophic lateral sclerosis 28
Autism spectrum disorder 29
Bipolar disorder 29
Epilepsy 30
Frontotemporal dementia 31
Major depressive disorder 31
Multiple Sclerosis 32
Parkinson's disease 36
Schizophrenia 38
Years of schooling and cognitive function 42
Brain volume 44
Mendelian disease overlap 44

Systematic colocalization comparison of AD risk GWAS and cortex eQTLs **45**
Method 45
Results 45

Colocalization of top MR hits with opposite effect directionalities between *MetaBrain* and eQTLGen **45**

MR comparison between *MetaBrain* and eQTLGen in multiple sclerosis **46**

Trans-eQTL results **48**

***Trans*-eQTLs in the 7p21.3 locus** **48**

***Cis*-eQTL index SNP enrichment for *trans*-eQTL SNPs** **51**

Comparison of predicted cell count proportions between AD patients and neurological controls **52**

Gene co-regulation network **52**

Gene prioritization using Downstreamer **53**

Overview of Downstreamer methodology **53**

Downstreamer step 1: Calculation of GWAS gene Z-scores **53**

Downstreamer step 2: Association of GWAS Z-scores with phenotypes **54**
Pre-processing of GWAS gene Z-scores and pruning of highly correlated genes 54
Generalized least squares model to calculate pathway enrichment and core gene scores 54
Pathway and gene set gene prediction 55
Co-regulation matrix 55

Integrating *cis*-eQTLs with Downstreamer 55
Downstreamer analysis in schizophrenia 56
Downstreamer analysis in MS 56
Downstreamer analysis in ALS 56
Acknowledgements 58
ROSMAP 58
Mayo 58
MSBB 58
CMC 58
GTEx 59
NABEC 59
TargetALS 59
Braineac 60
Data was collected from 60
European Nucleotide Archive 60
UCLA ASD, Bipseq, BrainGVEx and LIBD 60
Software 60
R packages 60
Python packages 60
Supplementary Figure Legends 61
Supplementary Table descriptions 69
References 76
Supplementary Figures 112

Supplementary Note

RNA-seq alignment and Quality control

PCA-based outlier identification

RNA-sequencing library preparation, and other technical factors can greatly influence the ability to quantify gene expression. Therefore, for a given sample such factors often influence the total variation. For example, such issues can be caused by problems during RNA-seq library preparation that led to an increased number of available transcripts to quantify, or conversely, a lack of variation in quantified transcripts (compared to other samples in the dataset). We therefore opted to identify RNA-seq outliers that were not explained by poor RNA-seq alignment metrics. For this purpose, we performed Principal Component Analysis (PCA) on the RNA data prior to normalization: we reasoned that the first two components capture excess or depletion of variation caused by technical problems. We identified 20 samples that were outliers in the PCA plot of the RNA-seq data, where principal component (PC) 1 was more than 4 standard deviations from the mean (**Supplementary Figure 5a**). Twenty outlier samples were removed and the PCs were recalculated (**Supplementary figure 5b**). We detected and removed 45 additional outlier samples. We confirmed no additional outlier samples in the third iteration and PC calculation (**Supplementary Figure 5c**), and 8,868 samples were taken through additional quality control (QC).

Covariate correction

We next removed genes with no variation and then TMM normalized, log_2-transformed, centered the genes, and Z-score transformed the RNA-seq counts per sample. PCA on the normalized expression data showed that datasets strongly cluster together (**Supplementary Figure 6a**), likely due to dataset specific technical differences (e.g., single-end versus paired-end sequencing). To correct for this, the normalized expression data was correlated against 77 covariates from different QC tools (FastQC[1], STAR[2], and Picard Tools[3]), such as percent protein coding, GC content, and 5' prime/3' prime bias. The top 20 correlated technical covariates (% coding bases, % mRNA bases, % intronic bases, median 3' prime bias, % usable bases, % intergenic bases, % UTR bases, % reads aligned in pairs, average mapped read length, average input read length, number of uniquely mapped reads, % reads with improper pairs, number of reads improper pairs, total sequences, total reads, % chimeras, number of HQ aligned reads, number of reads aligned, HQ aligned Q20 bases, HQ aligned bases) were regressed out of the expression data using a linear model. After covariate correction, clustering of datasets in PC1 and PC2 were no longer present (**Supplementary Figure 6b**).

Processing public samples from the European Nucleotide Archive

ENA Sample selection

The European Nucleotide Archive (ENA) contains many bulk RNA-seq samples from many different tissues. We aimed to select the majority of brain related samples from this database. To this end, we made use of the SkyMap[4] database, which consists of read counts for each sample in

ENA, quantified with Kallisto[5] v0.44.0 to GRCh build 38. We accessed this database through Synapse ([1]) and selected 74,052 samples where at least 70% of the reads were mapped for 34,739 genes. We log_2 transformed and quantile normalized the read counts and performed PCA on the sample covariance matrix in order to detect outlier samples, and observed clustering of tissues on PC2, no clear clustering of single end vs paired end reads, and clear clustering of single cell versus bulk RNA-seq on PC1 (**Supplementary Figure 1a**, **b and c**). We set a threshold of 0 on PC1 to select 60,163 bulk RNA-seq samples. We noticed a number of samples with perfectly correlated read counts. These are likely the same samples that have been uploaded to the public domain multiple times and, as a result, have been included multiple times in SkyMap[4]. We removed 1,244 perfectly correlated (duplicate) samples to remain with 58,919 samples.

In a previous analysis, we found that the following technical covariates all significantly correlated to our sample PC scores for multiple PCs (p-value<0.01): read length, paired/single end, total reads in the dataset, and percentage mapping read. This indicates that these technical factors affect the co-expression detected in the dataset, if not removed. Consequently, we next removed these covariates using linear regression, but decided not to correct for GC content per gene as this may also have biological meaning.

After the covariate correction, we recalculated the PCs, this time over the gene correlation matrix, and calculated PC scores for each sample for all PCs (**Supplementary Figure 1d**). We used the PC eigencoefficients to perform coregulation analysis using, limiting the analysis to 664 PCs having a Cronbach's alpha ≥0.7 (which is an often-used threshold to select reliable PCs). The resulting PC showed clear clustering of tissues. Next, we determined which samples were tissues and which were cell lines by using a prediction algorithm: for each PC the PC scores per sample we correlated the binary vector describing if a sample is a tissue or not, resulting in a 'tissue representation' score indicative of how well that PC captures the signal differentiating tissues from non-tissue samples. Next, we correlated these 'tissue representation' scores of each PC to the PC scores for a sample for each PC. The resulting score indicates how likely this sample is a tissue (higher is more likely). In a similar manner we calculated "Brain scores" (**Supplementary Figure 1e**). We used a cutoff of >2 for the resulting tissue score, selecting 2,446 samples. However, we observed a large cluster of 449 samples indicated as cell lines, which were from a single study including ERCC Spike in controls. As such we decided to remove these samples, leaving 1,997 samples. We finally investigated whether these samples included cancer samples, by calculating a 'cancer score', and observed 292 samples which could be indicated as brain cancer samples. To maximize our sample size, we opted to include those samples (**Supplementary Figure 1f**). The selected samples were then downloaded from ENA using their SRA identifiers.

Genotyping and imputation of ENA samples

Since we intended to include the ENA samples also in the eQTL analysis, genotype data was not available for these samples, we called genotypes using the RNA-seq reads. In a previous publication, we have shown that this can be performed with relatively high accuracy, suitable for eQTL analysis[6].

Prior to genotype calling, we first aligned the samples using STAR[15] (version 2.6.1c) with GENCODE[13] v32 primary assembly as a reference, while omitting patch sequences from the reference. During this process, some samples failed to align, leaving 1,761 samples for genotype calling. After converting aligned SAM files to sorted BAM files, we used several GATK[7] (version 4.0.8.1) steps for genotyping. In order we used MarkDuplicates to flag duplicated alignments, SplitNCigarReads to split reads into exon segments and clip reads overhanging introns, IndelRealignment to locally realign reads to minimize mismatching bases, BQSR to detect systematic errors made by the sequencer when it estimated the quality score of each base call, and HaplotypeCallerGvcf for estimating most likely genotypes and allele frequencies. Per interval in storage.googleapis.com/gatk-test-data/intervals/hg38.even.handcurated.20k.chr*.intervals (where chr* is chr1, chr2, etc), we created a genomic database using GATK's GenomicsDBImport. Finally, we performed joined genotype calling per interval, using GATK's GenotypeGVCF. Using this procedure, we called a total of 28,798,830 variants.

Since genes have varying read depths, genotype calls for many of the variants will have high missingness. We therefore first filtered out variants with >50% missingness using vcftools[8] v0.1.14, selecting calls for biallelic single nucleotide polymorphisms (SNPs) with minor allele frequency (MAF) >0.1%, a minimal read depth (DP) of 10, and a minimal genotype quality (GQ) of 20. After this filter, 160,175 SNPs remained. Next, we evaluated missingness per sample, using the plink v1.9 --missing option and detected 315 samples with >50% missing genotype calls. We then repeated the vcftools filter step while omitting these 315 samples, leaving 210,498 SNPs. To identify the ancestry of the included samples, we next PCA on the genotypes, after merging overlapping SNPs with 1000 genomes phase 3 v5a, and pruning with plink v1.9 --indep-pairwise 50 5 0.2. We evaluated the first two PCs (**Supplementary Figure 2**) and assigned sample ancestry labels by performing k-nearest neighbors (k=7) on these PCS. We observed a large cluster of 388 samples to the left of the samples labeled EUR. Upon closer inspection, these samples were from SRA project accession PRJNA208369, which is a study in the reliability of RNA-seq and includes spike in RNA sequences. We therefore removed those samples, leaving 1,060 samples for further processing.

We note that we did not explicitly filter for genotype calls with aberrant allelic bias (AB), this can lead to false positive genotype calls. It has been shown that this may affect downstream analyses, for example when performing allele specific expression analysis[9]. Therefore, we investigated the distribution of average AB across all genotyped variants in the ENA dataset (**Supplementary Figure 3a**). We observed that homozygous reference and alternate calls generally had expected average AB values (around 0 for homozygous reference, and 1 for homozygous alternate). For heterozygote calls, we observed that 83% of variants had an average AB between 0.2 and 0.8. Furthermore, 1% of variants had an average AB value <0.1 or >0.9, indicating that for the majority of the heterozygous calls have AB values that fall within the expected range. As such, we note that indeed a number of variants may contain false-positive genotype calls, but this is likely true for the minority of included variants.

We also note that we did not explicitly limit the genotype calling to exonic regions, because RNA-seq reads can also map to regions outside of genes, such as enhancer regions[10]. However, these calls can potentially be of poorer quality than those called in exonic regions due to lower coverage. We annotated variants using SNPEff[11]. Of the 101,499 variants in the ENA genotype call set before imputation, 13,212 (13.02%) are located in exons, 55,095 in introns (54.28%), 10,114 (9.96%) close to genes (5k up or downstream from gene), 20,797 intergenic (20.49%) and 170 other (0.17%), e.g. 3' and 5' UTR). Of the 1,488,359 variants in the ENA genotype call set after imputation, 141,448 (10.18%) are located in exons, 919,978 in introns (61.68%), 174,783 (11.74%) close to genes (5k up or downstream from gene), 241,720 intergenic (16.24%) and 1,932 other (0.1%, e.g. 3' and 5' UTR). We compared allele frequency estimates of the called and imputed variants with a MAF >1% between ENA and the AMP-AD datasets for EUR individuals and observed a correlation of 0.98 for variants in exonic regions and a correlation of 0.96 for variants located in intergenic regions, indicating that variant calls are highly correlated in terms of allele frequency regardless of their position to genes (**Supplementary Figure 3b).**

Regardless of these observations, we cannot exclude the possibility of false positive genotype calls in the ENA dataset. However, when comparing the eQTL Z-scores for ENA with those from the meta-analysis, we observed that 92% of the 8,610 eQTL genes had the same allelic direction of effect with the meta-analysis (**Supplementary Figure 3c**), suggesting that generally the lack of AB filtering or filtering of variants outside of exonic regions did not greatly affect the eQTL outcome.

The resulting genotypes still contain many missing values, while the joint genotyping approach of GATK provides genotype probabilities for many of the missing calls. We therefore replaced the missing calls by calculating genotyping likelihoods using the Beagle v4.1.11mar19.69c gtgl command, using the plink GRCh38 genome map as a reference genetic map. Then, we prepared the dataset for imputation by first lifting over the genotypes using GATK v4.1.4.1 LiftOverVCF using UCSC's hg38ToHg19 liftover chain file, and the hg19 genome build as a reference. Finally, we matched the allele encoding of the lifted over genotypes using the HRC.r1-1.GRCh37.wgs.mac5.sites.vcf.gz sites list and used the Michigan imputation server to impute using Haplotype Reference Consortium (HRC) v1.1 as a reference.

Definition of eQTL datasets

Genotype QC

For the array-based datasets, we first matched genotypes using GenotypeHarmonizer[12] using 1000 genomes phase 3 v5a (1kgp) as a reference, limited to variants having MAF >1%, <95% missingness and Hardy-Weinberg equilibrium p-value (HWE-P) <0.0001. Genotypes were then imputed using HRC v1.1 as a reference on the Michigan imputation server[13]. In all HRC imputed datasets, variants with imputation info score <0.3 were removed. For the whole genome sequencing (WGS) datasets, we removed indels and poorly genotyped SNPs having VQSR tranche <99.0, genotype quality <20, inbreeding coefficient <-0.3 and >5% missingness, setting

genotype calls with allelic depth <10 and allelic balance <0.2 or >0.8 as missing. WGS datasets were not imputed with HRC. Multi-allelic variants were then split into biallelic ones using the bcftools norm -m +snps command. Considering the small size of some of the datasets, we decided to focus further analysis on variants with MAF >1% and HWE-P >0.0001.

In each dataset, we removed genetically similar individuals by removing individuals with pihat >0.125, as calculated with PLINK 2.0[14]. Additionally, we merged genotypes with those from 1kgp, pruned genotypes with --indep-pairwise 50 5 0.2 in PLINK, and performed PCA on the sample correlation matrix. We performed k-nearest neighbors (k=7) on the first two PCs, using the known ancestry labels in 1kgp, to assign an ancestry to each genotyped sample. Most of the included samples were of EUR descent: 5,138 samples had an EUR assignment, 805 samples had an AFR assignment, and 573 samples were assigned to the other ancestries (**Supplementary Table 1, Figure 2b**).

Linking genotyped individuals with RNA-seq samples

For the purpose of eQTL analysis, we next assessed links between RNA-seq and genotype samples and noted that some individuals had multiple RNA-seq samples (e.g., from multiple brain regions) or multiple genotype samples (e.g., from different genotyping platforms).

In total, we were able to determine 7,644 links between RNA-seq samples and genotype samples (**Supplementary Table 1**), reflecting 3,525 EUR individuals, 624 AFR individuals and 510 individuals assigned to other ancestries. We then grouped linked RNA-seq samples based on ancestry and tissue group to prevent possible biases on eQTL results. For those individuals with multiple linked RNA-seq samples, we selected a sample at random within these groups. Within each tissue and ancestry group, we then selected unique genotype samples across datasets in such a way to maximize sample size per genotype dataset. For the eQTL analysis per tissue, we only considered those datasets having more than 30 unique linked samples available, and for which at least two independent datasets were available**.** Using these criteria for sample and dataset selection, we were able to create 7 eQTL discovery datasets: Basal ganglia-EUR, Cerebellum-EUR, Cortex-EUR, Cortex-AFR, Hippocampus-EUR and Spinal cord-EUR (**Supplementary Table 1, Figure 2c**).

Sample mix-up and duplicate identification

In each of these eQTL datasets, we next applied MixupMapper[15] per cohort to identify mismatched samples: MixupMapper uses eQTLs identified in a cohort to determine if the RNA-seq sample is the best match for its assigned genotype sample. Using this procedure, we detected a single mismatch in Braineac for Basal ganglia-EUR, and 4 mismatches in AMP-AD ROSMAP, 34 in BrainGVEX and 121 in the ENA cohort in Cortex-EUR (**Supplementary Table 1**).

Next, to detect potential duplicate genotype samples, for each of the eQTL discovery datasets, we merged the genotype datasets, filtered out variants with MAF <10% and HWE-P <0.001, and calculated a genetic pairwise relationship matrix after pruning the data using the –indep-pairwise 1000 50 0.8 option in PLINK2.0[14]. Using the values in the GRM, we selected individual pairs

with a value >0.2 as potential duplicates. From this set of pairs, we selected samples to keep using the following procedure: if one, but not both of the samples were from the ENA dataset, we opted to select the non-ENA sample, and otherwise, we selected the sample that had the highest 'PCT_USABLE_BASES' from their matching RNA-seq sample. Using this procedure, we detected multiple potential genotype duplicates across multiple datasets in the Cortex-EUR and Cortex-AFR eQTL discovery datasets (**Supplementary Table 1**).

This resulted in the following final eQTL datasets: Basal ganglia-EUR (n=208), Cerebellum-EUR (n=492), Cortex-EUR (n=2,683), Cortex-AFR (n=319), Hippocampus-EUR (n=208), and Spinal cord-EUR (n=108; **Supplementary Table 1, Figure 2c**).

eQTL analysis

Definition of *cis*- and *trans*-eQTL in this manuscript

While classically the definitions for *cis*- and *trans* are dependent on the biological mechanism[16], in this manuscript, we define *cis*-eQTLs as variants affecting nearby genes (1 megabase; Mb), and *trans*-eQTLs as variants affecting distal (>5 Mb) genes, with no clear distinction in mode of action.

Throughout this manuscript, we used the term *cis*-eQTLs to describe local eQTLs, and *trans*-eQTLs to describe distal effects. However, we acknowledge that *cis* and *trans* do not necessarily implicate local and distal, respectively, but rather the mode of action of the effect alleles[16]. Making this formal distinction would require additional analyses that determine the mode of allelic action, such as integration of QTLs from different molecular phenotypes to identify true *trans*-acting alleles (e.g.: transcription factor binding, protein expression and epigenetic signals) and allele specific expression to identify true *cis*-acting alleles.

We note that we did not evaluate eQTLs where the SNP-TSS distance was >1 Mb and <5 Mb, which potentially excludes detection of long-range *cis*-eQTLs or short-range *trans*-eQTLs. We expect however, that this excludes only a limited number of eQTLs, since we observed that this distance was <31Kb for 50% of *cis*-eQTLs (**Figure 3b**), indicating most *cis*-eQTLs are short-ranged. Additionally, we reasoned that the >5 Mb cutoff would prevent identification of false-positive *trans*-eQTLs due to long-range LD.

Cis-eQTLs

Our dataset consists of different tissues and ancestries, and samples have been collected in different institutes using different protocols. Consequently, combining these datasets to perform eQTL analysis is complicated, due to possible biases each of these factors may introduce. To resolve this issue, we opted to perform an eQTL meta-analysis within each of the defined eQTL discovery datasets.

To reduce the effect of possible gene expression outliers, we calculated Spearman's rank correlation coefficients for each eQTL in each dataset separately, and then meta-analyzed the resulting coefficients using a sample size weighted Z-score method, as described previously[17].

While we acknowledge that this method may provide less statistical power than the commonly used linear regression, we chose this method to provide conservative effect estimates.

To identify *cis*-eQTLs, we tested SNPs located within 1 Mb of the transcription start site (TSS), while for the identification of *trans*-eQTLs, we required this distance to be at least 5 Mb. For both analyses, we selected variants having a MAF>1%, and a HWE-P >0.0001. Using the GENCODE v32 annotation, we were able to quantify 58,243 genes, of which 19,373 are protein coding. While non-coding genes have been implicated to be important for brain function[18], these genes generally have poor genomic and functional annotations, meaning that it is often unknown in which pathway they function, and that there is uncertainty about their genomic sequence. We therefore focused our eQTL analyses on protein coding genes.

As before, the RNA-seq data was corrected for 20 technical covariates, and a series of variables indicating the originating dataset. To take into account effects of potential population stratification, we also corrected the RNA-seq data for 4 multi-dimensional scaling components derived from the genotype data using PLINK 2.0[14].

To account for any residual technical variation in the *cis*-eQTL analysis, we additionally corrected the gene expression data for PCs calculated over the RNA-seq sample correlation matrix. We determined the optimum number of PCs to remove, by performing a set of eQTL meta-analyses, removing up to 100 PCs, in steps of 10 PCs, for each eQTL dataset (**Supplementary Figure 7**). This correction was performed separately on each eQTL discovery dataset and eQTL analysis was then performed on the residual expression values.

To correct for multiple testing, we reperformed each of these meta-analyses, while permuting the sample labels. For each gene, we retained the p-value of the SNP with the lowest nominal association p-value in both the unpermuted and permuted results. Then, we fitted a beta distribution using the permuted p-values per gene, which we used to adjust the unpermuted nominal p-value, comparable to the approach implemented in FastQTL/QTLTools[19]. To determine significance, we then applied the q-values package in R[20] on the beta-distribution adjusted p-values per gene, using a lambda of 0.85, and considered genes with a q-value <0.05 as significant.

Optimal number of PCs to regress out

To determine the optimal number of PCs to remove for the *cis*-eQTL analysis, we used 250 permutations, and concluded that the optimum number of PCs to remove was 30 PCs for Basalganglia-EUR and Hippocampus-EUR, 60 PCs for Cerebellum-EUR, 40 PCs for Cortex-AFR, 80 PCs for Cortex-EUR, and 20 PCs for Spinalcord-EUR. We then repeated the *cis*-eQTL analyses in each of the eQTL datasets after correcting the optimal number of PCs using 1,000 permutations.

Independent, non-primary *cis*-eQTLs

Since *cis*-eQTL loci are known to often harbor multiple independent associations, we performed an iterative conditional analysis per eQTL dataset. Since our eQTL analyses were performed in a meta-analysis context, our conditional analysis consisted of several steps. From our initial meta-analysis (i.e., the first iteration), we had identified genes with a significant association, and the SNP with the strongest association per gene. For the second iteration of meta-analysis, we first removed the effect of the SNP having the strongest association in each included cohort using linear regression. We then repeated the meta-analysis on the residuals of that regression, focusing on genes that had a significant association in the first iteration. To determine significance of the results, we used the beta-distribution estimates for each gene that were obtained in the first iteration. We used these beta-distributions to adjust the nominal p-values of strongest SNP association per gene, and used calculated the q-value to correct for multiple testing. Genes with a q-value <0.05 were considered significant. We repeated this procedure in consecutive iterations, each time adding the SNP with the strongest association per gene to the linear regression model for that gene, until no significant associations were found. We note that because of the setup of our analysis it could be that variants identified in the second iteration could be in LD with variants of the first iteration, and consequently should not be considered as true secondary eQTL effects. This can happen for example if a variant is present in only a part of the included cohorts. Therefore, after completing all iterations for an eQTL dataset, for each gene, we determined the linkage disequilibrium (LD) between the variant of each iteration and the next iteration, and excluded variants when they had an r2 >0.8, or when no LD could be calculated because of mutually exclusive missingness between included cohorts. We finally re-ranked the remaining variants to determine secondary, tertiary, quaternary, etc. variants.

Trans-eQTLs

Since a genome-wide *trans*-eQTL analysis would result in a large multiple testing burden considering the billions of potential tests, we limited this analysis to a set of 228,819 variants with a known interpretation. This set constituted of variants that were either previously associated with traits, having a genome-wide association studies (GWAS) p-value $<5x10^{-8}$ in the IEU OpenGWAS database[21] and EBI GWAS catalog[22] on May 3rd, 2020, and additional neurological traits (see **Supplementary Table 17**) or were showing an association with q-value <0.05 in any of our discovery *cis*-eQTL analyses (including non-primary associations identified in the iterative conditional analysis). *Cis*-eQTLs in Cortex-EUR were highly concordant when replicated in Cortex-AFR (**Figure 3c**). Consequently, to maximize the sample size and statistical power, we meta-analyzed Cortex-EUR and Cortex-AFR datasets together. However, we omitted ENA, to prevent bias by genotypes called from RNA-seq samples (n=2,759). Furthermore, we created an additional meta-analysis dataset by also excluding AMP-AD samples (n=1,755), to prevent potential bias from the large number of neurodegenerative disease cases in that dataset.

For both these datasets, we first performed a *cis*-eQTL analysis, using the same method as for the other *cis*-eQTL meta-analyses (**Supplementary Table 2**), including a scan for the optimal number of PCs to remove, which were at 100 PCs (including AMP-AD samples) and 80 PCs (excluding AMP-AD samples; **Supplementary Figure 7**). To test for *trans*-eQTLs, we assessed

those combinations of SNPs and genes where the SNP-TSS distance was >5 Mb, or where gene and SNP were on different chromosomes. We note that we did not evaluate eQTLs where the SNP-TSS distance was >1 Mb and <5 Mb, which potentially excludes detection of long-range *cis*-eQTLs or short-range *trans*-eQTLs. We expect however, that this excludes only a limited number of eQTLs, since we observed that this distance was <31 kilobase (Kb) for 50% of *cis*-eQTLs (**Figure 3b**), indicating most *cis*-eQTLs are short-ranged. Additionally, we reasoned that the >5 Mb cutoff would prevent identification of false-positive *trans*-eQTLs due to long-range LD.

The multiple testing method that we applied to the *cis*-eQTLs does not readily translate to *trans*-eQTL analysis. To correct for multiple testing in the *trans*-eQTL analysis, we reperformed the *trans*-eQTL analyses, while permuting the sample labels 10 times. Using the permuted p-values, we created empirical null distributions and determined a false discovery rate (FDR) as the proportion of unpermuted observations over the permuted observations and considered associations with FDR <0.05 as significant. We note that our FDR estimate is evaluated on a genome-wide level, rather than per gene, and consequently FDR estimates stabilize after a few permutations[23]. As with the *cis*-eQTLs, we performed a scan for the optimal number of PCs to remove in the *trans*-eQTL analysis and observed that the number of trans-eQTLs stabilized after 20 PCs were removed (**Supplementary Figure 7**). For consistency with the *cis*-eQTL analysis with these datasets, we therefore chose to remove 100PCs when AMP-AD samples were included, and 80PCs when AMP-AD samples were excluded. Furthermore, we had previously observed that the number of significant *trans*-eQTLs increases when *cis*-eQTLs are removed, therefore, we also corrected the RNA-seq data for primary and non-primary *cis*-eQTLs prior to the final *trans*-eQTL analyses.

Removing cross-mapping artefacts from *trans*-eQTL results

When a *trans*-eQTL gene has similar paralogous genes in the close proximity (<5 Mb) of a given eQTL SNP, the apparent *trans*-eQTL effect may actually reflect a much stronger *cis*-eQTL effect, which might be caused by reads mapping to multiple positions in the genome[17]. While this should be corrected by not counting the RNA-seq reads assigned to multiple genomic features, there might still be some non-detected cases.

To remove such false positive *trans*-eQTLs, we created sets of 35 base pair (bp) "reads" from the human reference genome (GENCODE[24] v32) for each significant (FDR <0.05) *trans*-eQTL gene. To span the gene sequence, we used a shifting window approach, with each consecutive window shifting 2 bp, while also generating reads spanning exon-exon boundaries. We then created 10 Mb sequences centered around each significant *trans*-eQTL SNP. Then, we mapped the reads generated for the gene to the 10 Mb SNP region using BWA-mem[25] v0.7.15 for each *trans*-eQTL SNP-gene pair. We note that we did not explicitly require the reads to map to genes in order to account for potentially unannotated genes and pseudogenes in the genome. Finally, for each *trans*-eQTL, we determined the number of base pairs mapped, and divided this by the number of base pairs generated for the gene, resulting in a proportion of the gene mapped within 5 Mb of the SNP.

As *trans*-eQTLs with high proportions of genes mapping within the SNP region are more likely actually *cis*-eQTL effects, we opted to use 5% of gene mapping to the vicinity of SNP as a threshold to declare *trans*-eQTL to be potentially caused by cross-mapping. To correct the multiple testing threshold for the identified potential false positive *trans*-eQTLs, we then repeated the FDR estimation, leaving out the identified cross-mapping *trans*-eQTLs.

Comparison of eQTL multiple testing correction methods based on permutations

To determine the multiple correction threshold in eQTL analysis, generally a permutation-based strategy is used. Using the p-values obtained after permutation, a null distribution can be created, which is then applied to estimate the FDR. However, there are multiple ways to perform the permutations. For our initial release of eQTL summary statistics (2020-05-26 release), we used the same permutation strategy as was used for a previously published large blood based eQTL meta-analysis by Võsa *et al.*[17], called eQTLgen. For this purpose, we used the eQTL mapping pipeline software (EMP; https://github.com/molgenis/systemsgenetics/tree/master/eqtl-mapping-pipeline).

Contrary to the more commonly used FastQTL[26]/QTLTools[19] (hereafter: FastQTL), which uses a null distribution per gene for multiple testing correction, the EMP approach we used to generate our initial summary statistics calculates a null distribution over all genes, using the top associated variant per gene. Consequently, the resulting FDR estimates can be considered a global, 'genome-wide' multiple testing correction, rather than a local gene-level multiple testing correction. Another comparison of eQTL FDR methods suggested that this can lead to an increase in false positive findings[27]. In the Võsa et al manuscript, we therefore compared the EMP approach with standard multiple testing correction approaches, including Bonferroni correction, and Benjamini-Hochberg (BH) FDR (Supplementary Figure 2 of Võsa *et al.*). There, we observed that at an FDR <0.05 cutoff, our multiple testing correction approach was more stringent than BH-FDR <0.05, but less stringent than a Bonferroni corrected p-value<0.05, suggesting that the FDR estimates our approach provides were reasonable. Furthermore, in general, we found good agreement between significant (FDR <0.05) results in the previous studies that had used the EMP approach and results from other researchers using other approaches.

Here, we opted to also compare results obtained with our pipeline with those obtained from FastQTL using the same input data we used for our initial summary statistics release. For this comparison, we ran FastQTL with default options, using a *cis*-eQTL window of 1Mb, and 1,000-10,000 permutations to estimate the beta-distribution per gene. To determine significance of the FastQTL results, we applied the Storey *et al.*[20] q-value method (implemented in the R package 'qvalues') over the top associations per gene using a lambda of 0.85, as previously used in the GTEx eQTL pipeline (https://github.com/broadinstitute/gtex-pipeline), considering genes with a q-value < 0.05 as significant. 14,910 of the 18,451 tested genes were significant with this approach. First, we compared the top p-value per gene, and observed that EMP and FastQTL produce highly similar p-values (**Supplementary Figure 35a**), which is to be expected as both methods are based on correlation. However, when comparing FDR estimates, we observed that

the -$\log_{10}$ q-values produced by FastQTL were on average higher than the -$\log_{10}$ FDR estimates produced by EMP, resulting in more significant results for the FastQTL method (**Supplementary Figure 35b**). As such, we concluded that the EMP FDR approach produces on average more conservative results than FastQTL.

One reason for applying our EMP approach for our initial summary statistics is that this approach is able to perform eQTL meta-analysis across multiple datasets at once. We reasoned a meta-analysis approach is suitable for the *MetaBrain* eQTL datasets since they are comprised of samples from different studies, that were collected and generated at different institutes. Apart from our normalization approach, which corrects for major dataset differences, we reasoned that a meta-analysis approach would be best suited to account for potential residual dataset differences. FastQTL, however, does not have the ability to perform such a meta-analysis directly. While meta-analysis can be performed using FastQTL summary statistics from non-permuted data, this is less straightforward for the permuted results: when meta-analyses across 1,000 permutations need to be performed, the resulting summary statistics would require a large amount of disk space and compute time.

To solve this issue, we implemented the FastQTL multiple testing approach in a meta-analysis framework (mbQTL; https://github.com/molgenis/systemsgenetics/tree/master/mbQTL). For each gene, we performed sample size weighted meta-analysis across datasets for all SNPs within the 1Mb *cis*-window surrounding the gene and stored the SNP having the lowest p-value as the nominal p-value. We then repeated this meta-analysis 1,000 times, while permuting the sample labels, and kept the lowest p-value per permutation. Like in FastQTL, we used these 1,000 permuted p-values to fit a beta-distribution, which was used to adjust the nominal p-value. We then applied the Storey *et al*. q-values approach, using a lambda of 0.85, to calculate q-values for each gene, using the beta-distribution adjusted p-values as input. eQTLs with a q-value < 0.05 were considered significant. 14,925 out of the 18,417 tested genes were significant, which was very comparable to the FastQTL result (14,910 out of 18,451). Compared to FastQTL, we observed that the -$\log_{10}$ q-values produced by FastQTL were slightly higher than those produced by mbQTL, especially for smaller -$\log_{10}$ q-values (**Supplementary Figure 35c and d**).

Based on the q-value comparison, we hypothesized the mbQTL p-values were more stringent. We, and others, have observed previously that the SNP with the strongest association to a gene is generally located nearby the TSS. For example, 85% of the *cis*-eQTL SNPs in the eQTLgen study were located within 100 kb of the TSS. So, we evaluated the relationship between TSS distance and the significance cutoff for the three methods, by ranking all tested genes by p-value (lowest to highest), and plotting the TSS distance, which shows a relationship between significance and average TSS distance (**Supplementary Figure 35e**): highly significant eQTLs generally have a small TSS distance, which progressively becomes larger as significance decreases. We observed that the TSS distances were very comparable with the eQTLgen study, for EMP (79% of significant eQTLs <100 kb) and for both FastQTL (70% of significant eQTLs <100 kb) and mbQTL (70% of significant eQTLs <100 kb). We therefore reasoned that the multiple testing correction in mbQTL produced reasonable estimates of significance.

We have therefore repeated all *cis*-eQTL discovery analyses using the mbQTL method, performing 1,000 permutations, and using a similar beta-approximated null distribution as implemented in FastQTL. Consequently, together with the other adjustments made to the analysis compared to our previous summary statistics release, the number of significant eQTLs is now 1,880 (was 1,317) for Basalganglia-EUR, 10,577 (was 6,865) for Cerebellum-EUR, 16,169 (was 11,803) for Cortex-EUR, 4,797 (was 5,440) for Cortex-AFR, 1,265 (was 990) for Hippocampus-EUR, and 998 (was 811) for Spinalcord-EUR.

MAPT and the influence of patch sequences in GRCh38 on RNA-seq quantification

The eQTL association in the MAPT region is somewhat controversial: the xqtl browser[28] (mostafavilab.stat.ubc.ca/xqtl/), which is constructed using ROSMAP and CMC studies, finds very strong effects on the *MAPT* locus. The *MAPT* eQTL has also been found in the newer and much larger PsychENCODE study (n~1,400; resource.psychencode.org/), but curiously cannot be found in the newest GTEx release (gtexportal.org/) nor in the latest meta-analysis from Sieberts *et al.*[29], where ROSMAP is included as well (summary statistics are found here: synapse.org/#!Synapse:syn16984815). Initially, the *MAPT* eQTL was also not identified in our meta-analysis.

One of the reasons this can happen is because the *MAPT* region is represented by multiple contigs/patch/alt sequences. Each of these sequences contains a full copy of the *MAPT* gene (i.e., ENSG00000276155 and ENSG00000277956), which confuses alignment and gene quantification software when these patch sequences are kept in the genome reference (they were excluded in the latest GTEx release). As a consequence, read counts for these alternate gene copies correlate very strongly with genotype, introducing an eQTL (**Supplementary Figure 9**). After rerunning the alignment without the patch chromosomes, we now did identify the MAPT eQTL.

Genetic association at the *MAPT* locus is inconsistent[30]. Even after re-aligning to the primary genome to exclude the patch chromosomes, there is a lot of heterogeneity in signal left between datasets. The haplotypic signal is present after meta-analysis, but mostly comes from CMC and ENA, with the effect in other datasets being much lower (**Supplementary Figure 10**). Additionally, co-localization at the *MAPT* locus is difficult due to the *MAPT* haplotypes with many SNPs in high LD in the region.

Properties of primary, secondary, tertiary, and quaternary *cis*-eQTLs

In blood, we have shown recently genes with the highest expression are most often *cis*-eQTL genes, *cis*-eQTL SNPs are generally located within 50 Kb of the TSS and genes without a *cis*-eQTL have low tolerance for loss of function mutations[17]. In contrast, after we ranked genes by average expression in Cortex-EUR, we observed that genes within the second decile are most likely *cis*-eQTLs, rather than those in the 10th decile. Nevertheless, as in blood, we observed that the median expression level of primary eQTL genes (median $\log_2$(TMM) expression=2.588) was significantly higher than that of genes without a *cis*-eQTL (median expression=1.577, Wilcoxon

p-value=$5.5x10^{-174}$; **Figure 3b**). Comparing genes having only a primary eQTL association (median expression=2.557) with those having a secondary eQTL, we observed that genes with a secondary eQTL generally had slightly higher median expression level (median log_2(TMM) expression=2.607; Wilcoxon p-value=$3.49x10^{-14}$). Genes with additional independent eQTL associations showed a further decrease in median expression level (**Figure 3b**).

When evaluating the distance to TSS, we observed that the distance between the eQTL variant and the TSS shifts further away with additional independent eQTL associations: primary eQTLs had a median distance of 34 Kb, secondary eQTLs a median distance of 47 Kb, tertiary eQTLs a median distance of 61 Kb, and quaternary eQTLs a median distance of 77 Kb (**Figure 3b, middle**). This shift in distance between eQTL variant and TSS was smaller than that of previously published results in cortex[31], potentially due to the larger sample size of *MetaBrain* resulting in an increase in power to detect secondary associations. Like observed previously in blood, genes with low tolerance for loss of function mutations, as indicated by the pLI score, were less likely to be eQTL genes[17]: primary eQTL having lower pLI scores than those without (χ^2 p=$2.4x10^{-83}$). This effect was smaller but still significant for secondary (χ^2 p=$3x10^{-2}$), but not for tertiary and quarternary eQTLs (χ^2 p>0.05, **Figure 3b, right**).

It has been shown that in blood. with a large sample size, 95% of highly expressed genes have at least one independent eQTL effect, and that highly expressed genes that are not an eQTL have low tolerance to loss of function variants[17]. To test if this is also true for genes that are highly expressed in cortex, we calculated the mean and standard deviation over the log_{10}(TMM normalized expression+1) table (before quantile normalization and covariate correction) for all samples that were included in the Cortex-EUR eQTL dataset. We then subset the genes to only those that were tested for being an eQTL at each iteration (For primary eQTLs: only protein coding genes, for secondary eQTLs: only significant primary eQTL genes, for tertiary eQTLs: only significant secondary eQTL genes, for quaternary eQTLs: only significant tertiary eQTL genes). For each iteration, we divided the genes up in 10 bins, ranking the genes from low to high expression. For each bin, we calculated the proportion of genes that had a significant eQTL, and observed that the 4th decile had the most (**Supplementary Figure 11a**), with a drop in proportion of eQTLs for the 9th and 10th expression bin. To test if this was due to the fact that the genes in the bins with highly expressed genes had a lower standard deviation, we also determined the standard deviation per bin, and compared this to the standard deviation per bin of protein coding genes in BIOS[1], a blood eQTL dataset (**Supplementary Figure 11b**). Comparing between bins, we did not see difference in standard deviation for higher expressed bins in either *MetaBrain* or BIOS.

Functional enrichment showed little difference between the types of eQTLs. Using gProfiler[32] we determined enrichment of genes that only had a primary eQTL signal and genes that had multiple independent eQTL signals for GO[33], KEGG[34], Reactome[35], WP[36], TF[37], MIRNA[38], HPA[39], and HP[40]. For most of these enrichments we found around 50% overlap in enrichments between primary and non-primary eQTL genes. Enrichments that differed between the groups were mostly unspecific (e.g. protein transport, nitrogen compound transport). Notable, however,

is that of the 288 transcription factors enriched for targeting non-primary eQTLs, 286 (99.3%) are also enriched for primary eQTLs, out of the total of a total of 577 enriched transcription factors targeting primary eQTLs. (**Supplementary Figure 11c and d, Supplementary Table 4**).

Enrichment of primary vs non-primary *cis*-eQTL genes

Enrichment of primary and non-primary *cis*-eQTLs was done using g:Profiler[32] (Ensembl 103, Ensembl Genomes 50 (database built on 2021-04-01)) with default parameters and protein coding genes as background geneset.

Cortex eQTL concordance and heterogeneity between datasets

Cis-eQTL effect directions were highly concordant between datasets included in the Cortex-EUR meta-analysis (median Spearman r=0.77; median allelic concordance (AC)=83%; **Supplementary Figure 8a-d**), and the majority (between 68% and 76%) showed low to moderate heterogeneity (I^2<50%**; Supplementary Figure 8e, Supplementary Table 2**), indicating robustness of the identified effects across datasets.

eQTL agreement

eQTL agreement calculations

We have used four different measurements of agreement of eQTL effects when comparing between different brain regions or tissues: AC, π_1, R_b, and correlation of allelic fold change (caFC)[41]. Each of these measures evaluates different aspects of replication: AC is an indication of the proportion of effects that have a shared direction of effect within the set of eQTLs that is significant in both discovery and replication dataset and is expected to be 50% for random eQTL effects, π_1[42] estimates the proportion of eQTL effects that are true positive in the replication cohort, but does not take into account effect direction and can be dependent on replication dataset sample size, R_b[43] effectively estimates the correlation between eQTL effect slopes (e.g.: betas from linear regression), while controlling for potential covariance in standard errors of those slopes, and caFC measures the correlation between estimates of the fold change in expression values between alleles[41]. We note that while AC, π_1, and R_b can be calculated from summary statistics, caFC requires access to genotype and expression data. We therefore limited the caFC analysis to comparisons within the *MetaBrain* datasets, and to comparisons with the GTEx tissues.

To calculate AC, we took the SNP-gene combination with the lowest p-value in the discovery cohort for each gene if it was significant and matched these with the same SNP-gene pairs in the replication cohort if it was also significant. We then determined the percentage of those significant SNP-gene pairs had the same allelic direction of effect compared to the discovery cohort. To calculate π_1 we took the SNP-gene combination with the lowest p-value in the discovery cohort for each gene if it was significant, ordered the SNP-gene combinations from lowest to highest p-value, and matched these with the same SNP-gene combination in the replication cohort. SNP-gene combinations not tested in the replication cohort were removed. We then calculated the proportion of true null p-values (π_0) with the pi0est function of R, with

parameter p = the p-values of the match SNP-gene combinations in the replication cohort, and calculated π_1 with 1- π_0. Similarly, we calculated R_b by taking the SNP-gene combination with the lowest p-value in the discovery cohort for each gene if it was significant, matching those to the replication dataset, regardless of significance in the replication dataset. As an additional analysis, we performed R_b analysis while focusing on eQTL SNPs identified in the GTEx muscle tissue, to prevent potential biases caused by detection of eQTL SNPs in the same type of tissue, as described in the original R_b manuscript.

Finally, we applied a similar procedure to calculate caFC, by taking the SNP-gene combination with the lowest p-value in the discovery cohort for each gene if it was significant, matching those to the replication dataset, regardless of significance in the replication dataset. In *MetaBrain* datasets, we calculated allelic fold changes (aFC) using gene expression data that was $\log_2$ transformed and corrected for technical covariates and the suitable number of PCs for that dataset. We used Pearson correlation to calculate the correlation between the aFC estimates of two datasets. While for GTEx, we downloaded the GTEx-v8 normalized expression matrices and covariate matrices for each tissue from the GTEx portal website, after which we $\log_2$-transformed and corrected the gene expression matrix for each tissue using the covariates by ordinary least squares (OLS).

***Cis*-eQTL agreement between tissues and ancestries**

Tissue specific[44], brain region specific[29], and to a lesser extent, population specific[45] eQTLs have been observed in previous reports. Our dataset allowed us to evaluate the influence of these factors on eQTL agreement, by comparing the discovered eQTLs between different brain tissues and ancestries.

We examined eQTLs discovered in the Cortex-EUR and Cortex-AFR datasets and had access to a smaller East-Asian dataset (Cortex-EAS; n=208, limited to ENA dataset). Due to limited sample size, we observed fewer eQTLs in AFR (4,797) and EAS datasets (313). However, concordance in allelic direction was high (>92.95%) if the eQTL was significant (FDR <0.05) in both datasets (**Supplementary Figure 12**). Due to the limited sample size and genotype density of Cortex-EAS, concordance rates were generally lower for that ancestry. We observed similar trends for both the R_b estimates. The π_1 estimates rather showed that eQTLs detected in a large dataset (e.g. Cortex-EUR) are generally not significant in a smaller dataset such as Cortex-EAS. These results indicate that eQTLs from the same tissue generally share the same allelic direction across ancestries and that sample size strongly determines how many *cis*-eQTLs can be found and replicate significantly in another ancestry.

We next compared the different brain region datasets for the EUR ancestry. Again, we observed high concordance of allelic directionality (>91.05%; **Supplementary Figure 12**). Cerebellum had overall lower concordance with other brain regions (91.05%, 95.96%, 96.03%, 96.81% for Cortex-EUR, Basal ganglia, Hippocampus, and Spinal cord, respectively). We observed similar behavior as with the comparison between ancestries, where Rb estimates were generally high, but π_1 estimates greatly depended on sample size of the replication dataset. We therefore

reasoned differences with cerebellum might be independent of sample size, since cerebellum has the second largest sample size in *MetaBrain*. Rather, this suggests a difference in genetic regulation between cerebellum and other tissues of the brain.

We identified 5,767 *cis*-eQTL genes unique to Cortex-EUR, and 477 *cis*-eQTLs for Cerebellum-EUR that were not significant in Cortex-EUR (**Supplementary Figure 13a**). The large number of genes unique to Cortex-EUR is likely due to the much larger sample size of this dataset compared to the other *MetaBrain* eQTL discovery datasets: we expect that if the sample size of the other datasets would have been larger, the number of significant genes for those datasets would increase, and likely also the overlap of significant genes between datasets. Interestingly, however, we did observe many genes unique to Cerebellum-EUR, which consequently is less likely due to the sample size of this dataset. We therefore focused on the Cerebellum-EUR unique genes. For some of these genes, it is likely that they are a Cerebellum-EUR specific eQTL because they are expressed in cerebellum and not in cortex.

We observed that the expression levels of these 477 Cerebellum-EUR eQTL genes was binomially distributed in cortex, and assigned 185 genes on the left side of the minima of this binomial distribution as low-expressed cortex genes, and 292 genes on the right as high-expressed cortex genes (**Supplementary Figure 13b**). We observed that of the 477 genes, 398 (83.43%) had higher expression levels in cerebellum (**Supplementary Figure 13c**, blue line indicates the cut-off between low and high-expressed cortex genes). We took the 477 genes that are unique eQTL genes for Cerebellum-EUR, but high-expressed in cortex and used g:Profiler[32] to perform functional enrichment (**Supplementary Table 6**).

This analysis included TRANSFAC[37] enrichment of transcription factor sites around the eQTL genes. We used these enrichments to determine whether genes that are highly expressed in cortex but only have a *cis*-eQTL in cerebellum show a difference in enrichment for specific transcription factor binding sites. We therefore extracted all transcription factors from the g:Profiler table and because transcription factors can be enriched multiple times for different binding sites, we deduplicated the results. We converted the TRANSAC IDs to ENSEMBL IDs using GeneCards[46] and plotted the expression of each transcription factor in cerebellum and cortex (**Supplementary Figure 13d**). Three of these transcription factors (*EOMES, HES7,* and *IRX5*) are lowly expressed in cortex and highly expressed in cerebellum, and these could further explain why some of their target genes are eQTLs in cerebellum and not in cortex, while being expressed in both.

We next evaluated whether the high rate of sharing between tissues was specific to *MetaBrain*: we repeated the Cortex-EUR discovery while omitting GTEx samples, and subsequently attempted replication in the different GTEx tissues[47]. Cerebral and cortex regions of the brain had highest directional agreement with Cortex-EUR eQTLs (AC >96%, $R_b > 0.9$), and overlap of significant effects was highest with cortex tissues (π_1=0.69), while concordance in cerebellum

was more comparable to other, non-brain, tissues (**Figure 3d, Supplementary Figures 12 and 14; Supplementary Table 5**). π_1 estimates were comparable between GTEx tissues, potentially due to the comparable sample sizes between tissues within GTEx. The overall AC was the lowest in testis (78%, R_b=0.68) and whole blood (80%, R_b=0.72). We next evaluated whether the lower concordance in blood was due to the limited sample size of GTEx, by determining the number of concordant Cortex-EUR eQTLs in eQTLgen, a large blood-based dataset (n=31,684). This resulted in an AC of only 75%, an R_b of 0.52 and a π_1 of 0.83 (**Supplementary Figure 15**): thus 25% of the shared eQTLs between blood and brain show opposite allelic effects, while the majority of the Cortex-EUR eQTLs can be detected significantly in blood. We conclude that when eQTL cohort sample-sizes become large it becomes apparent that there is strong tissue-specific regulation, which can explain these opposite allelic effects[48].

Cell type deconvolution and ieQTL analysis

Estimation of cell type proportions

By leveraging cell type specific gene expression collected through scRNA-seq, a bulk tissue sample can be modelled as a parts-based representation of the distinct cell types it consists of. In such a model, the weights of each part (i.e.: cell type proportions) can be determined by deconvolution. In the deconvolution of the *MetaBrain* bulk expression data we used the TPM single-cell derived signature matrix published by the PsychENCODE consortium[49]. We TPM normalized the gene expression of the *MetaBrain* bulk RNA-seq cortex samples and extracted the 418 unique signature genes included in the PsychENCODE single-cell signature profile. We then applied $\log_2$ transformation on both the signature matrix as well as the bulk gene count matrix. Then, to enable the joint analysis of samples, we corrected the gene counts for 20 RNA-seq quality metrics using OLS. To maintain the information captured by relative expression differences between genes required for deconvolution, we rescaled the residuals to the original $\log_2$ transformed mean and standard deviation and replaced negative values with zero. Subsequently we applied non-negative least squares (NNLS)[50] using SciPy (version 1.4.1)[51] to model the bulk expression as a parts-based representation of the single-nucleus derived signature matrix. First introduced by Lawson and Hanson[50], the NNLS method is the basis of numerous deconvolution methods to date. In short, NNLS attempt to find a non-negative weight (coefficient) for each of the cell types that, when summed together, minimizes the least-squares distance to the observed gene counts. Lastly, we transformed the resulting coefficients into cell type proportions by dividing them over the sum of coefficients for each sample.

Since the average proportions of sub cell types were often very low, we opted to sum all the subtypes of cells for excitatory neurons, inhibitory neurons and oligodendrocytes (OPC and oligodendrocytes). After which, excitatory neurons were the most abundant cell type (average cell proportion: 28.1%), followed by astrocyte (21.4%), other neuron (16.4%), oligodendrocytes (14.5%), endothelial cells (12.2%), microglia (6.4%), and inhibitory neuron (1.1%; **Supplementary Figure 16b**). We observed that the first ±10 expression PCs correlated strongly with predicted cell type proportions for specific cell types (**Supplementary Figure 36**). We then correlated the cell type proportions with immunochemistry (IHC) counts from the ROSMAP

cohort[52]. It is however difficult to validate these cell type proportion predictions due to the small scale of the IHC experiment, but also because IHC and bulk RNA-seq reflect different aspects of gene or protein expression. Thus, there is a level of uncertainty for the expected proportion for each cell type[53,54].

Identification of cell type dependent eQTLs

With the predicted cell type proportions, we aimed to detect cell type interaction eQTLs (ieQTLs). For this we used Decon-QTL[55] to systematically test for significant interaction between each cell type proportion and genotype, while also controlling for the effect on expression of the other cell types. Whereas the original Decon-QTL limits the number of genotypes that have an opposite genotypic encoding to one interaction term, we enabled testing of all possible allele encodings to increase accuracy. For this we took the TMM expression counts, applied a $\log_2$ transformation and subsequently corrected for dataset indicator variables, 20 RNA-seq alignment metrics, and 4 genotype multidimensional scaling (MDS) components using OLS. As an additional step, we forced the data to the normal distribution per gene to reduce outliers. Finally, we inverted the $\log_2$ transformation. In our study, Decon-eQTL uses the sample size minus numbers of terms in the model as the degrees of freedom (i.e.: number of cell types times two). Since the predicted cell type proportions add up to 1, an argument could be made that one less degree of freedom should be used. We observed that this difference of one degree of freedom did not make a difference in the observed p-values per eQTL interaction (average correlation between p-values, r=1), potentially due to the sample size of our dataset. However, we note that this difference may become more significant in studies with smaller sample sizes (e.g.: <100 samples). The p-values calculated by Decon-QTL are corrected for multiple testing using BH on a per-cell-type basis.

Determining significance of ieQTLs through permutations

To test if the multiple testing strategy (Benjamini-Hochberg FDR; BH-FDR) applied in our study properly reflects the actual null distribution, we implemented a permutation-based approach in Decon-QTL. However, permuting interaction effects is not trivial. Suppose a simple linear regression model of $y \sim g + c + g*c$, where y is the expression of a gene, g is the genotype term, c is the cell type proportion to be studied, and g*c is the cell type interaction term. This model captures the relationship of these individual terms on gene expression, but not the individual terms which are often correlated as well. Permuting the y variable in this model would create a null distribution that determines the probability of the whole model (e.g. including the main g and c effects), and thus would not be suitable to estimate the significance of the interaction term. Furthermore, permuting the individual g, or c terms would result in the g*c term capturing a part of the effect that was lost due to permuting the main effect terms. Similarly, permuting just the interaction term g*c might cause a deflation of the permuted p-values (i.e. an over-representation of high p-values), because of the presence of the g and c terms in the model. Furthermore, Decon-QTL model is more complex than the example above, since it includes a term for each cell type and their interactions. The constraint in NNLS model requires the beta estimates for each of the permuted term to be positive, a requirement that cannot always be met during permutation, causing a further deflation of p-values.

To test if a permutation-based approach is feasible, we implemented an approach in Decon-QTL which permutes the genotype of one interaction term and then determines the empirical null distribution per cell type using 1,000 permutations. As expected, we observed that the NNLS method that Decon-QTL applies results in a deflation of p-values (**Supplementary Figure 37**). For the permuted p-values this tendence towards p-values equal to 1 is even more extreme, with 50% of all p-values >0.9692. Since the permuted p-values are not uniformly distributed, we were unable to apply a multiple testing correction step (e.g. q-values) analogous to the FastQTL approach. We therefore calculated the permutation FDR by determining the number of permuted p-values smaller or equal to the nominal p-values divided by the nominal p-value rank. We compared the results to the BH-FDR results and observed that the majority (87.76%) of the BH significant findings were also significant using this approach (**Supplementary Figure 38**).

Replication of cell type dependent eQTL effects

In order further confirm cell type specific eQTL effects identified in Cortex-EUR, we used three independent datasets: Cortex-AFR, ROSMAP single-nucleus[49], and Bryois *et al.*[56].

For the Cortex-AFR replication we used the Cortex-EUR eQTLs and applied the same procedure as described above for the prediction of cell type context and identification of ieQTLs. We calculated a BH-FDR on the p-values of the ieQTLs that were significant in Cortex-EUR in the respective cell type. Since Decon-QTL does not return any standard errors, we predicted beta and standard errors using the sample size, MAF, interaction beta, and interaction p-value[57] in order to calculate R_b metrics. We observed moderate agreement ($-0.17<R_b<0.98$, median R_b: 0.77, $0.00<\pi_1<0.06$, median π_1: 0.0, 43%<AC<72%, median AC: 0.67; **Supplementary Table 9, Supplementary Figure 17b**), which was highly dependent on the average predicted cell type proportion. The $\pi 1$ estimates especially suggest that the analysis in the Cortex-AFR dataset is somewhat underpowered. When focusing on the 177 ieQTLs that were significant in both datasets, all cell types had an AC>97% except inhibitory neurons, which had an AC of 62%.

We then replicated our findings in the ROSMAP single-nucleus data, encompassing 80,660 single-nucleus transcriptomes from the prefrontal cortex of 48 individuals with varying degrees of Alzheimer's disease pathology[58]. We used Seurat version 3.2.2[59] to analyze the data. First, we removed the genes that did not pass filtering as described previously[58], leaving us with 16,866 genes and 70,634 cells for further analysis. After this, we normalized the expression matrix on a per individual per cell type basis using sctransform[60] and visualized the normalized expression matrix using UMAP dimensionality reduction[61]. We observed that cell types, as defined by Mathys *et al*[62]*.,* for the majority cluster together (**Supplementary Figures 39 and 40**). We then created expression matrices for each broad cell type (excitatory neurons, oligodendrocytes, inhibitory neurons, astrocytes, oligodendrocyte precursor cells, microglia, pericytes and endothelial cells) by calculating the average expression per gene and per individual basis. We then used these cell-type datasets for eQTL mapping using the same procedure as the *trans*-eQTL analysis in bulk data. To correct for multiple testing, we confined the analysis to only test for primary *cis*- and *trans*-eQTLs that had a significant interaction with one or more cell types in

MetaBrain Cortex-EUR (BH-FDR <0.05), while also permuting the sample labels 100 times. For the calculation of the R_b metrics we used the beta and standard error of the eQTL effect in the respective cell type. We compared the allelic direction of the ieQTL in Cortex-EUR with the eQTL direction in matching cell types and observed good agreement for most cell types ($0.13<R_b<0.82$, median R_b: 0.68, $0<\pi_1< 0.63$, median π_1: 0.36, 48%<AC< 81%, median AC: 0.65; **Supplementary Table 9, Supplementary Figure 18b**). A total of 173 *cis*-eQTLs were significant in the Cortex-EUR and the ROSMAP snRNA-seq datasets of which 84 eQTLs (43 for oligodendrocyte and 41 for excitatory neuron) were dependent on the corresponding cell type in bulk with a 100% AC (**Supplementary Figure 18b**).

Finally, we replicated our findings using the single nucleus RNA-seq eQTL summary statistics of the recent preprint by Bryois *et al.*[56] In short, Bryois *et al.* mapped eQTLs in 8 cell types (excitatory neurons, oligodendrocytes, inhibitory neurons, astrocytes, oligodendrocyte precursor cells / committed oligodendrocyte progenitors, microglia, pericytes and endothelial cells) using data from 196 adult human brain tissues collected from both cortical grey as well as deep white matter. We overlapped their summary statistics with the Cortex-EUR *cis*-eQTLs and found, dependent on the cell type, that between 9,402 and 13,764 overlapped. We calculated a BH-FDR on the p-values of the ieQTLs that were significant in Cortex-EUR in the respective cell type. Since the summary statistics did not include standard errors nor MAF values, we predicted beta and standard errors using the *MetaBrain* Cortex-EUR MAF together with the eQTL sample size, beta, and p-value[57] from Bryois *et al.* to calculate R_b metrics. Comparing to Bryois *et al.*, we were able to test 54% of ieQTLs (1,734 out of 3,209, not considering other neuron), with a moderate to high agreement ($0.78<R_b<0.86$, median R_b: 0.84, $0.43<\pi_1<0.83$, median π_1: 0.69, 81%<AC<90%, median AC: 0.9), except for inhibitory neurons which had a lower agreement ($R_b=0.23$, $\pi_1=0.71$, AC=63%). Of the overlapping ieQTLs, 1,070 (62%) significantly replicated in the corresponding cell type with AC>94% (except inhibitory neurons; AC=71%; **Supplementary Table 9, Supplementary Figure 19b**).

Cis-eQTL LD overlap

We evaluated whether the detected *cis*-eQTLs could be linked to neurological traits and diseases, using three different approaches: we determined LD overlap, we performed a Mendelian Randomization (MR) approach and we performed statistical colocalization.

First, we investigated LD overlap between *cis*-eQTL SNPs showing the strongest association per gene (i.e. the index eSNP) and 37,759 variants previously identified in 1,736 GWAS result sets for neurological traits. For this analysis we focused on *MetaBrain cis*-eQTL discovery datasets from EUR ancestries, since most GWAS studies are limited to that ancestry. For each of the eQTL discovery datasets, we first clumped these GWAS SNPs into clusters by grouping all SNPs within 5Mb of each other when their $R^2 > 0.2$, using the genotypes for each eQTL discovery dataset to calculate LD. For each cluster, we then determined the LD between each member GWAS SNP and the top eQTL variants when their distance was < 5Mb. For each of the clumped GWAS regions, we reported the linked eQTL index SNPs in **Supplementary Table 10**.

Focusing on the Cortex-EUR *cis*-eQTLs, 1,915 (15%) of the 13,129 trait SNP clusters were in LD (r^2 >0.8, distance between SNPs <5 Mb, LD calculations performed in Cortex-EUR) with at least one eSNP, linking a total of 2,665 eQTL genes. Primary *cis*-eQTL SNPs provided the majority (n=1,381; 72%) of the linked GWAS clusters. Out of the 15,895 unique primary Cortex-EUR *cis*-eQTL SNPs, 1,980 (12%) were in LD with at least one GWAS SNP, while 881 (5%) out of 17,100 non-primary Cortex-EUR *cis*-eQTL SNPs were linked to at least one GWAS SNP. Consequently, we observed that primary eQTL SNPs were 2.6-fold more likely to be in LD with an GWAS SNP compared to non-primary eQTL SNPs (Fisher exact test p-value: $7.38x10^{-125}$). In Cortex-EUR, 73 GWAS result sets had 10 or more clusters in high LD, including body mass index (1139 clusters, 15%), insomnia (75 clusters, 14%), intelligence (57 clusters, 21%), depression (57 clusters, 19%), multiple sclerosis (30 clusters, 21%), schizophrenia (29 clusters, 18%), and Parkinson's disease (24 clusters, 30%; **Supplementary Table 10**).

Investigating LD overlap in Cerebellum-EUR, we observed smaller numbers: including non-primary *cis*-eQTLs, 1,141 (9%) out of 12.802 GWAS clusters were in LD with a least a single *cis*-eQTL SNP, reflecting 1,551 genes. Again, primary *cis*-eQTLs comprised the majority of these overlaps, with 987 (87%) GWAS clusters linked to a primary *cis*-eQTL SNP. There were 50 traits for which at least 10 GWAS clusters were in LD. These included body mass index (701 clusters, 9%), educational attainment (176 clusters, 8%), depression (32 clusters, 11%), neuroticism (18 clusters, 7%), schizophrenia (17 clusters, 11%), and Parkinson's disease (15 clusters, 19%; **Supplementary Table 10**).

Our LD overlap analysis indicates that up to 15% of brain-related GWAS loci can be linked to a *cis*-eQTL variant when using 2,683 samples. While this low overlap could be caused by incomplete power in the GWAS and eQTL studies, we believe this to be unlikely: in our recent study in whole blood using 31,684 individuals[17], the proportion of overlapping variants was 29.3%, indicating that a ten-fold increase in sample size would yield at most a two-fold increase in LD overlap. Nevertheless, increasing the sample size of bulk eQTL studies could improve future fine-mapping efforts, which may help efforts to link disease SNPs to genes[63]. We note that LD overlap has known limitations that may cause biased overlap estimates, including coincidental sharing of the association with the GWAS trait due to surrounding LD patterns in the genomic region, or lack of sharing due to uncertainty about the location of the causal variant.

Recently, it has been suggested that GWAS and eQTL variants have different distances towards the TSS and overlap with different kinds of regulatory elements[64]. It could therefore be that GWAS associations are better captured by non-primary eQTL effects, which are generally located further from the TSS. Our LD overlap analysis suggested a limited increase in overlap using non-primary eQTLs, but we also note that identifying such secondary eQTL effects could be improved by further increasing sample sizes of bulk RNA-seq studies, especially for eQTLs with small effect size.

Mendelian randomization (MR) and colocalization

Methods

Annotating suggestive MR findings

Methods for performing MR and colocalization are found in the main manuscript. In addition, we also compared the suggestive MR findings with two catalogues of Mendelian diseases, Development Disorder Genotype - Phenotype Database (DDG2P) and OrphaNet. Because we focused on neurological traits for MR, only the developmental disorders that affected Brain/Cognition in DDG2P, as specified in the "organ specificity list" column, were annotated[65] (data freeze 2020-12-16). We looked for genes that appear to follow an allelic series[66].

To annotate with OrphaNet data, the November 2020 release of XML files were downloaded from their GitHub repository[67], and Python package xmltodict[68] was used to aid in data parsing. Information was extracted from the following files and combined on OrphaNet ID via pandas[69] rare disease epidemiology, rare diseases natural history, genes associated with rare diseases and classifications of rare diseases.

Taylor expansion for the Wald ratio standard error

To account for the error in both the instrument-exposure and instrument-outcome when comparing Wald ratio (WR) on the same SNP instrument we used the two term Taylor expansion for computing the standard error (SE) of the WR. Let WR_{XY} denote the Wald ratio between the exposure (X, eQTL) and outcome (Y, multiple sclerosis) for a SNP instrument. Let B_{XY} and SE_{XY} denote the effect size and standard error of the eQTL effect and B_{ZY} and SE_{ZY} denote the same for the outcome SNP effect.

For the primary MR analysis in our study, we used the first term expansion only which accounts for the variance in the outcome SNP effect. Note: the contribution of the eQTL error to the WR SE will be almost negligible as highly significant eQTLs are selected ($p<5x10^{-8}$ in this study).

In the first term approximation, the Wald ratio is estimated as:

$$WR_{XY} = \frac{\beta_{ZY}}{\beta_{ZX}}$$

with variance:

$$Var(WR_{XY})_1 = \frac{{SE_{ZY}}^2}{{\beta_{ZX}}^2}$$

However, we can also expand the series by another term to account for the variance in the instrument-exposure (eQTL) effect:

$$Var(WR_{XY})_2 = Var(WR_{XY})_1 + \frac{\beta_{ZY}{}^2 SE_{ZX}{}^2}{\beta_{ZX}{}^4}$$

and obtain the standard error for this two-term expansion:

$$SE(WR_{XY})_2 = \sqrt{Var(WR_{XY})_2}$$

Results

Suggested MR findings (Wald ratio $p<5x10^{-5}$) for the disease trait outcomes and brain volume outcomes are reported in **Supplementary Table 12**. The p-value threshold for significance after adjusting for 348,449 tests performed is $1.43x10^{-7}$.

Alzheimer's disease

There were 23 significant Wald ratios for Alzheimer's disease[70] (AD), of which 8 passed colocalization (PP4 >0.7). Higher expression of five genes (*CR1*, *TSPAN14*, *ZNF668*, *INPP5D* and *APH1B*) and lower expression of three genes (*SLC39A13*, *PRSS36* and *ACE*) were associated with increased AD risk.

CR1 is a top-ranked AD risk gene expressed by microglia and involved in amyloid-b (Aβ) clearance. Previous studies have identified CR1-B/S allele, coding the longer isoform with additional C3b binding site, associated with increased AD risk[71,72]. Some evidence suggested increased activity of *CR1* can potentially inhibit complement cascade activated by Aβ and lead to enhanced Aβ deposition[73,74]. Consistently, we found higher expression of *CR1* associated with increased risk of AD (Wald ratio or WR=0.15, $p=1.4x10^{-23}$). However, the precise role of CR1 in AD pathogenesis is still unclear.

TSPAN14 resides in a recently identified AD risk locus[70], the protein it encodes belongs to the TspanC8 family of tetraspanins and interacts with ADAM10, which is encoded by another AD risk gene and mediates proteolytic cleavage of more than 40 substrates including APP and TREM2[75]. Overexpression of *TSPAN14* has been shown to increase cell surface expression of ADAM10[76]. Our MR and colocalization analysis using rs1902660 as an eQTL instrument variable found higher expression of *TSPAN14* as putatively causal for AD (MR Wald ratio=0.12, $p=4.7x10^{-9}$; Coloc PP4>0.95). Missense mutations in *ADAM10* that attenuate its α-secretase activity shift APP processing toward β-secretase-mediated cleavage, increase Aβ plaque load and reactive gliosis[77]. However, it is not well understood how cleavage activity of APP by ADAM10 is impacted by expression of *TSPAN14*, though some *in vitro* evidence showed decreased APP-ADAM10 cleavage by stably expressing *TSPAN14* in U2OS-N1 cells[76]. ADAM10 also mediates cleavage of TREM2, another microglial gene long-established in AD pathology[78]. Both human and preclinical data supported the association between TREM2 deficiency and risk of AD[78], and scRNA sequencing data also revealed more pronounced expression of *TSPAN14* and *ADAM10* in microglia than neurons[79]. Enhanced shedding of TREM2 has been discovered for a Han Chinese late onset AD-associated *TREM2* coding variant, while reduced TREM2 shedding has been

found when ADAM10 was inhibited[80,81]. Though the mechanism of TSPAN14 regulated TREM2-ADAM10 shedding remains unclear, it has been shown that overexpression of *TSPAN14* increases the surface expression of ADAM10[76]. Further experiments are needed to better understand how the effect of higher expression of *TSPAN14* affects the cleavage of TREM2 by ADAM10, which could potentially alter TREM2-dependent phagocytosis and microglial function.

ACE encodes for the angiotensin-converting enzyme that can convert angiotensin I to angiotensin II, where the latter can constrict blood vessels and increase blood pressure[82]. ACE-inhibitors are widely used as hypertension medication, but ACE also converts Aβ42 to Aβ 40 and has been implicated in AD[83]. Some evidence showed inhibiting ACE increases amyloid deposition in AD mice models and some hypertensive patients taking ACE inhibitors exhibited worsen decline in cognitive function[84]. However, other studies found lower AD risk for those who took ACE inhibitors[85,86] and null effects of ACE inhibition on Aβ levels *in vivo*[87,88]. A recent report found rare *ACE* coding variants and developed a knock-in mouse model for one missense mutation (p.R1279Q), and observed increased ACE protein and activity, together with EEG disruption, memory impairment, neuroinflammation and hippocampal neurodegeneration, but no effect on Aβ[89]. In addition, the adverse outcomes of *ACE* p.R1279Q can be rescued by brain-penetrant medications inhibiting ACE which seems conflicting with our MR findings suggesting lower expression of *ACE* leads to increased AD risk (WR=-0.1, $p=1.4x10^{-7}$).

APH1B contributes to γ-secretase step of APP leading to Aβ accumulation. It has been suggested that targeting Aph1B γ-secretase complex can potentially lower Aβ peptide production in human AD[90]. However, a recent study tested the impact of missense variant rs117618017 in *APH1B*, which was also the top *MetaBrain* eQTL for *APH1B*, observed no effect on the γ-secretase processing of established substrates compared with cells expressing wild-type *APH1B*[91].

SLC39A13 encodes for solute carrier family 39 member 13, a member of the LIV-1 subfamily of the ZIP transporter family. The encoded transmembrane protein functions as a zinc transporter. Homozygous mutations in this gene have been associated with the Ehlers-Danlos syndrome spondylodysplastic type 3 (OMIM 612350). The functional relationship between zinc levels and homeostasis in the brain with Alzheimer's disease pathology remains unclear. However, previous reports have found hippocampus and neocortex, especially layers III and IV, as zinc-rich areas where zinc-enriched neurons were identified.[92] In addition, zinc binds to APP and Aβ[93,94] and considerable evidences showed potential link between alterations to zinc and Aβ pathology of AD.[95] However, inconsistent findings were reported for zinc content in AD brains[96,97] and additional studies are needed to further understand the role of zinc and zinc transporters in AD pathology.[98]

INPP5D encodes for inositol polyphosphate-5-phosphatase D, a member of the inositol polyphosphate-5-phosphatase (INPP5) family with an N-terminal SH2 domain, an inositol phosphatase domain, and two C-terminal protein interaction domains. (RefSeq, Jul 2020) The protein functions as a negative regulator of myeloid cell proliferation and survival, and in the

brain it was identified as microglia-specific which plays an important role in microglial function. Specifically, INPP5D inhibits signal transduction initiated by activation of immune cell surface receptors, including Triggering receptor expressed on myeloid cells 2 (TREM2), Fc gamma receptor (FcγR) and Dectin-1.[99] Inhibiting INPP5D promotes microglial proliferation, phagocytosis, and increases lysosomal compartment size.[100] More recently Tsai *et al.*[101] reported that *INPP5D* is upregulated in AD and elevated *INPP5D* expression levels are associated with microglial markers and amyloid plaque density. Furthermore, in the 5xFAD mouse model, a disease-progression-dependent increase in *INPP5D* expression was observed in plaque-associated microglia[101], suggesting increased *INPP5D* expression as AD risk factor which was consistent with what we predicted using cortex-EUR eQTL-MR.

At the 16p11.2 locus harboring *ZNF668* and *PRSS36*, these two genes were both identified with MR and colocalization evidence. However, high LD in this region made it difficult to ascertain the causal gene and the biological involvements of these genes in AD remain unclear.

We also evaluated 61 of the 73 AD associated eQTL instruments for an interaction effect with cell type proportions. In total, 16 eQTL instruments were significant ieQTLs, 1 with endothelial cells (*CHRNE*), 3 with excitatory neurons (*PVR*, *ZNF646* and *SETD1A*), 3 with microglia's (*MS4A4A*, *CD33* and *HSD3B7*), 3 with oligodendrocytes (*CR1*, *STAG3* and *SLC25A48*), 3 with other neurons (*APOC2*, *EIF4E* and *SMIM19*), 2 with microglia's / other neurons (*SIGLEC11* and *HLA-DQA2*) and 1 with endothelial cells / oligodendrocytes (*TP53INP1*). Of these, 1 eQTL instruments passed the significance threshold for both MR and colocalization (*CR1*), 4 eQTL instruments passed the significance threshold only for MR (*PVR*, *APOC2*, *STAG3* and *MS4A4A*) and 8 eQTL instruments passed the significance threshold only for colocalization (*ZNF646*, *CHRNE*, *CD33*, *SIGLEC11*, *TP53INP1*, *SLC25A48*, *EIF4E* and *SMIM19*).

Attention deficit/hyperactivity disorder (ADHD)

There were no significant Wald ratios for attention deficit/hyperactivity disorder (ADHD). There were two suggestive signals ($p<5x10^{-5}$) for ADHD that pass colocalization, suggesting increased expression of *ARID5B* (WR=0.21, $p=3.80 \times 10^{-5}$) and *GIGYF2* (WR=0.17, $p=3.85x10^{-5}$) increased risk for ADHD. None of these loci were genome-wide significant in the outcome GWAS, so the lack of power could be a contributing factor for the sub-significant findings[102].

We also evaluated 17 of the 17 ADHD associated eQTL instruments for an interaction effect with cell type proportions. In total, 3 eQTL instruments were significant ieQTLs, 1 with astrocytes (*GMPPB*), 1 with endothelial cells (*TIE1*) and 1 with microglia's (*SIRPD*). Of these, 2 eQTL instruments passed the significance threshold only for colocalization (*GMPPB* and *SIRPD*).

Amyotrophic lateral sclerosis

There were three significant Wald ratio findings for amyotrophic lateral sclerosis (ALS) that passed colocalization using the latest ALS GWAS summary statistics as the outcome[103], *MOBP* (WR=0.29, $p=1.1x10^{-8}$), *SCFD1* (WR=0.092, $p=5.31x10^{-15}$) and *G2E3* (WR=0.092, p=5.70x10-

[15]). *MOBP*, is a myelin-associated oligodendrocytic basic protein exclusively expressed in the myelin of the central nervous system[104]. This gene may play a role across neurodegenerative disorders, as this locus is shared across other neurodegenerative GWASs including PSP and CBD[103]. *SCFD1* and *G2E3* are adjacent genes and the eQTL instruments are in high LD (EUR r^2=0.99), so it is difficult to discriminate the underlying biology at this locus. The *SCFD1* gene encodes for the Sm protein Sly1, and facilitates the SNARE complex formation[105]. The SNARE proteins facilitate neurotransmitter release and they have a protective role against neurodegeneration[106]. *G2E3* is a nucleo-cytoplasmic shuttling protein with a HECT domain that controls the protein localization. It has been hypothesized to play a role in cell cycle regulation and response to DNA damage[107].

We also evaluated 27 of the 29 ALS associated eQTL instruments for an interaction effect with cell type proportions. In total, 6 eQTL instruments were significant ieQTLs, 1 with astrocytes (*TNFSF13*), 1 with excitatory neurons (*LY6G5C*), 3 with other neurons (*ZNHIT3*, *DHRS11* and *CAMLG*) and 1 with excitatory neurons / other neurons (*RESP18*). Of these, 4 eQTL instruments passed the significance threshold only for colocalization (*RESP18*, *LY6G5C*, *CAMLG* and *TNFSF13*).

Autism spectrum disorder

There were no significant Wald ratio findings for autism spectrum disorder[108] (ASD), though five of the suggestive signals for ASD passed colocalization.

We also evaluated 11 of the 14 ASD associated eQTL instruments for an interaction effect with cell type proportions. In total, 5 eQTL instruments were significant ieQTLs, 1 with astrocytes (*GABBR1*), 3 with excitatory neurons (*ARL17A*, *CRHR1* and *KANSL1*) and 1 with astrocytes / excitatory neurons / microglia's (*ARL17B*). Of these, 1 eQTL instruments passed the significance threshold only for colocalization (*GABBR1*).

Bipolar disorder

There were 6 significant Wald ratios for bipolar disorder, 4 passed colocalization (PP4>0.7). We find that downregulating *DCLK3* (WR=-0.32, $p=4.79\times10^{-14}$) and *LMAN2L* (WR=-0.24, $p=1.78\times10^{-8}$) increase risk for bipolar disorder and upregulating *GNL3* (WR=0.19, $p=1.07\times10^{-8}$) and *HAPLN4* (WR=0.21, $p=4.44\times10^{-9}$) increase risk for bipolar disorder.

DCLK3 is a member of the doublecortin family and encodes serine/threonine kinase-domains that show substantial homology to Ca2+/calmodulin-dependent (Cam) protein kinases that involves in regulate cyclic AMP (cAMP) signaling. Broadly, members of the doublecortin family involve in neuronal migration, neurogenesis and eye receptor development and are associated with subcortical band heterotopia, lissencephaly, epilepsy, developmental dyslexia and retinitis pigmentosa[109].

LMAN2L encodes a transmembrane protein belonging to the L-type lectin group of type 1 membrane proteins. The LMAN2L protein is located at the endoplasmic reticulum and function

in the mammalian early secretory pathway as a cargo receptor in the transport of glycoproteins. Homozygous mutation *LMAN2L* is known to cause autosomal recessive mental retardation[110] and this gene has also been previously implicated with bipolar disorder due to its interaction with *ANK3*[111].

GNL3, G protein nucleolar 3, is important for stem cell proliferation and involved in maintaining stem cell self-renewal. It is also suggested that *GNL3* encodes for a protein that interacts with p53 and involves in cell death caused by overexpression, as well as tumorigenesis[112]. *GNL3* is associated with schizophrenia, bipolar disorder[113] and osteoarthritis[114].

There is very little information on the gene function of *HAPLN4*, hyaluronan and proteoglycan link protein 4. There are GWAS associations for bipolar disorder, lipid levels (LDL, triglyceride, total cholesterol;[115]) and metabolites near this gene[116]. Gene Ontology (GO) annotations related to *HAPLN4* include extracellular matrix structural constituent and hyaluronic acid binding.

We also evaluated 68 of the 70 bipolar disorder associated eQTL instruments for an interaction effect with cell type proportions. In total, 11 eQTL instruments were significant ieQTLs, 2 with excitatory neurons (*PACS1* and *LRIT2*), 1 with inhibitory neurons (*PBRM1*), 2 with microglia's (*LIMK2* and *ABHD15*), 5 with other neurons (*RHEBL1*, *MED24*, *UTP18*, *RPRD2* and *CDHR1*) and 1 with endothelial cells / oligodendrocytes (*STIMATE*). Of these, 7 eQTL instruments passed the significance threshold only for colocalization (*RHEBL1*, *PACS1*, *MED24*, *CDHR1*, *LIMK2*, *ABHD15* and *LRIT2*).

Epilepsy

MR analysis was performed for several epilepsy outcomes. Epilepsy (all documented cases) was the all-encompassing disease outcome, and additional epilepsy subtype outcomes were also analyzed, including generalized epilepsy (all documented cases), focal epilepsy (all documented cases), focal epilepsy (documented lesion negative), juvenile absence epilepsy, childhood absence epilepsy, focal epilepsy (documented hippocampal sclerosis), focal epilepsy (documented lesion other than hippocampal sclerosis), generalized epilepsy with tonic-clonic seizures and juvenile myoclonic epilepsy[117]. There was one significant Wald ratio that also passed colocalization. It suggests that upregulating *HSD3B7* increases risk for juvenile myoclonic epilepsy (WR=0.044, $p=3.96 \times 10^{-9}$). *HSD3B7* is thought to be a metabolic enzyme that participated in bile acid synthesis[118].

We also evaluated 27 of the 33 epilepsy associated eQTL instruments for an interaction effect with cell type proportions. In total, 5 eQTL instruments were significant ieQTLs, 1 with astrocytes (*ACSF2*), 1 with excitatory neurons (*ZNF646*) and 3 with microglia's (*SIRPD*, *HSD3B7* and *HSD3B7*). Of these, 1 eQTL instruments passed the significance threshold for both MR and colocalization (*HSD3B7*) and 3 eQTL instruments passed the significance threshold only for colocalization (*SIRPD*, *ACSF2* and *HSD3B7*).

Frontotemporal dementia
There was one significant Wald ratio finding for all subtypes of frontotemporal dementia (FTD) that also passed colocalization[119]. It suggested that upregulating *BTNL2* (WR=0.75, p=$2.20x10^{-10}$) increased risk for FTD. This gene is located near the major histocompatibility complex (MHC) region, and the underlying biology of this locus is thought to be driven by the critical immunological genes in the region[119]. The protein encoded by *BTNL2* is known to inhibit T cell activation[120], so the direction of effect found in the MR analysis is confusing. A protein truncating splicing variant in *BTNL2* that reduces protein function has been associated with an inflammatory disease known as sarcoidosis[121], where small subset of the patients present with rapidly progressing dementia[122]. No signal was found for frontotemporal dementia, TDP-43 subtype GWAS outcome, likely due to the low resolution of the GWAS summary statistics.

We also evaluated 3 of the 3 FTD associated eQTL instruments for an interaction effect with cell type proportions, none of which were significant.

None of the FTD TDP-subtype (FTD-TDP) findings had a significant Wald ratio finding[123].

Major depressive disorder
There were four significant Wald ratio findings for Depression (broad) or major depressive disorder[124], of which two also passed colocalization. Upregulating *NEGR1* (WR=0.0044, p=$4.07x10^{-12}$) and *SLC12A5* (WR=0.024, p=$8.03x10^{-8}$) increase risk for major depressive disorder.

In addition to depression, *NEGR1*, encoding neuronal growth regulator 1, is associated with neurociticism, insomnia[125,126], schizophrenia[127], cognitive function[128–130], late onset Alzheimer's disease[131], BMI[132], and lupus[133] in GWAS. The pleiotropic association between *NEGR1* and psychiatric traits and BMI and obesity-related traits suggests a neuronal component of obesity[134].

SLC12A5, solute carrier family 12 member 5, encodes the neuronal KCC2 channel that is the major extruder of intracellular chloride in mature neurons. SLC12A5 is exclusively expressed in the central nervous system. Homozygous or compound heterozygous mutation in the *SLC12A5* causes early infantile epileptic encephalopathy (OMIM: 616645) and heterozygous mutation in the *SLC12A5* gene increases the susceptibility of generalized epilepsy (OMIM: 616685). In addition to depression, *SLC12A5* is associated with neuroticism[135] and chronotype[136] in GWAS. Of note, *SLC12A5* did not show genome-wide significant association in the depression GWAS analyzed in this study, and *SLC12A5* is also a significant MR finding for multiple sclerosis.

We also evaluated 34 of the 42 major depressive disorder associated eQTL instruments for an interaction effect with cell type proportions. In total, 5 eQTL instruments were significant ieQTLs, 1 with endothelial cells (*HLA-C*), 1 with excitatory neurons (*ZNF445*), 2 with other neurons (*AREL1* and *TCTEX1D1*) and 1 with endothelial cells / microglia's (*BTN3A2*). Of these, 1 eQTL instruments passed the significance threshold only for colocalization (*AREL1*).

Multiple Sclerosis
Through our *MetaBrain* eQTL MR approach followed by colocalization filtering, we found 25 genes that are significant and supported by GWAS-eQTL colocalization for multiple sclerosis (MS). MR analysis suggested higher expression of twelve genes (*SLC12A5*, *CYP24A1*, *TRAF3*, *IFITM1*, *TBX6*, *TSFM*, *AVIL*, *MAST3*, *ABCB9*, *NPEPPS*, *TSPAN31* and *SCO2*) and lower expression of thirteen genes (*MPV17L2*, *CCDC155*, *IFITM3*, *TTC34*, *EEF1AKMT3*, *RNFT1*, *IL7*, *MYNN*, *RGS1*, *ZNF746*, *TYMP*, *PPM1F* and *CLECL1*) in the cortex causally associated with greater risk in MS.

For *SLC12A5* we observed a conflicting directionality where loss of function mutations were found previously to be pathogenic for pediatric epilepsy[137,138], but our MR results showed increased expression associated with MS risk (WR=0.44, p=5.2x10^{-13}). Epileptic genes are generally tightly regulated so it is possible to observe detrimental effects in both directions. Interestingly, this locus also harbors *CD40*, which is another strong candidate gene for MS risk but *CD40* locus did not colocalize with the cortex *cis*-eQTL. A secondary signal for *CD40* likely exists at this locus but was undetected based on current data, due to potential cell-type specific effects where *SLC12A5* is more neuronal but *CD40* acts through microglia, phagocytes and endothelial cells[139].

CYP24A1 is discussed in the main results. The instrument SNP, rs2259735 is in strong LD (r^2>0.9) with the reported MS risk SNP rs2248137[140]. *CYP24A1* encodes for a mitochondrial cytochrome P450 hydroxylase that catalyzes the inactivation of 1,25-dihydroxyvitamin D_3 (calcitriol), the active form of vitamin D[141]. Loss of function mutations in *CYP24A1* increase serum calcitriol and cause hereditary vitamin D-mediated PTH-independent hypercalcemia[142,143]. In the brain, vitamin D plays vital functions in regulating calcium-mediated neuronal excitotoxicity, reducing oxidative stress and regulating synaptic activity[144].

TRAF3 encodes one of the tumor necrosis factor receptor (TNFR) associated factors binding one of the TNFR superfamily member CD40, and TRAF3 plays an inhibitory role in CD40 signaling in B lymphocytes[145]. Although it remains mechanistically unclear how genetically determined higher expression of *TRAF3* contributes to MS risk (WR=0.26, p=8x10^{-9}), TRAF3 deficiency was shown to disrupt the signaling pathway of Epstein Barr virus (EBV)-encoded CD40 mimic, Latent Membrane Protein 1 (*LMP1*), which is necessary for EBV-associated lymphoproliferation and potentially MS etiology[146].

IFITM1 and *IFITM3* are interferon (IFN)-induced transmembrane proteins and are involved in IFN signaling pathways. IFN-β was the first therapy approved that could change the course of MS. However, we observed opposite directionalities between *IFITM1* (WR=0.49, p=1.8x10^{-8}) vs. *IFITM3* (WR=-0.3, p=2.3x10^{-8}) in MS risk. The biological mechanisms for IFITMs in MS remain unclear but a recent report suggested IFITM1 increases the infectivity of EBV[147], further evidence is needed to understand the involvement of IFITMs in MS etiology.

We also found two genes: *TBX6* and *CCDC155*, though the biological understanding of their involvement in MS is lacking, with colocalization evidence for *cis*-methylation QTL (*cis*-mQTL) in CD4+ T cells derived from MS patients[148]. For the *TBX* locus, the MS risk allele (rs3809627-C) is associated with hypomethylation and consistently we observed higher expression of *TBX6* is associated with increased MS risk (WR=0.2, p=3.2x10^{-8}). For the *CCDC155* locus, the MS risk allele (rs1465697-T) is associated with hypermethylation and we also observed lower expression of *CCDC155* associated with MS risk (WR=-0.58, p=9.3x10^{-10}).

MPV17L2 is associated with mitochondria[149]. Although the functional mechanism of how *MPV17L2* contribute to MS remain unclear, mitochondria dysfunction can contribute to neurodegeneration and axonal loss in MS[150,151].

We found lower expression of *TTC34* associated with higher risk in MS (WR=-0.43, p=2.1x10^{-15}), but the biological mechanisms of *TTC34* involvement in MS etiology remain unclear. A few reported relevance of *TTC34* in autoimmune diseases such as systemic lupus erythematosus (SLE)[152,153] and type I diabetes[154].

We found three genes with significant MR associations and positive colocalization signals from the 12q14.1 locus, *EEF1AKMT3*, *TSFM* and *TSPAN31*. Lower expression of *EEF1AKMT3* (WR=-0.13, p=9x10^{-11}) and higher expression of *TSFM* (WR=0.16, p=1.6x10^{-10}) and TSPAN31 (WR=0.48, p=5.1x10^{-11}) were found putatively causal for increasing MS risk. *EEF1AKMT3* encodes a lysine methyltransferase targeting methylation of Lys-165 in EEF1A in an aminoacyl-tRNA- and GTP-dependent manner[155,156]. *EEF1A* encodes eukaryotic elongation factor 1A (with two isoforms, EEF1A1 and EEF1A2) and plays an important role in regulation of protein elongation and synthesis[157]. Lys-165 methylation of EEF1A-coupled with upregulation of *EEF1AKMT3* can be increased by various types of stress including ER-stress[155] and *EEF1A* overexpression under ER-stress has been found to prevent apoptosis[158]. Previous reports showed evidence on the potential involvement of EEF1A and apoptosis pathway in neurodegeneration and PD[159–161]. In humans, mutations in *EEF1A2* have been linked with multiple neurological deficits including developmental delay, autistic behaviors and epilepsy[162–164], and dysregulation of EEF1A is also observed in AD[165,166]. *TSFM* encodes a mitochondrial translation elongation factor and plays an important role in mitochondrial protein translation. Mutations in *TSFM* have been associated with rare infantile mitochondrial disorders with various clinical manifestations including fatal encephalomyopathy, cardiomyopathy, neuropathy and childhood onset ataxia[167–169]. Although the biological mechanisms of *TSFM* in MS etiology are not well-understood, the involvement of mitochondrial genes and pathways have been described in MS[151], including two (*TSFM* and *MPV17L2*) that were found by our MR analysis. *TSPAN31* is the natural antisense transcript of cyclin dependent kinase 4 (CDK4) and regulates the expression of *CDK4* mRNA and protein[170]. Higher expression of *TSPAN31* can potentially down-regulate *CDK4* and prevent cell proliferation, and CDK4/6-inihibitors have been developed as a therapeutic strategy to treat multiple cancers[171]. Some evidence has been shown for the potential involvement of CDK4 in regulating immune cells where CDK4/6-inhibition promotes T-cell activity[172,173]. However,

others have also reported potential treatment effect of CDK4/6-inhibitors for autoimmune diseases such as RA without obvious effect on immune response[174].

NPEPPS, encodes for a protein that is also known as puromycin-sensitive aminopeptidase and hydrolyzes physiological endogenous peptides such as dynorphins and enkephalins, and contributes to the degradation of these peptides in the brain[175]. Interestingly, NPEPPS has been reported to degrade tau protein and inhibit tau-induced neurodegeneration[176,177], as well as regulate SOD1 via proteolysis in ALS[178]. However, we found higher gene expression of *NPEPPS* leading to higher risk in MS (WR=0.43, $p=1.1\times10^{-10}$).

RNFT1, encoding Ring finger protein transmembrane 1, is an E3 ubiquitin-protein ligase participating in the ubiquitin proteasome system (UPS) that induces protein degradation. This protein also has zinc-binding activities[179]. Giordana *et al.* observed abnormally strong colocalization of ubiquitin in the myelinated white matter of all six MS patients analyzed, suggesting that MS patients may suffer from UPS deficits[180].

Interleukin 7 (encoded by *IL7*) regulates naïve and memory CD8+ T cells and has been known to be a critical gene for early T-cell development[181]. This gene has been suggested to play a role in autoimmunity. A decrease systemic IL-7 and soluble IL-7Rα was observed in MS patients[182], and our MR findings also suggest that abnormalities in the IL-7 pathway could increase risk for MS.

SCO2, which encodes synthesis of cytochrome c oxidase (COX) 2, is a COX assembly gene that modulates proton transfer across the inner mitochondrial membrane. Loss of function mutations of *SCO2* also cause an autosomal recessive form of fatal cardioencephalomyopathy and COX deficiency[183], Loss of function mutations in the functional catalytic domain of SCO2 causes an autosomal dominant form of myopia[184]. Several reports have suggested a link between mitochondrial dysfunction and multiple sclerosis[150].

Myoneurin (*MYNN*) encodes for a protein with a classic C2H2 zinc finger motif and a BTB/POZ protein-protein interaction domain. There is limited information published about the function of this protein, though based on its protein domains, it is hypothesized to play a regulatory function in gene expression and transcriptional activation and repression[185].

TYMP encodes for thymidine phosphorylase, an angiogenic factor which promotes angiogenesis in vivo and stimulates the in vitro growth of a variety of endothelial cells.[186] Mutations leading to TYMP depletion cause mitochondrial DNA depletion syndrome I. (OMIM 603041) In inflammatory CNS diseases such as MS, TYMP was suggested as an important astrocyte-derived permeability factor responsible for reactive astrocytes-driven blood–brain barrier breakdown.[187] In the multiple sclerosis model experimental autoimmune encephalitis, TYMP and VEGFA co-localize to reactive astrocytes, and correlate with blood–brain barrier permeability. In addition, TYMP and VEGFA both localize to reactive astrocytes in multiple sclerosis lesion samples.

RGS1 encodes for regulator of G protein signaling 1, a member of the regulator of G-protein signaling family. RGS1 attenuates the signaling activity of G-proteins by binding to activated, GTP-bound G alpha subunits and acting as a GTPase activating protein (GAP), increasing the rate of conversion of the GTP to GDP. (RefSeq, Jul 2008) Previous reports have shown higher levels of RGS1 expression in MS patients as well as in response to interferon (IFN)-γ therapy.[188] In B lymphocytes, RGS1 impairs Gαi signaling responses[189] and its silencing enhances responsiveness to chemokines such as chemokine (C-X-C motif) ligand (CXCL)12 and CXCL13 and impairs desensitization[190]. *RGS1* overexpression inhibits T cell migration in response to chemokines that control lymphoid homing, while depletion of RGS1 selectively enhances such chemotaxis. Given that RGS1 is the most abundant RGS protein in microglia, it's involvement in the regulation of microglia chemotaxis could be important for MS etiology.

MAST3 encodes for microtubule associated serine/threonine kinase 3, it has been previously reported as relevant for multiple inflammatory disease including rheumatoid arthritis[191,192] and inflammatory bowel disease[193,194]. *MAST3* has also been found associated with neurodevelopmental diseases and epilepsy[195], but it's potential involvement in MS remains unclear.

PPM1F encodes for protein phosphatase, Mg2+/Mn2+ dependent 1F, a member of the PP2C family of Ser/Thr protein phosphatases. PP2C family members are known to be negative regulators of cell stress response pathways. (RefSeq, Jul 2008) PPM1F has been reported as potential regulator of pathogenic genes for anxiety behaviors as well as mental illnesses[196–199], but it's role in CNS inflammation or MS remains unknown.

AVIL encodes for advillin, a member of the gelsolin/villin family of actin regulatory proteins. This protein has structural similarity to villin. It binds actin and may play a role in the development of neuronal cells that form ganglia. (RefSeq, Jul 2008) Mutations in *AVIL* have been reported as pathogenic for nephrotic syndrome type 21, an autosomal recessive renal disorder characterized by onset of kidney dysfunction in the first year of life. (OMIM 618594) In the CNS, advillin has been reported to be relevant for neuropathic pain[200] and glioblastoma.[201,202]

ABCB9 encodes for ATP binding cassette subfamily B member 9, a membrane-associated protein belonging to the superfamily of ATP-binding cassette (ABC) transporters. ABC proteins transport various molecules across extra- and intra-cellular membranes. This protein is a member of the MDR/TAP subfamily. Members of the MDR/TAP subfamily are involved in multidrug resistance as well as antigen presentation. This family member functions in the translocation of peptides from the cytosol into the lysosomal lumen. (RefSeq, Jul 2008) Pleiotropic genetic associations of *ABCB9* were reported for schizophrenia and MS[203] but a clear understanding of its role in CNS illnesses remains lacking.

ZNF746 encodes for zinc finger protein 746, which belongs to a subset of Kruppel-associated box (KRAB) zinc finger (ZNF) transcription factors. ZNF746 was found coimmunoprecipitated with parkin from whole human striatum or mouse brain[204] ZNF746 levels were elevated in the

cingulate cortex of patients with autosomal recessive Parkinson's disease (PD), in the striatum and substantia nigra of patients with sporadic PD, and in an adult mouse PD model following conditional parkin knockout[204] ZNF746 also binds to transcriptional coactivator PGC1-alpha in regulating mitochondrial biogenesis and dopamine neuron degeneration[204,205] However, its involvement in neuroinflammation or MS remains unclear.

CLECL1 is discussed in the main results.

We also evaluated 151 of the 199 MS associated eQTL instruments for an interaction effect with cell type proportions. In total, 37 eQTL instruments were significant ieQTLs, 3 with astrocytes (*HLA-DOB*, *MICA* and *LRRC34*), 2 with endothelial cells (*HLA-C* and *EFCAB13*), 7 with excitatory neurons (*LY6G5C*, *IQCB1*, *IL7*, *ZNF774*, *SKIV2L*, *PVR* and *RRP12*), 6 with microglia's (*EEF1AKMT3*, *CLECL1*, *IFITM1*, *TEAD2*, *DEXI* and *MALT1*), 6 with oligodendrocytes (*SFTA2*, *MUCL3*, *ASPHD1*, *C4orf36*, *AC099489.1* and *HSD17B8*), 6 with other neurons (*TSFM*, *HLA-DPB1*, *CASQ1*, *ZNHIT3*, *DHRS11* and *UBASH3B*), 2 with microglia's / other neurons (*HLA-DRB1* and *HLA-DQA2*), 2 with excitatory neurons / other neurons (*CYP24A1* and *RMI2*), 1 with microglia's / oligodendrocytes (*PSORS1C1*), 1 with astrocytes / excitatory neurons (*RGS14*) and 1 with endothelial cells / microglia's / oligodendrocytes (*HIST1H3E*). Of these, 6 eQTL instruments passed the significance threshold for both MR and colocalization (*CYP24A1*, *EEF1AKMT3*, *TSFM*, *CLECL1*, *IL7* and *IFITM1*), 11 eQTL instruments passed the significance threshold only for MR (*HLA-DRB1*, *HLA-DQA2*, *HLA-C*, *HLA-DOB*, *SFTA2*, *MUCL3*, *MICA*, *LY6G5C*, *RMI2*, *IQCB1* and *HLA-DPB1*) and 7 eQTL instruments passed the significance threshold only for colocalization (*ASPHD1*, *C4orf36*, *CASQ1*, *ZNHIT3*, *PVR*, *RRP12* and *UBASH3B*).

Parkinson's disease

There were 6 significant WR findings for Parkinson's disease (PD) that passed colocalization (PP4>0.7) that suggest increasing expression of *KANSL1* (WR=-0.25, p=3.35x10^{-19}), *RAB29* (WR=0.21, p=1.03x10^{-8}) and *SCARB2* (WR=0.29, p=6.54x10^{-8}) and decreased expression of *CD38* (WR=-0.24, p=6.99x10^{-14}), *HSD3B7* (WR=-0.46, p=1.90x10^{-10}) and *SETD1A* (WR=-0.67, p=2.43x10^{-9}) increased risk for disease.

KANSL1 encodes for a nuclear protein involved in chromatin modifications a member of a histone acetyltransferase (HAT) complex. We observe that decreased *KANSL1* expression increases risk for PD. Interestingly, loss of function mutations in *KANSL1* causes Koolen-De Vries syndrome, a neurodevelopmental disorder with associated hypotonia[206]. *KANSL1* deficiency is known to cause autophagosome accumulation leading to lysosomal impairment[207].

The finding that increased *RAB29* expression increases risk for PD risk is interesting, because Rab29 controls LRRK2 activation, localization and possibly phosphorylation as well. *LRRK2* mutations cause the most common autosomal dominant form of Parkinson's disease and this gene plays a role in the endolysosomal regulation. Pathogenic *LRRK2* mutations are more readily recruit to the Golgi and activated by Rab29[208,209].

Scavenger Receptor Class B Member 2 (encoded by *SCARB2*) is a type III glycoprotein located in the endosomal/lysosomal cell compartment and regulates endolysosomal transport. Autophagy dysregulation has been repeatedly reported as a driving factor for Parkinson's disease[210]. This gene also causes an autosomal recessive form of progressive myoclonic epilepsy-4 (EPM4), whereby the mutations found in three unrelated probands resulted in the lack of *SCARB2* protein[211].

CD38, encoding a protein also known as cyclic ADP-ribose hydrolase, plays a role in regulating microglia through microglial activation and activation-induced cell death[212]. This enzyme is also responsible for nicotinamide adenine dinucleotide (NAD) degradation, and NAD levels are important in age-related metabolic decline and have been observed to decrease with age[213]. CD38 forms a complex with LRRK2, a protein that is overactivated in a Mendelian form of PD. The CD38-LRRK2 complex is activated by TFEB after internalization from the plasma membrane[214]. There is conflicting evidence whether increasing or attenuating *CD38* is beneficial for neurodegeneration[215], although the eQTL effect direction is consistent in our study across included datasets. Follow up studies are required to fully disentangle the mechanisms of this disease variant.

Interestingly, while decreased *HSD3B7* levels could increase risk for PD, we had noted above that upregulating this gene increases risk for juvenile myoclonic epilepsy. The latest PD GWAS publication in eQTLGen also reported significant SMR findings for this gene was also reported in blood (eQTLGen[17]) and in brain[43] eQTL dataset in opposite directions of effect. The results did not pass the HEIDI p-value threshold, suggesting a potential pleiotropic association invalidating the MR finding. Thus, this finding should be interpreted with caution. We were underpowered to detect associations with Parkinson's disease due to the lack of power with the publicly available summary statistics[216].

SETD1A encodes for a component a histone methyltransferase complex. Missense mutations have been observed to cause an autosomal dominant form of early-onset epilepsy with or without developmental delay[217]. Haploinsufficiency of *SETD1A* causes neurodevelopmental disorder with speech impairment and dysmorphic facies (NEDSID)[218].

We also evaluated 42 of the 50 PD associated eQTL instruments for an interaction effect with cell type proportions. In total, 15 eQTL instruments were significant ieQTLs, 2 with astrocytes (*CD38* and *ADORA2B*), 8 with excitatory neurons (*ARL17A*, *CRHR1*, *KANSL1*, *SETD1A*, *LRRC37A*, *LRRC37A2*, *ZNF646* and *CCDC158*), 1 with microglia's (*HSD3B7*), 1 with oligodendrocytes (*FAM200B*), 1 with other neurons (*NUP42*), 1 with astrocytes / excitatory neurons / microglia's (*ARL17B*) and 1 with oligodendrocytes / other neurons (*ZSWIM7*). Of these, 4 eQTL instruments passed the significance threshold for both MR and colocalization (*KANSL1*, *CD38*, *HSD3B7* and *SETD1A*), 3 eQTL instruments passed the significance threshold only for MR (*ARL17A*, *CRHR1* and *ARL17B*) and 2 eQTL instruments passed the significance threshold only for colocalization (*ZSWIM7* and *ADORA2B*).

Schizophrenia

There are 32 MR findings that colocalize (PP4>0.7) for schizophrenia (SCZ) risk[219], suggesting that increasing expression of *ATP13A1* (WR=0.30, p=1.08x10^{-7}), *CACNA1I* (WR=0.32, p=1.22x10^{-8}), *CLCN3* (WR=0.24, p=1.14x10^{-7}), *CNTN4* (WR=0.26, p=9.19x10^{-10}), *DOC2A* (WR=0.27, p=1.97x10^{-9}), *GLYCTK* (WR=0.19, p=7.22x10^{-8}), *GNL3* (p=0.15, p=3.46x10^{-9}), *GPANK1* (WR=0.24, p=2.56x10^{-13}), *HIST1H4K* (WR=0.63, p=1.40x10^{-18}), *INO80E* (WR=0.11, p=2.47x10^{-9}), *KCTD13* (WR=0.39, p=9.30x10^{-9}), *KMT2E* (WR=0.29, p=1.64x10^{-8}), *PPP1R18* (WR=0.48, p=1.11x10^{-19}), *RERE* (WR=0.16, p=4.99x10^{-8}), *SF3B1* (p=0.21, p=6.97x10^{-11}), *SNAP91* (WR=0.23, p=6.34x10^{-9}), *TAOK2* (WR=0.44, p=1.05x10^{-9}), *TM6SF2* (WR=0.26, p=7.06x10^{-9}), *TMEM219* (WR=0.30, p=1.87x10^{-9}) and *ZNF823* (WR=0.19, 4.14x10^{-8}) and decreasing expression of *ASPHD1* (WR=-0.23, p=1.33x10^{-9}), *ATG13* (WR=-0.29, p-1.23x10^{-9}), *FTCDNL1* (WR=-0.16, p=7.97x10^{-14}), *FURIN* (WR=-0.23, p=2.55x10^{-12}), *GATAD2A* (WR=-0.12, p=1.34x10^{-8}), *MDK* (WR=-0.29, p=2.23x10^{-10}), *MICB* (WR=-0.37, p=1.82x10^{-15}), *PCCB* (WR=-0.12, 1.12x10^{-9}), *PLEKHO1* (WR=-0.35, p=1.97x10^{-9}), *PTPRU* (WR=-0.19, p=4.28x10^{-8}), *THOC7* (WR=-0.14, p=6.18x10^{-10}) and *VPS45* (WR=-0.27, p=1.14x10^{-8}) increases risk for schizophrenia.

ASPHD1, Aspartate Beta-Hydroxylase Domain Containing 1, is a protein coding gene associated with 16p11.2 deletion syndrome[220]. In this region, 16p11.2 microduplications have been associated with a 14.5-fold increased risk of schizophrenia[221].

ATG13, autophagy-related 13, encodes for a protein critical in the formation of autophagosomes[222]. There is growing evidence that autophagy plays a key role in the pathophysiology of schizophrenia, where reduced levels of autophagy genes were round in the post-mortuem hippocampus samples of schizophrenia patients compared to control subjects[223].

ATP13A1, encoding ATPase 13A1, is part of the P5-type ATPase that localizes in the endoplasmic reticulum. It modulates MR1, monomorphic antigen-presenting molecule major histocompatibility complex-I-related protein 1[224], though its role in schizophrenia is not clear.

CACNA1I, Calcium Voltage-Gated Channel Subunit Alpha1 I, encodes the pore-forming alpha subunit of a voltage gated calcium channel. Gain-of-function mutations that affect channel gating properties have been reported to cause neurodevelopmental disorders and epilepsy. Rare missense mutations have been suggested follow an allelic series for schizophrenia[225].

CLCN3, encoding chloride voltage-gated channel 3, did not show genome-wide significant association in schizophrenia GWAS analyzed in this study; however, it was associated in another published schizophrenia GWAS[226]. *CLCN3* encodes a member of the voltage-gated chloride channel (ClC) family, which is present in all cell types and localized in plasma membranes and in intracellular vesicles. Besides schizophrenia, *CLCN3* is also associated with Parkinson's disease[216] described above.

CNTN4, contactin 4, encodes a member of the contactin family of immunoglobulins, which are axon-associated cell adhesion molecules that function in neuronal network formation and plasticity. *CNTN4* is known to be associated with chromosome 3p deletion syndrome. In GWAS, *CNTN4* variants are associated schizophrenia, autism, intelligence, amyotrophic lateral sclerosis, as well as gallbladder cancer[227] and basophil count[228].

DOC2A, encoding Double C2 Domain Alpha, is suggested to play a role in neurotransmitter release. Like *ASPHD1* described above, this gene resides in the 16p11.2 deletion/duplication region.

FTCDNL1 encodes for formiminotransferase cyclodeaminase N-terminal like, and this protein has been related to transferase activity and folic acid binding in Gene ontology and is associated with schizophrenia, osteoporosis[229], fracture and type 2 diabetes[230] in GWAS.

FURIN, also known as furin paired basic amino acid cleaving enzyme, encodes a type 1 membrane bound protease that belongs to the subtilisin-like proprotein convertase family. The protease processes protein and peptide precursors trafficking through regulated or constitutive branches of the secretory pathway and is expressed in many tissues, including neuroendocrine and brain. *FURIN* is associated with epilepsy through regulating GABA-A receptors-mediated inhibitory synaptic transmission[231]. More recently, it is also found that *FURIN* plays a crucial role in SARS-CoV-2 spike protein cleavage, which mediates the virus entry into cells[232]. It is also associated with schizophrenia, autism[233], insomnia[125], risk-taking behavior[234], as well as hypertension/blood pressure[125], coronary artery disease[235] and parental longevity[236].

GATAD2A, GATA zinc finger domain containing 2A, encodes a protein as a subunit of the methyl-CpG-binding protein-1 complex (MeCP1). MeCP1 deacetylates methylated nucleosomes to repress gene expression[237]. *GATAD2A* is associated with schizophrenia and thought to have pleiotropic effects on breast cancer risk[238]. It's also associated with type 2 diabetes[239].

GLYCTK, glycerate kinase, encodes a member of the glycerate kinase type-2 family and encoded enzyme catalyzes the phosphorylation of (R)-glycerate. GLYCTK did not show genome-wide significant association in schizophrenia GWAS analyzed in this study. It is known to be associated with D-glyceric aciduria (glycerate kinase deficiency; OMIM: 220120). The rare autosomal recessive metabolic disorder has highly variable phenotypes from severe phenotypes like encephalopathy, severe mental retardation, seizures, microcephaly, to mild phenotypes such as mild speech delay or even normal development[240].

GNL3 is described in the bipolar section.

GPANK1, G-Patch Domain and Ankyrin Repeats 1, is a gene within the human major histocompatibility complex with unknown function[241].

HIST1H4K is the gene that encodes for Histone H4, and HDAC inhibitors have been hypothesized to act synergistically with antipsychotic drugs[242].

INO80E, INO80 complex subunit E, is associated with schizophrenia, body mass index[243] and depression-related symptoms[244].

KCTD13, Potassium Channel Tetramerization Domain Containing 13, is involved in synaptic transmission and this gene resides in the 16p11.2 locus with copy number findings[245].

KMT2E, Lysine Methyltransferase 2E, acts as a transcriptional regulator via its role in chromatin remodeling. Heterozygous mutations in *KMT2E* cause a spectrum of neurodevelopmental disorders and epilepsy[246].

MDK, or midkine, encodes a retinoic acid-responsive, heparin-binding growth factor expressed in various cell types during embryogenesis. MDK protein promotes angiogenesis, cell growth, and cell migration in particular during tumorigenesis. It is associated with schizophrenia, autism[233] and smoking initiation[247] in GWAS and has been targeted as a therapeutic for a variety of different diseases, such as non-small cell lung cancer[248] and hepatocellular carcinoma[249].

MICB, MHC Class I Polypeptide-Related Sequence B, is a stress-induced ligand for the NKG2D receptor[250]. In addition to contributing to schizophrenia risk, polymorphisms in this gene also appear to be associated with human herpes virus seropositivity, suggesting possible gene-environment interactions contributing to schizophrenia[251,252].

PCCB, propionyl-CoA carboxylase subunit beta, encodes the beta subunit of the propionyl-CoA carboxylase (PCC) enzyme, which is involved in the catabolism of propionyl-CoA. *PCCB* defects are known to be a cause of propionic acidemia type II (PA-2) (OMIM: 606054), intellectual disability (OMIM: 617635) and are associated with autism in propionic acidemia patients[253]. In GWAS, *PCCB* is associated psychiatric traits including schizophrenia, cognitive performance[254], neuroticism[255]; anthropometric traits including height[256], body mass index[257], and waist-hip ratio[257] and metabolic traits including circulating fibrinogen levels[258], C-reactive protein levels[259], HDL cholesterol[260] and triglycerides[261].

PLEKHO1, Pleckstrin Homology Domain Containing O1, plays a role in the regulation of actin cytoskeleton[262], and its role in schizophrenia is not clear.

PPP1R18, Protein Phosphatase 1 Regulatory Subunit 18, encodes a phostensin near the HLA locus. This gene is implicated in neurodevelopment and suggested to play a role in synaptic plasticity via its interaction with phosphatase PP1[263].

PTPRU, Protein Tyrosine Phosphatase Receptor Type U, is a member of the protein tyrosine phosphatase family that participates in many cellular processes including. cell growth,

differentiation, mitotic cycle and oncogenic transformation. It is expressed in the cortex and involved in neural development[264].

RERE, also known as arginine-glutamic acid dipeptide repeats, encodes a member of the atrophin family of arginine-glutamic acid (RE) dipeptide repeat-containing proteins. The *RERE* gene is a nuclear receptor coregulator that positively regulates retinoic acid signaling. *RERE* is known to be associated with neurodevelopmental disorder (OMIM: 616975), which is an autosomal dominant syndrome characterized by developmental delay, intellectual disability, and behavioral disorders, such as autism spectrum disorders[265]. *RERE* is also associated with a wide range of phenotypes including cognitive function[254,266], neuroticism[126,267], depression[268], smoking[247], well-being[135], white blood cell count[228,269], ophthalmology measure[270,271] and myopia[272], blood pressure[273], heel bone mineral density[274] and asthma[275,276].

SF3B1, splicing factor 3b subunit 1, encodes subunit 1 of the splicing factor 3b protein complex, which is part of the U2 small nuclear ribonucleoproteins complex (U2 snRNP) that has an essential role in the selection of the precursor mRNA branch-site adenosine (the nucleophile for the first step of splicing)[277]. It has been shown that inhibition of *SF3B1* reduced cell proliferation, induced apoptosis, and resulted in cell cycle arrest human gastric cancer cells in vitro[278]. The somatic mutations in *SF3B1* have been associated with prolactinomas[279] and chronic lymphocytic leukemia[280]. *SF3B1* is also associated with schizophrenia and depression[281] GWASs.

SNAP91, Synaptosome Associated Protein 91, colocalizes with active synaptogenesis and synaptic maturation[282]. It is expressed exclusively in neurons and enriched in the presynaptic terminal, where it's protein product helps recycle clathrin coated vesicles at the presynaptic membrane[283].

TAOK2, TAO Kinase 2, is a gene located at the 16p11.2 locus, and it's protein product has multiple roles in the cell including cell signaling, microtubule organization and stability as well as apoptosis[284]. This gene is a direct target FMRP, the protein that causes Fragile X syndrome, and it is also implicated in Autism spectrum disorder susceptibility[285].

THOC7, THO complex 7, encodes a protein that is part of the THO complex, which together with ALY and UAP56 forms the human TREX complex. The TREX complex is recruited to spliced mRNAs by a transcription-independent mechanism and is recruited in a splicing- and cap-dependent manner to a region near the 5' end of the mRNA where it functions in mRNA export to the cytoplasm via the TAP/NFX1 pathway. In addition to schizophrenia, *THOC7* is also associated with neutrophil count[269].

TM6SF2, Transmembrane 6 Superfamily Member 2, is a gene primarily expressed in the liver and small intestine and this gene is known to play a large role in liver metabolism[286]. It has been implicated in susceptibility to non-alcoholic fatty liver disease and hepatic fibrosis progression in

patients with non-alcoholic fatty liver disease[287]. However, there has not been an established functional connection with schizophrenia.

TMEM219, encoding Transmembrane Protein 219, has been shown to be a direct binding partner of IL13RA2[288], although its function still remains unknown.

VPS45, encoding Vacuolar Protein Sorting 45 Homolog, is observed to be highly expressed in peripheral blood mononuclear cells. This gene causes an autosomal recessive form of neutropenia[289], and suggests a role as an inflammatory mediator.

Little is known about *ZNF823*, zinc finger protein 823 and its gene function. It has been associated with metabolite levels[290].

We also evaluated 194 of the 231 SCZ associated eQTL instruments for an interaction effect with cell type proportions. In total, 36 eQTL instruments were significant ieQTLs, 3 with astrocytes (*NAGA*, *AS3MT* and *KCNN3*), 2 with endothelial cells (*ZKSCAN3* and *HLA-C*), 5 with excitatory neurons (*PTPRU*, *JRK*, *MICB*, *SLCO4C1* and *TMEM81*), 1 with inhibitory neurons (*PBRM1*), 10 with oligodendrocytes (*FTCDNL1*, *PCCB*, *ASPHD1*, *ZCCHC17*, *NMRAL1*, *ZSCAN29*, *CORO7*, *CNPPD1*, *SFTA2* and *MUCL3*), 7 with other neurons (*THOC7*, *CNOT7*, *RPRD2*, *ZMAT2*, *NDUFAF7*, *METTL21A* and *MSR1*), 1 with endothelial cells / microglia's (*BTN3A2*), 2 with microglia's / oligodendrocytes (*HLA-DMA* and *PSORS1C1*), 1 with astrocytes / excitatory neurons (*ZSCAN31*), 1 with endothelial cells / oligodendrocytes (*STIMATE*), 1 with astrocytes / oligodendrocytes / other neurons (*BORCS7*), 1 with astrocytes / endothelial cells (*SLC9C2*) and 1 with astrocytes / inhibitory neurons (*TNFRSF13C*). Of these, 5 eQTL instruments passed the significance threshold for both MR and colocalization (*FTCDNL1*, *THOC7*, *PCCB*, *ASPHD1* and *PTPRU*), 5 eQTL instruments passed the significance threshold only for MR (*BTN3A2*, *HLA-DMA*, *ZKSCAN3*, *ZSCAN31* and *STIMATE*) and 12 eQTL instruments passed the significance threshold only for colocalization (*CNOT7*, *ZCCHC17*, *NMRAL1*, *ZSCAN29*, *CORO7*, *CNPPD1*, *TMEM81*, *NDUFAF7*, *METTL21A*, *MSR1*, *SLC9C2* and *KCNN3*).

Years of schooling and cognitive function

There are 32 findings for cognitive function[124] and 70 MR findings for years of schooling[17,80,268] that passed colocalization (PP4>0.7, **Supplementary Table 12**). This is the first report of MR with brain *cis*-eQTL instruments for the cognitive function and years of schooling outcomes. Lee *et al.* only reported brain eQTLs from GTEx, and there were only 2 loci that had eQTLs for *PITPNM2* at 12q24.31 and two genes at 22q13.1, *TAB1* and *MGAT3* (Table S16 in Lee *et al*[254]).

We investigated pathways enriched for highly co-regulated genes of the findings that passed MR and colocalization using the *MetaBrain* GeneNetwork browser. The top HPO pathway enrichments for cognitive function include Cerebellar hypoplasia ($p=1.1x10^{-6}$) and Aplasia/Hypoplasia of the cerebellum ($p=2.6x10^{-6}$), suggesting an important role of the cerebellum in cognitive function. Cerebellum volume and function has long been linked to

intelligence[291–293]. The top three HPO enrichment terms for years of schooling included Cerebellar hypoplasia (p=4.9x10^{-9}), Abnormal nervous system physiology (p=3.2x10^{-8}) and Abnormal skeletal muscle morphology (p=1.3x10^{-7}). The findings suggest that contributions from important neurodevelopmental genes could factor into the years of schooling trait. Interestingly, there is also pathway enrichment of a muscle phenotype, and motor delay is a common co-morbidity with intellectual disability/developmental delay[294].

There are 11 genes with significant findings for both traits, *CALN1*, *CYB561D1*, *CYHR1*, *DBN1*, *KIFC2*, *NPIPB9*, *RHEBL1*, *SLC22A23*, *SORT1*, *SYPL2* and *TUFM*. There is shared genetic basis between years of schooling and cognitive function, and they are genetically correlated with the cerebral cortical morphology[295]. Moreover, the genes common to these two traits may influence cognitive ability.

We also evaluated 674 of the 752 Years of schooling and cognitive function associated eQTL instruments for an interaction effect with cell type proportions. In total, 149 eQTL instruments were significant ieQTLs, 26 with astrocytes (*NPIPB7*, *SCG3*, *GMPPB*, *NAGA*, *ZNF502*, *GSTM4*, *RNF182*, *ZNF501*, *NPIPB7*, *GMPPB*, *ID4*, *CHRNB1*, *HIST1H1C*, *ELOA3B*, *ELOA3D*, *NT5C2*, *ZNF501*, *TRPM7*, *ZNF502*, *COX6B1*, *FAT3*, *STMP1*, *CZIB*, *PPP1CB*, *NAGA* and *SCG3*), 4 with endothelial cells (*THSD7B*, *DNAH11*, *TIE1* and *LAMC3*), 25 with excitatory neurons (*AFF3*, *GOLGA6L10*, *ELP1*, *ARL17A*, *CRHR1*, *CCDC32*, *FKBP11*, *EFCAB5*, *KANSL1*, *SIRPB1*, *CRHR1*, *ARL17A*, *KANSL1*, *AFF3*, *SLC35G6*, *AP2B1*, *PILRA*, *ARTN*, *WDR27*, *RRP12*, *LY6D*, *MYO15A*, *ELP1*, *WFIKKN1* and *PACS1*), 1 with inhibitory neurons (*PBRM1*), 19 with microglia's (*SEMA3F*, *SLC22A23*, *MYBPHL*, *CHADL*, *PSRC1*, *ELOVL7*, *FAM107B*, *ELOVL7*, *ABITRAM*, *ESR2*, *FCER1A*, *SLC22A23*, *HIST1H3C*, *MYBPHL*, *EEF1AKMT3*, *OLR1*, *PSRC1*, *C2orf92* and *WDR5B*), 12 with oligodendrocytes (*CUEDC2*, *ARMC1*, *PCCB*, *PAIP2B*, *SH3BGR*, *FPGT*, *CUEDC2*, *SNF8*, *PDE1A*, *CNOT9*, *ARMC1* and *AIF1L*), 35 with other neurons (*RHEBL1*, *AKTIP*, *ZMAT2*, *GATB*, *FAM180B*, *DHRS11*, *ZNHIT3*, *C1QTNF4*, *RABGAP1L*, *NKIRAS1*, *TFB1M*, *CYP2D6*, *SYNDIG1L*, *C20orf27*, *RHEBL1*, *SMIM19*, *SYNDIG1L*, *GALNT18*, *FIBP*, *PIAS2*, *CALU*, *GSDME*, *WDR92*, *CRYZ*, *AKTIP*, *EEF1AKMT2*, *TSFM*, *FAM184A*, *CYB5D2*, *RABGAP1L*, *NIPSNAP1*, *TGM5*, *SNX4*, *DHRS11* and *GATB*), 4 with microglia's / oligodendrocytes (*TEX14*, *PHACTR4*, *BCHE* and *DHFR*), 8 with astrocytes / oligodendrocytes (*WBP2NL*, *CEP192*, *LRRIQ3*, *ZCWPW1*, *SNORC*, *RFT1*, *CEP192* and *SCN11A*), 1 with endothelial cells / oligodendrocytes (*STIMATE*), 2 with astrocytes / excitatory neurons / microglia's (*ARL17B* and *ARL17B*), 5 with astrocytes / oligodendrocytes / other neurons (*CWF19L1*, *KYAT3*, *KYAT3*, *CWF19L1* and *KYAT3*), 1 with endothelial cells / microglia's (*BTN3A2*), 1 with astrocytes / excitatory neurons (*POM121L2*), 2 with excitatory neurons / other neurons (*CCDC65* and *HYAL3*), 1 with astrocytes / endothelial cells / microglia's (*ERAP2*) and 2 with endothelial cells / excitatory neurons (*PILRB* and *UPK1A*). Of these, 22 eQTL instruments passed the significance threshold for both MR and colocalization (*RHEBL1*, *PBRM1*, *SLC22A23*, *FAM180B*, *DHRS11*, *C1QTNF4*, *NPIPB7*, *ID4*, *KANSL1*, *LRRIQ3*, *RHEBL1*, *FPGT*, *ABITRAM*, *ZCWPW1*, *ESR2*, *SLC35G6*, *SLC22A23*, *AP2B1*, *SMIM19*, *SNF8*, *SYNDIG1L* and *GALNT18*), 29 eQTL instruments passed the significance threshold only for MR (*NPIPB7*, *AKTIP*, *SEMA3F*, *ZMAT2*, *TEX14*, *CUEDC2*, *GATB*, *AFF3*, *GOLGA6L10*, *SCG3*, *ZNHIT3*, *GMPPB*, *WBP2NL*,

ELOVL7, *GMPPB*, *ARL17B*, *CRHR1*, *ARL17A*, *AFF3*, *FCER1A*, *CUEDC2*, *TIE1*, *PILRA*, *PILRB*, *ARTN*, *CHRNB1*, *HIST1H3C*, *FIBP* and *HIST1H1C*) and 37 eQTL instruments passed the significance threshold only for colocalization (*ARMC1*, *ELP1*, *RABGAP1L*, *THSD7B*, *ZNF502*, *TFB1M*, *SYNDIG1L*, *CEP192*, *FAM107B*, *DNAH11*, *C20orf27*, *SIRPB1*, *ZNF501*, *PIAS2*, *ELOA3B*, *ELOA3D*, *RRP12*, *NT5C2*, *ZNF501*, *EEF1AKMT2*, *TRPM7*, *ZNF502*, *CNOT9*, *COX6B1*, *UPK1A*, *CWF19L1*, *CYB5D2*, *OLR1*, *FAT3*, *CEP192*, *TGM5*, *SCN11A*, *CZIB*, *SNX4*, *DHRS11*, *LAMC3* and *SCG3*).

Brain volume

MR analysis was performed for several brain volume outcomes for amygdala, caudate, hippocampus, intercranial, nucleus accumbens, pallidum, putamen and thalamus[296]. Of which, there were 2 significant findings for intracranial volume that also passed colocalization. The findings suggest that decreasing the expression of *KANSL1* (WR=-14375, p=5.45×10^{-8}) and *SPPL2C* (WR=-44730, p=1.04×10^{-8}) increases brain volume. Interestingly, these two genes reside on the MAPT haplotype described above.

We also evaluated 17 of the 18 Brain volume associated eQTL instruments for an interaction effect with cell type proportions. In total, 6 eQTL instruments were significant ieQTLs, 1 with endothelial cells (*MYLK2*), 1 with excitatory neurons (*KANSL1*), 2 with oligodendrocytes (*KCNS3* and *SMIM22*), 1 with microglia's / oligodendrocytes (*SEPTIN12*) and 1 with astrocytes / excitatory neurons / microglia's (*ARL17B*). Of these, 1 eQTL instruments passed the significance threshold for both MR and colocalization (*KANSL1*) and 4 eQTL instruments passed the significance threshold only for colocalization (*KCNS3*, *SEPTIN12*, *SMIM22* and *ARL17B*).

Mendelian disease overlap

Of the significant WR findings that pass colocalization, 23 gene-indication pairs overlap a gene annotated in the DDG2P database of Mendelian neurodevelopmental disorders, of which 13 overlapped with brain-related disease traits, including Alzheimer's disease, depression, epilepsy, multiple sclerosis and schizophrenia (**Supplementary Table 12**). The gene function is described in the section above. These findings suggest these genes could play a role in the etiology of multiple disorders. Five findings are in the same direction of effect with Mendelian diseases, *CLCN3*, *CYP24A1*, *HSD3B7*, *PCCB, SETD1A* and *SLC39A13*. Targeted therapies for these genes could be efficacious for multiple indications. For example, loss of function mutations in *PCCB* cause autosomal recessive propionic acidemia[297], a metabolic disorder caused by deficiency of propionyl-CoA carboxylase, causing accumulation of toxic compounds in the blood, including propionyl-CoA, propionic acid, ketones and ammonia[298]. Interestingly, this disorder is associated with visual hallucinations and psychosis, and the psychiatric symptoms last longer than the metabolic imbalance[299], suggesting that metabolic imbalance arising from *PCCB* deficits could also be driving a neuropsychiatric clinical presentation, as suggested by the WR findings that decreased *PCCB* expression increases SCZ risk.

Systematic colocalization comparison of AD risk GWAS and cortex eQTLs

Method

For AD, we first sought out to compare colocalization of *MetaBrain* Cortex-EUR *cis*-eQTLs with the AD GWAS findings by Schwartzentruber *et al.*[70] in which smaller cortex or microglia eQTL datasets were used including BrainSeq[300], ROSMAP[28], xQTL-eQTL[28], AMP-AD including CMC[29] and primary microglia[301]. We examined 36 genome-wide significant AD loci excluding the *APOE* locus. Nine loci were found with multiple independent signals so conditional analysis was done using GCTA-COJO with the 1000 Genome European LD reference panel. Colocalization was then tested for all independent signals against primary Cortex-EUR *cis*-eQTLs. Our analysis was split into two parts: first, we aimed at replicating 587 pair-wise protein-coding gene brain eQTL colocalizations examined in Schwartzentruber *et al.*[70]; second, we aimed at including all *cis*-eQTL protein-coding genes around AD GWAS loci additionally identified in *MetaBrain* Cortex-EUR, by extracting all significant *cis*-eQTLs (FDR <0.05) within 500 Kb of the GWAS lead SNPs. The R package, coloc, was used to estimate the posterior probability of a shared causal variant between AD GWAS loci and Cortex-EUR *cis*-eQTLs. For each GWAS lead SNP, a +/- 500 Kb region was constructed and colocalization was tested using the default priors.

Results

In total, 833 pairwise colocalizations were tested (**Supplementary Table 13**). Of which, 33 findings colocalized (PP4 >0.7). We replicated (PP4>0.7) the majority of AD genes including *FCER1G*, *CR1*, *TREM2*, *CD2AP*, *CCDC6*, *TSPAN14*, *APH1B*, *KAT8*, *PRSS36*, *ZNF668*, *ACE*, *SLC39A13*, *BIN1*, *CHRNE* and *CD33*. Noticeably we found *BIN1* cortex eQTL colocalization which was only reported in microglia by Schwartzentruber *et al.*[70] In addition, we also identified several novel colocalization signals including *SLTM* at the *ADAM10* locus (PP4=0.76), *ADAMTS4* at the *ADAMTS4* locus (PP4=0.82), *TAS2R41* (PP4=0.79) and *TAS2R60* (PP4=0.98) at the *EPHA1* locus, *INPP5D* at the *INPP5D* locus (PP4=0.99), *MS4A4E* at the *MS4A4A* locus (PP4=0.75), *CLU* at the *PTK2B-CLU* locus (PP4=0.91) and *PRSS8* at the *VKORC1* locus (PP4=0.98). We also didn't find colocalization for several genes including *ABCA7*, *HSD3B7*, *NDUFS2*, *SPPL2A* which were identified in Schwartzentruber *et al.*[70] using ROSMAP data, although our results had much denser SNP coverage at these loci with much stronger eQTL signals. Further evidence is needed to interpret these loci and the potential causal genes.

Colocalization of top MR hits with opposite effect directionalities between *MetaBrain* and eQTLGen

We identified 1,587 top MR hits using *MetaBrain* Cortex-EUR (**Supplementary Table 12**) that passed the suggestive threshold ($p<5x10^{-5}$). After comparing the eQTL effect between Cortex-EUR and eQTLgen for the MR instruments, we found 1,016 MR top hits of which the Cortex-EUR instruments were significant (p<0.05) in eQTLgen, but 235 MR hits showed allelic discordance between Cortex-EUR and eQTLgen. We then checked if the Cortex-EUR instruments are in high LD with the top eQTLgen eQTL for the allelic discordant MR hits and found 62 with *MetaBrain* instruments in high LD with the top eQTLGen eQTL (r^2 >0.8). (**Supplementary Table 15**) Colocalization was then checked for these 62 MR hits in both

MetaBrain and eQTLgen to identify potentially shared causal genes between cortex and blood but with opposite directionalities for neurological traits. Colocalization methods were described in the Methods section of the main text. As a result, we found 25 MR top hits that colocalized with both *MetaBrain* Cortex-EUR and eQTLgen, and 9 of them also passed the MR Bonferroni correction: *DBN1* for intelligence and years of schooling, *ZNF746* and *AVIL* for multiple sclerosis, *SCFD1* for ALS, *KCTD13* for SCZ, *GATAD2A* for SCZ and years of schooling and *ZCWPW1* for years of schooling (**Supplementary Figure 22**).

MR comparison between *MetaBrain* and eQTLGen in multiple sclerosis

MS is hypothesized to be largely mediated by the immune system as an outcome of an inflammatory insult to the central nervous system and peripheral tissues. As both blood and brain cell types could play an important role in the etiology of the disease, we compared the MR results using *cis*-eQTL instruments in both *MetaBrain* Cortex-EUR and eQTLgen blood tissue, primarily to assess whether or not there were significant MR findings that show discrepant directions of effect between the tissue types.

For each data set, we selected genome-wide significant eQTLs at $p<5x10^{-8}$ cut-off and LD clumped to form a set of independent eQTLs for each study. The *MetaBrain* instruments consisted of 10,510 eQTLs across 8,949 genes and the eQTLGen instruments consisted of 41,157 eQTLs across 16,189 genes. We looked up the SNP effect in the MS GWAS for these instruments, harmonised the effects and performed MR to obtain a set of Wald ratios (WR) estimating the causal effect between gene expression and MS pertaining to the eQTL in each study. The WR measures the change in MS risk per unit change in gene expression through the effect allele of the instrumenting eQTL. A positive WR would indicate that increased gene expression is related to increased MS risk, whereas a negative WR would indicate that decreased gene expression is related to increased multiple sclerosis risk. Therefore, if the WRs agree between studies, then there is agreement on the direction of the expression effect acting on MS risk (i.e., whether promoting or inhibiting the gene would increase MS risk). In total, the *MetaBrain* WR set consisted of 9,392 WRs across 8,295 genes and the eQTLGen WR set consisted of 34,044 WRs across 16,567 genes.

We first assessed whether the MR results agreed between *MetaBrain* and eQTLGen for each gene, comparing the WRs on the different SNP instruments selected within each study. This represents the naïve analysis where, for example, a researcher tries to infer genes causally related to MS by conducting MR relying on eQTLs from blood only (i.e., blind to brain specific tissue effects). For genes which were instrumented by more than one SNP, we selected the top hit WR (lowest p-value) to do the comparison on, removing the trans-chromosomal eQTLGen instruments which were not on the same chromosome as the *MetaBrain* instruments. This reduced the *MetaBrain* WR set by 12% from 9,392 to 8,295 WRs and the eQTLGen instrument set by 82% from 34,044 to 5,919 WRs unique to the gene. We then compared the *MetaBrain* instruments against eQTLGen and found that the proportion of the WR effects which agreed to be very low: 2,291 (38.7%) of the 5,919 shared instrumented genes showing WRs with opposite direction between the studies (**Supplementary Table 14A, Supplementary Figure 23, top panel**). We observed no major change in the WR agreement among genes which showed robust association with multiple sclerosis (at WR p-value $<5x10^{-5}$). Within the 103 genes associated in

MetaBrain 36 genes (35.0%) had an opposite WR to eQTLGen and within the 152 genes associated in eQTLGen 56 genes (36.8%) had an opposite WR to *MetaBrain*.

Taking a closer look at the 209 top MR hits (**Supplementary Table 12**) identified for MS in *MetaBrain* Cortex-EUR, we identified a total of 47 genes that did not have a significant (p<5e-8) *cis*-eQTL in blood tissue, of which 15 genes (*RRP12*, *PVR*, *MARK3*, *RGS1*, *SLC12A5*, *CCDC155*, *MYNN*, *HIST1H1D, ASPHD1*, *LIME1*, *ZNHIT3*, *MYO19*, *SCO2*, *PIGW* and *ATG16L2*) also passed colocalization in Cortex-EUR (**Supplementary Table 16**). Interestingly, we also identified 29 genes where MR in Cortex-EUR and eQTLGen showed both suggestive signal ($p<5x10^{-5}$) but opposite directionalities suggesting potentially tissue-dependent putative causal genes. Furthermore, three of them (*AVIL*, *KCTD13* and *ZNF746*) also passed colocalization in both Cortex-EUR and eQTLGen, and the top *MetaBrain* Cortex-EUR eQTL was also in high LD with or the same as the top eQTLGen eQTL. (**Supplementary Figure 24**). We then compared MR results on the same SNP instrument to account for the discrepant WRs that could be due to the *MetaBrain* and eQTLGen eQTLs not being in LD (i.e., the instruments selected for each study are not sharing the same causal variant in the region). In the previous comparison, only 178 (3%) of the WRs had been derived from the same SNP instrument. This would represent the scenario where, for example, a researcher is able to incorporate information on brain tissue eQTL effects to improve specificity of blood eQTL based MR analysis to identify the causal genes in MS. To do the comparison we looked up the full *MetaBrain* instrument set (all 9,392 eQTLs) in the eQTLGen study and re-performed the MR between these eQTLGen effects and MS. We found a shared instrument for 7,274 (77.4%) of the 9,932 *MetaBrain* eQTLs within eQTLGen which we could conduct the MR on. As the eQTLs from the lookup in eQTLGen will not always be significant at the $p<5x10^{-8}$ level this could result in poorly calibrated standard errors for the WR. Therefore, to account for the standard error in the eQTL instrument, we computed the standard errors in the WRs with the second term approximation in the Taylor expansion included (see section: Taylor expansion for the Wald ratio standard error). Although fixing on the same SNP instrument improved the WR agreement between the studies, the discordancy remained relatively high (**Supplementary Table 14B**): 1,891 (26%) from the 7,274 WRs showed an opposite direction of effect. However, in contrast to the previous comparison done on the same genes, the WR agreement did improve for genes associated with multiple sclerosis (WR p-value $<5x10^{-5}$) when also restricted to the same instruments. Within the 124 genes associated in *MetaBrain* 19 genes (15.3%) had opposite WRs to eQTLGen and within the 75 genes associated in eQTLGen 8 genes (10.6%) had opposite WRs to the *MetaBrain* study.

Due to our WR comparison being limited to the set of eQTL instruments which intersect with the MS GWAS only, we also performed a comparison on the eQTL effect sizes across all the instruments available. As we anticipated, the findings from this analysis were consistent with the MR comparison with a considerable proportion of the eQTL effects showing opposing expression effect directions. We found that 7,986 of the 10,510 *MetaBrain* instruments (76.0%) were present in eQTLGen, of which 2,066 eQTLs (25.9%) had opposite direction of expression effect. Of which, 5,269 (66.0%) of these eQTLs reached $p<5x10^{-8}$ cut-off used to select instruments, and 1,019 eQTLs (19.3%) had opposing expression effect in *MetaBrain*. In the reverse lookup, 23,968 of the 41,157 eQTLGen instruments (58.2%) were present in *MetaBrain*,

of which 7,826 eQTLs (32.7%) had opposing expression effect. 10,987 (45.8%) of these eQTLs reached $p<5x10^{-8}$, of which 2,432 (22.1%) showed opposing expression effect in eQTLGen.

Of the 135 genes with MR findings in Cortex-EUR for MS, we identified 28 genes without a significant eQTLGen instrument, including 3 genes (*SLC12A5, CCDC155* and *MYNN*) for which we found both MR significance and colocalization in *MetaBrain* (**Supplementary Table 16**). For 25 MS genes, we were able to compare gene expression levels in blood and different brain regions using GTEx samples. We downloaded median gene expression from https://gtexportal.org/home/datasets (GTEx_Analysis_2017-06-05_v8_RNASeQCv1.1.9_gene_median_tpm.gct.gz). The majority (n=16; 64%) had almost no expression in blood, including SLC12A5, 3 (12%) had lower expression in blood than in cortical tissues, 2 (8%) had comparable expression in blood and cortical tissues (CCDC155 and MYNN) and 4 (16%) had higher expression in blood than in cortical tissues (**Supplementary Figure 25**).

Trans-eQTL results

Trans-eQTLs in the 7p21.3 locus

We performed *trans*-eQTL analysis using four different approaches. We first created a dataset consisting of all Cortex-EUR and Cortex-AFR datasets, while excluding the ENA dataset (n=2,759). We then performed *trans*-eQTL analysis on this dataset while not removing any PCs, and after removing 100 PCs (**Supplementary Figure 7**). This yielded 2,791 (0PCs) and 737 (100 PCs) *trans*-eQTLs respectively (**Supplementary Table 17**). We observed that 85% of the *trans*-eQTLs in the dataset that was not corrected for PCs were originating from 33 variants in a 7p21.3 locus containing the *TMEM106B* gene. We also observed that these 7p21.3 eQTLs generally had a higher heterogeneity than *trans*-eQTLs not located on chr7 (**Supplementary Figure 26**), suggesting that their effect sizes are not consistent across the different datasets. Upon further investigation, we observed that the majority of the *trans*-eQTL signal of the 7p21.3 *trans*-eQTLs was specific to the included AMP-AD datasets, which include Alzheimer's disease (AD) patients. We therefore also reperformed the *trans*-eQTL analysis while excluding AMP-AD datasets (n=1,755), this time correcting for 0PCs and 80PCs (**Supplementary Figure 7**). In this analysis, we identified 64 (0PCs) and 221 (80PCs) *trans*-eQTLs, respectively (**Supplementary Table 17**). Excluding AMP-AD datasets removed the 7p21.3 *trans*-eQTLs, further suggesting that these effects were specific to the AMP-AD dataset.

However, the 7p21.3 locus containing the *TMEM106B* gene has been described in the context of AD before. First, it has previously been associated with Frontotemporal lobar degeneration (FTLD)[302], and more recently with AD as well[303]. Second, the locus has been associated with changes in predicted neuron proportions[304], as well as changes in gene expression levels[305,306]. Considering these previous observations, we decided to further investigate the *trans*-eQTLs from this locus.

The majority (72%) of these *trans*-eQTLs were associated with two SNPs, rs11974335 and rs10950398 (R^2=0.98), which are both located in an intron of *TMEM106B* and were associated with 1,002 and 699 genes, respectively (**Supplementary Figure 27a and b**). QQ-plots of associations from the 7p21.3 locus for SNPs with a significant *trans*-eQTL association when not correcting for PCs showed a high lambda inflation of 2.14, which considerably decreased to 1.05 when correcting for 100 PCs (**Supplementary Figure 27c**). When performing *cis*-eQTL analysis in the same dataset as was used for *trans*-eQTL analysis (Cortex-EUR + AFR, but without ENA), we did observe a significant *cis*-eQTL for *TMEM106B* (**Supplementary Table 2**), although this effect was weaker ($p=2x10^{-7}$) than the *cis*-eQTL on nearby gene *THSD7A* ($p=7x10^{-19}$), and not linked to rs1990622 (R^2 <0.22). The majority of the *trans*-eQTLs genes were significantly correlated with *THSD7A* and *TMEM106B* (**Supplementary Figure 27d**), complicating the identification of a potential causal *cis*-eQTL gene in this region that might drive the observed *trans*-eQTLs.

Of note, the *trans*-eQTLs in the 7p21.3 locus included rs1990622 (R^2=0.96 with rs11974335), an index variant for FTLD[302] located downstream of *TMEM106B*, and is in high LD (R^2=0.97) with recently identified AD associated variant rs13237518[303]. Variant rs1990622 had a *cis*-eQTL on *THSD7A* (SNP–TSS distance >411Kb; **Supplementary Figure 27e**). The FTLD risk allele rs1990622-A decreased expression of this gene, which is suggested to be involved in neuro-angiogenesis[307] (**Supplementary Figure 27e**). rs1990622 was associated with 46 *trans*-eQTL genes, all of which were negatively regulated by the FTLD risk allele (**Supplementary Figure 28**). Downregulated genes included genes involved in calcium transport such as *CALB2*, and *CBLN1*, and potassium transport, such as *KCND3*, *KCHN5* and *KCTD2*. Collectively, downregulated *trans*-eQTL genes for rs11974335, rs10950398 and rs1990622 were enriched for neuron related processes, such as synaptic signaling ($p=1.3x10^{-28}$) and nervous system development ($p=2.9x10^{-21}$), while upregulated genes were enriched for gliogenesis ($p=1.6x10^{-8}$) and oligodendrocyte differentiation ($p=3.1x10^{-6}$; **Supplementary Table 18**).

While rs11974335 and rs10950398, rs1990622 are in high LD (R^2>0.9), we observed only a limited number of *trans*-eQTL genes for rs1990622. rs11974335 and rs10950398 were tested in only 6 datasets with WGS derived genotypes, the majority of which were AMP-AD datasets, while rs1990622 was tested in 17 datasets, suggesting that heterogeneity between datasets decreased the number of significant *trans*-eQTLs for rs1990622, also suggested by high I^2 values of chr7 *trans*-eQTLs (**Supplementary Figure 26)**. Indeed, *trans*-eQTLs for rs1990622 were highly heterogeneous across the included datasets, being most pronounced in the AMP-AD and UCLA_ASD datasets, and less so in other datasets of comparable sample size, such as CMC (**Supplementary Figure 28**).

As a potential source for heterogeneity, we considered that many of the observed *trans*-eQTLs might be due to differences in cell type proportions that might be caused by the presence of AD cases in the AMP-AD datasets. We therefore first associated the predicted cell type proportions with genotypes. We observed 21 associations from 15 SNPs (FDR <0.05; **Supplementary Table 19**). All associations were located in in the 7p21.3 locus. This included rs11974335 and

rs10950398, which were associated with predicted excitatory neuron proportions. The variant rs1990622 was not significantly associated with excitatory neurons (FDR=0.35; **Supplementary Figure 27e**), presumably because of high heterogeneity across the tested datasets, similar to the *trans*-eQTLs. However, the rs11974335-G allele was associated with a decrease in excitatory neurons, which is correlated to the FTLD rs1990622-A risk allele. Previously, the rs1990622-A FTLD risk allele was reported to be associated with a decrease in neuron proportions[308,309].

Like the observed *trans*-eQTLs, these neuron proportion associations were most pronounced in the AMP-AD datasets. However, when comparing average excitatory neuron proportions in AMP-AD with those in other datasets, we did not observe many significant differences (**Supplementary Figure 27e**; **Supplementary Table 20**). Nevertheless, we observed a strong relationship between the *trans*-eQTL Z-scores and the correlation of *trans*-eQTL gene expression levels and neuron proportions (R^2 >0.88 for rs11974335 and rs10950398; R^2 =0.1277 for rs1990622; **Supplementary Figure 27f; Supplementary Table 21**). Comparing Alzheimer cases versus controls in the AMP-AD dataset, we observed that neuron proportions were lower (**Supplementary Figure 29**) and that *trans*-eQTL Z-scores were slightly higher (**Supplementary Figure 27g**).

While these results suggest that it is likely that the observed 7p21.3 are specific to AMP-AD, and may be dependent on excitatory cell type proportions, we next considered testing these differences using interaction analysis. We performed interaction analyses with Decon-QTL to test each of the four sets of *trans*-eQTL results for interactions with the predicted cell type proportions. We observed that 392 of the 2,702 tested *trans*-eQTLs had a significant interaction (BH-FDR<0.05) with one of the predicted cell type proportions, when 0 PCs were removed, and AMP-AD datasets were included. Most of these interactions were with the excitatory (n=181), other neuron (n=91) and inhibitory neuron (n=84) cell types, and almost all interactions originated from the 7p21.3 locus (93%). Almost all neuronal interactions were no longer significant when the data was corrected for PCs (695 *trans*-ieQTLs and 7 other neuron interactions) or when AMP-AD datasets were excluded (61 *trans*-ieQTLs and 11 neuronal interactions; 7 excitatory neurons, 4 inhibitory neurons). These results are summarized in **Supplementary Table 22**.

We attempted to assess agreement of the cell type proportion interactions with *trans*-eQTL effect sizes with their matching cell types in the ROSMAP single nucleus dataset, but observed very few effects that were also significant in the single nucleus data (**Supplementary Table 23**). We observed highest rates of agreement for the interactions with the excitatory neuron cell type from the *trans*-eQTL results including AMP-AD without removal of PCs. This suggests that at least some of the *trans*-eQTLs can also be discovered in a single nucleus dataset. We note however that this specific single nucleus dataset consists of AMP-AD samples, and that the small sample size of this single nucleus dataset probably does not provide enough power to detect *trans*-eQTL effects, leading to low rates of agreement.

Next, we assessed whether the 7p21.3 *trans*-eQTLs showed any interaction with AD status. To this end, we used a linear model with an interaction term (expression ~ snp + case/control status + snp x case/control status) using the eQTLs and expression data that included AMP-AD samples, but was not corrected for PCs. Performing these analyses over all AD samples (N=412) and non-neurological control samples (N=951; we explicitly only included samples with such a label in this analysis) included in the meta-analysis did indeed show significant *trans*-eQTL interactions with AD status (423 significant out of 2,690 tested effects; **Supplementary Table 24**). Almost all SNPs were located on chromosome 7 (n=418; 99%). However, these effects were no longer significant when dataset indicator variables were included in the model, or when the analysis was limited to AMP-AD datasets only (**Supplementary Figure 27h**). Similarly, we also evaluated whether the predicted cell type proportion interactions were dependent on AD status, and observed 27 interactions with excitatory neurons, 23 with inhibitory neurons, and 4 with microglia, while testing 296 SNPs. However, most of these interaction effects were no longer significant when correcting for PCs (490 *trans*-ieQTLs and 37 cell type proportion interactions; 30 inhibitory neurons, 7 microglia). Focusing the analysis on samples from only the AMP-AD datasets, none of interaction effects remained significant (**Supplementary Table 19)**.

Finally, we also considered other potential explanations for the observed 7p21.3 effects. For instance, we determined if the locus harbored any high frequency copy number variations. This is an unlikely scenario because CNVs in this region are extremely rare in gnomAD SVs v2.1. Furthermore, if a CNV would be the cause of the observed effects, we would expect the CNV to also have an effect in the datasets other than AMP-AD, unless the CNV would have been associated with AD. However, to our knowledge, such an association has not been identified in this locus so far. Another explanation could be population stratification. However, we considered this to be unlikely as well, because we corrected the expression data for MDS components derived from the genotype data, and because we reasoned that such population stratification should also impact other cohorts with mixed ancestries, such as the CMC dataset.

In other words, our analyses suggest that the observed 7p21.3 effects are specific to AMP-AD datasets, but cannot be significantly attributed to differences between AD cases and controls. Consequently, we speculate that another technical or biological factor present in the AMP-AD datasets, that is not captured by the information available to us, may explain these associations.

Cis-eQTL index SNP enrichment for *trans*-eQTL SNPs

461 (88%) of the *trans*-eQTL SNPs identified when correcting for 100 PCs were associated with a significant *cis*-eQTL in Cortex-EUR. However, this overlap also included SNPs that were not necessarily the *cis*-eQTL index SNPs (i.e.: those with the strongest association with a gene), but could rather be variants tagging the index SNP because of LD. Within the set of SNPs we tested for *trans*-eQTLs that also overlapped with *cis*-eQTL SNPs in Cortex-EUR, we therefore determined how many SNPs were a *cis*-eQTL index SNP. 150 (33%) of the 461 significant *trans*-eQTL SNPs overlapping a *cis*-eQTL SNP were also the *cis*-eQTL index SNP, whereas of the 109,172 SNPs that did overlap a *cis*-eQTL SNP but that were not a *trans*-eQTL SNP, 15,745 (14.42%) were index SNPs, which represents an enrichment (Fisher exact test p-value=1.2×10^{-28}). This indicates that *cis*-eQTL index SNPs more often yield trans-eQTL effects in brain as

compared to other *cis*-eQTL variants. 29 were also *cis*-eQTL SNPs in tissues other than cortex, suggesting that *trans*-eQTLs can also be observed for *cis*-eQTLs index SNPs identified in other tissues (**Supplementary Table 17**).

Comparison of predicted cell count proportions between AD patients and neurological controls

To assign Alzheimer's disease status for AMP-AD samples we used either CERAD or cogdx score, whichever was available. For cogdx, individuals with a score of 4 or 5 were assigned Alzheimer's disease status, and individuals with a score of 1 as non-neurological control. For CERAD, individuals with a score of 3 were assigned AD status, and individuals with score of 1 as non-neurological control. Individuals with other gogdx or CERAD scores were not included in this comparison.

Gene co-regulation network

We previously have done this for a heterogenous set of RNA-seq samples spanning across all available tissue types and cell lines (n=31,499)[310,311], which showed that such a co-regulation network can be informative for interpreting GWAS studies[64] and helpful in the identification of new genes that cause rare diseases[65].

For the gene co-regulation network, gene expression was quantified using Kallisto[5] (version 0.43.1) to be in line with the gene expression quantification for the gene co-regulation network as described by Deelen *et al*.[311] CRAM files created during RNA-seq processing for the eQTL analysis were converted to FASTQ files using SAMtools[312] (version 1.9). Quantification was done against the Ensembl[313] v98 transcriptome with the patch chromosomes removed. The index was built using default options, with k-mer size = 31. For both paired-end and single-end samples the --bias option was used. For the paired-end samples all other options used default values. For single-end samples --fragment-length=200 and --sd=20 were used, for all other options the default values were used.

After transcript quantification, transcript counts were summed to gene counts using GENCODE[24] v32 primary assembly GTF for transcript to gene mapping. Genes that showed zero variance over all samples were removed for further analysis. Raw counts were quantile normalized before running PCA analysis to identify and remove possible outliers, but no outliers were detected, likely because only samples that passed the QC for the eQTL analysis were included. Subsequently, raw counts were normalized by the median of ratios method described in DESeq[314] before technical covariates identified for the eQTL analysis were removed. From the DESeq[314] normalized and covariate removed expression data a gene-gene Pearson correlation matrix was calculated and eigenvector and eigenvalues were calculated on this matrix using eigenvalue decomposition. The eigenvectors were centered and scaled and a gene-gene Pearson correlation matrix was calculated on the eigenvector matrix.

For 6 types of gene set databases (KEGG, REACTOME, HPO, GO Molecular Function, GO Biological Process, and GO Cellular Component) coregulation and gene set predictions were

calculated with GeneNetwork. The gene set predictions use the number of eigenvectors to include as a parameter. To determine the optimal number of eigenvectors to use we repeated the gene set predictions for n eigenvectors between 25-1000 with steps of 25, and 1000-2000 with steps of 200, and for each gene set database selected the number of eigenvectors with the highest mean AUC (**Supplementary Figure 32**). Heatmaps of the Pearson correlation of the AUC values between different steps shows that there is not much difference in AUC's once a certain number of eigenvectors has been reached (generally around 100-225 eigenvectors, **Supplementary Figure 33**). The AUC was calculated using a leave-one-out procedure described previously[311]. Given the optimal number of eigenvectors, we created for all genes (coding and non-coding) enrichment scores for each of the 6 databases.

Gene prioritization using Downstreamer

Overview of Downstreamer methodology

In short, Downstreamer associates a gene level prioritization score (GWAS gene Z-scores) to a gene-gene co-regulation matrix to find genes that have many connections at the expression level to genes inside GWAS loci (core genes). In addition, Downstreamer can identify pathway enrichments by switching the co-regulation matrix for pathway annotations. Downstreamer implements a strategy that can do these associations while accounting for LD structure and chromosomal organization. Downstreamer operates in two steps: In the first step, the GWAS gene Z-scores are calculated for the GWAS trait and a null distribution. In the second step, the GWAS gene Z-scores are associated with the phenotypes outlined above. The details on these steps are outlined in the sections below. The Downstreamer method is described in more detail in a recent preprint by Bakker *et al*[315].

Downstreamer step 1: Calculation of GWAS gene Z-scores

The primary step in the Downstreamer converts the GWAS summary statistics from p-values per variant to an aggregate p-value per gene (gene p-value) while accounting for local LD structure. This aggregate gene level p-value represents the GWAS signal potentially attributable to that gene.

First, we applied genomic control to correct for inflation in the GWAS signal. We then integrated the procedure from the PASCAL[316] method to Downstreamer so that we can aggregate variant p-values into a gene p-value while accounting for the LD structure. We aggregated all variants within a 25 Kb window around the start and end of a gene using the non-Finnish European samples of the 1000 Genomes (1000G) Project Phase 3 to calculate LD[317]. We calculated these GWAS gene p-values for all 20,327 protein-coding genes (Ensembl[313] release v75). The gene p-values were then converted to Z-scores for use in subsequent analysis. These are referred to as GWAS gene Z-scores.

To account for the long-range effects of haplotype structure which results in genes getting a similar GWAS gene Z-score, we use a generalized least squares (GLS) regression model for all

regressions done in Downstreamer. The GLS model takes a correlation matrix that models this gene-gene correlation.

To calculate this correlation matrix, we first simulated 10,000 random phenotypes by drawing phenotypes from a normal distribution and then associating them to the genotypes of the 1000G Phase 3 non-Finnish European samples. Here, we only use the overlapping variants between the real traits and the permuted GWASs to avoid biases introduced by genotyping platforms or imputation. We then calculate the GWAS gene Z-scores for each of the 10,000 simulated GWAS signals as described above. Next, we calculate the Pearson correlations between the GWAS gene Z-scores. As simulated GWAS signals are random and independent of each other, any remaining correlation between GWAS gene Z-scores reflects the underlying LD patterns and chromosomal organization of genes.

We simulated an additional 10,000 GWASs as described above to empirically determine enrichment p-values, and finally, we used an additional 100 simulations to estimate the FDR of Downstreamer associations.

Downstreamer step 2: Association of GWAS Z-scores with phenotypes

Pre-processing of GWAS gene Z-scores and pruning of highly correlated genes

For each GWAS, both real and simulated, the GWAS Z-scores were re-scaled to fit a normal distribution to ensure that outliers would not have disproportionate weights. Limitations in the PASCAL methodology result in ties at the minimum significance level of $1x10^{-12}$ for highly significant genes, so we use the minimum SNP p-value from the GWAS to identify the most significant gene and resolve the tie. We then used a linear model to correct for gene length, as longer genes will typically harbor more SNPs.

Sometimes, two (or more) genes will be so close to one another that their GWAS gene Z-scores are highly correlated, violating the assumptions of the linear model. Thus, genes with a Pearson correlation r ≥0.8 in the 10,000 GWAS permutations were collapsed into 'meta-genes' and treated as one gene. Meta-gene Z-scores were averaged across the input Z-scores. The GWAS Z-scores of the meta genes were scaled (mean=0, standard deviation=1).

Generalized least squares model to calculate pathway enrichment and core gene scores

We used a GLS regression to associate the GWAS gene Z-scores to the pathway Z-scores and co-regulation Z-scores. These two analyses result in the pathway enrichments and core gene prioritizations, respectively. We used the gene-gene correlation matrix derived from the 10,000 permutations as a measure of conditional covariance of the error term (**Ω**) in the GLS to account for the relationships between genes due to LD and proximity. The pseudo-inverse of **Ω** is used as a substitute for **Ω**-1

The formula of the GLS is as follows:

β=(XTΩ-1X)-1XTΩ-1y

Where β is the estimated effect size of pathway, term or gene from the co-regulation matrix, Ω is the gene-gene correlation matrix, X is the design matrix of real GWAS Z-scores and y is the vector of gene Z-scores per pathway, term or gene from the co-regulation matrix. As we standardized the predictors, we did not include an intercept in the design matrix and X only contains one column with the real GWAS gene Z-scores. We estimated the betas for the 10,000 random GWASs in the same way and subsequently used them to estimate the empirical p-value for β.

Pathway and gene set gene prediction
To identify pathway and disease enrichments, we used the following databases: Human Phenotype Ontology (HPO)[40], Kyoto Encyclopedia of Genes and Genomes (KEGG)[34], Reactome[35] and Gene Ontology[33] (GO) Biological Process, Cellular Component and Molecular Function. We have previously predicted how much each gene contributes to these gene sets, resulting in a Z-score per pathway or term per gene[311]. In a parallel step, genes were collapsed into meta-genes to ensure compatibility with the GWAS gene Z-scores, following the same procedure as in the GWAS pre-processing. Meta-gene Z-scores were calculated as the Z-score sum divided by the square root of the number of genes. Finally, all pathway Z-scores were scaled (mean=0, standard deviation=1).

Co-regulation matrix
To calculate core scores, we used three different brain-derived co-regulation networks: one based on all 8,544 brain samples, one limited to 6,527 cortex samples and one limited to 715 cerebellum samples. We compared this to a previously generated co-regulation matrix that is based on a large multi-tissue gene network[311]. This network was generated using the publicly available RNA-seq samples that were downloaded from the ENA (https://www.ebi.ac.uk/ena). After QC, 56,435 genes and 31,499 samples covering a wide range of human cell-types and tissues remained. We performed a PCA on this dataset and selected 165 components representing 50% of the variation that offered the best prediction of gene function. We then selected the protein coding genes and centered and scaled the eigenvectors for these 165 components (mean=0, standard deviation=1) such that each component was given equal weight. The first components mostly describe tissue differences[318], so this normalization ensures that tissue-specific-patterns do not disproportionately drive the co-regulation matrix. The co-regulation matrix is defined as the Pearson correlation between the genes from the scaled eigenvector matrix. The diagonal of the co-regulation matrix was set to zero to eliminate the disproportionate effect of the gene to its gene p-value. Pearson r values were converted to Z-scores.

Integrating *cis*-eQTLs with Downstreamer
Adding primary and non-primary eQTL variants from Cortex-EUR to the *Dowstreamer* analysis using the variantGene option did not markedly affect the key gene prediction scores (correlation in Z-scores ≥0.95; **Supplementary Figure 34**).

Downstreamer analysis in schizophrenia

The Downstreamer analysis, that uses co-expression to identify genes that co-expressed with genes in GWAS loci, prioritized 184 Bonferroni significant (p-value $\leq 2.55\times10^{-6}$) genes for Schizophrenia (**Supplementary Table 25**). Ten of these prioritized genes are located within 250kb of a genome wide significant GWAS hit, the remaining 174 genes are not directly identified by GWAS. An enrichment analysis revealed that 57 (31%) of these prioritized genes are known to cause Mendelian forms of Intellectual disability and/or global developmental delay (Enrichment p-values: HP:0001249: 3.76×10^{-17} and HP:0001263: 1.58×10^{-14}) (**Supplementary Table 25**). Another interesting enrichment was for the chromatin organization Reactome pathway, 26 (14%) of the prioritized genes are annotated to this pathway (p-value: 1.89×10^{-20}). Of these 26 chromatin organization genes there were 18 (69%) that are overlapping with Intellectual disability and global developmental delay genes. For rare neurodevelopmental disorders it is known that the causative gene is often involved in chromatin organization[319]. For schizophrenia there are also already indications of the importance of chromatin modeling, for instance the childhood onset of schizophrenia that is caused by a damaging variant in the chromatin remodeling *CHD2* gene[320]. Here it is worthwhile to mention *CHD2* is one of the 184 genes that we predict to be important for schizophrenia. The overlap of our prioritized genes with neurodevelopmental disorders fits the hypothesis that schizophrenia is, or partly is, a neurodevelopmental disorder[321] and implicates altered chromatin organization as one of the causative mechanisms for schizophrenia.

Downstreamer analysis in MS

Neurotrophins are polypeptides secreted by immunological cell types. In the brain, neurotrophin concentrations are important to promote the survival and proliferation of neurons as well as synaptic transmission. In MS patients, neurotrophin reactivity is higher in MS plaques, whereby neurotrophins are released by peripheral immune cells directly to the inflammatory lesions, suggesting a protective role of this signaling process[73,74]. Neurotrophins are also released by glial cells in the brain, including microglia and astrocytes, and their role in stimulating neuronal growth and survival could also contribute to an overall neuroprotective effect[75]. In the heterogeneous network, we observed high expression for these genes in immune-related tissues (**Supplementary Figure 31a**), supporting the “outside-in hypothesis” that the immune system may be a potential trigger for MS[34,76]. The brain specific network showed high expression in spinal cord and cerebellum but lower expression in cortex samples (**Supplementary Figure 31b**), which could be highlighting the specific biological processes taking place in these CNS regions that lead to disease. For example, the cerebellum is responsible for muscle coordination and ataxia occurs in approximately 80% of MS patients with symptoms[77]. We speculate that both dysregulation of the immune system and dysregulation of certain neurological processes is a prerequisite for developing MS.

Downstreamer analysis in ALS

The main results of this analysis are presented in the main text.

We used our recently developed clinical symptom prediction algorithm[311] to predict the functions of the genes prioritized by Downstreamer. Many of these genes were enriched for the 'gait

disturbance' HPO term. These genes are associated with ALS (highlighted in blue), brain-related disorders (including *DNAJC5*, *HTT*, *HUWE1*, *TSC1* and *YEATS2*) or muscle-related disorders (including *KMT2B*). While various loci have been identified for both familial and sporadic forms of ALS, the function of the positional candidate genes within these loci is still unclear.

Acknowledgements

ROSMAP

The results published here are in whole or in part based on data obtained from the AMP-AD Knowledge Portal (doi:10.7303/syn2580853) Study data were provided by the Rush Alzheimer's Disease Center, Rush University Medical Center, Chicago. Data collection was supported through funding by NIA grants P30AG10161, R01AG15819, R01AG17917, R01AG30146, R01AG36836, U01AG32984, U01AG46152, the Illinois Department of Public Health, and the Translational Genomics Research Institute.
Genotype data: doi:10.1038/mp.2017.20. RNAseq: doi:10.1038/s41593-018-0154-9. snRNA-seq: doi:10.7303/syn18485175

Mayo

The results published here are in whole or in part based on data obtained from the AMP-AD Knowledge Portal (doi:10.7303/syn2580853). Study data were provided by the following sources: The Mayo Clinic Alzheimer's Disease Genetic Studies, led by Dr. Nilufer Taner and Dr. Steven G. Younkin, Mayo Clinic, Jacksonville, FL using samples from the Mayo Clinic Study of Aging, the Mayo Clinic Alzheimer's Disease Research Center, and the Mayo Clinic Brain Bank. Data collection was supported through funding by NIA grants P50 AG016574, R01 AG032990, U01 AG046139, R01 AG018023, U01 AG006576, U01 AG006786, R01 AG025711, R01 AG017216, R01 AG003949, NINDS grant R01 NS080820, CurePSP Foundation, and support from Mayo Foundation. Study data includes samples collected through the Sun Health Research Institute Brain and Body Donation Program of Sun City, Arizona. The Brain and Body Donation Program is supported by the National Institute of Neurological Disorders and Stroke (U24 NS072026 National Brain and Tissue Resource for Parkinsons Disease and Related Disorders), the National Institute on Aging (P30 AG19610 Arizona Alzheimer's Disease Core Center), the Arizona Department of Health Services (contract 211002, Arizona Alzheimer's Research Center), the Arizona Biomedical Research Commission (contracts 4001, 0011, 05-901 and 1001 to the Arizona Parkinson's Disease Consortium) and the Michael J. Fox Foundation for Parkinson's Research. doi:10.1038/sdata.2016.89

MSBB

The results published here are in whole or in part based on data obtained from the AMP-AD Knowledge Portal (doi:10.7303/syn2580853). These data were generated from postmortem brain tissue collected through the Mount Sinai VA Medical Center Brain Bank and were provided by Dr. Eric Schadt from Mount Sinai School of Medicine.

CMC

Data were generated as part of the CommonMind Consortium supported by funding from Takeda Pharmaceuticals Company Limited, F. Hoffman-La Roche Ltd and NIH grants R01MH085542, R01MH093725, P50MH066392, P50MH080405, R01MH097276, RO1-MH-075916, P50M096891, P50MH084053S1, R37MH057881, AG02219, AG05138, MH06692, R01MH110921, R01MH109677, R01MH109897, U01MH103392, and contract HHSN271201300031C through IRP NIMH. Brain tissue for the study was obtained from the following brain bank collections: The Mount Sinai NIH Brain and Tissue Repository, the University of Pennsylvania Alzheimer's Disease Core Center, the University of Pittsburgh

NeuroBioBank and Brain and Tissue Repositories, and the NIMH Human Brain Collection Core. CMC Leadership: Panos Roussos, Joseph Buxbaum, Andrew Chess, Schahram Akbarian, Vahram Haroutunian (Icahn School of Medicine at Mount Sinai), Bernie Devlin, David Lewis (University of Pittsburgh), Raquel Gur, Chang-Gyu Hahn (University of Pennsylvania), Enrico Domenici (University of Trento), Mette A. Peters, Solveig Sieberts (Sage Bionetworks), Thomas Lehner, Stefano Marenco, Barbara K. Lipska (NIMH).

GTEx

The Genotype-Tissue Expression (GTEx) Project was supported by the Common Fund of the Office of the Director of the National Institutes of Health (commonfund.nih.gov/GTEx). Additional funds were provided by the NCI, NHGRI, NHLBI, NIDA, NIMH, and NINDS. Donors were enrolled at Biospecimen Source Sites funded by NCI\Leidos Biomedical Research, Inc. subcontracts to the National Disease Research Interchange (10XS170), Roswell Park Cancer Institute (10XS171), and Science Care, Inc. (X10S172). The Laboratory, Data Analysis, and Coordinating Center (LDACC) was funded through a contract (HHSN268201000029C) to the The Broad Institute, Inc. Biorepository operations were funded through a Leidos Biomedical Research, Inc. subcontract to Van Andel Research Institute (10ST1035). Additional data repository and project management were provided by Leidos Biomedical Research, Inc.(HHSN261200800001E). The Brain Bank was supported supplements to University of Miami grant DA006227. Statistical Methods development grants were made to the University of Geneva (MH090941 & MH101814), the University of Chicago (MH090951, MH090937, MH101825, & MH101820), the University of North Carolina - Chapel Hill (MH090936), North Carolina State University (MH101819), Harvard University (MH090948), Stanford University (MH101782), Washington University (MH101810), and to the University of Pennsylvania (MH101822). The datasets used for the analyses described in this manuscript were obtained from dbGaP at http://www.ncbi.nlm.nih.gov/gap through dbGaP accession number phs000424.v7.p2 on 02/27/2020.

NABEC

Data was collected from dbGAP accession phs001301.v1.p1, which was generated by J. R. Gibbs, M. van der Brug, D. Hernandez, B. Traynor, M. Nalls, S-L. Lai, S. Arepalli, A. Dillman, I. Rafferty, J. Troncoso, R. Johnson, H. R. Zielke, L. Ferrucci, D. Longo, M.R. Cookson, and A.B. Singleton. The NABEC dataset was generated at National Institute on Aging, Bethesda, MD, USA, Institute of Neurology, University College London, London, UK, The Scripps Research Institute, Jupiter, FL, USA, Johns Hopkins University, Baltimore, MD, USA, and the University of Maryland Medical School, Baltimore, MD, USA. NABEC was funded by Z01 AG000949-02. National Institutes of Health, Bethesda, MD, USA and Z01 AG000015-49. National Institutes of Health, Bethesda, MD, USA.

TargetALS

This data set was generated and supported by the following: Target ALS Human Postmortem Tissue Core, New York Genome Center for Genomics of Neurodegenerative Disease, Amyotrophic Lateral Sclerosis Association and TOW Foundation.

Braineac
Data was collected from doi.org/10.1038/s41467-020-14483-x, which was generated by Mina Ryten, David Zhang, and Karishma D'Sa, Sebastian Guelfi and Regina Reynolds. Mina Ryten, David Zhang, and Karishma D'Sa were supported by the UK Medical Research Council (MRC) through the award of Tenure-track Clinician Scientist Fellowship to Mina Ryten (MR/N008324/1). Sebastian Guelfi was supported by Alzheimer's Research UK through the award of a PhD Fellowship (ARUK-PhD2014-16). Regina Reynolds was supported through the award of a Leonard Wolfson Doctoral Training Fellowship in Neurodegeneration. All RNA sequencing data performed as part of this study were generated by the commercial company AROS Applied Biotechnology A/S (Denmark). We also would like to thank Guelfi *et al*.[124] for the use of their data.

European Nucleotide Archive
We would like to thank all donors and their families, principal investigators and their funding bodies for each of the projects included from the European Nucleotide Archive.

UCLA ASD, Bipseq, BrainGVEx and LIBD
Data were generated as part of the PsychENCODE Consortium supported by: U01MH103339, U01MH103365, U01MH103392, U01MH103340, U01MH103346, R01MH105472, R01MH094714, R01MH105898, R21MH102791, R21MH105881, R21MH103877, and P50MH106934 awarded to: Schahram Akbarian (Icahn School of Medicine at Mount Sinai), Gregory Crawford (Duke), Stella Dracheva (Icahn School of Medicine at Mount Sinai), Peggy Farnham (USC), Mark Gerstein (Yale), Daniel Geschwind (UCLA), Thomas M. Hyde (LIBD), Andrew Jaffe (LIBD), James A. Knowles (USC), Chunyu Liu (UIC), Dalila Pinto (Icahn School of Medicine at Mount Sinai), Nenad Sestan (Yale), Pamela Sklar (Icahn School of Medicine at Mount Sinai), Matthew State (UCSF), Patrick Sullivan (UNC), Flora Vaccarino (Yale), Sherman Weissman (Yale), Kevin White (UChicago) and Peter Zandi (JHU).

Software
R
python
Java

R packages
ggrepel[322],ggplot[323],ggpubr[324],viridis[325],lattice[326],gridextra[327],data.table[328],dplyr[329],readxl[330],scales[331],GGally[332],edgeR[333],ggExtra[334],gtable[335],matrixStats[336],naniar[337],plyr[338],reshape2[339],stringr[340],tidyr[341],tidyverse[342],topGO[343],g:Profiler[32]

Python packages
pandas[69], seaborn[344], matplotlib[345], scipy[51], numpy[346], statsmodels.api[347], tabix[348,349], sklearn[350],upsetplot[351,352],wget[353],beautifultable[354],sqlite3[355]

Supplementary Figure Legends

Supplementary Figure 1. European Nucleotide Archive brain sample selection. (a) Principal component (PC) analysis on the expression data of 74,052 samples included in the SkyMap database shows clustering on tissue type but also many outliers with high PC1 scores. **(b)** Coloring on single and paired-end sequencing shows no clear clustering. **(c)** Coloring single cell identifies the samples with high PC1 scores as single-cell samples. **(d)** Re-calculation of PCs on all samples with PC score <0 in panel A-D, after covariate correction. **(e)** Brain and Tissue score calculated by correlating expression of known tissue and brain samples to each of the PCs. **(f)** As panel F, cancer score was calculated by correlating expression of known cancer genes to all PCs.

Supplementary Figure 2. Assigning ancestry through principal component analysis. For each of the included datasets principal component (PC) scores are calculated on their genotypes. Samples are clustered with the 1000 genomes samples (left). The right panels show dataset genotype samples without 1000 genomes samples on the right projected on the same PCs. Using k-nearest neighbors clustering, samples are assigned an ancestry based on their closeness to the 1000 genomes samples of an ancestry.

Supplementary Figure 3. Properties of ENA genotype calls. (**a**) Allelic balance distribution of genotype calls in ENA before imputation shows clear distinction between homozygous reference, heterozygous and homozyous alternate calls. (**b**) Comparison of allele frequencies between ENA genotype calls and all other included datasets, after imputation shows highly similar allele frequencies independent on the type of variant. R is the Pearson correlation. P-value test statistic is based on Pearson's product moment correlation coefficient and follows a t distribution with 1,487,861-2 degrees of freedom. (**c**) Comparison of *cis*-eQTL Z-scores between meta-analysis and ENA shows high allelic concordance.

Supplementary Figure 4. RNA-seq alignment QC. The two main RNA-seq QC metrics that are used for filtering samples. (**a**) Percentage coding bases colored by dataset and (**b**) percentage of reads aligned colored per dataset. The red dotted line is the threshold for filtering (10% for coding bases and 60% for percentage reads aligned respectively). Triangles are samples filtered out by any of the RNA-seq QC metrics.

Supplementary Figure 5. Sample filtering by PCA. Principal component analysis (PCA) plot before normalization and covariate removal. For all plots the red line indicates 4 standard deviations from the mean and red dots are samples to be filtered out. **(a)** PCA on all samples after removing alignment QC outliers. **(b)** PCA on samples after removal of outlier samples from A. **(c)** PCA on samples after removal of outlier samples of A and B.

Supplementary Figure 6. PCA before and after covariate correction. (**a**) PC1 and PC2 on normalized expression data before covariate correction, colored on dataset. (**b**) PC1 and PC2 on normalized expression data after covariate correction.

Supplementary Figure 7. PCA optima for each dataset. *Cis*- and *trans*-eQTL analyses were performed with increasing numbers of principal components removed from the data. The optimal number of PCs to remove is dependent on the dataset.

Supplementary Figure 8. eQTL Z-score comparison between datasets. The pairwise spearman correlation and concordance of direction of the *cis*-eQTL Z-scores between all cohorts, and between each cohort and the meta-analysis Z-score. As two examples, (**a**) shows the Z-score comparison between Cortex-EUR eQTL datasets EUR-LIBD_h650 and EUR-UCLA_ASD, and (**b**) shows the Z-score comparison between the meta-analysis Z-score and the Cortex-EUR cohort EUR-AMPAD-ROSMAP-V2. (**c**) Correlation for each pairwise combination of cohorts between each other (small dots), and with the meta-analysis Z-scores (large dots). (**d**) Directional concordance for each pairwise combination of cohorts between each other (small dots), and with the meta-analysis Z-scores (large dots). The dots in (**c**) and (**d**) that correspond to the (**a**) and (**b**) plots are shown by the grey dottes lines. For both (c) and (d): Spinal cord n = 3, Hippocampus n = 3, Cortex-EUR n = 136, Cortex-AFR n = 6, Cerebellum n = 10, and Basal ganglia n = 3 cohort-cohort and cohort-meta combination. Boxplots show median (line in box), interquartile range (25th and 75th percentile, box), and minimum and maximum value (whiskers), excluding outliers (outliers are defined as less than Q1 – 1.5*(IQ3-IQ1) or greater than Q3 + 1.5*(IQ3-IQ1)). (**e**) Heterogeneity measured using I^2 shows generally low heterogeneity between dataset effect sizes.

Supplementary Figure 9. Reads mapping on patch chromosome version of MAPT. Number of reads mapped to the MAPT gene located on the primary assembly (ENSG00000186868) and the MAPT genes located on the patch chromosomes (ENSG00000276155 and ENSG00000277956). Each dot is an individual, and the color shows if they are homozygous reference (0/0), heterozygous (0/1), or homozygous alternative (1/1) for a SNP (rs34619181) located in the MAPT gene. Left plot compares counts mapped to ENSG00000186868 (ref) to those mapped to ENSG00000276155 (patch), middle plot compares ENSG00000186868 (ref) and ENSG00000277956 (patch), right plot compares ENSG00000276155 (patch) and ENSG00000277956 (patch).

Supplementary Figure 10. EQTL z-scores in the MAPT locus. Z-scores (y-axis) of the MAPT locus (x-axis) for all the datasets used in the Cortex-EUR meta-analysis. Left upper plot shows the meta-analysis Z-score. Blue dots are the top 100 associations.

Supplementary Figure 11. Properties of non-primary *cis*-eQTLs. (a) Percentage of tested genes that are a significant eGene per expression bin. Genes are grouped in ten bins based on average expression, from 10% lowest (bin 1) to 10% highest (bin 10). **(b)** Log10 of standard deviation (plus pseudo count) of all tested genes in each expression bin from A. For BIOS, n = 1,836 genes for bin 1, n = 1,835 genes for bins 2-10. For *MetaBrain*, n = 1,931 genes for bins 1-3, 5-6, and 8-10, and n = 1,930 genes for bins 4 and 7. Boxplots show median (line in box), interquartile range (25th and 75th percentile, box), and minimum and maximum value (whiskers), excluding outliers (outliers are defined as less than Q1 – 1.5*(IQ3-IQ1) or greater than Q3 + 1.5*(IQ3-IQ1)). Violin plot shows the distribution of the data. **(c)** g:profiler enrichment for all genes with a single independent eQTL effect. **(d)** g:profiler enrichment for all genes with multiple independent eQTL effects.

Supplementary Figure 12. Overview of different *cis*-eQTL agreement analyses. Left to right: comparisons between ancestries in Cortex, comparisons between brain regions in *MetaBrain* and comparisons between Cortex-EUR (excluding GTEx) and GTEx. Top to bottom show different

measures for agreement: R_b, AC, π_1, and correlation of allelic fold change (caFC). The R_b muscle SNPs focuses the analysis on a set of muscle eQTL SNPs detected in GTEx as an unbiased analysis. Top numbers in R_b, AC, and caFC are the shared number of eQTLs.

Supplementary Figure 13. Properties of cerebellum specific eQTLs. (**a**) UpSet plot of the number of eQTL genes per brain region for European datasets. (**b**) The distribution of log2(TMM+1) expression in cortex of the 472 eQTL genes that were only significant in cerebellum. Blue line is the minima of the fitted bimodal distribution (dotted red line) and is used as cut-off point in panel **c** (**c**) The expression in cortex (x-axis) and cerebellum (y-axis) of the 846 eQTL genes that were only significant eQTLs in cerebellum. The blue line is the cut-off from panel **b**. (**d**) The mean expression (dots) and standard deviation (error bars) of the 30 transcription factors that are enriched for binding to transcription sites around the 662 genes for cortex (x-axis) and cerebellum (y-axis). The 3 transcription factors that are labelled are lower expressed in cortex and higher expressed in cerebellum. For each transcription factor, n = 2,683 samples for Cortex and n = 492 samples for cerebellum.

Supplementary Figure 14. Correlation of effect sizes between Cortex primary eQTLs and GTEx eQTLs. The replication between primary *cis*-eQTLs of Cortex-EUR (discovery) with all the GTEx tissues (replication). The x-axis is the number of eQTLs that is significant in both discovery and replication, and the y-axis is the R_b.

Supplementary Figure 15. Comparison of meta-analysis Z-scores for eQTLs detected in the different *MetaBrain* datasets (x-axis), and eQTLgen (y-axis). P-value calculated using two-sided χ^2-test.

Supplementary Figure 16. Distributions of predicted cell proportions. Boxplots show median (line in box), interquartile range (25th and 75th percentile, box), and minimum and maximum value (whiskers). Violin plots show distribution of the data. **(a)** Predicted cell type proportions in *MetaBrain* cortex European using the PsychENCODE reference profile. Developmental cell types were discarded in the prediction of these cell types. The value above each violin denotes the average cell fraction in percentages over all samples. (**b**) Aggregated cell type proportions in *MetaBrain* Cortex-EUR. Subtypes of cells for excitatory neurons, inhibitory neurons and oligodendrocytes (OPC and oligodendrocytes) are summed together. The value above each violin denotes the average cell fraction in percentages over all samples. For both (**a**) and (**b**) n = 2,683 samples for all cell types.

Supplementary Figure 17. Replication of Cortex-EUR ieQTLs in Cortex-AFR. Each figure in this plot represents a comparison between Cortex-EUR (x-axis) and Cortex-AFR (y-axis). Each dot represents one *cis*-eQTL, and the legend shows the sample size, Pearson correlation coefficient, the AC, and, if applicable, the R_b and π_1 statistics. Each column is a comparison between equivalent cell types in both datasets. Each row illustrates a different filtering on which eQTLs are shown. The values denote the log interaction beta from Decon-QTL. (**a**) All overlapping (i)eQTLs (**b**) (i)eQTLs filtered on being significant in Cortex-EUR (**c**) (i)eQTLs filtered on being significant in each respective dataset. If applicable, the 15 (i)eQTLs with the lowest p-value are labelled. Colored bands indicate 95% confidence interval around regression line.

Supplementary Figure 18. Replication of cortex *cis*-eQTLs in snRNA-seq data from ROSMAP. Each figure in this plot represents a comparison between bulk RNA-seq (x-axis) and ROSMAP single-nucleus RNA-seq (y-axis). Each dot represents one *cis*-eQTL, and the legend shows the sample size, Pearson correlation coefficient, the AC, and, if applicable, the R_b and π_1 statistics. Each column is a comparison between equivalent cell types in both datasets. Each row illustrates a different filtering on which eQTLs are shown. The x-axis always denotes the log interaction beta from Decon-QTL, the y-axis always denotes the log beta of the eQTL effect in the single-nucleus dataset. (**a**) All overlapping (i)eQTLs (**b**) (i)eQTLs filtered on being significant in *MetaBrain* Cortex-EUR (**c**) ieQTLs filtered on being significant in each respective dataset. If applicable, the 15 ieQTLs with the lowest p-value are labelled. Colored bands indicate 95% confidence interval around regression line.

Supplementary Figure 19. Replication of cortex *cis*-eQTLs in snRNA-seq data from Bryois. Each figure in this plot represents a comparison between bulk RNA-seq (x-axis) and Bryois *et al*. single-nucleus RNA-seq (y-axis). Each dot represents one *cis*-eQTL, and the legend shows the sample size, Pearson correlation coefficient, the AC, and, if applicable, the R_b and π_1 statistics. Each column is a comparison between equivalent cell types in both datasets. Each row illustrates a different filtering on which eQTLs are shown. The x-axis always denotes the log interaction beta from Decon-QTL, the y-axis always denotes the log beta of the eQTL effect in the single-nucleus dataset. (**a**) All overlapping (i)eQTLs (**b**) (i)eQTLs filtered on being significant in *MetaBrain* Cortex-EUR (**c**) (i)eQTLs filtered on being significant in each respective dataset. If applicable, the 15 (i)eQTLs with the lowest p-value are labelled.

Supplementary Figure 20. Bulk interacting eQTLs replicating in single-nucleus data. Replication of cell type interaction eQTLs for *NKAIN1* **(a)**, *STMN4* **(b)**, *AMPD3* **(c)**, *FAM221A* **(d)**, *CD82* **(e),** and *CD38* **(f)**. First column**:** Violinplot of the eQTL effect in Cortex-EUR bulk RNA-seq. The x-axis shows the genotype alleles, the y-axis shows the gene expression in TMM counts, the colors indicate the SNP genotype, with yellow being the minor allele. Boxplots show median (line in box), interquartile range (25th and 75th percentile, box), and minimum and maximum value (whiskers). Violin plots show distribution of the data. Second column: Cell type interacting eQTL effect in Cortex-EUR bulk RNA-seq. The x-axis shows the estimated cell type proportion, the y-axis shows the gene expression in TMM counts, each dot represents a sample, and the colors indicate the SNP genotype, with yellow being the minor allele. Values under the alleles are Pearson correlation coefficients. Third and fourth column: Forest plot of the eQTL betas with effect direction relative to the minor allele when replicating the eQTL effect in ROSMAP single-nucleus data (third column; n=38) and Bryois *et al*. 2021 (fourth column; n = 196). Dots indicate eQTL beta, error bars indicate ±1 standard error. Each row denotes a cell type specific dataset: astrocytes (AST), endothelial cells (END), excitatory neurons (EX), inhibitory neurons (IN), microglia (MIC), oligodendrocyte precursor cells (OPC), oligodendrocytes (OLI), pericytes (PER) and endothelial cells (END). Cell types highlighted in bold reflect the equivalent to the cell type used in the interaction eQTL.

Supplementary Figure 21. Mendelian Randomization summary. Each plot is for a different trait (Intelligence, Intracranial volume, Putamen volume, Years of schooling, Alzheimer's disease, Amyotrophic Lateral Sclerosis, Depression (broad), Frontotemporal Dementia,

Parkinson's disease, Bipolar disorder, Generalized epilepsy, juvenile myoclonic epilepsy, multiple sclerosis and schizophrenia). For each SNP the effect allele (EA) is given, the eQTL beta of the EA on the given gene (left forest plot), the odds ratio (disease traits) or beta (quantitative traits) of the EA on the phenotype (right forest plot), and the Wald ratio p-value of the mendelian randomization analysis.

Supplementary Figure 22. Colocalization regional plots for nine significant MR findings in Cortex-EUR that were replicated in eQTLGen with allelic discordance. Regional plots were made for nine MR findings (*DBN1* for intelligence and years of schooling, *ZNF746* and *AVIL* for multiple sclerosis, *SCFD1* for ALS, *KCTD13* for SCZ, *GATAD2A* for SCZ and years of schooling, and *ZCWPW1* for years of schooling) in Cortex-EUR (top), eQTLGen (middle) and outcome GWAS (bottom) to show colocalization. These nine findings all passed Bonferroni threshold ($p<1.43x10^{-7}$) in Cortex-EUR, with eQTL effects replicated in eQTLGen ($p<0.05$), showed colocalization for both Cortex-EUR and eQTLGen but opposite directions of effect.

Supplementary Figure 23. Scatterplots comparing MR effects for multiple sclerosis derived using instruments from the *MetaBrain* versus eQTLGen studies. The top panel shows the Wald ratio comparison on the same gene but with the different SNP instruments selected by each study (matching on the top Wald ratio finding if gene instrumented with multiple SNPs in the study) and the bottom panel the Wald ratio comparison between *MetaBrain* instruments and eQTLGen matching on both the same gene and SNP instrument. Genes which showed opposite direction of Wald ratio effect between *MetaBrain* and eQTLGen are colored in red and the genes with the same direction in blue.

Supplementary Figure 24. Colocalization regional plots for three suggestive MR findings for multiple sclerosis that showed opposite directions of effect between Cortex-EUR and eQTLGen. Regional plots were made for three suggestive MR findings for MS (*AVIL*, *KCTD13*, *ZNF746*) in Cortex-EUR (top), eQTLGen (middle) and MS GWAS (bottom) to show colocalization. All three were suggestive signals in Cortex-EUR as well as eQTLGen ($p<5x10^{-5}$), showed colocalization for both Cortex-EUR and eQTLGen but opposite directions of effect.

Supplementary Figure 25. Expression of genes with opposite effects with eQTLGen. Log_{10} of median **e**xpression of brain and blood tissue samples in GTEx for 5 multiple sclerosis genes for which there are no significant eQTLgen instruments, but for which we did find significant MR and colocalization in *MetaBrain*.

Supplementary Figure 26. Heterogeneity of *trans*-eQTLs in Cortex. Measured by I^2. A high amount of heterogeneity is observed when the gene expression data is not corrected for PCs, which disappears when AMP-AD datasets are excluded. Similarly, heterogeneity decreases when the gene expression data is corrected for PCs.

Supplementary Figure 27. *Trans*-eQTLs in the 7p21.3 locus. (a) Dot plot showing the location of the significant *trans*-eQTLs with and without PCA correction. **(b)** Overview of the *TMEM106B* locus. **(c)** QQ-plots after correcting for 0 PCs or 100 PCs of all *trans*-eQTL associations between genes and SNPs in the 7p21.3 locus. SNPs were limited to those that had a significant *trans*-eQTL association when no PCs were removed. No cross-mapping correction

applied and created using estlambda2 function in R, using permuted p-values to determine expected □^{2}. (**d**) Correlation of *trans*-eQTL gene expression levels with *TMEM106B* and *THSD7A*. (**e**) Comparison of effect sizes between datasets of rs1990622-A for nearby genes TMEM106B and THSD7A, *trans*-eQTL gene CALB2, and predicted excitatory neuron proportion. Dots indicate beta, error bars indicate 95% confidence interval. Violin plots indicate distributions of predicted excitatory neuron cell counts per dataset with dots being the median value. Meta-analysis n=2,683. (**f**) Pearson correlations between *trans*-eQTL Z-scores and correlation of *trans*-eQTL genes with excitatory neuron proportions. (**g**) Correlation between *trans*-eQTL Z-scores observed in Alzheimer's disease cases versus non-neurological controls in the AMP-AD datasets. (**h**) Interaction between *CALB2 trans*-eQTL for rs1990622 and Alzheimer's disease status, using (left) all samples in Cortex-EUR with a Alzheimer's disease or non-neurological control label, (middle) limited to AMP-AD datasets, and (right) excluding AMP-AD. Boxplots show median (line in box), interquartile range (25th and 75th percentile, box), and minimum and maximum value (whiskers), excluding outliers (outliers are defined as less than Q1 – 1.5*(IQ3-IQ1) or greater than Q3 + 1.5*(IQ3-IQ1)). The x-axis shows the disease status, the y-axis shows the gene expression, each dot is a sample and is colored by the SNP genotype.

Supplementary Figure 28. Forest plots for rs1990622 *trans*-eQTLs. Forest plots for each of the *trans*-eQTL genes associated with rs1990622. Each plot shows the *trans*-eQTL beta (dots) and 95% confidence interval (error bars) for each of the included datasets and the meta-analysis. Effect directions are relative to the A allele of rs1990622. Sizes of dots are relative to sample size of each dataset. *Trans*-eQTL effects are most pronounced in AMP-AD datasets. Meta analysis n=2,683.

Supplementary Figure 29. Cell type proportions in Alzheimer's disease patients. Predicted cell count proportions for the AMP-AD samples that were used in the Cortex-EUR eQTL analysis for individuals with Alzheimer's disease and non-neurological controls. Each dot is the predicted cell proportion for one sample. Numbers under the box plots indicate the number of samples plotted. Values above the line are p-values from a two-sided t-test between groups. Boxplots show median (line in box), interquartile range (25th and 75th percentile, box), and minimum and maximum value (whiskers), excluding outliers (outliers are defined as less than Q1 – 1.5*(IQ3-IQ1) or greater than Q3 + 1.5*(IQ3-IQ1)).

Supplementary Figure 30. Replication of cortex *trans*-eQTLs (100 PCs) in snRNA-seq data from ROSMAP. Each figure in this plot represents a comparison between bulk RNA-seq (x-axis) and ROSMAP single-nucleus RNA-seq (y-axis). Each dot represents one *trans*-eQTL, and the legend shows the sample size, Pearson correlation coefficient, the AC, and, if applicable, the R_b and $\pi 1$ statistics. Each column is a comparison between equivalent cell types in both datasets. Each row illustrates a different filtering on which eQTLs are shown. The x-axis always denotes the log interaction beta from Decon-QTL, the y-axis always denotes the log beta of the eQTL effect in the single-nucleus dataset. (**a**) All overlapping (i)eQTLs (**b**) (i)eQTLs filtered on being significant in *MetaBrain* (**c**) (i)eQTLs filtered on being significant in each respective dataset. Colored bands indicate 95% confidence interval around regression line.

Supplementary Figure 31. (a) UMAP representation of heterogeneous multi-tissue gene network. Immune and blood cell types show increased gene expression levels for genes prioritized using *Downstreamer* for multiple sclerosis, while decreased expression is observed in brain related tissues. **(b)** Within *MetaBrain*, those same genes show lower expression in cortex, but higher expression in spinal cord and cerebellum.

Supplementary figure 32. Comparison of AUC distribution for different eigenvector cut-offs. The quality of the gene network that we built for *MetaBrain* is measured by an AUC for each gene derived from a leave-one-out procedure. One of the parameters to build the network is the number of eigenvectors to use after PCA over the gene correlation matrix. Here we show for the 6 annotation categories (KEGG, REACTOME, GO Biological Process, GO Molecular Function, GO Cellular Component, and HPO) the AUC mean (dot) and standard deviation (lines) at different eigenvector cut-offs. The red dot and line indicate the eigenvector cut-off that was used for that annotation category.

Supplementary Figure 33. **Heatmaps of the Pearson correlation of the AUC values between different eigenvector cut-offs.** Correlation was calculated between the different eigenvector cutoffs for the 6 annotation categories.

Supplementary Figure 34. Downstreamer with and without including eQTL information. The Downstreamer analysis was repeated while explicitly including *cis*-eQTL SNPs, but this did not alter results.

Supplementary Figure 35. Comparison between eQTL multiple testing correction methods based on permutations. (a) -log10 p-value comparison between EMP and FastQTL shows highly similar p-values for tested eQTLs. **(b)** $-\log_{10}$ FDR and q-value comparison between EMP and FastQTL shows that FastQTL produces more significant results. eQTL mapping pipeline p-values are capped at 4.946e-324 as this is the lowest value Java can return. **(c)** Comparison of $-\log_{10}$ q-values between mbQTL and FastQTL showing highly similar q-values. **(d)** Zoom in shows that major differences between methods are near significance thresholds, and that FastQTL q-values are slightly higher compared to those from mbQTL. (a-d) R is the pearson correlation. P-value test statistic is based on Pearson's product moment correlation coefficient and follows a t distribution with 18,140-2 (a, b) or 18,417-2 (c,d) degrees of freedom. **(e)** distance to TSS for EMP, FastQTL and mbQTL, visualized by ranking each eQTL (significant and not significant) by the nominal p-value. This shows that variance in TSS increases the less significant an eQTL is. Red dotted line indicates FDR/q-value < 0.05. Blue dotted line indicates FDR/q-value < 0.01.

Supplementary Figure 36. Pearson correlation heatmap of predicted cell fractions versus expression principal components. A heatmap showing the correlation between the predicted cell type proportions and TPM expression matrix principal components. The PCA components are determined over the full expression matrix (n genes = 57,886), a subset of which is directly used for the prediction of the cell type proportions. Each cell contains the Pearson correlation coefficient; blue denotes a negative correlation, red a positive correlation and white denotes no correlation.

Supplementary Figure 37. Decon-QTL nominal p-value per cell type. Decon-QTL nominal p-value distribution per cell type for all tested eQTLs.

Supplementary Figure 38. Decon-QTL multiple testing comparison. Pairwise comparison per cell type of the interaction FDR values calculated using Benjamini-Hochberg (BH; x-axis) and a permutation-based FDR (EMP; y-axis). Each point is an ieQTL and is colored as follows: green denotes significant in both analyses, blue denotes only significant on the x-axis, orange denotes only significant on the y-axis, and grey is not significant. The horizontal and vertical dashed lines show the significance threshold of FDR = 0.05. The r denotes the Spearman correlation coefficient.

Supplementary Figure 39. SnRNA-seq visualization by cell type. UMAP dimensionality reduction plot of 39 snRNA-seq samples from ROSMAP. Each dot represents a single cell (n=70,634). The dots are colored by their corresponding cell type: excitatory neurons (EX), oligodendrocytes (OLI), inhibitory neurons (IN), astrocytes (AST), oligodendrocyte precursor cells (OPC), microglia (MIC), pericytes (PER) and endothelial cells (END).

Supplementary Figure 40. SnRNA-seq visualization by cell type. UMAP dimensionality reduction plot of 39 snRNA-seq samples from ROSMAP. Each dot represents a single cell (n=70,634). The dots are colored by their corresponding cell type subcluster: excitatory neurons (EX), oligodendrocytes (OLI), inhibitory neurons (IN), astrocytes (AST), oligodendrocyte precursor cells (OPC), microglia (MIC), pericytes (PER) and endothelial cells (END).

Supplementary Table descriptions

Supplementary Table 1. Number of samples and individuals.

Sheet Genotype QC: The number of genotype individuals and samples pre-QC (**column C-H**) and post-QC (column I-N) for the different RNA-seq (**column A**) and genotype (**column B**) datasets. Columns are: **PreQC**: Number of initial genotype samples processed for QC. **PostQC**: Number of genotype samples left after QC filtering. **RNA-seq dataset**: Name of the complete dataset. **Genotype dataset**: Name of the genotype dataset. Some datasets have multiple genotype platforms, or multiple smaller datasets that are part of the larger RNA-seq dataset. **Individuals:** The number of individuals per dataset. **EUR**: Number of genotype samples per dataset of individuals of European ancestry. **AFR**: Number of genotype samples per dataset of individuals of African ancestry. **EAS**: Number of genotype samples per dataset of individuals of East-Asian ancestry. **SAS**: Number of genotype samples per dataset of individuals of South-Asian ancestry. **AMR:** Number of genotype samples per dataset of individuals of Ad Mixed American ancestry.

Sheet **RNA-QC**: The number of RNA-seq samples at different steps of QC and for different brain regions. Cells A2-F18 have the number of samples at different QC steps. Columns are: **Dataset**: dataset name. **Number of RNA-seq samples**: Number of RNA-seq samples processed to go through QC. **Alignment QC**: Number of RNA-seq samples left after filtering on alignment QC (e.g.:percent reads aligned). **RNA-seq PCA outliers - step 1**: Number of RNA-seq samples left after filtering samples >4SD from mean of PC1. **RNA-seq PCA outliers - step 2**: Number of samples left after recalculating PCA and again removing samples >4SD fom mean of PC1. **Covariate removal:** Number of samples left after covariate removal. **RNA Tissue grouping:** the meta-data across different datasets uses different granularity of tissue annotation. Tissues were grouped accordingly.

Sheet Sample Links: RNA-seq samples linked to genotype samples. Left top: numbers of RNA-seq sample linked to a genotype sample per dataset, per ancestry. Top right: number of unique individuals per dataset per ancestry. Middle: number of uniquely linked individuals per dataset, per ancestry and per tissue group. Bottom: numbers of individuals used from each dataset and ancestry for *cis*- and *trans*-eQTL analysis.

Sheet Mixup detection: sample mismatches between genotype and RNA-seq samples detected per dataset using MixupMapper.

Sheet eQTL Meta-analysis datasets: Number of samples selected from each dataset for each meta-analysis.

Supplementary Table 2. C*is*-eQTL summary statistics.

Cis-eQTL summary statistics listing index variant per gene (FDR<0.05). One sheet per eQTL discovery dataset. Genomic positions are GRCh38. eQTL Rank: whether the eQTL is a primary, secondary, tertiary, quaternary, or higher eQTL.

Supplementary Table 3. Number of *cis*- and *trans*-eQTLs. For each dataset the number of *cis*- and *trans*-eQTL SNPs, genes, and SNP-gene combinations found at q-value < 0.05 (*cis*-eQTLs) or FDR<0.05 (*trans*-eQTLs). Columns are: **Basalganglia, Cerebellum, Cortex, Hippocampus, Spinalcord**: the five different brain regions for which eQTL calling was done. **EUR:** Number of eQTLs with samples from European ancestry. **AFR**: Number of eQTLs with samples from African ancestry. **EAS**: Number of eQTLs with samples from East-Asian ancestry. **EUR+AFR, wo ENA, no PCA**: Number of eQTLs with samples from EUR and AFR ancestries, excluding

samples from the ENA cohorts, and using gene expression levels that were not corrected for principal components.

Supplementary Table 4. Gene set enrichment summary statistics for primary and higher rank eQTLs. Gene set enrichment summary statistics generated using g:Profiler for genes having only a primary eQTL effect (sheet Primary eQTL), and those also having a non-primary eQTL (sheet Non-primary eQTL), and their overlap (sheet Enrichment overlap).

Supplementary Table 5. *cis*-eQTL replication. Replication between *cis*-eQTLs of different *MetaBrain* regions and all GTEx tissues. For GTEx comparisons, discovery was performed in the Cortex-EUR dataset while excluding GTEx, and then replicated in each GTEx tissue. **Sheets ending with '-AC'**: allelic concordance. **Concordance**: proportion of shared eQTLs with the same direction. **Concordance (q-value<0.05):** proportion of shared eQTLs with same direction that are also significant at q-value<0.05 in the replication dataset. **Sheets ending with '-Rb'**: Rb analysis. R**b**: Rb score for replications. **Rb se**: Standard error of Rb. Rb and Rb se colums (GTEx muscle SNPs): Rb analysis performed with eQTL SNPs identified in the GTEx muscle tissue. **Sheets ending with '-Pi1'**: π_1.values for each replication analysis. **Sheets ending with '-aFC'**: allelic fold change correlations. Correlation of aFC: correlation of aFC values between discovery and replication dataset. Correlation of aFC (q-value<0.05): correlation of aFC values between discovery and replication dataset for those eQTLs that were also significant in the replication dataset.

Supplementary Table 6. Gene set enrichment summary statistics generated using g:Profiler for genes which have an eQTL effect in cerebellum and not in cortex, and are highly expressed in cortex.

Supplementary Table 7. eQTLgen *cis*-eQTL replication. *MetaBrain cis*-eQTLs (FDR<0.05) as discovery cohort and eQTLgen eQTLs as replication cohort. Top table: FDR<0.05 in MetaBrain discovery only. Bottom table: FDR<0.05 in both MetaBrain and eQTLgen datasets. **Shared**: number of shared eQTLs. **Concordant**: number of shared eQTLs that has the same allelic direction of effect. **Concordant over total**: proportion of concordant eQTLs over the total number of eQTLs discovered. **Concordant over shared:** proportion of concordant eQTLs over number of shared eQTLs. **Pi1**: π1.values for replications. **Rb:** R_b score for replications. **Rb_SE:** Standard error of R_b. R_b **P:** P-value of R_b (two-sided).

Supplementary Table 8. *Cis*-eQTL cell type deconvolution summary statistics. Gene: eQTL gene ensebl ID. **Gene symbol**: eQTL gene symbol. **SNP**: eQTL SNP. **Alleles**: SNP alleles. **Allele assessed**: the allele to which the betas are directed. **N**: the sample size. **HW pval**: the Hardy-Weinberg equilibrioum p-value. **Minor allele**: the minor allele. **MAF**: the minor allele frequency. **Overall z-score**: the meta-analysis eQTL z-score. **Columns ending with pvalue**: p-value for the cell-type interaction. **Columns ending with beta**: beta for the cell-type proportion term. **Columns ending with interaction beta**: beta for the genotype x cell-type interaction term. **Columns ending with BH-FDR**: the Benjamini-Hochberg FDR. **Columns ending with Perm-FDR**: the permutation FDR.

Supplementary Table 9. Replication of the *MetaBrain* cortex primary *cis*-ieQTLs. Sheet Cortex AFR: replication in *MetaBrain* Cortex-AFR**. Sheet ROSMAP Single-Nucleus:** replication in ROSMAP single-nucleus**. Sheet Bryois et al. 2021:** replication in Bryois *et al.* 2021 single-nucleus**. Gene**: eQTL gene ensebl ID. **Gene symbol**: eQTL gene symbol. **SNP**: eQTL SNP. **Alleles**: SNP alleles. **Allele assessed**: the allele to which the betas are directed. **Columns ending with N**: the sample size. **Columns ending with HW pval**: the Hardy-Weinberg equilibrioum p-value. **Columns ending with Minor allele**: the minor allele. **Columns ending with MAF**: the minor allele frequency. **Columns ending with interaction beta**: beta for the genotype x cell-type interaction term or single-cell eQTL. **Columns ending with pvalue**: p-value for the cell-type interaction or single-cell eQTL. **Columns ending with BH-FDR**: the Benjamini-Hochberg FDR. For the replication datasets this FDR is calculated over the discovery significant (Benjamini-Hochberg FDR <0.05) rows of the respective cell type.

Supplementary Table 10. eQTL SNPs in linkage disequilibrium with GWAS SNPs. The GWAS SNPs that are in high linkage disequilibrium (LD) with the *cis*-eQTL SNPs. Each sheet is a different M*etaBrain* eQTL dataset from EUR ancestries. The sheet Included Traits lists GWAS traits that were tested. Columns are: **eQTL rank**: the rank of conditional eQTLs (1=primary, 2=secondary, etc). **TraitId**: GWAS ID of the GWAS SNP. **Trait**: Name of the GWAS trait. **TraitSNP**: the GWAS variant. **TraitP**: GWAS p-value. **EQTLSNP**: the eQTL SNP. **EQTLP**: eQTL P-value. **EQTLGene**: the eQTL genes that the linked SNP affects. **EQTLGeneSymbol**: HGNC name of the linked genes. **LD(rsq): the LD r2. GWAS Cluster Size**: Number of GWAS SNPs in LD with TraitSNP. SNPs In **GWAS Cluster**: SNPs that are in LD with the TraitSNP.

Supplementary Table 11. List of traits used in Mendelian randomization and colocalization analysis.

Supplementary Table 12 eQTL SNPs which showed evidence of genetic colocalization with tested brain-related traits. ID, Chromosome, Position, SNP, Effect Allele, Non Effect Allele: Position of instrumenting SNP with effect allele used during the harmonization procedure. **Proxy used, Proxy SNP**: whether proxy lookup had to be performed to find SNP in outcome GWAS and the rsid of the proxy used. ***MetaBrain* SNP effects**: gene name and summary statistics for the instrument-exposure SNP association (*MetaBrain* eQTL). **Outcome SNP effects**: outcome name (neurological trait) and summary statistics for the harmonized instrument-outcome SNP association. **MR effects**: single SNP Wald ratio effect between the instrumented eQTL and neurological outcome. **Coloc results**: colocalization probability of both traits sharing the same causal variant in the region. **Decon-QTL results**: **eQTL SNP**: the SNP that was tested for cell type dependent effects. In some cases, a SNP which is in high LD with the instrument SNP is used for Decon-QTL. **LD R-squared**: the LD between SNP and eQTL SNP. Columns listing Decon-QTL results: **beta**: the beta of the interaction term in the Decon-QTL model with respect to the Effect Allele column. **FDR**: the Benjamini-Hochberg corrected interaction p-value. **Mendelian Disorders**: overlap of genes with Development Disorder Genotype - Phenotype Database (DDG2P) and OrphaNet.

Supplementary Table 13. Colocalization results for latest AD GWAS loci with *MetaBrain* Cortex-EUR primary eQTLs (**columns A to P** were adapted from Schwartzentruber *et al.* for comparisons and **columns Q to Y** are *MetaBrain* findings. **Category - 1**: previously identified

and replicated in *MetaBrain* Cortex-EUR, **2**: novel results found by *MetaBrain* Cortex-EUR, **3**: previously identified but not replicated in *MetaBrain* Cortex-EUR.

Supplementary Table 14. Mendelian Randomization comparison between *MetaBrain* and eQTLGen on multiple sclerosis outcome. (a) Wald ratio comparison on the same gene using different SNP instruments. For this analysis, the Wald ratio effects for the top hit eQTL for each gene within each study were compared. (b) Wald ratio comparison on the same gene fixing on the same eQTL instrument between studies. For this analysis, the eQTLGen Wald ratios were re-derived using the second Taylor expansion error term on the same SNP instruments as *MetaBrain*.

Supplementary Table 15. Colocalization of MR suggestive hits with high LD but allelic discordance. This table displays the colocalization results for 62 suggestive MR findings from Cortex-EUR with eQTL instruments replicated in eQTLGen ($p<0.05$) but allelic discordance (opposite directionalities of alleles). Highlighted rows are findings with colocalization in both Cortex-EUR and eQTLGen.

Supplementary Table 16. Comparison of MR suggestive hits for MS between *MetaBrain* and eQTLGen. This table displays 209 suggestive MR signals for multiple sclerosis in Cortex-EUR and the replication MR and colocalization results of corresponding genes in eQTLGen.

Supplementary Table 17. *Trans*-eQTL summary statistics. Sheet SheetOverview lists all *trans*-eQTL analyses represented by this Supplementary Table. **Percentage cross-mapping:** percentage of the gene that can be mapped within 5Mb of the *trans*-eQTL SNP.

Supplementary Table 18. Gene set enrichments for 7p21.3 *trans*-eQTL genes. Gene set enrichments calculated using g:Profiler. Sheet downregulated genes: gene set enrichments for genes that show downregulation due to the 7p21.3 *trans*-eQTL effect alleles. Sheet upregulated genes: gene set enrichments for genes that show upregulation due to the 7p21.3 *trans*-eQTL effect alleles.

Supplementary Table 19. Summary statistics for predicted cell-type proportions and SNP associations / AD interactions. Sheet cell fraction GWAS: associations ($FDR<0.05$) while limiting to Cortex-EUR+AFR samples. Sheet interaction summary stats: overview of cell-type proportion interactions with AD status for multiple set of *trans* SNPs. Sheet starting with 'no ENA': interaction result between AD status and *trans* SNPS. Each sheet is a different set of *trans*-eQTLs (e.g.: with or without AMP-AD, with or without PC correction). **SNP**: eQTL SNP. **N**: the sample size. **Columns ending with FDR**: the Benjamini-Hochberg interaction FDR per cell type.

Supplementary Table 20. Differences in predicted cell type proportions between included datasets. T-test p-values comparing cell type proportions for pairwise comparisons between the datasets included in the *trans*-eQTL analysis for SNP rs1990622. One sheet per cell type.

Supplementary Table 21. Gene-cell count correlations and 7p21.3 *trans*-eQTL Z-scores. *Trans*-eQTL Z-scores for three SNPs (rs11974335, rs10950398, and rs1990622), and Spearman correlations of the *trans*-eQTL genes (0 PCs removed) with predicted cell type proportions.

Supplementary Table 22. *Trans*-eQTL cell type deconvolution summary statistics (100 PCs). Gene: eQTL gene ensebl ID. **Gene symbol**: eQTL gene symbol. **SNP**: eQTL SNP. **Alleles**: SNP alleles. **Allele assessed**: the allele to which the betas are directed. **N**: the sample size. **HW pval**: the Hardy-Weinberg equilibrioum p-value. **Minor allele**: the minor allele. **MAF**: the minor allele frequency. **Overall z-score**: the meta-analysis eQTL z-score. **Columns ending with pvalue**: p-value for the cell-type interaction. **Columns ending with beta**: beta for the cell-type proportion term. **Columns ending with interaction beta**: beta for the genotype x cell-type interaction term. **Columns ending with BH-FDR**: the Benjamini-Hochberg FDR.

Supplementary Table 23. Replication of the *MetaBrain* cortex *trans*-ieQTLs in ROSMAP single-nucleus dataset. Sheet summary stats: overview of the *trans*-eQTL interactions with predicted cell type proportions and their replication in the ROSMAP single-nucleus dataset status for multiple set of *trans* SNPs. **Replication overlap**: number of eQTLs overlapping between *MetaBrain* bulk in ROSMAP single-nucleus. **Log beta pearson r / allelic concordance / Rb / pi1:** concordance metrics when filtering on bulk significant ieQTLs. Sheet starting with 'no ENA': interaction result between AD status and *trans* SNPS. Each sheet is a different set of *trans*-eQTLs (e.g.: with or without AMP-AD, with or without PC correction). **Gene**: eQTL gene ensembl ID. **Gene symbol**: eQTL gene symbol. **SNP**: eQTL SNP. **Alleles**: SNP alleles. **Allele assessed**: the allele to which the betas are directed. **Columns ending with pvalue**: p-value for the cell-type interaction or single-cell eQTL. **Columns ending with FDR**: the Benjamini-Hochberg FDR. For the replication datasets this FDR is calculated over the discovery significant (Benjamini-Hochberg FDR <0.05) rows of the respective cell type. **Columns ending with beta**: beta for the cell-type proportion term. **Columns ending with interaction beta**: beta for the genotype x cell-type interaction term.

Supplementary Table 24. Sheet summary stats: overview of the *trans*-eQTL interactions with AD status. Sheet starting with 'no ENA': interaction result between *trans*-eQTLs and AD status. Each sheet is a different set of *trans*-eQTLs (e.g., with or without PC correction). **SNP**: eQTL SNP. **Gene**: eQTL gene ensembl ID. **N**: the sample size. **Columns ending with FDR**: the Benjamini-Hochberg FDR for the interaction term. Each column is a different analysis: default includes all datasets, dataset corrected first performs a correcting for dataset dummy variables prior to mapping interactions, and only AMP-AD samples is self-explanatory.

Supplementary Table 25. Downstreamer results for amyotrophic lateral sclerosis in EUR and Asian ancestries. Sheet overview: lists set of ontologies tested for this phenotype. Sheet GenePrioritization_MetaBrain: gene prioritization performed in all *MetaBrain* samples. Sheet GenePrioritization_MetaBrainCortexOnly: gene prioritization performed in *MetaBrain* cortex samples. GenePrioritization_MetaBrainCerebellumOnly: gene prioritization performed in *MetaBrain* cerebellum samples. Sheets Reactome_MetaBrain, GO_BP_MetaBrain, GO_CC_MetaBrain, GO_MF_MetaBrain, KEGG_MetaBrain, and HPO_MetaBrain: gene set enrichments for coregulated genes identified using Downstreamer. Sheets

Expression_MetaBrain, Expression_HCA, and GtexV8_relative: expression enrichment using all MetaBrain samples, Human Cell Atlas, and GTEx v8.

Supplementary Table 26. Downstreamer results for Parkinson's disease. Sheet overview: lists set of ontologies tested for this phenotype. Sheet GenePrioritization_MetaBrain: gene prioritization performed in all *MetaBrain* samples. Sheet GenePrioritization_MetaBrainCortexOnly: gene prioritization performed in *MetaBrain* cortex samples. GenePrioritization_MetaBrainCerebellumOnly: gene prioritization performed in *MetaBrain* cerebellum samples. Sheets Reactome_MetaBrain, GO_BP_MetaBrain, GO_CC_MetaBrain, GO_MF_MetaBrain, KEGG_MetaBrain, and HPO_MetaBrain: gene set enrichments for coregulated genes identified using Downstreamer. Sheets Expression_MetaBrain, Expression_HCA, and GtexV8_relative: expression enrichment using all MetaBrain samples, Human Cell Atlas, and GTEx v8.

Supplementary Table 27. Downstreamer results for schizophrenia. Sheet overview: lists set of ontologies tested for this phenotype. Sheet GenePrioritization_MetaBrain: gene prioritization performed in all *MetaBrain* samples. Sheet GenePrioritization_MetaBrainCortexOnly: gene prioritization performed in *MetaBrain* cortex samples. GenePrioritization_MetaBrainCerebellumOnly: gene prioritization performed in *MetaBrain* cerebellum samples. Sheets Reactome_MetaBrain, GO_BP_MetaBrain, GO_CC_MetaBrain, GO_MF_MetaBrain, KEGG_MetaBrain, and HPO_MetaBrain: gene set enrichments for coregulated genes identified using Downstreamer. Sheets Expression_MetaBrain, Expression_HCA, and GtexV8_relative: expression enrichment using all MetaBrain samples, Human Cell Atlas, and GTEx v8.

Supplementary Table 28. Downstreamer results for Alzheimer's disease. Sheet overview: lists set of ontologies tested for this phenotype. Sheet GenePrioritization_MetaBrain: gene prioritization performed in all *MetaBrain* samples. Sheet GenePrioritization_MetaBrainCortexOnly: gene prioritization performed in *MetaBrain* cortex samples. GenePrioritization_MetaBrainCerebellumOnly: gene prioritization performed in *MetaBrain* cerebellum samples. Sheets Reactome_MetaBrain, GO_BP_MetaBrain, GO_CC_MetaBrain, GO_MF_MetaBrain, KEGG_MetaBrain, and HPO_MetaBrain: gene set enrichments for coregulated genes identified using Downstreamer. Sheets Expression_MetaBrain, Expression_HCA, and GtexV8_relative: expression enrichment using all MetaBrain samples, Human Cell Atlas, and GTEx v8.

Supplementary Table 29. Downstreamer results for multiple sclerosis. Sheet overview: lists set of ontologies tested for this phenotype. Sheet GenePrioritization_MetaBrain: gene prioritization performed in all *MetaBrain* samples. Sheet GenePrioritization_MetaBrainCortexOnly: gene prioritization performed in *MetaBrain* cortex samples. GenePrioritization_MetaBrainCerebellumOnly: gene prioritization performed in *MetaBrain* cerebellum samples. Sheets Reactome_MetaBrain, GO_BP_MetaBrain, GO_CC_MetaBrain, GO_MF_MetaBrain, KEGG_MetaBrain, and HPO_MetaBrain: gene set enrichments for coregulated genes identified using Downstreamer. Sheets Expression_MetaBrain, Expression_HCA, and GtexV8_relative: expression enrichment using all MetaBrain samples, Human Cell Atlas, and GTEx v8.

Supplementary Table 30. Downstreamer results for amyotrophic lateral sclerosis in EUR ancestry. Sheet overview: lists set of ontologies tested for this phenotype. Sheet GenePrioritization_MetaBrain: gene prioritization performed in all *MetaBrain* samples. Sheet GenePrioritization_MetaBrainCortexOnly: gene prioritization performed in *MetaBrain* cortex samples. GenePrioritization_MetaBrainCerebellumOnly: gene prioritization performed in *MetaBrain* cerebellum samples. Sheets Reactome_MetaBrain, GO_BP_MetaBrain, GO_CC_MetaBrain, GO_MF_MetaBrain, KEGG_MetaBrain, and HPO_MetaBrain: gene set enrichments for coregulated genes identified using Downstreamer. Sheets Expression_MetaBrain, Expression_HCA, and GtexV8_relative: expression enrichment using all MetaBrain samples, Human Cell Atlas, and GTEx v8.

Supplementary Table 31. ENA accession IDs. List of study accession IDs collected from European Nucleotide Archive. Columns are: **study_accession:** ID of the study in ENA. **run_accession:** ID of all the ENA runs included in this study (before QC).

References

1. Babraham Bioinformatics - FastQC A Quality Control tool for High Throughput Sequence Data. https://www.bioinformatics.babraham.ac.uk/projects/fastqc/.
2. Dobin, A. *et al.* STAR: ultrafast universal RNA-seq aligner. *Bioinformatics* **29**, 15–21 (2013).
3. Broad Institute. Picard Tools. (2019).
4. Tsui, B., Dow, M., Skola, D. & Carter, H. Extracting allelic read counts from 250,000 human sequencing runs in Sequence Read Archive. *bioRxiv* 386441 (2018) doi:10.1101/386441.
5. Bray, N. L., Pimentel, H., Melsted, P. & Pachter, L. Near-optimal probabilistic RNA-seq quantification. *Nat. Biotechnol.* **34**, 525–527 (2016).
6. Deelen, P. *et al.* Calling genotypes from public RNA-sequencing data enables identification of genetic variants that affect gene-expression levels. *Genome Medicine* **7**, 30 (2015).
7. McKenna, A. *et al.* The Genome Analysis Toolkit: A MapReduce framework for analyzing next-generation DNA sequencing data. *Genome Res.* **20**, 1297–1303 (2010).
8. Danecek, P. *et al.* The variant call format and VCFtools. *Bioinformatics* **27**, 2156–2158 (2011).
9. Castel, S. E., Levy-Moonshine, A., Mohammadi, P., Banks, E. & Lappalainen, T. Tools and best practices for data processing in allelic expression analysis. *Genome Biology* **16**, 195 (2015).
10. Ye, R., Cao, C. & Xue, Y. Enhancer RNA: biogenesis, function, and regulation. *Essays Biochem* **64**, 883–894 (2020).
11. Cingolani, P. *et al.* A program for annotating and predicting the effects of single nucleotide polymorphisms, SnpEff. *Fly* **6**, 80–92 (2012).
12. Deelen, P. *et al.* Genotype harmonizer: automatic strand alignment and format conversion for genotype data integration. *BMC Research Notes* **7**, 901 (2014).

13. Das, S. *et al.* Next-generation genotype imputation service and methods. *Nat Genet* **48**, 1284–1287 (2016).

14. Chang, C. C. *et al.* Second-generation PLINK: rising to the challenge of larger and richer datasets. *Gigascience* **4**, (2015).

15. Westra, H.-J. *et al.* MixupMapper: correcting sample mix-ups in genome-wide datasets increases power to detect small genetic effects. *Bioinformatics* **27**, 2104–2111 (2011).

16. Albert, F. W. & Kruglyak, L. The role of regulatory variation in complex traits and disease. *Nat Rev Genet* **16**, 197–212 (2015).

17. Võsa, U. *et al.* Large-scale cis- and trans-eQTL analyses identify thousands of genetic loci and polygenic scores that regulate blood gene expression. *Nat Genet* **53**, 1300–1310 (2021).

18. Roberts, T. C., Morris, K. V. & Wood, M. J. A. The role of long non-coding RNAs in neurodevelopment, brain function and neurological disease. *Philosophical Transactions of the Royal Society B: Biological Sciences* **369**, (2014).

19. Delaneau, O. *et al.* A complete tool set for molecular QTL discovery and analysis. *Nat Commun* **8**, 15452 (2017).

20. Storey, J. D. A direct approach to false discovery rates. *Journal of the Royal Statistical Society: Series B (Statistical Methodology)* **64**, 479–498 (2002).

21. Lyon, M. S. *et al.* The variant call format provides efficient and robust storage of GWAS summary statistics. *Genome Biology* **22**, 32 (2021).

22. Buniello, A. *et al.* The NHGRI-EBI GWAS Catalog of published genome-wide association studies, targeted arrays and summary statistics 2019. *Nucleic Acids Res* **47**, D1005–D1012 (2019).

23. Westra, H.-J. *et al.* Systematic identification of trans-eQTLs as putative drivers of known disease associations. *Nat Genet* **45**, 1238–1243 (2013).

24. Frankish, A. *et al.* GENCODE reference annotation for the human and mouse genomes. *Nucleic Acids Res* **47**, D766–D773 (2019).

25. Li, H. Aligning sequence reads, clone sequences and assembly contigs with BWA-MEM. *arXiv:1303.3997 [q-bio]* (2013).

26. Ongen, H., Buil, A., Brown, A. A., Dermitzakis, E. T. & Delaneau, O. Fast and efficient QTL mapper for thousands of molecular phenotypes. *Bioinformatics* **32**, 1479–1485 (2016).

27. Huang, Q. Q., Ritchie, S. C., Brozynska, M. & Inouye, M. Power, false discovery rate and Winner's Curse in eQTL studies. *Nucleic Acids Res* **46**, e133 (2018).

28. Ng, B. *et al.* An xQTL map integrates the genetic architecture of the human brain's transcriptome and epigenome. *Nat Neurosci* **20**, 1418–1426 (2017).

29. Sieberts, S. K. *et al.* Large eQTL meta-analysis reveals differing patterns between cerebral cortical and cerebellar brain regions. *Scientific Data* **7**, 340 (2020).

30. Allen, M. *et al.* Association of MAPT haplotypes with Alzheimer's disease risk and MAPT brain gene expression levels. *Alzheimer's Research & Therapy* **6**, 39 (2014).

31. Dobbyn, A. *et al.* Landscape of Conditional eQTL in Dorsolateral Prefrontal Cortex and Co-localization with Schizophrenia GWAS. *Am J Hum Genet* **102**, 1169–1184 (2018).

32. Raudvere, U. *et al.* g:Profiler: a web server for functional enrichment analysis and conversions of gene lists (2019 update). *Nucleic Acids Research* **47**, W191–W198 (2019).

33. The Gene Ontology Resource: 20 years and still GOing strong. *Nucleic Acids Res* **47**, D330–D338 (2019).

34. Kanehisa, M. & Goto, S. KEGG: kyoto encyclopedia of genes and genomes. *Nucleic Acids Res.* **28**, 27–30 (2000).

35. Jassal, B. *et al.* The reactome pathway knowledgebase. *Nucleic Acids Res.* **48**, D498–D503 (2020).

36. Slenter, D. N. *et al.* WikiPathways: a multifaceted pathway database bridging metabolomics to other omics research. *Nucleic Acids Research* **46**, D661–D667 (2018).

37. Wingender, E., Dietze, P., Karas, H. & Knüppel, R. TRANSFAC: a database on transcription factors and their DNA binding sites. *Nucleic Acids Res* **24**, 238–241 (1996).

38. Huang, H.-Y. *et al.* miRTarBase 2020: updates to the experimentally validated microRNA–target interaction database. *Nucleic Acids Research* **48**, D148–D154 (2020).

39. Pontén, F., Jirström, K. & Uhlen, M. The Human Protein Atlas—a tool for pathology. *The Journal of Pathology* **216**, 387–393 (2008).

40. Köhler, S. *et al.* Expansion of the Human Phenotype Ontology (HPO) knowledge base and resources. *Nucleic Acids Res.* **47**, D1018–D1027 (2019).

41. Mohammadi, P., Castel, S. E., Brown, A. A. & Lappalainen, T. Quantifying the regulatory effect size of cis-acting genetic variation using allelic fold change. *Genome Res* **27**, 1872–1884 (2017).

42. Holland, D. *et al.* Estimating Effect Sizes and Expected Replication Probabilities from GWAS Summary Statistics. *Frontiers in Genetics* **7**, (2016).

43. Qi, T. *et al.* Identifying gene targets for brain-related traits using transcriptomic and methylomic data from blood. *Nat Commun* **9**, (2018).

44. Genetic effects on gene expression across human tissues. *Nature* **550**, 204–213 (2017).

45. Shang, L. *et al.* Genetic Architecture of Gene Expression in European and African Americans: An eQTL Mapping Study in GENOA. *The American Journal of Human Genetics* **106**, 496–512 (2020).

46. Stelzer, G. *et al.* The GeneCards Suite: From Gene Data Mining to Disease Genome Sequence Analyses. *Current Protocols in Bioinformatics* **54**, 1.30.1-1.30.33 (2016).

47. GTEx Consortium. The GTEx Consortium atlas of genetic regulatory effects across human tissues. *Science* **369**, 1318–1330 (2020).

48. Fu, J. *et al.* Unraveling the Regulatory Mechanisms Underlying Tissue-Dependent Genetic Variation of Gene Expression. *PLOS Genetics* **8**, e1002431 (2012).

49. Wang, D. *et al.* Comprehensive functional genomic resource and integrative model for the human brain. *Science* **362**, (2018).

50. Lawson, C. L. & Hanson, R. J. *Solving Least Squares Problems*. (Society for Industrial and Applied Mathematics, 1995). doi:10.1137/1.9781611971217.

51. Virtanen, P. *et al.* SciPy 1.0: fundamental algorithms for scientific computing in Python. *Nature Methods* **17**, 261–272 (2020).

52. Patrick, E. *et al.* Deconvolving the contributions of cell-type heterogeneity on cortical gene expression. *PLOS Computational Biology* **16**, e1008120 (2020).

53. Herculano-Houzel, S. The human brain in numbers: a linearly scaled-up primate brain. *Front. Hum. Neurosci.* **3**, (2009).

54. von Bartheld, C. S., Bahney, J. & Herculano-Houzel, S. The Search for True Numbers of Neurons and Glial Cells in the Human Brain: A Review of 150 Years of Cell Counting. *J Comp Neurol* **524**, 3865–3895 (2016).

55. Aguirre-Gamboa, R. *et al.* Deconvolution of bulk blood eQTL effects into immune cell subpopulations. *BMC Bioinformatics* **21**, 243 (2020).

56. Bryois, J. *et al.* Cell-type-specific cis-eQTLs in eight human brain cell types identify novel risk genes for psychiatric and neurological disorders. *Nat Neurosci* **25**, 1104–1112 (2022).

57. Zhu, Z. *et al.* Integration of summary data from GWAS and eQTL studies predicts complex trait gene targets. *Nat Genet* **48**, 481–487 (2016).

58. Mathys, H. *et al.* Single-cell transcriptomic analysis of Alzheimer's disease. *Nature* **570**, 332–337 (2019).

59. Stuart, T. *et al.* Comprehensive Integration of Single-Cell Data. *Cell* **177**, 1888-1902.e21 (2019).

60. Hafemeister, C. & Satija, R. Normalization and variance stabilization of single-cell RNA-seq data using regularized negative binomial regression. *Genome Biology* **20**, 296 (2019).

61. McInnes, L., Healy, J. & Melville, J. UMAP: Uniform Manifold Approximation and Projection for Dimension Reduction. *arXiv:1802.03426 [cs, stat]* (2020).

62. Mathys, H. *et al.* Single-cell transcriptomic analysis of Alzheimer's disease. *Nature* **570**, 332–337 (2019).

63. Gazal, S. *et al.* Combining SNP-to-gene linking strategies to identify disease genes and assess disease omnigenicity. *Nat Genet* 1–10 (2022) doi:10.1038/s41588-022-01087-y.

64. Limited overlap of eQTLs and GWAS hits due to systematic differences in discovery | bioRxiv. https://www.biorxiv.org/content/10.1101/2022.05.07.491045v1.

65. gene2phenotype. https://www.ebi.ac.uk/gene2phenotype/downloads.

66. Plenge, R. M. Priority index for human genetics and drug discovery. *Nat Genet* **51**, 1073–1075 (2019).

67. Hanauer, M. Orphanet/Orphadata_aggregated. (2021).

68. Blech, M. martinblech/xmltodict. (2021).

69. McKinney, W. Data Structures for Statistical Computing in Python. *Proceedings of the 9th Python in Science Conference* 56–61 (2010) doi:10.25080/Majora-92bf1922-00a.

70. Schwartzentruber, J. *et al.* Genome-wide meta-analysis, fine-mapping and integrative prioritization implicate new Alzheimer's disease risk genes. *Nature Genetics* 1–11 (2021) doi:10.1038/s41588-020-00776-w.

71. Brouwers, N. *et al.* Alzheimer risk associated with a copy number variation in the complement receptor 1 increasing C3b/C4b binding sites. *Molecular psychiatry* **17**, 223–33 (2012).

72. Kucukkilic, E. *et al.* Complement receptor 1 gene (CR1) intragenic duplication and risk of Alzheimer's disease. *Human genetics* **137**, 305–314 (2018).

73. Dunkelberger, J. R. & Song, W. C. Complement and its role in innate and adaptive immune responses. *Cell research* **20**, 34–50 (2010).

74. Maier, M. *et al.* Complement C3 deficiency leads to accelerated amyloid beta plaque deposition and neurodegeneration and modulation of the microglia/macrophage phenotype in amyloid precursor protein transgenic mice. *The Journal of neuroscience : the official journal of the Society for Neuroscience* **28**, 6333–41 (2008).

75. Matthews, A. L. *et al.* Regulation of Leukocytes by TspanC8 Tetraspanins and the 'Molecular Scissor' ADAM10. *Frontiers in immunology* **9**, 1451 (2018).

76. Jouannet, S. *et al.* TspanC8 tetraspanins differentially regulate the cleavage of ADAM10 substrates, Notch activation and ADAM10 membrane compartmentalization. *Cellular and molecular life sciences : CMLS* **73**, 1895–915 (2016).

77. Suh, J. *et al.* ADAM10 missense mutations potentiate β-amyloid accumulation by impairing prodomain chaperone function. *Neuron* **80**, 385–401 (2013).

78. Ulland, T. K. & Colonna, M. TREM2 - a key player in microglial biology and Alzheimer disease. *Nature reviews. Neurology* **14**, 667–675 (2018).

79. Li, Q. *et al.* Developmental Heterogeneity of Microglia and Brain Myeloid Cells Revealed by Deep Single-Cell RNA Sequencing. *Neuron* **101**, 207-223.e10 (2019).

80. Schlepckow, K. *et al.* An Alzheimer-associated TREM2 variant occurs at the ADAM cleavage site and affects shedding and phagocytic function. *EMBO molecular medicine* **9**, 1356–1365 (2017).

81. Thornton, P. *et al.* TREM2 shedding by cleavage at the H157-S158 bond is accelerated for the Alzheimer's disease-associated H157Y variant. *EMBO Mol Med* **9**, 1366–1378 (2017).

82. Bernstein, K. E. *et al.* A modern understanding of the traditional and nontraditional biological functions of angiotensin-converting enzyme. *Pharmacological reviews* **65**, 1–46 (2013).

83. Zou, K. *et al.* Angiotensin-converting enzyme converts amyloid beta-protein 1-42 (Abeta(1-42)) to Abeta(1-40), and its inhibition enhances brain Abeta deposition. *The Journal of neuroscience : the official journal of the Society for Neuroscience* **27**, 8628–35 (2007).

84. Liu, S. *et al.* A clinical dose of angiotensin-converting enzyme (ACE) inhibitor and heterozygous ACE deletion exacerbate Alzheimer's disease pathology in mice. *The Journal of biological chemistry* **294**, 9760–9770 (2019).

85. Quitterer, U. & AbdAlla, S. Improvements of symptoms of Alzheimer`s disease by inhibition of the angiotensin system. *Pharmacological research* **154**, 104230 (2020).

86. Ding, J. *et al.* Antihypertensive medications and risk for incident dementia and Alzheimer's disease: a meta-analysis of individual participant data from prospective cohort studies. *The Lancet. Neurology* **19**, 61–70 (2020).

87. Eckman, E. A. *et al.* Regulation of steady-state beta-amyloid levels in the brain by neprilysin and endothelin-converting enzyme but not angiotensin-converting enzyme. *The Journal of biological chemistry* **281**, 30471–8 (2006).

88. Hemming, M. L., Selkoe, D. J. & Farris, W. Effects of prolonged angiotensin-converting enzyme inhibitor treatment on amyloid beta-protein metabolism in mouse models of Alzheimer disease. *Neurobiology of disease* **26**, 273–81 (2007).

89. Cuddy, L. K. *et al.* Aβ-accelerated neurodegeneration caused by Alzheimer's-associated ACE variant R1279Q is rescued by angiotensin system inhibition in mice. *Science translational medicine* **12**, (2020).

90. Serneels, L. *et al.* gamma-Secretase heterogeneity in the Aph1 subunit: relevance for Alzheimer's disease. *Science (New York, N.Y.)* **324**, 639–42 (2009).

91. Zhang, X. *et al.* Negative evidence for a role of APH1B T27I variant in Alzheimer's disease. *Human molecular genetics* **29**, 955–966 (2020).

92. Frederickson, C. J., Suh, S. W., Silva, D., Frederickson, C. J. & Thompson, R. B. Importance of zinc in the central nervous system: the zinc-containing neuron. *J Nutr* **130**, 1471S–83S (2000).

93. Esch, F. S. *et al.* Cleavage of amyloid beta peptide during constitutive processing of its precursor. *Science* **248**, 1122–1124 (1990).

94. Bush, A. I. *et al.* A novel zinc(II) binding site modulates the function of the beta A4 amyloid protein precursor of Alzheimer's disease. *J Biol Chem* **268**, 16109–16112 (1993).

95. Lovell, M. A. A potential role for alterations of zinc and zinc transport proteins in the progression of Alzheimer's disease. *J Alzheimers Dis* **16**, 471–483 (2009).
96. Religa, D. *et al.* Elevated cortical zinc in Alzheimer disease. *Neurology* **67**, 69–75 (2006).
97. Panayi, A. E., Spyrou, N. M., Iversen, B. S., White, M. A. & Part, P. Determination of cadmium and zinc in Alzheimer's brain tissue using inductively coupled plasma mass spectrometry. *J Neurol Sci* **195**, 1–10 (2002).
98. Lyubartseva, G. & Lovell, M. A. A potential role for zinc alterations in the pathogenesis of Alzheimer's disease. *Biofactors* **38**, 98–106 (2012).
99. Peng, Q. *et al.* TREM2- and DAP12-dependent activation of PI3K requires DAP10 and is inhibited by SHIP1. *Sci Signal* **3**, ra38 (2010).
100. Pedicone, C. *et al.* Pan-SHIP1/2 inhibitors promote microglia effector functions essential for CNS homeostasis. *J Cell Sci* **133**, jcs238030 (2020).
101. Tsai, A. P. *et al.* INPP5D expression is associated with risk for Alzheimer's disease and induced by plaque-associated microglia. *Neurobiol Dis* **153**, 105303 (2021).
102. Demontis, D. *et al.* Discovery of the first genome-wide significant risk loci for attention deficit/hyperactivity disorder. *Nat Genet* **51**, 63–75 (2019).
103. van Rheenen, W. *et al.* Common and rare variant association analyses in amyotrophic lateral sclerosis identify 15 risk loci with distinct genetic architectures and neuron-specific biology. *Nat Genet* **53**, 1636–1648 (2021).
104. Yamamoto, Y. *et al.* Cloning and expression of myelin-associated oligodendrocytic basic protein. A novel basic protein constituting the central nervous system myelin. *J Biol Chem* **269**, 31725–31730 (1994).

105. Demircioglu, F. E., Burkhardt, P. & Fasshauer, D. The SM protein Sly1 accelerates assembly of the ER-Golgi SNARE complex. *Proc Natl Acad Sci U S A* **111**, 13828–33 (2014).
106. Burgoyne, R. D. & Morgan, A. Chaperoning the SNAREs: a role in preventing neurodegeneration? *Nat Cell Biol* **13**, 8–9 (2011).
107. Brooks, W. S., Banerjee, S. & Crawford, D. F. G2E3 is a nucleo-cytoplasmic shuttling protein with DNA damage responsive localization. *Exp Cell Res* **313**, 665–76 (2007).
108. Grove, J. *et al.* Identification of common genetic risk variants for autism spectrum disorder. *Nat Genet* **51**, 431–444 (2019).
109. Ohmae, S. *et al.* Molecular identification and characterization of a family of kinases with homology to Ca2+/calmodulin-dependent protein kinases I/IV. *J Biol Chem* **281**, 20427–20439 (2006).
110. Rafiullah, R. *et al.* Homozygous missense mutation in the LMAN2L gene segregates with intellectual disability in a large consanguineous Pakistani family. *Journal of Medical Genetics* **53**, 138–144 (2016).
111. Lim, C. H. *et al.* Genetic association of LMAN2L gene in schizophrenia and bipolar disorder and its interaction with ANK3 gene polymorphism. *Prog Neuropsychopharmacol Biol Psychiatry* **54**, 157–162 (2014).
112. Tsai, R. Y. L. & McKay, R. D. G. A nucleolar mechanism controlling cell proliferation in stem cells and cancer cells. *Genes Dev* **16**, 2991–3003 (2002).
113. Goes, F. S. *et al.* Genome-wide association of mood-incongruent psychotic bipolar disorder. *Transl Psychiatry* **2**, e180 (2012).

114. Styrkarsdottir, U. *et al.* Meta-analysis of Icelandic and UK data sets identifies missense variants in SMO, IL11, COL11A1 and 13 more new loci associated with osteoarthritis. *Nat Genet* **50**, 1681–1687 (2018).

115. Southam, L. *et al.* Whole genome sequencing and imputation in isolated populations identify genetic associations with medically-relevant complex traits. *Nat Commun* **8**, 15606 (2017).

116. Kettunen, J. *et al.* Genome-wide study for circulating metabolites identifies 62 loci and reveals novel systemic effects of LPA. *Nat Commun* **7**, 11122 (2016).

117. International League Against Epilepsy Consortium on Complex, E. Genome-wide mega-analysis identifies 16 loci and highlights diverse biological mechanisms in the common epilepsies. *Nat Commun* **9**, 5269 (2018).

118. Schwarz, M. *et al.* The bile acid synthetic gene 3beta-hydroxy-Delta(5)-C(27)-steroid oxidoreductase is mutated in progressive intrahepatic cholestasis. *J Clin Invest* **106**, 1175–84 (2000).

119. Ferrari, R. *et al.* Frontotemporal dementia and its subtypes: a genome-wide association study. *Lancet Neurol* **13**, 686–99 (2014).

120. Nguyen, T., Liu, X. K., Zhang, Y. & Dong, C. BTNL2, a butyrophilin-like molecule that functions to inhibit T cell activation. *J Immunol* **176**, 7354–60 (2006).

121. Valentonyte, R. *et al.* Sarcoidosis is associated with a truncating splice site mutation in BTNL2. *Nat Genet* **37**, 357–64 (2005).

122. Fortes, G. C. C. *et al.* Rapidly progressive dementia due to neurosarcoidosis. *Dement Neuropsychol* **7**, 428–434 (2013).

123. Van Deerlin, V. M. *et al.* Common variants at 7p21 are associated with frontotemporal lobar degeneration with TDP-43 inclusions. *Nat Genet* **42**, 234–9 (2010).

124. Howard, D. M. *et al.* Genome-wide association study of depression phenotypes in UK Biobank identifies variants in excitatory synaptic pathways. *Nat Commun* **9**, 1470 (2018).

125. Jansen, P. R. *et al.* Genome-wide analysis of insomnia in 1,331,010 individuals identifies new risk loci and functional pathways. *Nat Genet* **51**, 394–403 (2019).

126. Nagel, M., Watanabe, K., Stringer, S., Posthuma, D. & van der Sluis, S. Item-level analyses reveal genetic heterogeneity in neuroticism. *Nat Commun* **9**, 905 (2018).

127. Goes, F. S. *et al.* Genome-wide association study of schizophrenia in Ashkenazi Jews. *Am J Med Genet B Neuropsychiatr Genet* **168**, 649–659 (2015).

128. Sherva, R. *et al.* Genome-wide association study of rate of cognitive decline in Alzheimer's disease patients identifies novel genes and pathways. *Alzheimers Dement* **16**, 1134–1145 (2020).

129. Savage, J. E. *et al.* Genome-wide association meta-analysis in 269,867 individuals identifies new genetic and functional links to intelligence. *Nat Genet* **50**, 912–919 (2018).

130. Davies, G. *et al.* Study of 300,486 individuals identifies 148 independent genetic loci influencing general cognitive function. *Nat Commun* **9**, 2098 (2018).

131. Kunkle, B. W. *et al.* Genetic meta-analysis of diagnosed Alzheimer's disease identifies new risk loci and implicates Aβ, tau, immunity and lipid processing. *Nature genetics* **51**, 414–430 (2019).

132. Zhu, Z. *et al.* Shared genetic and experimental links between obesity-related traits and asthma subtypes in UK Biobank. *J Allergy Clin Immunol* **145**, 537–549 (2020).

133. Hom, G. *et al.* Association of systemic lupus erythematosus with C8orf13-BLK and ITGAM-ITGAX. *N Engl J Med* **358**, 900–909 (2008).

134. Mägi, R. *et al.* Contribution of 32 GWAS-identified common variants to severe obesity in European adults referred for bariatric surgery. *PLoS One* **8**, e70735 (2013).

135. Baselmans, B. M. L. *et al.* Multivariate genome-wide analyses of the well-being spectrum. *Nat Genet* **51**, 445–451 (2019).

136. Jones, S. E. *et al.* Genome-wide association analyses of chronotype in 697,828 individuals provides insights into circadian rhythms. *Nat Commun* **10**, 343 (2019).

137. Puskarjov, M. *et al.* A variant of KCC2 from patients with febrile seizures impairs neuronal Cl- extrusion and dendritic spine formation. *EMBO reports* **15**, 723–9 (2014).

138. Stödberg, T. *et al.* Mutations in SLC12A5 in epilepsy of infancy with migrating focal seizures. *Nature communications* **6**, 8038 (2015).

139. Zhang, Y. *et al.* Purification and Characterization of Progenitor and Mature Human Astrocytes Reveals Transcriptional and Functional Differences with Mouse. *Neuron* **89**, 37–53 (2016).

140. International Multiple Sclerosis Genetics Consortium. Multiple sclerosis genomic map implicates peripheral immune cells and microglia in susceptibility. *Science* **365**, (2019).

141. Jones, G., Prosser, D. E. & Kaufmann, M. 25-Hydroxyvitamin D-24-hydroxylase (CYP24A1): its important role in the degradation of vitamin D. *Archives of biochemistry and biophysics* **523**, 9–18 (2012).

142. Schlingmann, K. P. *et al.* Mutations in CYP24A1 and idiopathic infantile hypercalcemia. *N Engl J Med* **365**, 410–21 (2011).

143. Cappellani, D. *et al.* Hereditary Hypercalcemia Caused by a Homozygous Pathogenic Variant in the CYP24A1 Gene: A Case Report and Review of the Literature. *Case reports in endocrinology* **2019**, 4982621 (2019).

144. Mpandzou, G., Aït Ben Haddou, E., Regragui, W., Benomar, A. & Yahyaoui, M. Vitamin D deficiency and its role in neurological conditions: A review. *Rev Neurol (Paris)* **172**, 109–122 (2016).

145. Bishop, G. A., Stunz, L. L. & Hostager, B. S. TRAF3 as a Multifaceted Regulator of B Lymphocyte Survival and Activation. *Frontiers in immunology* **9**, 2161 (2018).

146. Xie, P., Hostager, B. S. & Bishop, G. A. Requirement for TRAF3 in signaling by LMP1 but not CD40 in B lymphocytes. *The Journal of experimental medicine* **199**, 661–71 (2004).

147. Hussein, H. A. M. & Akula, S. M. miRNA-36 inhibits KSHV, EBV, HSV-2 infection of cells via stifling expression of interferon induced transmembrane protein 1 (IFITM1). *Sci Rep* **7**, 17972 (2017).

148. Roostaei, T. *et al.* Proximal and distal effects of genetic susceptibility to multiple sclerosis on the T cell epigenome. *Nat Commun* **12**, 7078 (2021).

149. Dalla Rosa, I. *et al.* MPV17L2 is required for ribosome assembly in mitochondria. *Nucleic acids research* **42**, 8500–15 (2014).

150. Campbell, G. & Mahad, D. J. Mitochondrial dysfunction and axon degeneration in progressive multiple sclerosis. *FEBS Lett* **592**, 1113–1121 (2018).

151. Barcelos, I. P. de, Troxell, R. M. & Graves, J. S. Mitochondrial Dysfunction and Multiple Sclerosis. *Biology (Basel)* **8**, (2019).

152. Lanata, C. M. *et al.* Genetic contributions to lupus nephritis in a multi-ethnic cohort of systemic lupus erythematous patients. *PloS one* **13**, e0199003 (2018).

153. Ross, K. A. Coherent somatic mutation in autoimmune disease. *PloS one* **9**, e101093 (2014).

154. Sharma, A. *et al.* Identification of non-HLA genes associated with development of islet autoimmunity and type 1 diabetes in the prospective TEDDY cohort. *Journal of autoimmunity* **89**, 90–100 (2018).

155. Malecki, J. *et al.* The novel lysine specific methyltransferase METTL21B affects mRNA translation through inducible and dynamic methylation of Lys-165 in human eukaryotic elongation factor 1 alpha (eEF1A). *Nucleic acids research* **45**, 4370–4389 (2017).

156. Hamey, J. J., Wienert, B., Quinlan, K. G. R. & Wilkins, M. R. METTL21B Is a Novel Human Lysine Methyltransferase of Translation Elongation Factor 1A: Discovery by CRISPR/Cas9 Knockout. *Molecular & cellular proteomics : MCP* **16**, 2229–2242 (2017).

157. Knight, J. R. P. *et al.* Control of translation elongation in health and disease. *Disease models & mechanisms* **13**, (2020).

158. Talapatra, S., Wagner, J. D. & Thompson, C. B. Elongation factor-1 alpha is a selective regulator of growth factor withdrawal and ER stress-induced apoptosis. *Cell death and differentiation* **9**, 856–61 (2002).

159. Chalorak, P., Dharmasaroja, P. & Meemon, K. Downregulation of eEF1A/EFT3-4 Enhances Dopaminergic Neurodegeneration After 6-OHDA Exposure in C. elegans Model. *Frontiers in neuroscience* **14**, 303 (2020).

160. Prommahom, A. & Dharmasaroja, P. Effects of eEF1A2 knockdown on autophagy in an MPP(+)-induced cellular model of Parkinson's disease. *Neuroscience research* (2020) doi:10.1016/j.neures.2020.03.013.

161. Garcia-Esparcia, P. *et al.* Altered machinery of protein synthesis is region- and stage-dependent and is associated with α-synuclein oligomers in Parkinson's disease. *Acta neuropathologica communications* **3**, 76 (2015).

162. Cao, S. *et al.* Homozygous EEF1A2 mutation causes dilated cardiomyopathy, failure to thrive, global developmental delay, epilepsy and early death. *Human molecular genetics* **26**, 3545–3552 (2017).

163. Lam, W. W. *et al.* Novel de novo EEF1A2 missense mutations causing epilepsy and intellectual disability. *Molecular genetics & genomic medicine* **4**, 465–74 (2016).

164. Nakajima, J. *et al.* De novo EEF1A2 mutations in patients with characteristic facial features, intellectual disability, autistic behaviors and epilepsy. *Clinical genetics* **87**, 356–61 (2015).

165. Beckelman, B. C. *et al.* Dysregulation of Elongation Factor 1A Expression is Correlated with Synaptic Plasticity Impairments in Alzheimer's Disease. *Journal of Alzheimer's disease : JAD* **54**, 669–78 (2016).

166. Beckelman, B. C., Zhou, X., Keene, C. D. & Ma, T. Impaired Eukaryotic Elongation Factor 1A Expression in Alzheimer's Disease. *Neuro-degenerative diseases* **16**, 39–43 (2016).

167. Ahola, S. *et al.* Mitochondrial EFTs defects in juvenile-onset Leigh disease, ataxia, neuropathy, and optic atrophy. *Neurology* **83**, 743–51 (2014).

168. Emperador, S. *et al.* Molecular-genetic characterization and rescue of a TSFM mutation causing childhood-onset ataxia and nonobstructive cardiomyopathy. *European journal of human genetics : EJHG* **25**, 153–156 (2016).

169. Smeitink, J. A. *et al.* Distinct clinical phenotypes associated with a mutation in the mitochondrial translation elongation factor EFTs. *American journal of human genetics* **79**, 869–77 (2006).

170. Wang, J. *et al.* TSPAN31 is a critical regulator on transduction of survival and apoptotic signals in hepatocellular carcinoma cells. *FEBS letters* **591**, 2905–2918 (2017).

171. Gao, X., Leone, G. W. & Wang, H. Cyclin D-CDK4/6 functions in cancer. *Advances in cancer research* **148**, 147–169 (2020).

172. Deng, J. *et al.* CDK4/6 Inhibition Augments Antitumor Immunity by Enhancing T-cell Activation. *Cancer discovery* **8**, 216–233 (2018).

173. Goel, S. *et al.* CDK4/6 inhibition triggers anti-tumour immunity. *Nature* **548**, 471–475 (2017).

174. Sekine, C. *et al.* Successful treatment of animal models of rheumatoid arthritis with small-molecule cyclin-dependent kinase inhibitors. *Journal of immunology (Baltimore, Md. : 1950)* **180**, 1954–61 (2008).

175. Safavi, A. & Hersh, L. B. Degradation of dynorphin-related peptides by the puromycin-sensitive aminopeptidase and aminopeptidase M. *Journal of neurochemistry* **65**, 389–95 (1995).

176. Kudo, L. C. *et al.* Puromycin-sensitive aminopeptidase (PSA/NPEPPS) impedes development of neuropathology in hPSA/TAU(P301L) double-transgenic mice. *Human molecular genetics* **20**, 1820–33 (2011).

177. Karsten, S. L. *et al.* A genomic screen for modifiers of tauopathy identifies puromycin-sensitive aminopeptidase as an inhibitor of tau-induced neurodegeneration. *Neuron* **51**, 549–60 (2006).

178. Ren, G. *et al.* Cu, Zn-superoxide dismutase 1 (SOD1) is a novel target of Puromycin-sensitive aminopeptidase (PSA/NPEPPS): PSA/NPEPPS is a possible modifier of amyotrophic lateral sclerosis. *Molecular neurodegeneration* **6**, 29 (2011).

179. Lin, Y.-H. *et al.* Identification of ten novel genes involved in human spermatogenesis by microarray analysis of testicular tissue. *Fertil Steril* **86**, 1650–1658 (2006).

180. Giordana, M. T., Richiardi, P., Trevisan, E., Boghi, A. & Palmucci, L. Abnormal ubiquitination of axons in normally myelinated white matter in multiple sclerosis brain. *Neuropathol Appl Neurobiol* **28**, 35–41 (2002).

181. O'Connor, A. M., Crawley, A. M. & Angel, J. B. Interleukin-7 enhances memory CD8(+) T-cell recall responses in health but its activity is impaired in human immunodeficiency virus infection. *Immunology* **131**, 525–536 (2010).

182. Kreft, K. L. *et al.* Decreased systemic IL-7 and soluble IL-7Rα in multiple sclerosis patients. *Genes Immun* **13**, 587–592 (2012).

183. Papadopoulou, L. C. *et al.* Fatal infantile cardioencephalomyopathy with COX deficiency and mutations in SCO2, a COX assembly gene. *Nat Genet* **23**, 333–337 (1999).

184. Tran-Viet, K.-N. *et al.* Mutations in SCO2 are associated with autosomal-dominant high-grade myopia. *Am J Hum Genet* **92**, 820–826 (2013).

185. Alliel, P. M. *et al.* Myoneurin, a novel member of the BTB/POZ-zinc finger family highly expressed in human muscle. *Biochem Biophys Res Commun* **273**, 385–391 (2000).

186. Miyadera, K. *et al.* Role of thymidine phosphorylase activity in the angiogenic effect of platelet derived endothelial cell growth factor/thymidine phosphorylase. *Cancer Res* **55**, 1687–1690 (1995).

187. Chapouly, C. *et al.* Astrocytic TYMP and VEGFA drive blood-brain barrier opening in inflammatory central nervous system lesions. *Brain* **138**, 1548–1567 (2015).

188. Lee, J.-K. & Bou Dagher, J. Regulator of G-protein Signaling (RGS)1 and RGS10 Proteins as Potential Drug Targets for Neuroinflammatory and Neurodegenerative Diseases. *AAPS J* **18**, 545–549 (2016).

189. Moratz, C. *et al.* Regulator of G protein signaling 1 (RGS1) markedly impairs Gi alpha signaling responses of B lymphocytes. *J Immunol* **164**, 1829–1838 (2000).

190. Han, J.-I., Huang, N.-N., Kim, D.-U. & Kehrl, J. H. RGS1 and RGS13 mRNA silencing in a human B lymphoma line enhances responsiveness to chemoattractants and impairs desensitization. *J Leukoc Biol* **79**, 1357–1368 (2006).

191. Xu, Q. *et al.* MAST3 modulates the inflammatory response and proliferation of fibroblast-like synoviocytes in rheumatoid arthritis. *Int Immunopharmacol* **77**, 105900 (2019).

192. Wang, Y. *et al.* MiR-125a-3p inhibits cell proliferation and inflammation responses in fibroblast-like synovial cells in rheumatoid arthritis by mediating the Wnt/β-catenin and NF-κB pathways via targeting MAST3. *J Musculoskelet Neuronal Interact* **21**, 560–567 (2021).

193. Labbé, C. *et al.* MAST3: a novel IBD risk factor that modulates TLR4 signaling. *Genes Immun* **9**, 602–612 (2008).

194. Labbé, C. *et al.* Genome-wide expression profiling implicates a MAST3-regulated gene set in colonic mucosal inflammation of ulcerative colitis patients. *Inflamm Bowel Dis* **18**, 1072–1080 (2012).

195. Spinelli, E. *et al.* Pathogenic MAST3 Variants in the STK Domain Are Associated with Epilepsy. *Ann Neurol* **90**, 274–284 (2021).

196. Meng, F. *et al.* PPM1F in Dentate Gyrus Modulates Anxiety-Related Behaviors by Regulating BDNF Expression via AKT/JNK/p-H3S10 Pathway. *Mol Neurobiol* **58**, 3529–3544 (2021).

197. Liu, J. *et al.* PPM1F in hippocampal dentate gyrus regulates the depression-related behaviors by modulating neuronal excitability. *Exp Neurol* **340**, 113657 (2021).

198. Sullivan, D. R. *et al.* The PPM1F gene moderates the association between PTSD and cortical thickness. *J Affect Disord* **259**, 201–209 (2019).

199. Wingo, A. P. *et al.* Expression of the PPM1F Gene Is Regulated by Stress and Associated With Anxiety and Depression. *Biol Psychiatry* **83**, 284–295 (2018).

200. Chuang, Y.-C., Lee, C.-H., Sun, W.-H. & Chen, C.-C. Involvement of advillin in somatosensory neuron subtype-specific axon regeneration and neuropathic pain. *Proc Natl Acad Sci U S A* **115**, E8557–E8566 (2018).

201. Xie, Z. & Li, H. The discovery of AVIL as a bona fide oncogene in glioblastoma. *Mol Cell Oncol* **7**, 1804309 (2020).

202. Xie, Z. *et al.* A cytoskeleton regulator AVIL drives tumorigenesis in glioblastoma. *Nat Commun* **11**, 3457 (2020).

203. Pouget, J. G. *et al.* Cross-disorder analysis of schizophrenia and 19 immune-mediated diseases identifies shared genetic risk. *Hum Mol Genet* **28**, 3498–3513 (2019).

204. Shin, J.-H. *et al.* PARIS (ZNF746) repression of PGC-1α contributes to neurodegeneration in Parkinson's disease. *Cell* **144**, 689–702 (2011).

205. Stevens, D. A. *et al.* Parkin loss leads to PARIS-dependent declines in mitochondrial mass and respiration. *Proc Natl Acad Sci U S A* **112**, 11696–11701 (2015).

206. Koolen, D. A. *et al.* Mutations in the chromatin modifier gene KANSL1 cause the 17q21.31 microdeletion syndrome. *Nat Genet* **44**, 639–641 (2012).

207. Linda, K. *et al.* Imbalanced autophagy causes synaptic deficits in a human model for neurodevelopmental disorders. *Autophagy* 1–20 (2021) doi:10.1080/15548627.2021.1936777.

208. Purlyte, E. *et al.* Rab29 activation of the Parkinson's disease-associated LRRK2 kinase. *EMBO J* **37**, 1–18 (2018).

209. Kuwahara, T. & Iwatsubo, T. The Emerging Functions of LRRK2 and Rab GTPases in the Endolysosomal System. *Front Neurosci* **14**, 227 (2020).

210. Dehay, B. *et al.* Pathogenic lysosomal depletion in Parkinson's disease. *J Neurosci* **30**, 12535–12544 (2010).

211. Berkovic, S. F. *et al.* Array-based gene discovery with three unrelated subjects shows SCARB2/LIMP-2 deficiency causes myoclonus epilepsy and glomerulosclerosis. *Am J Hum Genet* **82**, 673–684 (2008).

212. Mayo, L. *et al.* Dual role of CD38 in microglial activation and activation-induced cell death. *J Immunol* **181**, 92–103 (2008).

213. Camacho-Pereira, J. *et al.* CD38 Dictates Age-Related NAD Decline and Mitochondrial Dysfunction through an SIRT3-Dependent Mechanism. *Cell Metab* **23**, 1127–1139 (2016).

214. Nabar, N. R. *et al.* LRRK2 is required for CD38-mediated NAADP-Ca2+ signaling and the downstream activation of TFEB (transcription factor EB) in immune cells. *Autophagy* **18**, 204–222 (2022).

215. Guerreiro, S., Privat, A.-L., Bressac, L. & Toulorge, D. CD38 in Neurodegeneration and Neuroinflammation. *Cells* **9**, E471 (2020).

216. Nalls, M. A. *et al.* Identification of novel risk loci, causal insights, and heritable risk for Parkinson's disease: a meta-analysis of genome-wide association studies. *Lancet Neurol* **18**, 1091–1102 (2019).

217. Yu, X. *et al.* De Novo and Inherited SETD1A Variants in Early-onset Epilepsy. *Neurosci Bull* **35**, 1045–1057 (2019).

218. Kummeling, J. *et al.* Characterization of SETD1A haploinsufficiency in humans and Drosophila defines a novel neurodevelopmental syndrome. *Mol Psychiatry* **26**, 2013–2024 (2021).

219. Ripke, S. *et al.* Biological insights from 108 schizophrenia-associated genetic loci. *Nature* **511**, 421–427 (2014).

220. Chung, W. K., Roberts, T. P., Sherr, E. H., Snyder, L. G. & Spiro, J. E. 16p11.2 deletion syndrome. *Curr Opin Genet Dev* **68**, 49–56 (2021).

221. McCarthy, S. E. *et al.* Microduplications of 16p11.2 are associated with schizophrenia. *Nat Genet* **41**, 1223–1227 (2009).

222. Mercer, C. A., Kaliappan, A. & Dennis, P. B. A novel, human Atg13 binding protein, Atg101, interacts with ULK1 and is essential for macroautophagy. *Autophagy* **5**, 649–662 (2009).

223. Merenlender-Wagner, A. *et al.* Autophagy has a key role in the pathophysiology of schizophrenia. *Mol Psychiatry* **20**, 126–132 (2015).

224. Kulicke, C. A. *et al.* The P5-type ATPase ATP13A1 modulates major histocompatibility complex I-related protein 1 (MR1)-mediated antigen presentation. *J Biol Chem* **298**, 101542 (2022).

225. Baez-Nieto, D. *et al.* Analysing an allelic series of rare missense variants of CACNA1I in a Swedish schizophrenia cohort. *Brain* awab443 (2021) doi:10.1093/brain/awab443.

226. Pardiñas, A. F. *et al.* Common schizophrenia alleles are enriched in mutation-intolerant genes and in regions under strong background selection. *Nat Genet* **50**, 381–389 (2018).

227. Cha, P.-C. *et al.* A genome-wide association study identifies SNP in DCC is associated with gallbladder cancer in the Japanese population. *J Hum Genet* **57**, 235–237 (2012).

228. Astle, W. J. *et al.* The Allelic Landscape of Human Blood Cell Trait Variation and Links to Common Complex Disease. *Cell* **167**, 1415-1429.e19 (2016).

229. Kou, I. *et al.* Common variants in a novel gene, FONG on chromosome 2q33.1 confer risk of osteoporosis in Japanese. *PLoS One* **6**, e19641 (2011).

230. Anderson, D. *et al.* First genome-wide association study in an Australian aboriginal population provides insights into genetic risk factors for body mass index and type 2 diabetes. *PLoS One* **10**, e0119333 (2015).

231. Yang, Y. *et al.* Transgenic overexpression of furin increases epileptic susceptibility. *Cell Death Dis* **9**, 1058 (2018).

232. Shang, J. *et al.* Cell entry mechanisms of SARS-CoV-2. *Proc Natl Acad Sci U S A* **117**, 11727–11734 (2020).

233. Autism Spectrum Disorders Working Group of The Psychiatric Genomics Consortium. Meta-analysis of GWAS of over 16,000 individuals with autism spectrum disorder highlights a novel locus at 10q24.32 and a significant overlap with schizophrenia. *Mol Autism* **8**, 21 (2017).

234. Karlsson Linnér, R. *et al.* Genome-wide association analyses of risk tolerance and risky behaviors in over 1 million individuals identify hundreds of loci and shared genetic influences. *Nat Genet* **51**, 245–257 (2019).

235. Matsunaga, H. *et al.* Transethnic Meta-Analysis of Genome-Wide Association Studies Identifies Three New Loci and Characterizes Population-Specific Differences for Coronary Artery Disease. *Circ Genom Precis Med* **13**, e002670 (2020).

236. Pilling, L. C. *et al.* Human longevity: 25 genetic loci associated in 389,166 UK biobank participants. *Aging (Albany NY)* **9**, 2504–2520 (2017).

237. Brackertz, M., Boeke, J., Zhang, R. & Renkawitz, R. Two highly related p66 proteins comprise a new family of potent transcriptional repressors interacting with MBD2 and MBD3. *J Biol Chem* **277**, 40958–40966 (2002).

238. Lu, D. *et al.* A shared genetic contribution to breast cancer and schizophrenia. *Nat Commun* **11**, 4637 (2020).

239. Zhao, W. *et al.* Identification of new susceptibility loci for type 2 diabetes and shared etiological pathways with coronary heart disease. *Nat Genet* **49**, 1450–1457 (2017).

240. Sass, J. O. *et al.* D-glyceric aciduria is caused by genetic deficiency of D-glycerate kinase (GLYCTK). *Hum Mutat* **31**, 1280–1285 (2010).

241. Spies, T., Blanck, G., Bresnahan, M., Sands, J. & Strominger, J. L. A new cluster of genes within the human major histocompatibility complex. *Science* **243**, 214–217 (1989).

242. Thomas, E. A. Histone Posttranslational Modifications in Schizophrenia. *Adv Exp Med Biol* **978**, 237–254 (2017).

243. Locke, A. E. *et al.* Genetic studies of body mass index yield new insights for obesity biology. *Nature* **518**, 197–206 (2015).

244. Sun, H. *et al.* ACF chromatin-remodeling complex mediates stress-induced depressive-like behavior. *Nat Med* **21**, 1146–1153 (2015).

245. Escamilla, C. O. *et al.* Kctd13 deletion reduces synaptic transmission via increased RhoA. *Nature* **551**, 227–231 (2017).

246. O'Donnell-Luria, A. H. *et al.* Heterozygous Variants in KMT2E Cause a Spectrum of Neurodevelopmental Disorders and Epilepsy. *Am J Hum Genet* **104**, 1210–1222 (2019).

247. Liu, M. *et al.* Association studies of up to 1.2 million individuals yield new insights into the genetic etiology of tobacco and alcohol use. *Nat Genet* **51**, 237–244 (2019).

248. Zhang, F. *et al.* Clinical value of jointly detection pleural fluid Midkine, pleural fluid adenosine deaminase, and pleural fluid carbohydrate antigen 125 in the identification of nonsmall cell lung cancer-associated malignant pleural effusion. *J Clin Lab Anal* **32**, e22576 (2018).

249. Mashaly, A. H., Anwar, R., Ebrahim, M. A., Eissa, L. A. & El Shishtawy, M. M. Diagnostic and Prognostic Value of Talin-1 and Midkine as Tumor Markers in Hepatocellular Carcinoma in Egyptian Patients. *Asian Pac J Cancer Prev* **19**, 1503–1508 (2018).

250. Bauer, S. *et al.* Activation of NK cells and T cells by NKG2D, a receptor for stress-inducible MICA. *Science* **285**, 727–729 (1999).

251. Shirts, B. H. *et al.* Polymorphisms in MICB are associated with human herpes virus seropositivity and schizophrenia risk. *Schizophr Res* **94**, 342–353 (2007).

252. Wahbeh, M. H. & Avramopoulos, D. Gene-Environment Interactions in Schizophrenia: A Literature Review. *Genes (Basel)* **12**, 1850 (2021).

253. Witters, P. *et al.* Autism in patients with propionic acidemia. *Mol Genet Metab* **119**, 317–321 (2016).
254. Lee, J. J. *et al.* Gene discovery and polygenic prediction from a genome-wide association study of educational attainment in 1.1 million individuals. *Nat Genet* **50**, 1112–1121 (2018).
255. Nagel, M. *et al.* Meta-analysis of genome-wide association studies for neuroticism in 449,484 individuals identifies novel genetic loci and pathways. *Nat Genet* **50**, 920–927 (2018).
256. Lango Allen, H. *et al.* Hundreds of variants clustered in genomic loci and biological pathways affect human height. *Nature* **467**, 832–838 (2010).
257. Pulit, S. L. *et al.* Meta-analysis of genome-wide association studies for body fat distribution in 694 649 individuals of European ancestry. *Hum Mol Genet* **28**, 166–174 (2019).
258. Sabater-Lleal, M. *et al.* Multiethnic meta-analysis of genome-wide association studies in >100 000 subjects identifies 23 fibrinogen-associated Loci but no strong evidence of a causal association between circulating fibrinogen and cardiovascular disease. *Circulation* **128**, 1310–1324 (2013).
259. Ligthart, S. *et al.* Genome Analyses of >200,000 Individuals Identify 58 Loci for Chronic Inflammation and Highlight Pathways that Link Inflammation and Complex Disorders. *Am J Hum Genet* **103**, 691–706 (2018).
260. Surakka, I. *et al.* The impact of low-frequency and rare variants on lipid levels. *Nat Genet* **47**, 589–597 (2015).
261. Hoffmann, T. J. *et al.* A large electronic-health-record-based genome-wide study of serum lipids. *Nat Genet* **50**, 401–413 (2018).

262. Bosc, D. G. *et al.* Identification and characterization of CKIP-1, a novel pleckstrin homology domain-containing protein that interacts with protein kinase CK2. *J Biol Chem* **275**, 14295–14306 (2000).

263. Hédou, G. F. *et al.* Protein phosphatase 1-dependent bidirectional synaptic plasticity controls ischemic recovery in the adult brain. *J Neurosci* **28**, 154–162 (2008).

264. Grad, M. *et al.* Altered White Matter and microRNA Expression in a Murine Model Related to Williams Syndrome Suggests That miR-34b/c Affects Brain Development via Ptpru and Dcx Modulation. *Cells* **11**, 158 (2022).

265. Fregeau, B. *et al.* De Novo Mutations of RERE Cause a Genetic Syndrome with Features that Overlap Those Associated with Proximal 1p36 Deletions. *Am J Hum Genet* **98**, 963–970 (2016).

266. Lam, M. *et al.* Pleiotropic Meta-Analysis of Cognition, Education, and Schizophrenia Differentiates Roles of Early Neurodevelopmental and Adult Synaptic Pathways. *Am J Hum Genet* **105**, 334–350 (2019).

267. Turley, P. *et al.* Multi-trait analysis of genome-wide association summary statistics using MTAG. *Nat Genet* **50**, 229–237 (2018).

268. Howard, D. M. *et al.* Genome-wide meta-analysis of depression identifies 102 independent variants and highlights the importance of the prefrontal brain regions. *Nat Neurosci* **22**, 343–352 (2019).

269. Chen, M.-H. *et al.* Trans-ethnic and Ancestry-Specific Blood-Cell Genetics in 746,667 Individuals from 5 Global Populations. *Cell* **182**, 1198-1213.e14 (2020).

270. Craig, J. E. *et al.* Multitrait analysis of glaucoma identifies new risk loci and enables polygenic prediction of disease susceptibility and progression. *Nat Genet* **52**, 160–166 (2020).

271. Springelkamp, H. *et al.* New insights into the genetics of primary open-angle glaucoma based on meta-analyses of intraocular pressure and optic disc characteristics. *Hum Mol Genet* **26**, 438–453 (2017).

272. Hysi, P. G. *et al.* Meta-analysis of 542,934 subjects of European ancestry identifies new genes and mechanisms predisposing to refractive error and myopia. *Nat Genet* **52**, 401–407 (2020).

273. Giri, A. *et al.* Trans-ethnic association study of blood pressure determinants in over 750,000 individuals. *Nat Genet* **51**, 51–62 (2019).

274. Morris, J. A. *et al.* An atlas of genetic influences on osteoporosis in humans and mice. *Nat Genet* **51**, 258–266 (2019).

275. Zhu, Z. *et al.* Shared genetics of asthma and mental health disorders: a large-scale genome-wide cross-trait analysis. *Eur Respir J* **54**, (2019).

276. Han, Y. *et al.* Genome-wide analysis highlights contribution of immune system pathways to the genetic architecture of asthma. *Nat Commun* **11**, 1776 (2020).

277. Zhang, Z. et al. Molecular architecture of the human 17S U2 snRNP. *Nature* **583**, 310–313 (2020).

278. Zhang, Y. *et al.* Inhibition of Splicing Factor 3b Subunit 1 (SF3B1) Reduced Cell Proliferation, Induced Apoptosis and Resulted in Cell Cycle Arrest by Regulating Homeobox A10 (HOXA10) Splicing in AGS and MKN28 Human Gastric Cancer Cells. *Med Sci Monit* **26**, e919460 (2020).

279. Li, C. *et al.* Somatic SF3B1 hotspot mutation in prolactinomas. *Nat Commun* **11**, 2506 (2020).

280. Tang, A. D. *et al.* Full-length transcript characterization of SF3B1 mutation in chronic lymphocytic leukemia reveals downregulation of retained introns. *Nat Commun* **11**, 1438 (2020).

281. Hyde, C. L. *et al.* Identification of 15 genetic loci associated with risk of major depression in individuals of European descent. *Nat Genet* **48**, 1031–1036 (2016).

282. Zhou, S., Sousa, R., Tannery, N. H. & Lafer, E. M. Characterization of a novel synapse-specific protein. II. cDNA cloning and sequence analysis of the F1-20 protein. *J Neurosci* **12**, 2144–2155 (1992).

283. Ryan, T. A. A pre-synaptic to-do list for coupling exocytosis to endocytosis. *Curr Opin Cell Biol* **18**, 416–421 (2006).

284. Zihni, C. *et al.* Prostate-derived sterile 20-like kinase 1-alpha induces apoptosis. JNK- and caspase-dependent nuclear localization is a requirement for membrane blebbing. *J Biol Chem* **282**, 6484–6493 (2007).

285. de Anda, F. C. *et al.* Autism spectrum disorder susceptibility gene TAOK2 affects basal dendrite formation in the neocortex. *Nat Neurosci* **15**, 1022–1031 (2012).

286. Mahdessian, H. *et al.* TM6SF2 is a regulator of liver fat metabolism influencing triglyceride secretion and hepatic lipid droplet content. *Proc Natl Acad Sci U S A* **111**, 8913–8918 (2014).

287. Anstee, Q. M. & Day, C. P. The Genetics of Nonalcoholic Fatty Liver Disease: Spotlight on PNPLA3 and TM6SF2. *Semin Liver Dis* **35**, 270–290 (2015).

288. Lee, C.-M. *et al.* IL-13Rα2 uses TMEM219 in chitinase 3-like-1-induced signalling and effector responses. *Nat Commun* **7**, 12752 (2016).

289. Vilboux, T. *et al.* A congenital neutrophil defect syndrome associated with mutations in VPS45. *N Engl J Med* **369**, 54–65 (2013).

290. Rhee, E. P. *et al.* A genome-wide association study of the human metabolome in a community-based cohort. *Cell Metab* **18**, 130–143 (2013).

291. Paradiso, S., Andreasen, N. C., O'Leary, D. S., Arndt, S. & Robinson, R. G. Cerebellar size and cognition: correlations with IQ, verbal memory and motor dexterity. *Neuropsychiatry Neuropsychol Behav Neurol* **10**, 1–8 (1997).

292. Parmeggiani, A., Posar, A., Scaduto, M. C., Chiodo, S. & Giovanardi-Rossi, P. Epilepsy, intelligence, and psychiatric disorders in patients with cerebellar hypoplasia. *J Child Neurol* **18**, 1–4 (2003).

293. Yoon, Y. B. *et al.* Brain Structural Networks Associated with Intelligence and Visuomotor Ability. *Sci Rep* **7**, 2177 (2017).

294. Jeste, S. S. The Neurology of Autism Spectrum Disorders. *Curr Opin Neurol* **24**, 132–139 (2011).

295. Ge, T. *et al.* The Shared Genetic Basis of Educational Attainment and Cerebral Cortical Morphology. *Cereb Cortex* **29**, 3471–3481 (2019).

296. Hibar, D. P. *et al.* Common genetic variants influence human subcortical brain structures. *Nature* **520**, 224–9 (2015).

297. Tahara, T., Kraus, J. P. & Rosenberg, L. E. An unusual insertion/deletion in the gene encoding the beta-subunit of propionyl-CoA carboxylase is a frequent mutation in Caucasian propionic acidemia. *Proc Natl Acad Sci U S A* **87**, 1372–1376 (1990).

298. Wolf, B. *et al.* Propionic acidemia: A clinical update. *The Journal of Pediatrics* **99**, 835–846 (1981).

299. Dejean de la Bâtie, C. *et al.* Acute psychosis in propionic acidemia: 2 case reports. *J Child Neurol* **29**, 274–279 (2014).

300. Jaffe, A. E. *et al.* Developmental and genetic regulation of the human cortex transcriptome illuminate schizophrenia pathogenesis. *Nature Neuroscience* **21**, 1117–1125 (2018).

301. Young, A. M. H. *et al.* A map of transcriptional heterogeneity and regulatory variation in human microglia. *Nat Genet* **53**, 861–868 (2021).

302. Li, Z. *et al.* Genetic variants associated with Alzheimer's disease confer different cerebral cortex cell-type population structure. *Genome Medicine* **10**, 43 (2018).

303. Bellenguez, C. *et al.* New insights into the genetic etiology of Alzheimer's disease and related dementias. *Nat Genet* **54**, 412–436 (2022).

304. Li, B. K. *et al.* Pineoblastoma segregates into molecular sub-groups with distinct clinico-pathologic features: a Rare Brain Tumor Consortium registry study. *Acta Neuropathol* **139**, 223–241 (2020).

305. Ren, Y. *et al.* TMEM106B haplotypes have distinct gene expression patterns in aged brain. *Molecular Neurodegeneration* **13**, 35 (2018).

306. Yang, C. *et al.* Rewiring Neuronal Glycerolipid Metabolism Determines the Extent of Axon Regeneration. *Neuron* **105**, 276-292.e5 (2020).

307. Kuo, M.-W., Wang, C.-H., Wu, H.-C., Chang, S.-J. & Chuang, Y.-J. Soluble THSD7A Is an N-Glycoprotein That Promotes Endothelial Cell Migration and Tube Formation in Angiogenesis. *PLOS ONE* **6**, e29000 (2011).

308. Park, Y. *et al.* Single-cell deconvolution of 3,000 post-mortem brain samples for eQTL and GWAS dissection in mental disorders. *bioRxiv* 2021.01.21.426000 (2021) doi:10.1101/2021.01.21.426000.
309. Li, Z. *et al.* The TMEM106B FTLD-protective variant, rs1990621, is also associated with increased neuronal proportion. *Acta Neuropathol* **139**, 45–61 (2020).
310. Pers, T. H. *et al.* Biological interpretation of genome-wide association studies using predicted gene functions. *Nature Communications* **6**, 5890 (2015).
311. Deelen, P. *et al.* Improving the diagnostic yield of exome- sequencing by predicting gene–phenotype associations using large-scale gene expression analysis. *Nat Commun* **10**, 1–13 (2019).
312. Li, H. *et al.* The Sequence Alignment/Map format and SAMtools. *Bioinformatics* **25**, 2078–2079 (2009).
313. Yates, A. D. *et al.* Ensembl 2020. *Nucleic Acids Res* **48**, D682–D688 (2020).
314. Love, M. I., Huber, W. & Anders, S. Moderated estimation of fold change and dispersion for RNA-seq data with DESeq2. *Genome Biology* **15**, 550 (2014).
315. Bakker, O. B. *et al.* Linking common and rare disease genetics through gene regulatory networks. *medRxiv* (2021) doi:10.1101/2021.10.21.21265342.
316. Lamparter, D., Marbach, D., Rueedi, R., Kutalik, Z. & Bergmann, S. Fast and Rigorous Computation of Gene and Pathway Scores from SNP-Based Summary Statistics. *PLOS Computational Biology* **12**, e1004714 (2016).
317. The 1000 Genomes Project Consortium. A global reference for human genetic variation. *Nature* **526**, 68–74 (2015).

318. Deelen, P. *et al.* Improving the diagnostic yield of exome- sequencing by predicting gene–phenotype associations using large-scale gene expression analysis. *Nat Commun* **10**, 1–13 (2019).

319. Gabriele, M., Lopez Tobon, A., D'Agostino, G. & Testa, G. The chromatin basis of neurodevelopmental disorders: Rethinking dysfunction along the molecular and temporal axes. *Progress in Neuro-Psychopharmacology and Biological Psychiatry* **84**, 306–327 (2018).

320. Poisson, A. *et al.* Chromatin remodeling dysfunction extends the etiological spectrum of schizophrenia: a case report. *BMC Med Genet* **21**, (2020).

321. What is schizophrenia: A neurodevelopmental or neurodegenerative disorder or a combination of both? A critical analysis. https://www.ncbi.nlm.nih.gov/pmc/articles/PMC2824976/.

322. Slowikowski, K. *et al.* ggrepel: Automatically Position Non-Overlapping Text Labels with 'ggplot2'. (2021).

323. Wickham, H. *ggplot2: Elegant Graphics for Data Analysis*. (Springer-Verlag, 2009). doi:10.1007/978-0-387-98141-3.

324. Kassambara, A. ggpubr: 'ggplot2' Based Publication Ready Plots. (2020).

325. Garnier, S., Ross, N., Rudis, B., Sciaini, M. & Scherer, C. viridis: Default Color Maps from 'matplotlib'. (2018).

326. Sarkar, D. *Lattice: Multivariate Data Visualization with R*. (Springer-Verlag, 2008). doi:10.1007/978-0-387-75969-2.

327. Auguie, B. & Antonov, A. gridExtra: Miscellaneous Functions for 'Grid' Graphics. (2017).

328. Dowle, M. *et al.* data.table: Extension of 'data.frame'. (2021).

329. Wickham, H., François, R., Henry, L., Müller, K. & RStudio. dplyr: A Grammar of Data Manipulation. (2021).

330. Wickham, H. *et al.* readxl: Read Excel Files. (2019).

331. Wickham, H., Seidel, D. & RStudio. scales: Scale Functions for Visualization. (2020).

332. Schloerke, B. *et al.* GGally: Extension to 'ggplot2'. (2021).

333. Robinson, M. D., McCarthy, D. J. & Smyth, G. K. edgeR: a Bioconductor package for differential expression analysis of digital gene expression data. *Bioinformatics* **26**, 139–140 (2010).

334. Attali, D. & Baker, C. ggExtra: Add Marginal Histograms to 'ggplot2', and More 'ggplot2' Enhancements. (2019).

335. Wickham, H., Pedersen, T. L. & RStudio. gtable: Arrange 'Grobs' in Tables. (2019).

336. Bengtsson, H. *et al.* matrixStats: Functions that Apply to Rows and Columns of Matrices (and to Vectors). (2021).

337. Tierney, N. *et al.* naniar: Data Structures, Summaries, and Visualisations for Missing Data. (2020).

338. Wickham, H. The Split-Apply-Combine Strategy for Data Analysis. *Journal of Statistical Software* **40**, 1–29 (2011).

339. Wickham, H. Reshaping Data with the reshape Package. *Journal of Statistical Software* **21**, 1–20 (2007).

340. Wickham, H. & RStudio. stringr: Simple, Consistent Wrappers for Common String Operations. (2019).

341. Wickham, H. & RStudio. tidyr: Tidy Messy Data. (2020).

342. Welcome to the Tidyverse. https://tidyverse.tidyverse.org/articles/paper.html.

343. Alexa, A. & Rahnenfuhrer, J. topGO: Enrichment Analysis for Gene Ontology. (2021) doi:10.18129/B9.bioc.topGO.

344. Waskom, M. *et al.* mwaskom/seaborn: v0.11.1 (December 2020). (2020) doi:10.5281/ZENODO.592845.

345. Hunter, J. D. Matplotlib: A 2D Graphics Environment. *Comput. Sci. Eng.* **9**, 90–95 (2007).

346. Harris, C. R. *et al.* Array programming with NumPy. *Nature* **585**, 357–362 (2020).

347. Seabold, S. & Perktold, J. Statsmodels: Econometric and Statistical Modeling with Python. in 92–96 (2010). doi:10.25080/Majora-92bf1922-011.

348. Li, H. Tabix: fast retrieval of sequence features from generic TAB-delimited files. *Bioinformatics* **27**, 718–719 (2011).

349. Slowikowski, K. slowkow/pytabix. (2020).

350. Pedregosa, F. *et al.* Scikit-learn: Machine Learning in Python. *MACHINE LEARNING IN PYTHON* 6.

351. Lex, A., Gehlenborg, N., Strobelt, H., Vuillemot, R. & Pfister, H. UpSet: Visualization of Intersecting Sets. *IEEE Transactions on Visualization and Computer Graphics* **20**, 1983–1992 (2014).

352. Nothman, J. UpSetPlot: Draw Lex et al.'s UpSet plots with Pandas and Matplotlib.

353. wget: pure python download utility.

354. Singh, P. beautifultable: Print text tables for terminals.

355. Leifer, C. pysqlite3: DB-API 2.0 interface for Sqlite 3.x.

Supplementary Figures

Supplementary Figure 1 - European Nucleotide Archive brain sample selection

(a) Principal component (PC) analysis on the expression data of 74,052 samples included in the SkyMap database shows clustering on tissue type but also many outliers with high PC1 scores. (b) Coloring on single and paired-end sequencing shows no clear clustering. (c) Coloring single cell identifies the samples with high PC1 scores as single-cell samples. (d) Re-calculation of PCs on all samples with PC score <0 in panel A-D, after covariate correction. (e) Brain and Tissue score calculated by correlating expression of known tissue and brain samples to each of the PCs. (f) As panel F, cancer score was calculated by correlating expression of known cancer genes to all PCs.

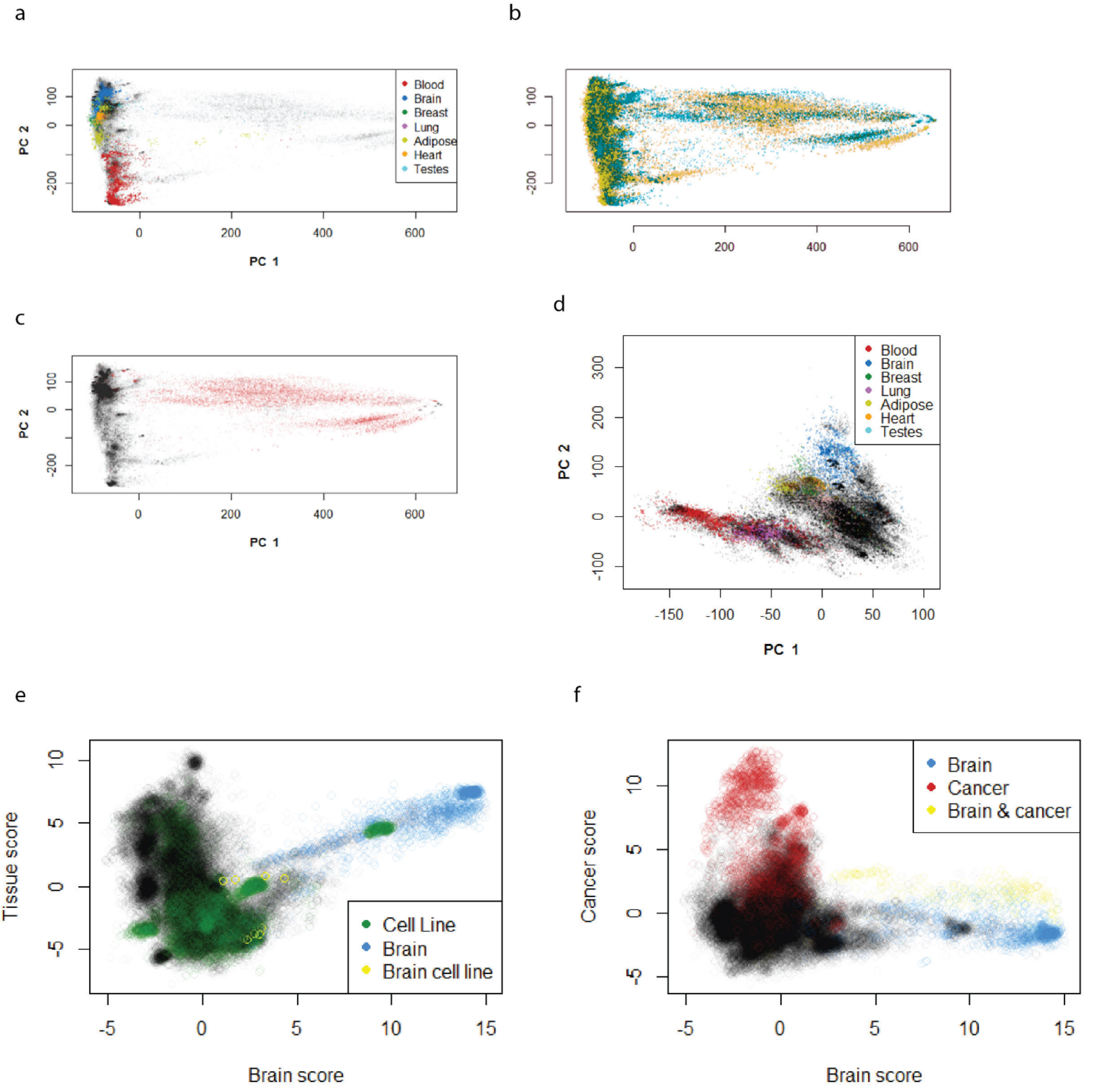

Supplementary Figure 2 - Assigning ancestry through principal component analysis

For each of the included datasets principal component (PC) scores are calculated on their genotypes. Samples are clustered with the 1000 genomes samples (left). The right panels show dataset genotype samples without 1000 genomes samples on the right projected on the same PCs. Using k-nearest neighbors clustering, samples are assigned an ancestry based on their closeness to the 1000 genomes samples of an ancestry.

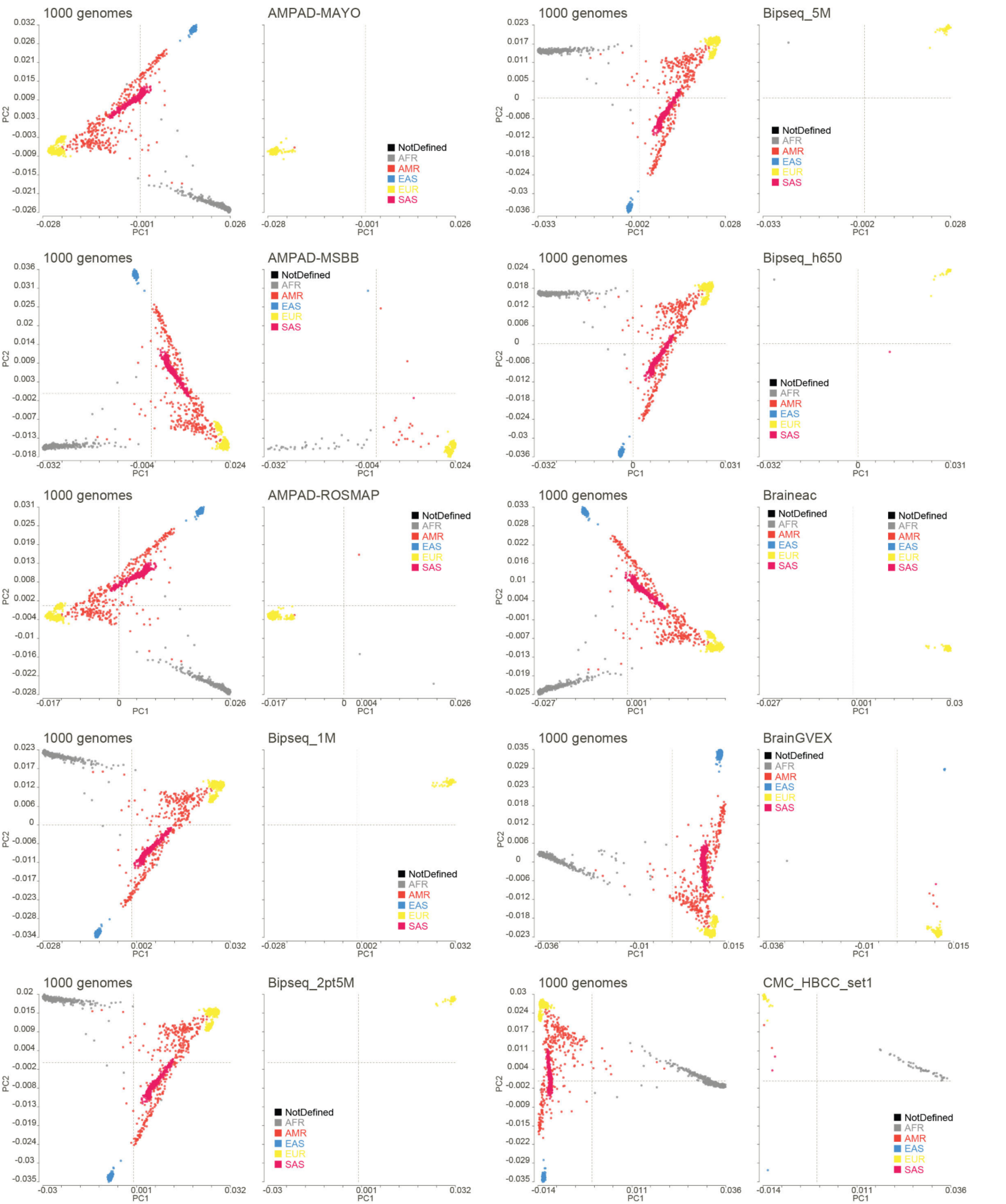

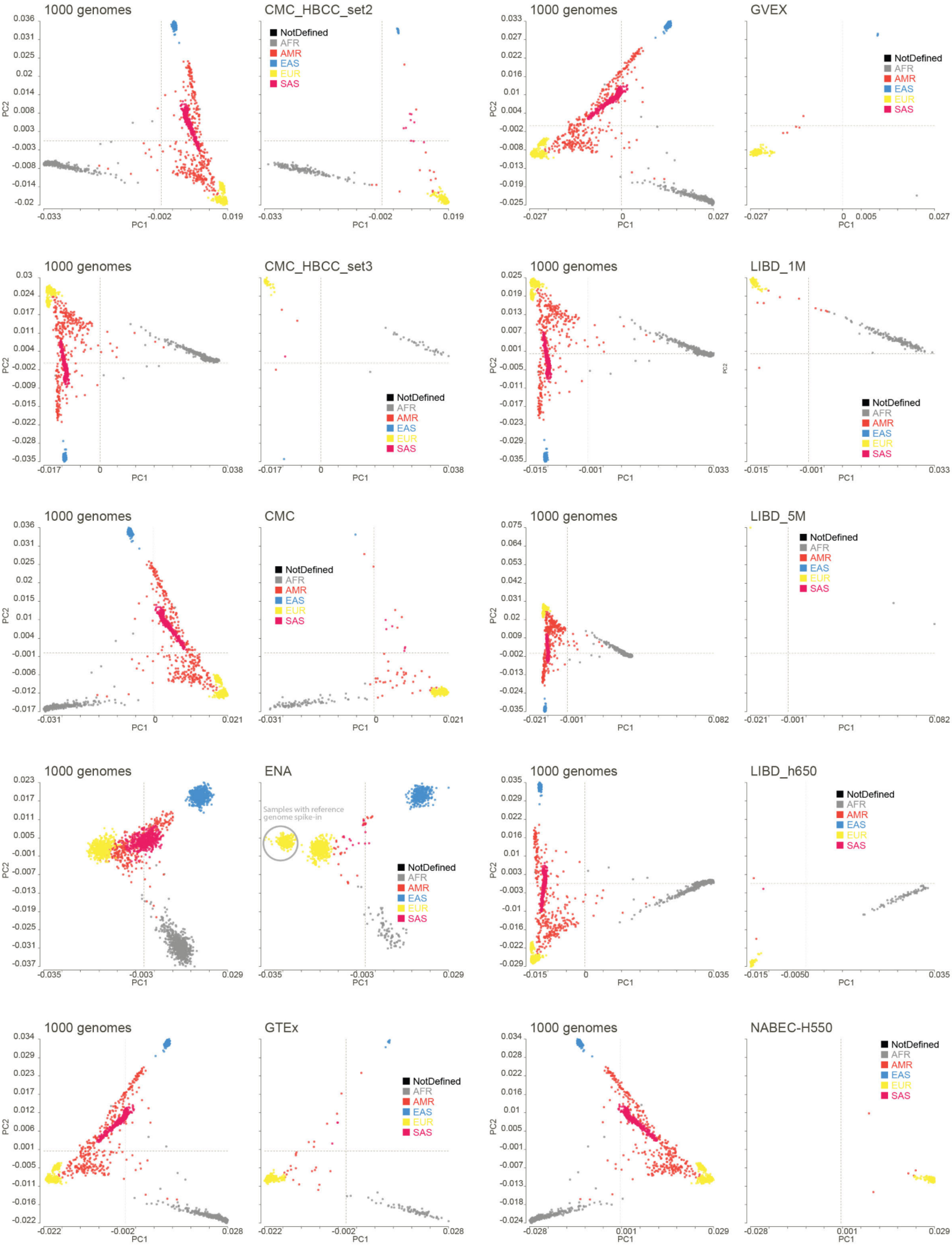

1000 genomes
CMC_HBCC_set2
1000 genomes
GVEX
1000 genomes
CMC_HBCC_set3
1000 genomes
LIBD_1M
1000 genomes
CMC
1000 genomes
LIBD_5M
1000 genomes
ENA
Samples with reference genome spike-in
1000 genomes
LIBD_h650
1000 genomes
GTEx
1000 genomes
NABEC-H550
NotDefined
AFR
AMR
EAS
EUR
SAS
PC1
PC2

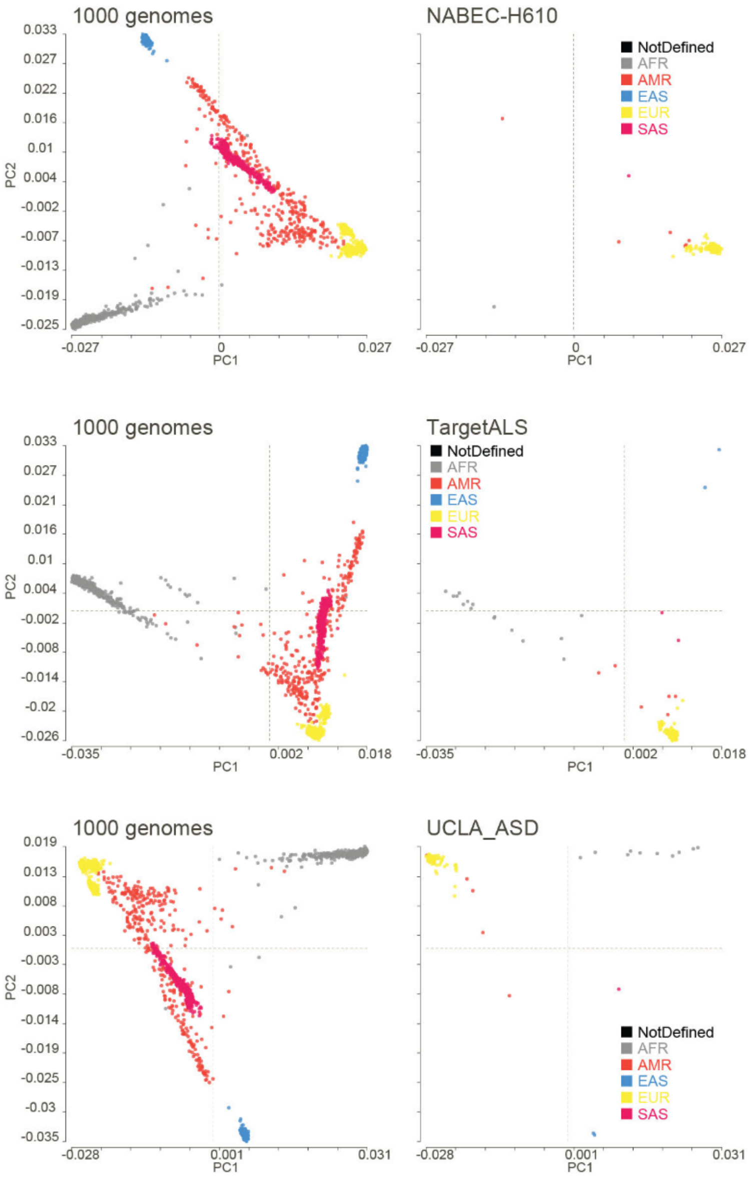

1000 genomes
NABEC-H610
NotDefined
AFR
AMR
EAS
EUR
SAS
PC1
PC2
1000 genomes
TargetALS
PC1
PC2
1000 genomes
UCLA_ASD
PC1
PC2

Supplementary Figure 3 - Properties of ENA genotype calls

(a) Allelic balance distribution of genotype calls in ENA before imputation shows clear distinction between homozygous reference, heterozygous and homozyous alternate calls. (b) Comparison of allele frequencies between ENA genotype calls and all other included datasets, after imputation shows highly similar allele frequencies independent on the type of variant. R is the Pearson correlation. P-value test statistic is based on Pearson's product moment correlation coefficient and follows a t distribution with 1,487,861-2 degrees of freedom. (c) Comparison of *cis* -eQTL Z-scores between meta-analysis and ENA shows high allelic concordance.

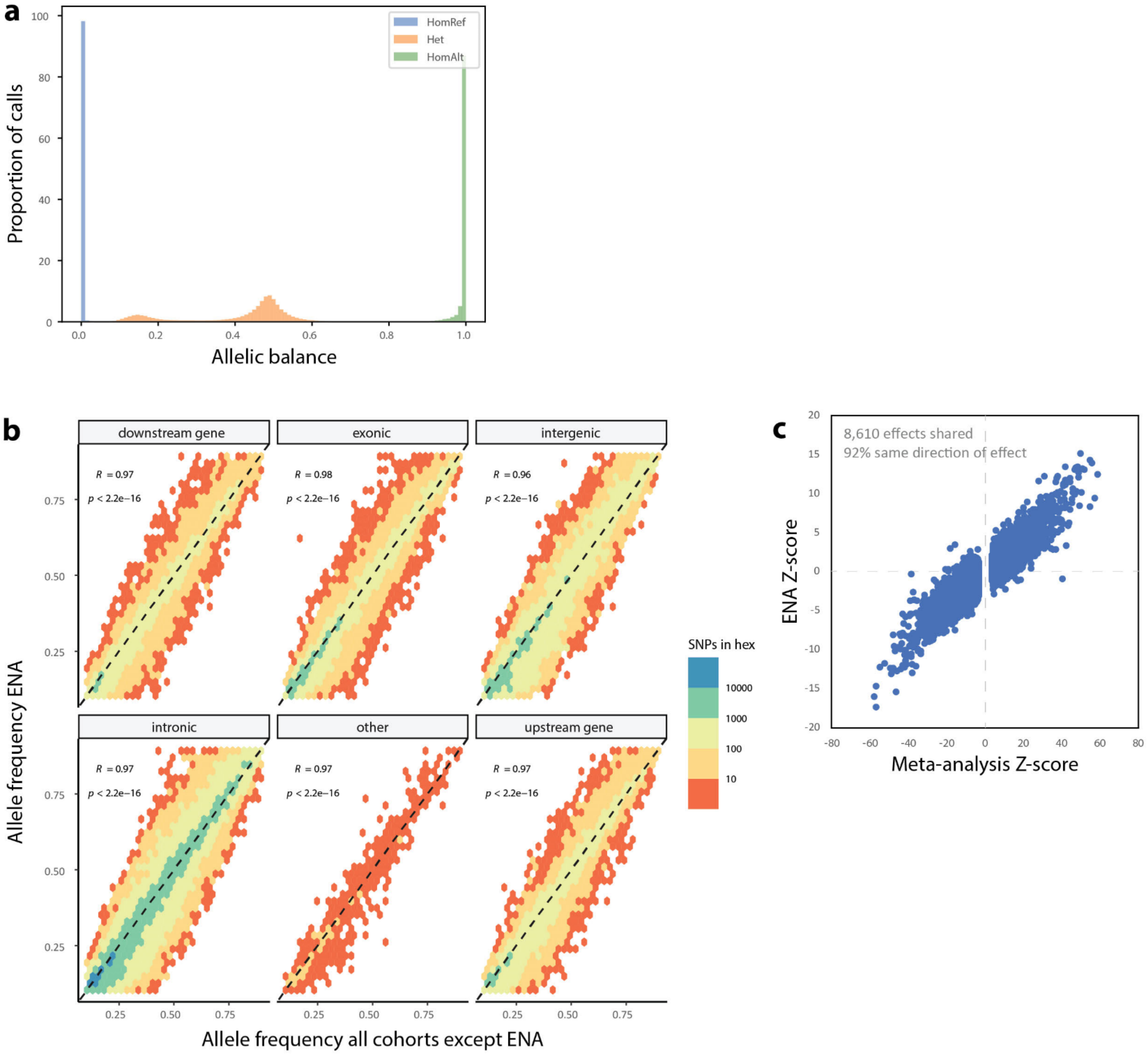

Supplementary Figure 4 - RNA-seq alignment QC

The two main RNA-seq QC metrics that are used for filtering samples. (a) Percentage coding bases colored by dataset and (b) percentage of reads aligned colored per dataset. The red dotted line is the threshold for filtering (10% for coding bases and 60% for percentage reads aligned respectively). Triangles are samples filtered out by any of the RNA-seq QC metrics.

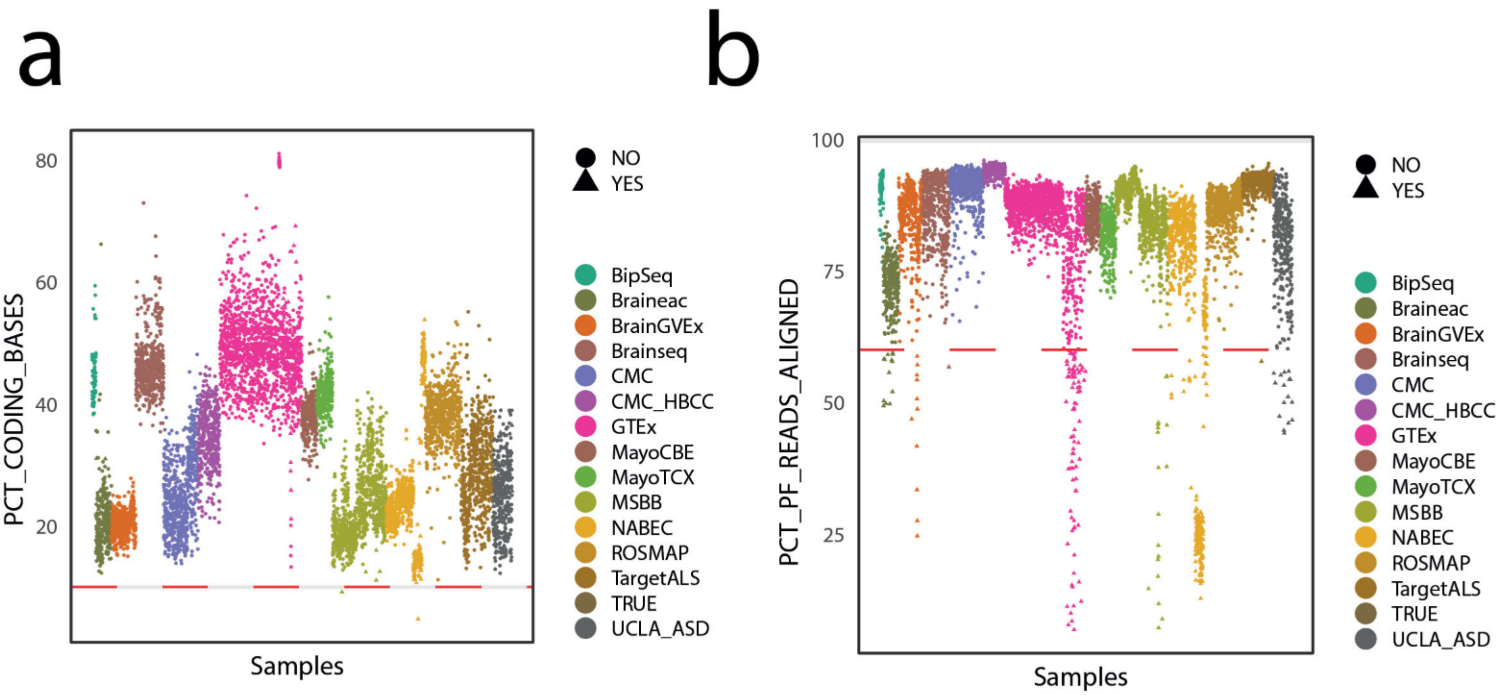

Supplementary Figure 5 - Sample filtering by PCA

Principal component analysis (PCA) plot before normalization and covariate removal. For all plots the red line indicates 4 standard deviations from the mean and red dots are samples to be filtered out. (a) PCA on all samples after removing alignment QC outliers. (b) PCA on samples after removal of outlier samples from A. (c) PCA on samples after removal of outlier samples of A and B.

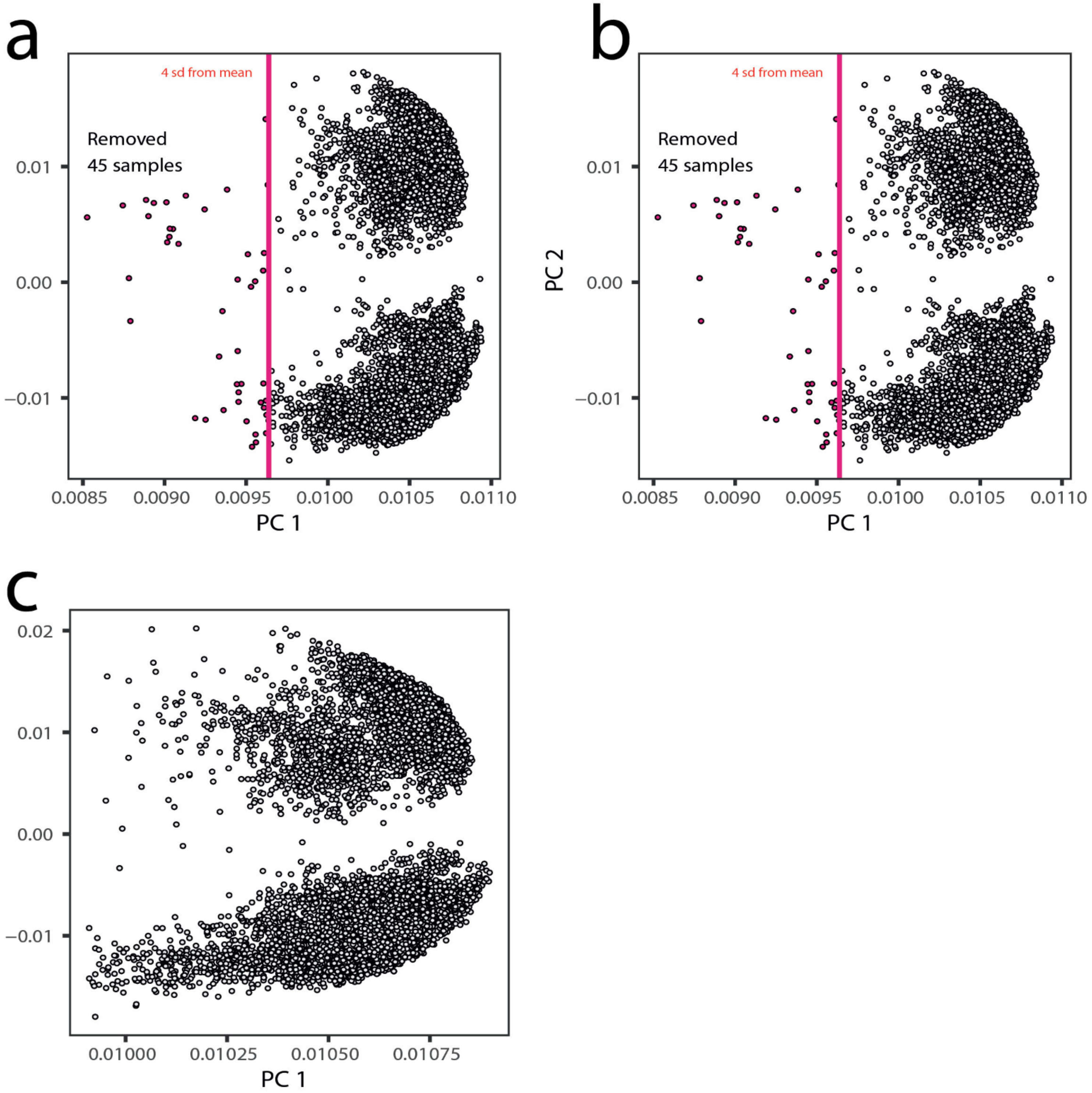

Supplementary Figure 6 - PCA before and after covariate correction

(a) PC1 and PC2 on normalized expression data before covariate correction, colored on dataset. (b) PC1 and PC2 on normalized expression data after covariate correction.

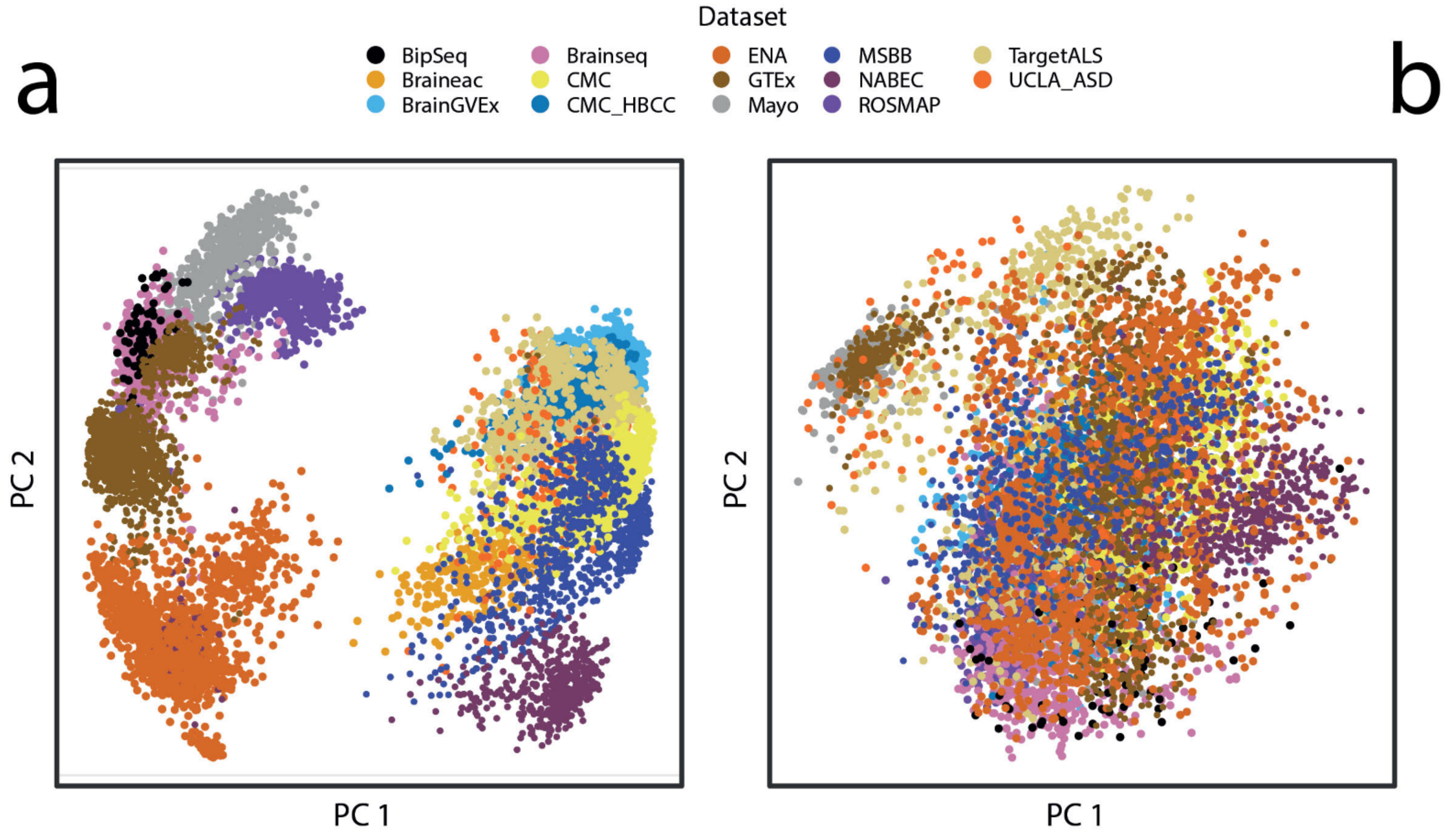

Supplementary Figure 7 - PCA optima for each dataset

Cis- and *trans*-eQTL analyses were performed with increasing numbers of principal components removed from the data. The optimal number of PCs to remove is dependent on the dataset.

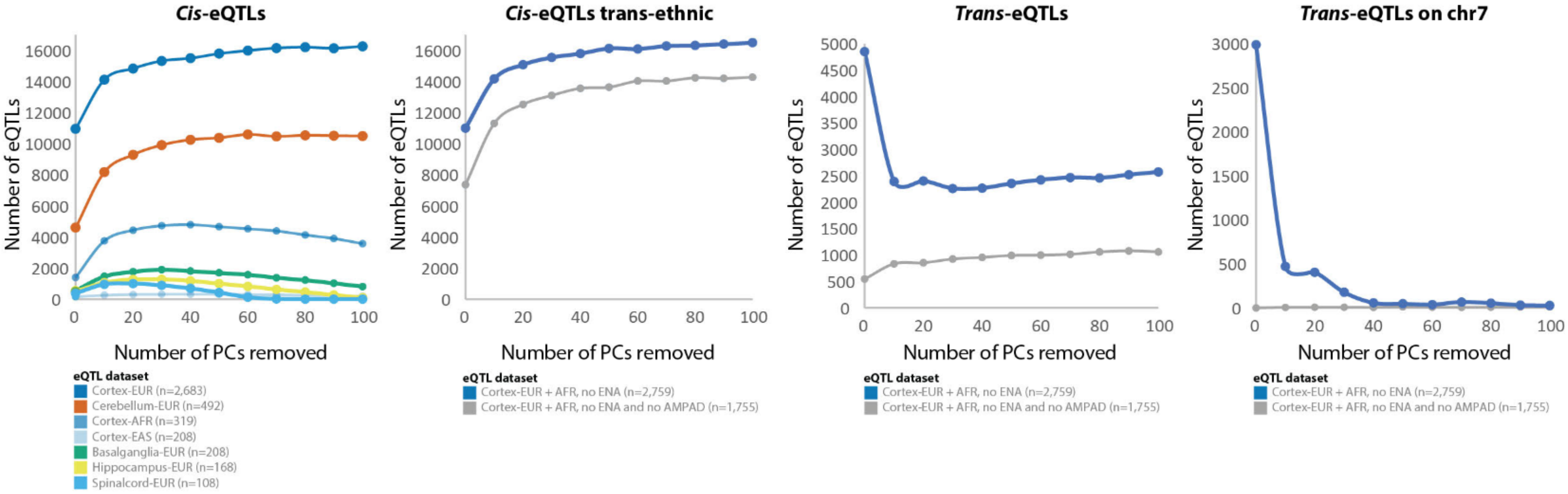

Supplementary Figure 8 - eQTL Z-score comparison between datasets

The pairwise spearman correlation and concordance of direction of the *cis*-eQTL Z-scores between all cohorts, and between each cohort and the meta-analysis Z-score. As two examples, (a) shows the Z-score comparison between Cortex-EUR eQTL datasets EUR-LIBD_h650 and EUR-UCLA_ASD, and (b) shows the Z-score comparison between the meta-analysis Z-score and the Cortex-EUR cohort EUR-AMPAD-ROSMAP-V2. (c) Correlation for each pairwise combination of cohorts between each other (small dots), and with the meta-analysis Z-scores (large dots). (d) Directional concordance for each pairwise combination of cohorts between each other (small dots), and with the meta-analysis Z-scores (large dots). The dots in (c) and (d) that correspond to the (a) and (b) plots are shown by the grey dottes lines. For both (c) and (d): Spinal cord n = 3, Hippocampus n = 3, Cortex-EUR n = 136, Cortex-AFR n = 6, Cerebellum n = 10, and Basal ganglia n = 3 cohort-cohort and cohort-meta combination. Boxplots show median (line in box), interquartile range (25th and 75th percentile, box), and minimum and maximum value (whiskers), excluding outliers (outliers are defined as less than Q1 – 1.5*(IQ3-IQ1) or greater than Q3 + 1.5*(IQ3-IQ1)). (e) Heterogeneity measured using I² shows generally low heterogeneity between dataset effect sizes.

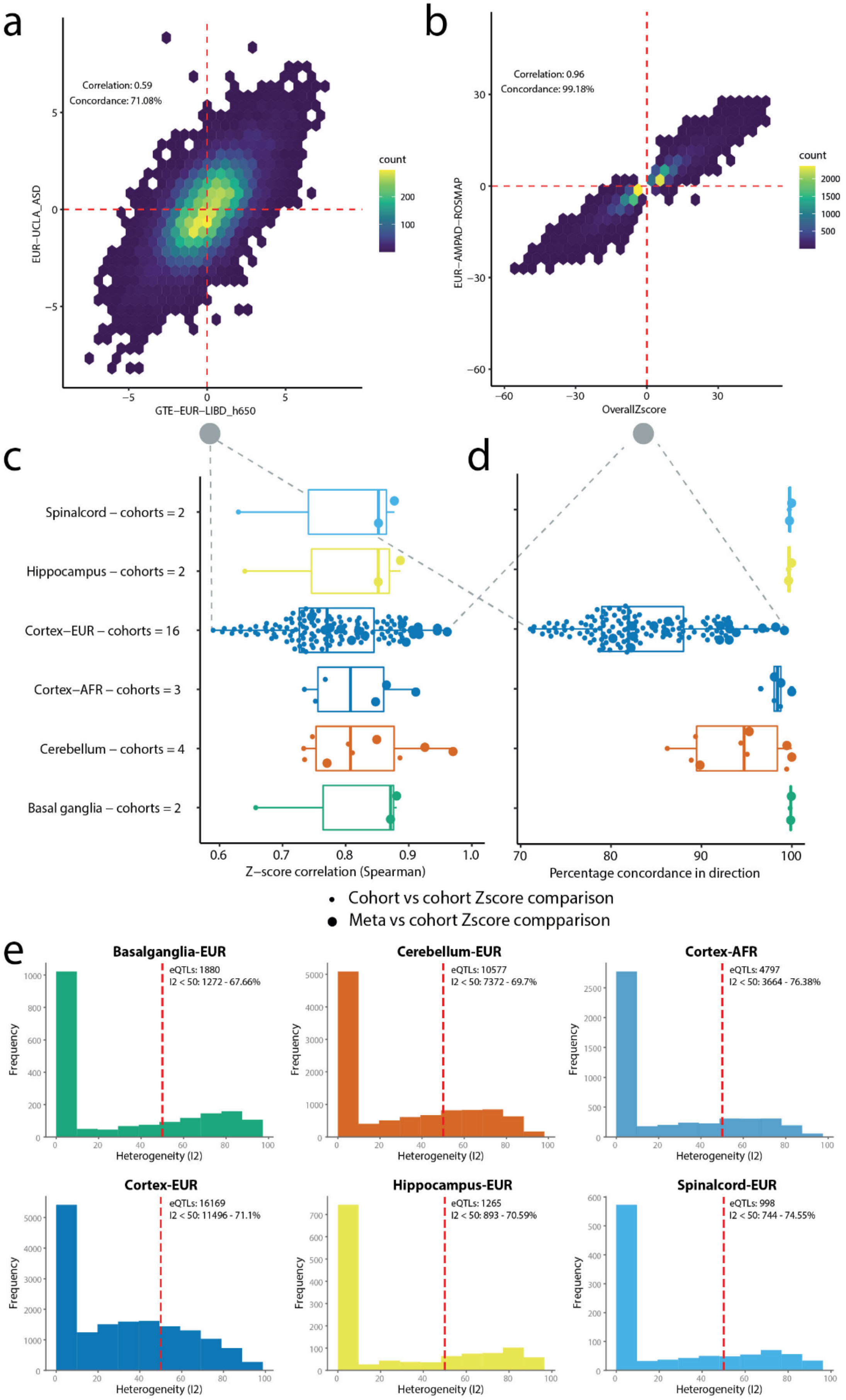

Supplementary Figure 9 - Reads mapping on patch chromosome version of *MAPT*

Number of reads mapped to the *MAPT* gene located on the primary assembly (ENSG00000186868) and the *MAPT* genes located on the patch chromosomes (ENSG00000276155 and ENSG00000277956). Each dot is an individual, and the color shows if they are homozygous reference (0/0), heterozygous (0/1), or homozygous alternative (1/1) for a SNP (rs34619181) located in the *MAPT* gene. Left plot compares counts mapped to ENSG00000186868 (ref) to those mapped to ENSG00000276155 (patch), middle plot compares ENSG00000186868 (ref) and ENSG00000277956 (patch), right plot compares ENSG00000276155 (patch) and ENSG00000277956 (patch).

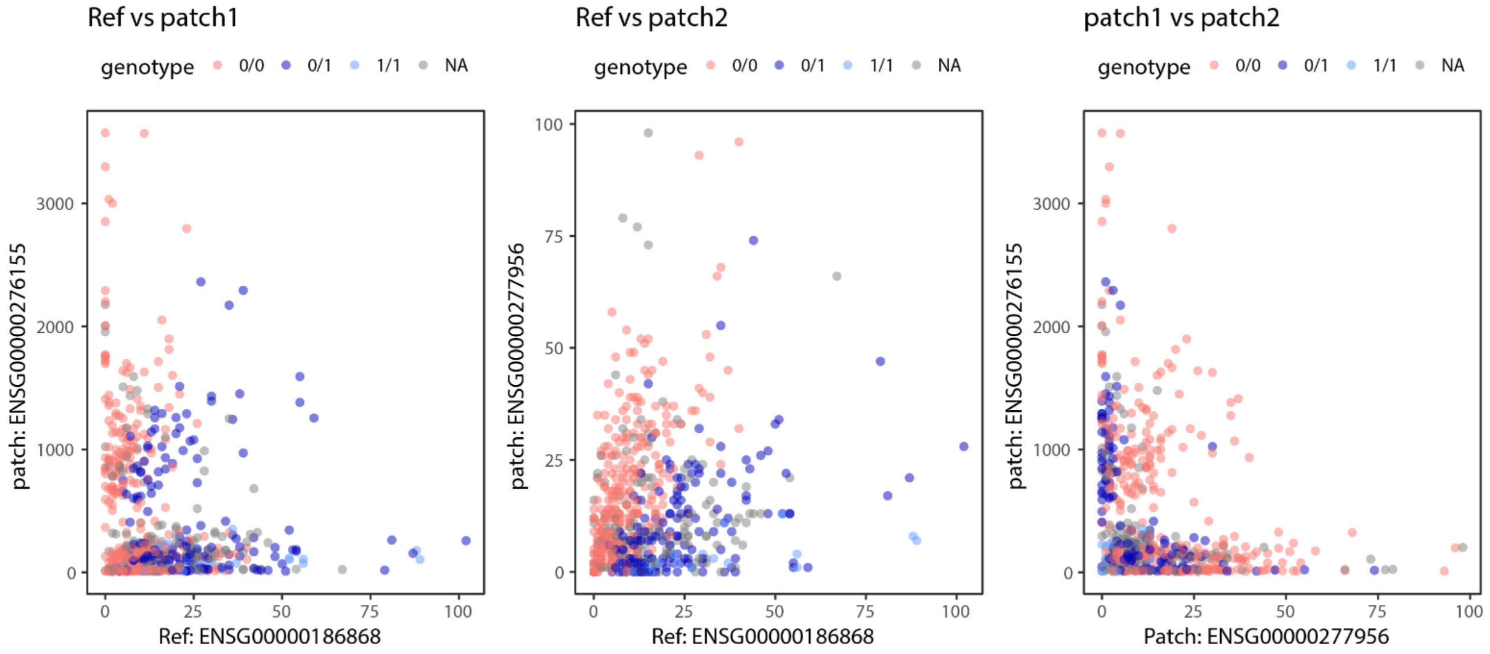

Supplementary Figure 10 - EQTL z-scores in the *MAPT* locus

Z-scores (y-axis) of the *MAPT* locus (x-axis) for all the datasets used in the Cortex-EUR meta-analysis. Left upper plot shows the meta-analysis Z-score. Blue dots are the top 100 associations.

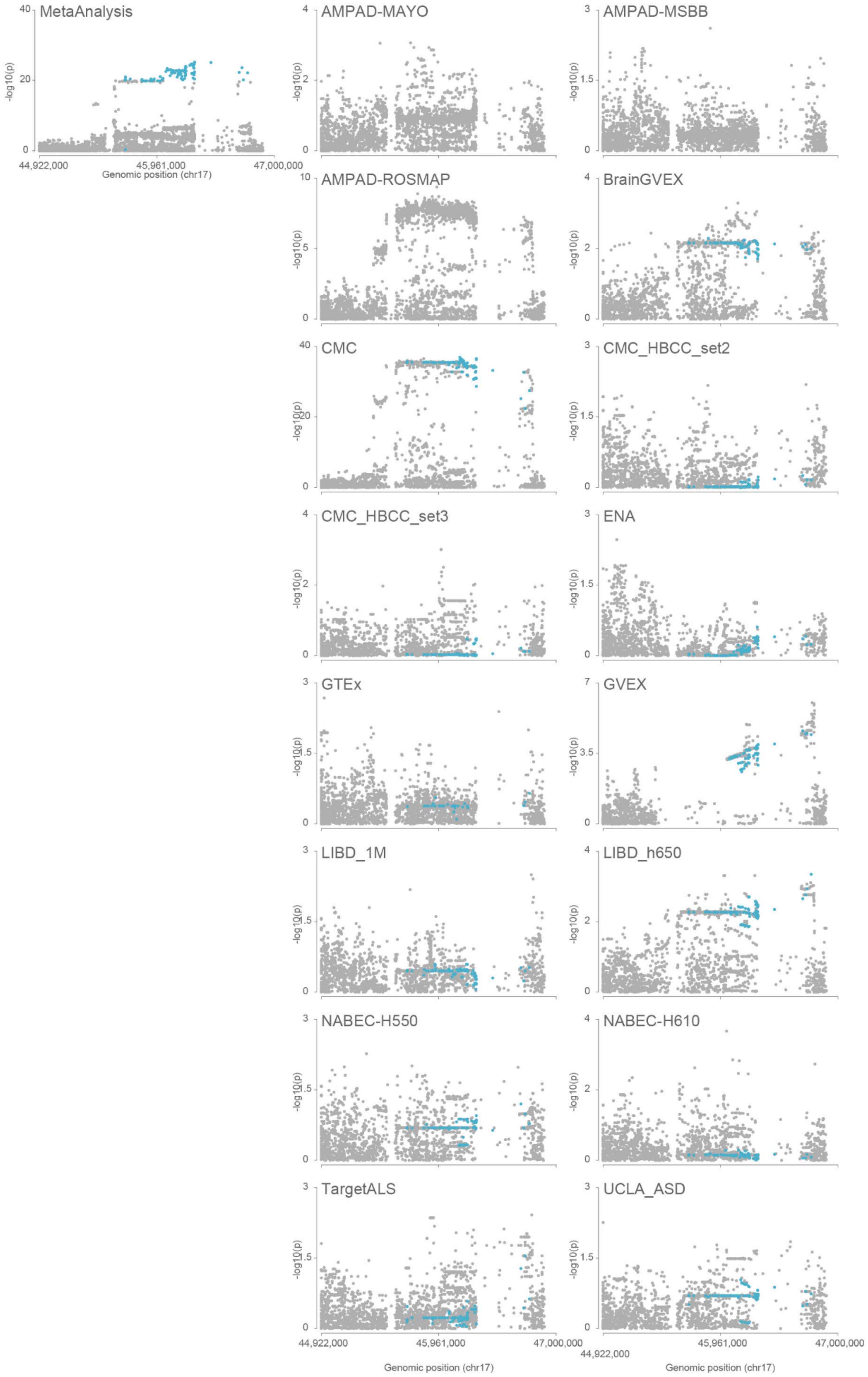

Supplementary Figure 11 - Properties of non-primary *cis*-eQTLs

(a) Percentage of tested genes that are a significant eGene per expression bin. Genes are grouped in ten bins based on average expression, from 10% lowest (bin 1) to 10% highest (bin 10). (b) Log10 of standard deviation (plus pseudo count) of all tested genes in each expression bin from A. For BIOS, n = 1,836 genes for bin 1, n = 1,835 genes for bins 2-10. For *MetaBrain*, n = 1,931 genes for bins 1-3, 5-6, and 8-10, and n = 1,930 genes for bins 4 and 7. Boxplots show median (line in box), interquartile range (25th and 75th percentile, box), and minimum and maximum value (whiskers), excluding outliers (outliers are defined as less than Q1 – 1.5*(IQ3-IQ1) or greater than Q3 + 1.5*(IQ3-IQ1)). Violin plot shows the distribution of the data. (c) g:profiler enrichment for all genes with a single independent eQTL effect. (d) g:profiler enrichment for all genes with multiple independent eQTL effects.

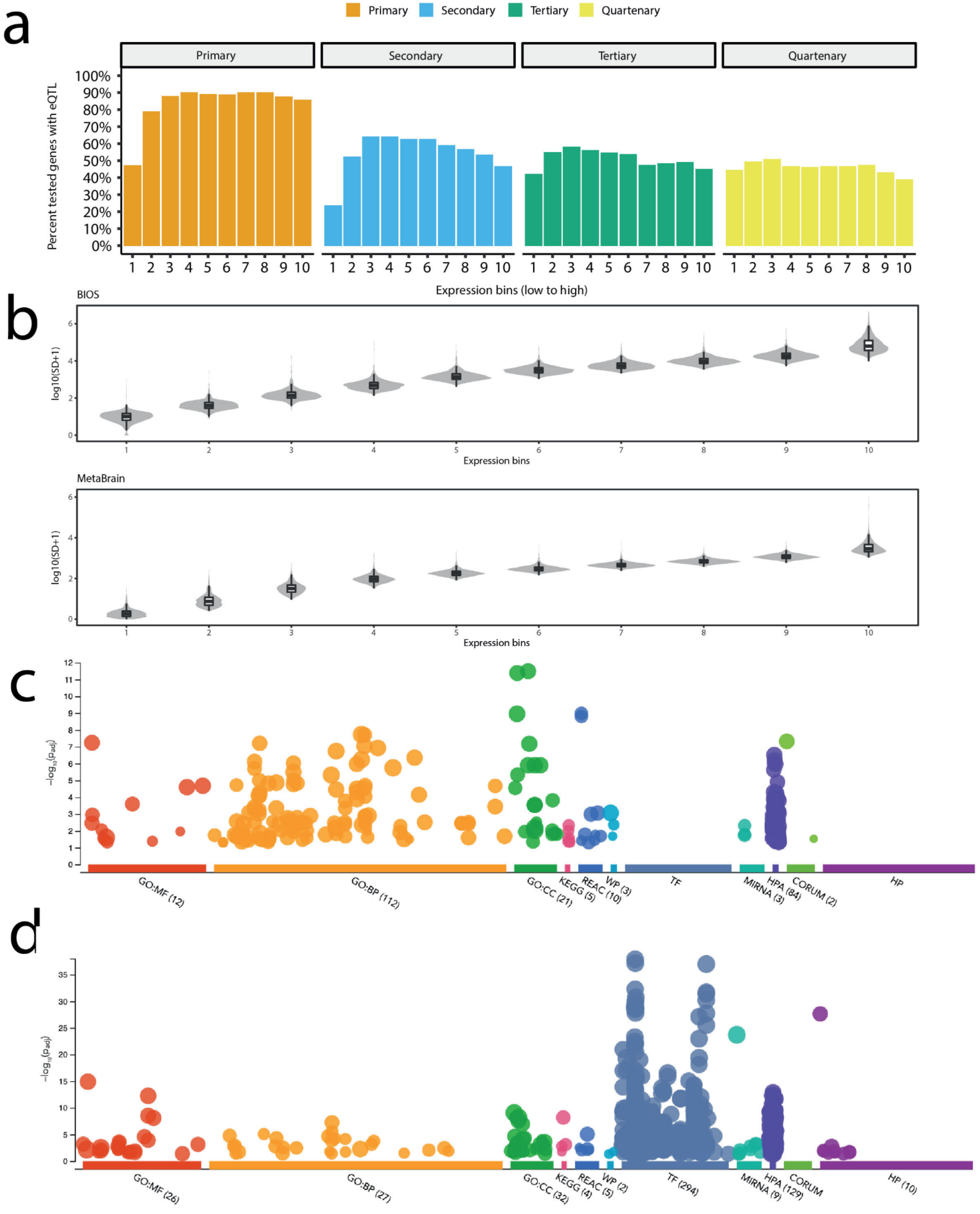

Supplementary Figure 12 - Overview of different *cis*-eQTL agreement analyses

Left to right: comparisons between ancestries in Cortex, comparisons between brain regions in *MetaBrain* and comparisons between Cortex-EUR (excluding GTEx) and GTEx. Top to bottom show different measures for agreement: Rb, AC, π1 and correlation of allelic fold change (aFC). The Rb muscle SNPs focuses the analysis on a set of muscle eQTL SNPs detected in GTEx as an unbiased analysis. Top numbers in Rb, AC, and correlation of aFC are the shared number of eQTLs.

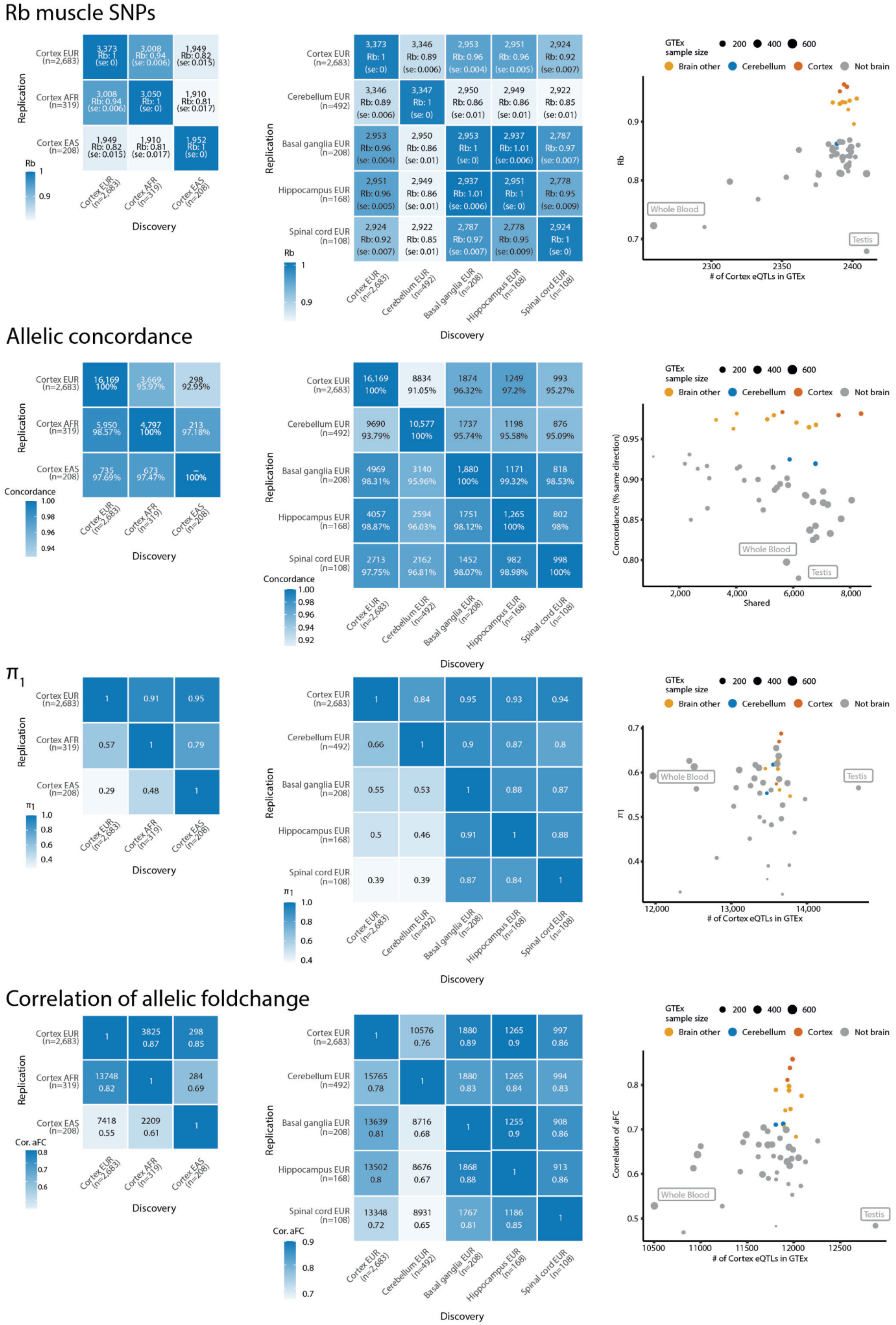

Supplementary Figure 13 - Properties of cerebellum specific eQTLs

(a) UpSet plot of the number of eQTL genes per brain region for European datasets. (b) The distribution of log2(TMM+1) expression in cortex of the 472 eQTL genes that were only significant in cerebellum. Blue line is the minima of the fitted bimodal distribution (dotted red line) and is used as cut-off point in panel c (c) The expression in cortex (x-axis) and cerebellum (y-axis) of the 846 eQTL genes that were only significant eQTLs in cerebellum. The blue line is the cut-off from panel b. (d) The mean expression (dots) and standard deviation (error bars) of the 30 transcription factors that are enriched for binding to transcription sites around the 662 genes for cortex (x-axis) and cerebellum (y-axis). The 3 transcription factors that are labelled are lower expressed in cortex and higher expressed in cerebellum. For each transcription factor, n = 2,683 samples for Cortex and n = 492 samples for cerebellum.

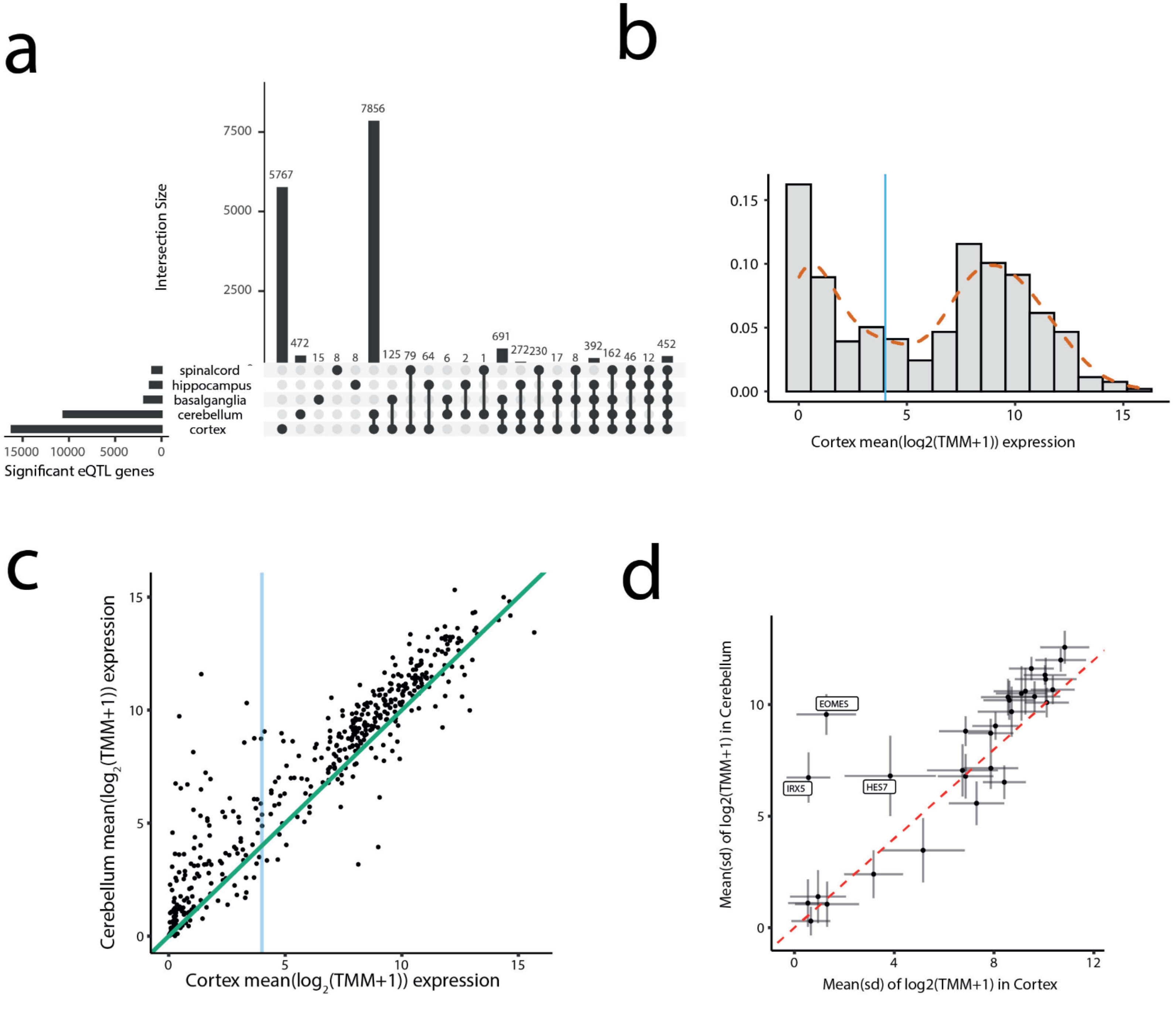

Supplementary Figure 14 - Correlation of effect sizes between Cortex primary eQTLs and GTEx eQTLs

The replication between primary *cis*-eQTLs of Cortex-EUR (discovery) with all the GTEx tissues (replication). The x-axis is the number of eQTLs that is significant in both discovery and replication, and the y-axis is the Rb.

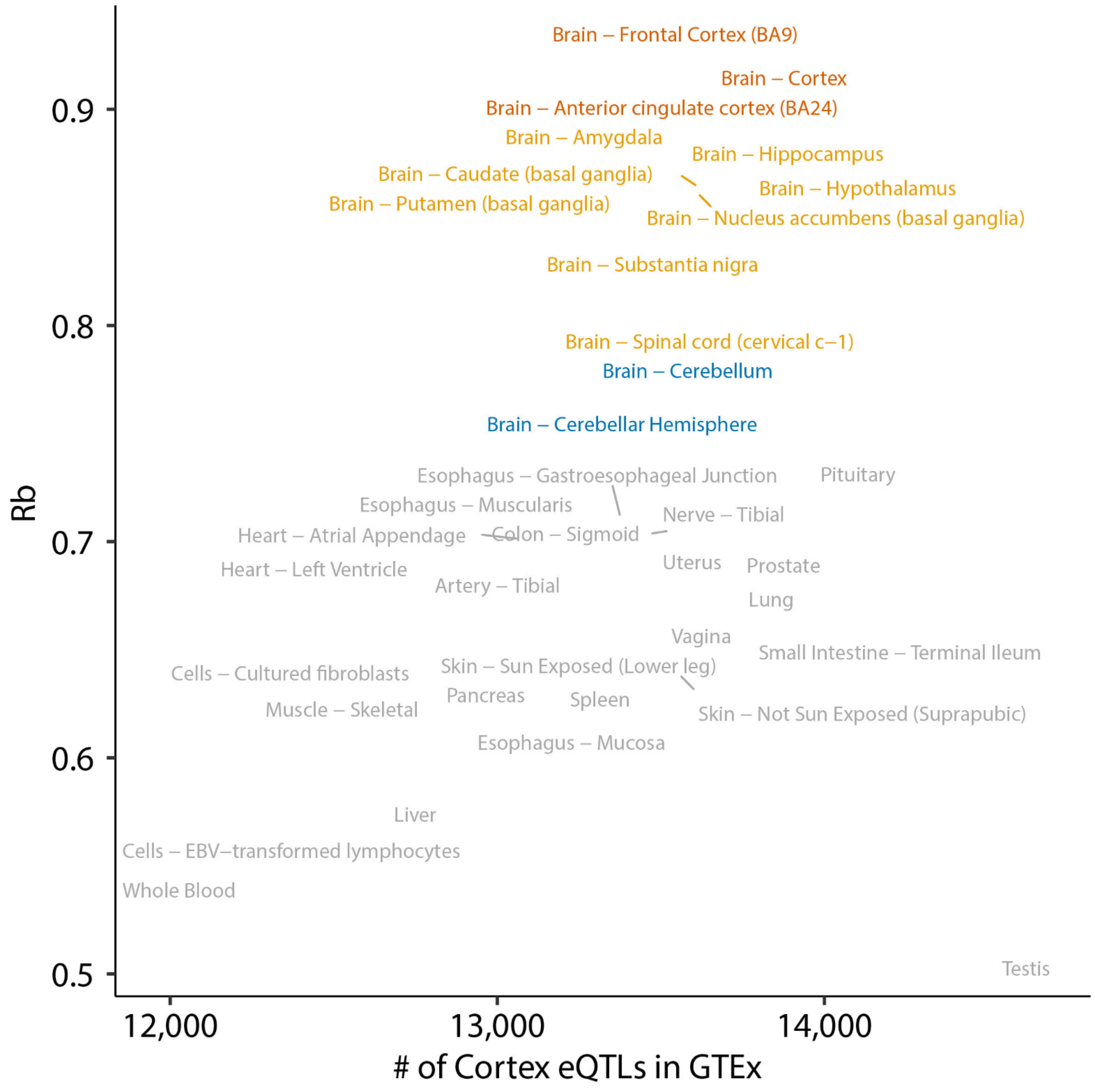

Supplementary Figure 15

Comparison of meta-analysis Z-scores for eQTLs detected in the different MetaBrain datasets (x-axis), and eQTLgen (y-axis). P-value calculated using two-sided chi²-test.

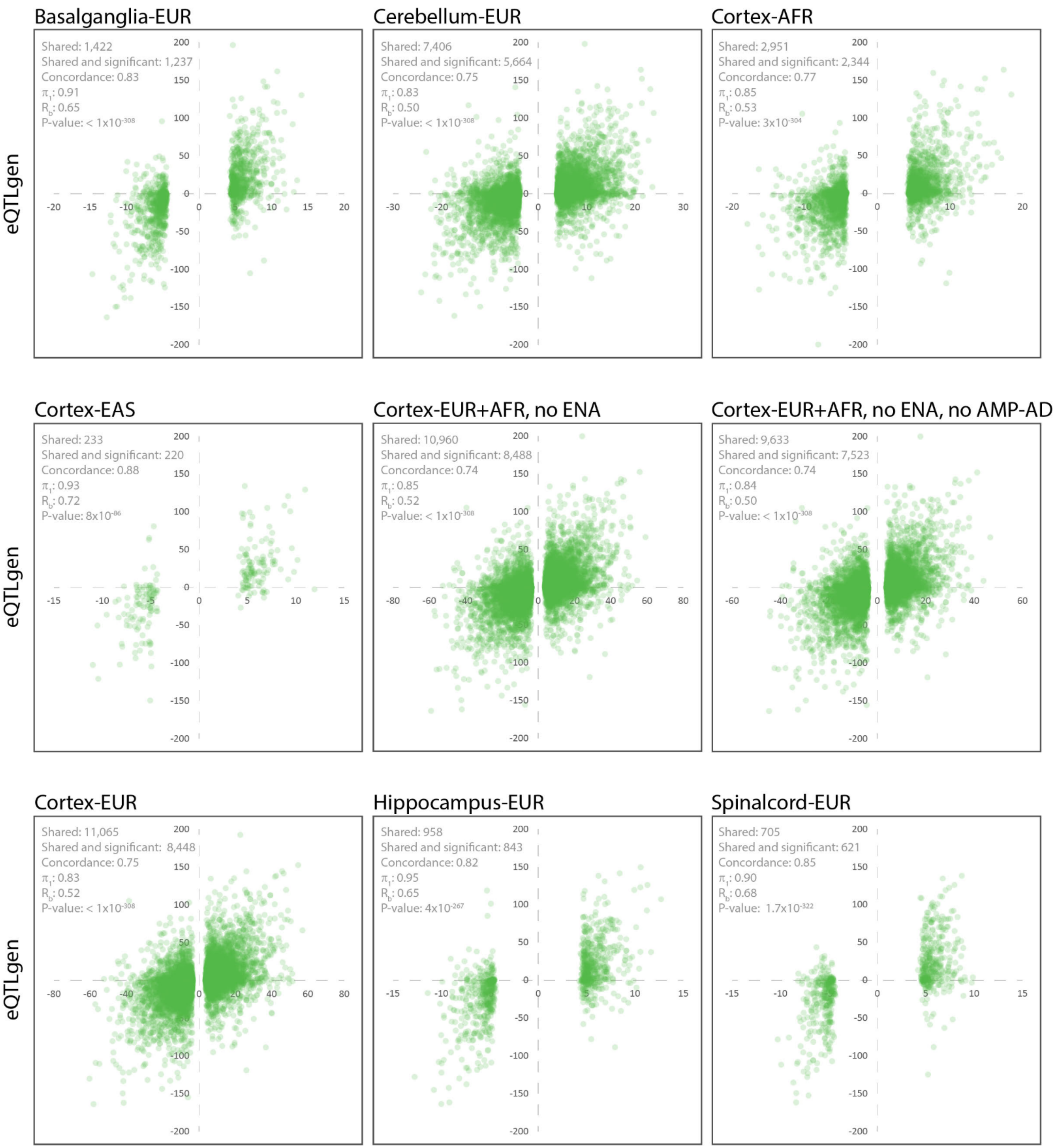

Supplementary Figure 16 - Distributions of predicted cell proportions

Boxplots show median (line in box), interquartile range (25th and 75th percentile, box), and minimum and maximum value (whiskers). Violin plots show distribution of the data. (a) Predicted cell type proportions in *MetaBrain* cortex European using the PsychENCODE reference profile. Developmental cell types were discarded in the prediction of these cell types. The value above each violin denotes the average cell fraction in percentages over all samples. (b) Aggregated cell type proportions in *MetaBrain* Cortex-EUR. Subtypes of cells for excitatory neurons, inhibitory neurons and oligodendrocytes (OPC and oligodendrocytes) are summed together. The value above each violin denotes the average cell fraction in percentages over all samples. For both (a) and (b) n = 2,683 samples for all cell types.

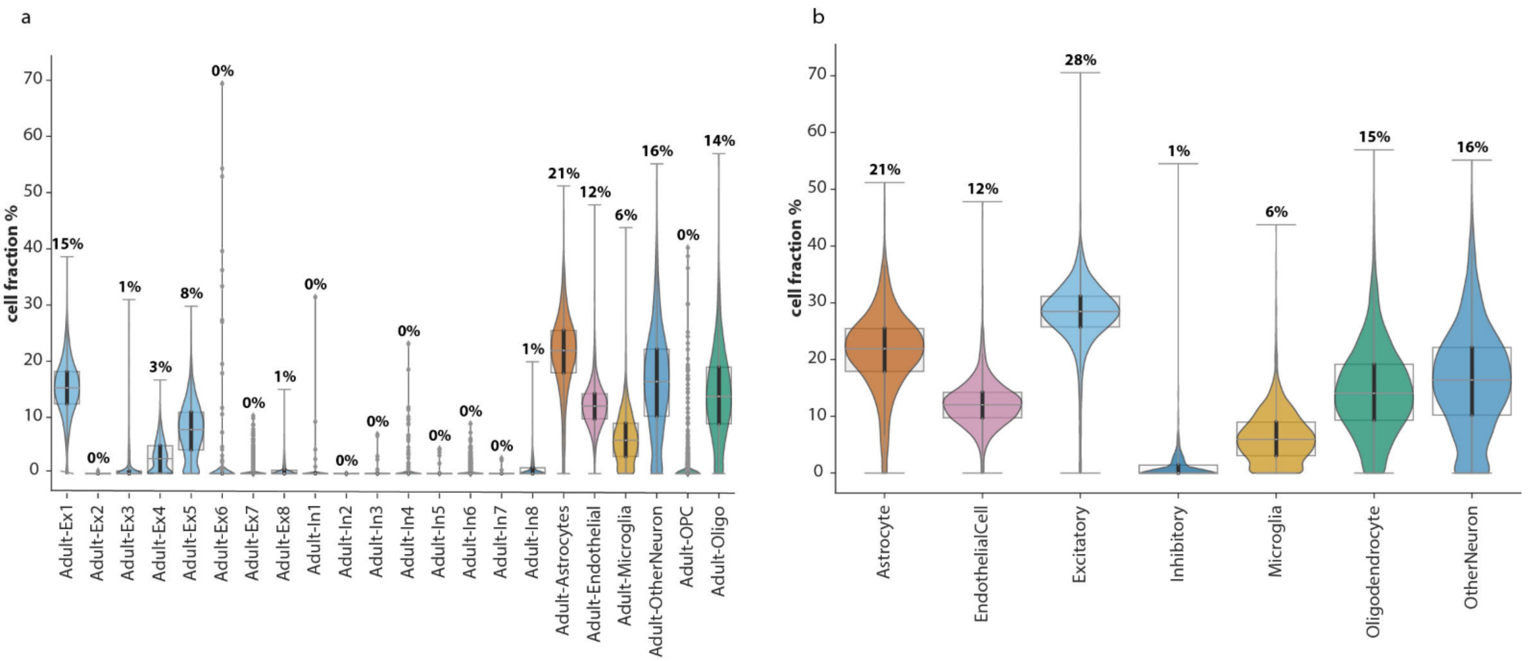

Supplementary Figure 17 - Replication of Cortex-EUR ieQTLs in Cortex-AFR

Each figure in this plot represents a comparison between Cortex-EUR (x-axis) and Cortex-AFR (y-axis). Each dot represents one *cis*-eQTL, and the legend shows the sample size, Pearson correlation coefficient, the AC, and, if applicable, the Rb and π1 statistics. Each column is a comparison between equivalent cell types in both datasets. Each row illustrates a different filtering on which eQTLs are shown. The values denote the log interaction beta from Decon-QTL. (a) All overlapping (i)eQTLs (b) (i)eQTLs filtered on being significant in Cortex-EUR (c) (i)eQTLs filtered on being significant in each respective dataset. If applicable, the 15 (i)eQTLs with the lowest p-value are labelled. Colored bands indicate 95% confidence interval around regression line.

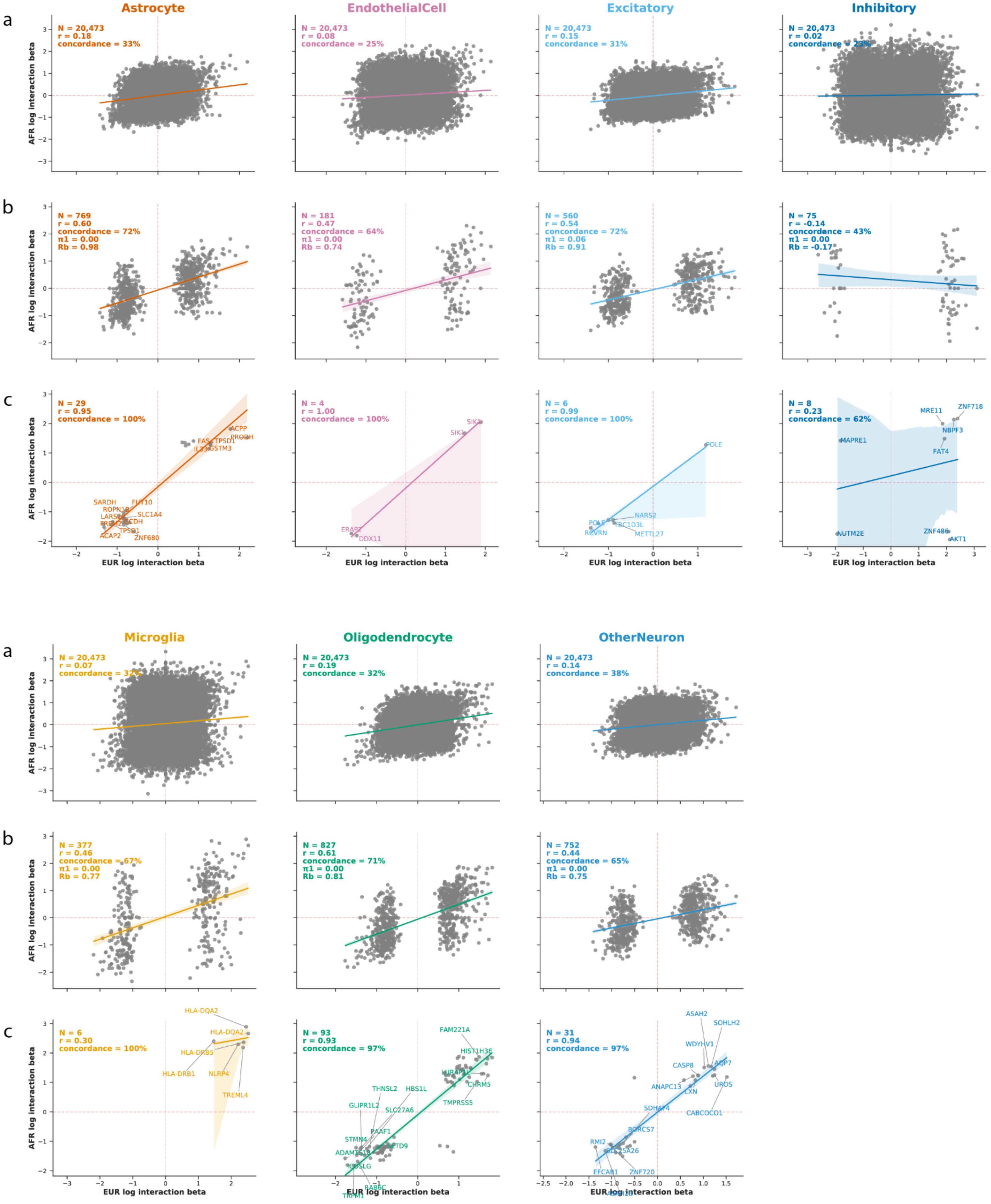

Supplementary Figure 18 - Replication of cortex *cis*-eQTLs in snRNA-seq data from ROSMAP

Each figure in this plot represents a comparison between bulk RNA-seq (x-axis) and ROSMAP single-nucleus RNA-seq (y-axis). Each dot represents one cis-eQTL, and the legend shows the sample size, Pearson correlation coefficient, the AC, and, if applicable, the Rb and π1 statistics. Each column is a comparison between equivalent cell types in both datasets. Each row illustrates a different filtering on which eQTLs are shown. The x-axis always denotes the log interaction beta from Decon-QTL, the y-axis always denotes the log beta of the eQTL effect in the single-nucleus dataset. (a) All overlapping (i)eQTLs (b) (i)eQTLs filtered on being significant in MetaBrain Cortex-EUR (c) ieQTLs filtered on being significant in each respective dataset. If applicable, the 15 ieQTLs with the lowest p-value are labelled. Colored bands indicate 95% confidence interval around regression line.

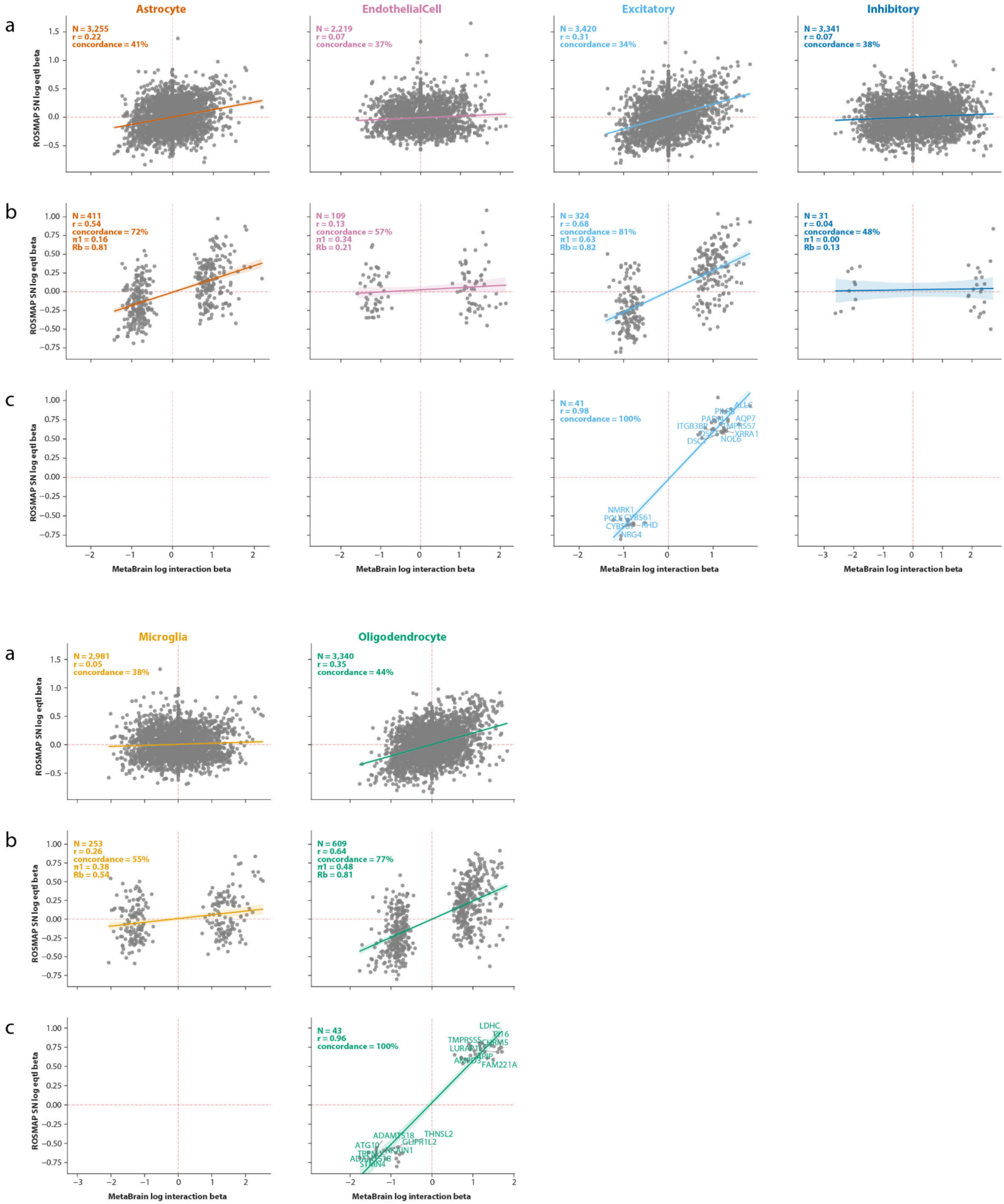

Supplementary Figure 19 - Replication of cortex *cis*-eQTLs in snRNA-seq data from Bryois *et al.*

Each figure in this plot represents a comparison between bulk RNA-seq (x-axis) and Bryois *et al.* single-nucleus RNA-seq (y-axis). Each dot represents one *cis*-eQTL, and the legend shows the sample size, Pearson correlation coefficient, the AC, and, if applicable, the Rb and π1 statistics. Each column is a comparison between equivalent cell types in both datasets. Each row illustrates a different filtering on which eQTLs are shown. The x-axis always denotes the log interaction beta from Decon-QTL, the y-axis always denotes the log beta of the eQTL effect in the single-nucleus dataset. (a) All overlapping (i)eQTLs (b) (i)eQTLs filtered on being significant in MetaBrain Cortex-EUR (c) (i)eQTLs filtered on being significant in each respective dataset. If applicable, the 15 (i)eQTLs with the lowest p-value are labelled.

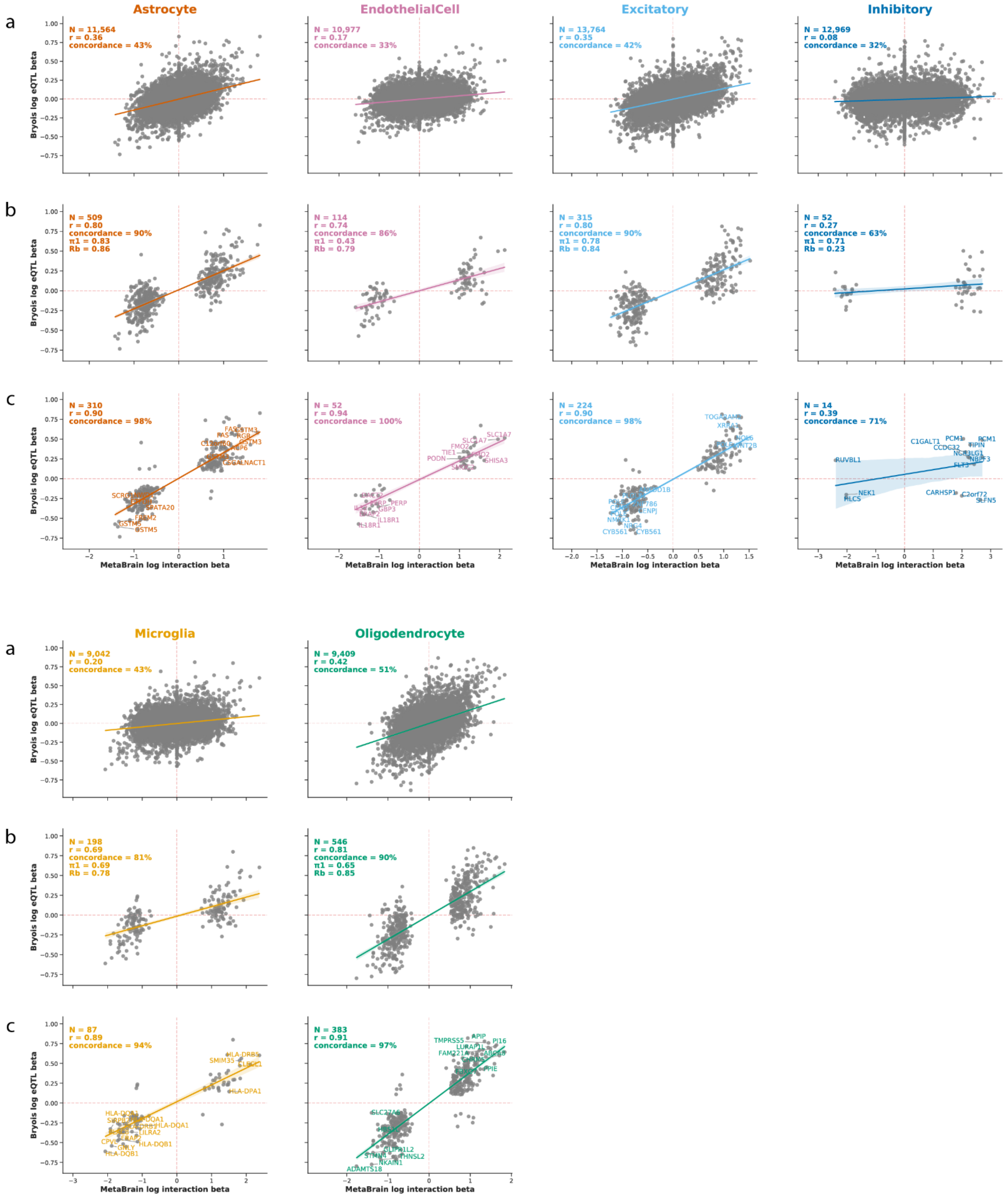

Supplementary Figure 20 - Replication of cortex *cis*-eQTLs in snRNA-seq data from Bryois *et al.*

Replication of cell type interaction eQTLs for *NKAIN1* (a), *STMN4* (b), *AMPD3* (c), *FAM221A* (d), *CD82* (e), and *CD38* (f). First column: Violinplot of the eQTL effect in Cortex-EUR bulk RNA-seq. The x-axis shows the genotype alleles, the y-axis shows the gene expression in TMM counts, the colors indicate the SNP genotype, with yellow being the minor allele. Boxplots show median (line in box), interquartile range (25th and 75th percentile, box), and minimum and maximum value (whiskers). Violin plots show distribution of the data. Second column: Cell type interacting eQTL effect in Cortex-EUR bulk RNA-seq. The x-axis shows the estimated cell type proportion, the y-axis shows the gene expression in TMM counts, each dot represents a sample, and the colors indicate the SNP genotype, with yellow being the minor allele. Values under the alleles are Pearson correlation coefficients. Third and fourth column: Forest plot of the eQTL betas with effect direction relative to the minor allele when replicating the eQTL effect in ROSMAP single-nucleus data (third column; n=38) and Bryois et al. 2021 (fourth column; n = 196). Dots indicate eQTL beta, error bars indicate ±1 standard error. Each row denotes a cell type specific dataset: astrocytes (AST), endothelial cells (END), excitatory neurons (EX), inhibitory neurons (IN), microglia (MIC), oligodendrocyte precursor cells (OPC), oligodendrocytes (OLI), pericytes (PER) and endothelial cells (END). Cell types highlighted in bold reflect the equivalent to the cell type used in the interaction eQTL.

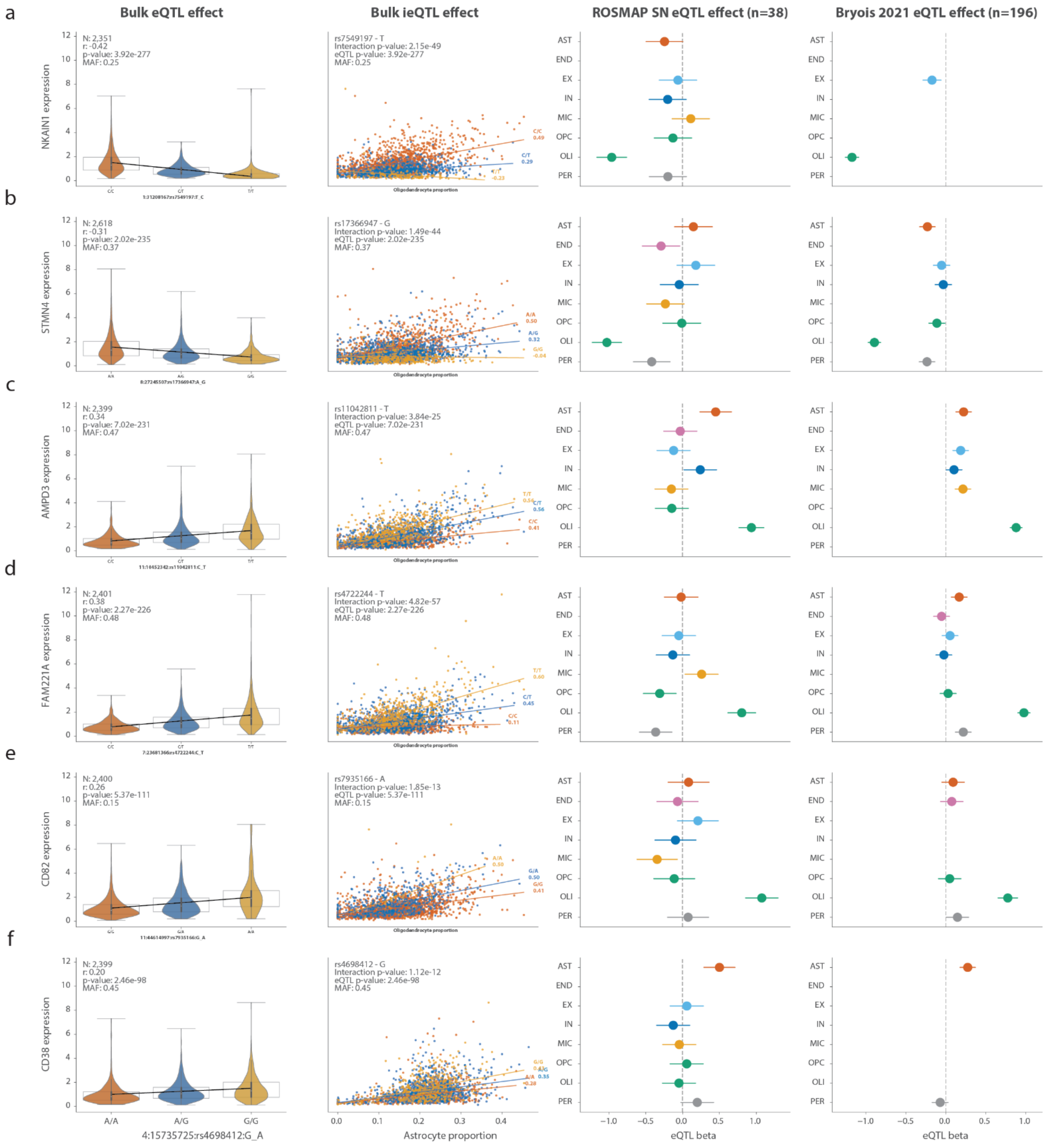

Supplementary Figure 21 - Mendelian Randomization summary

Each plot is for a different trait (Intelligence, Intracranial volume, Putamen volume, Years of schooling, Alzheimer's disease, Amyotrophic Lateral Sclerosis, Depression (broad), Frontotemporal Dementia, Parkinson's disease, Bipolar disorder, Generalized epilepsy, juvenile myoclonic epilepsy, multiple sclerosis and schizophrenia). For each SNP the effect allele (EA) is given, the eQTL beta of the EA on the given gene (left forest plot), the odds ratio (disease traits) or beta (quantitative traits) of the EA on the phenotype (right forest plot), and the Wald ratio p-value of the mendelian randomization analysis.

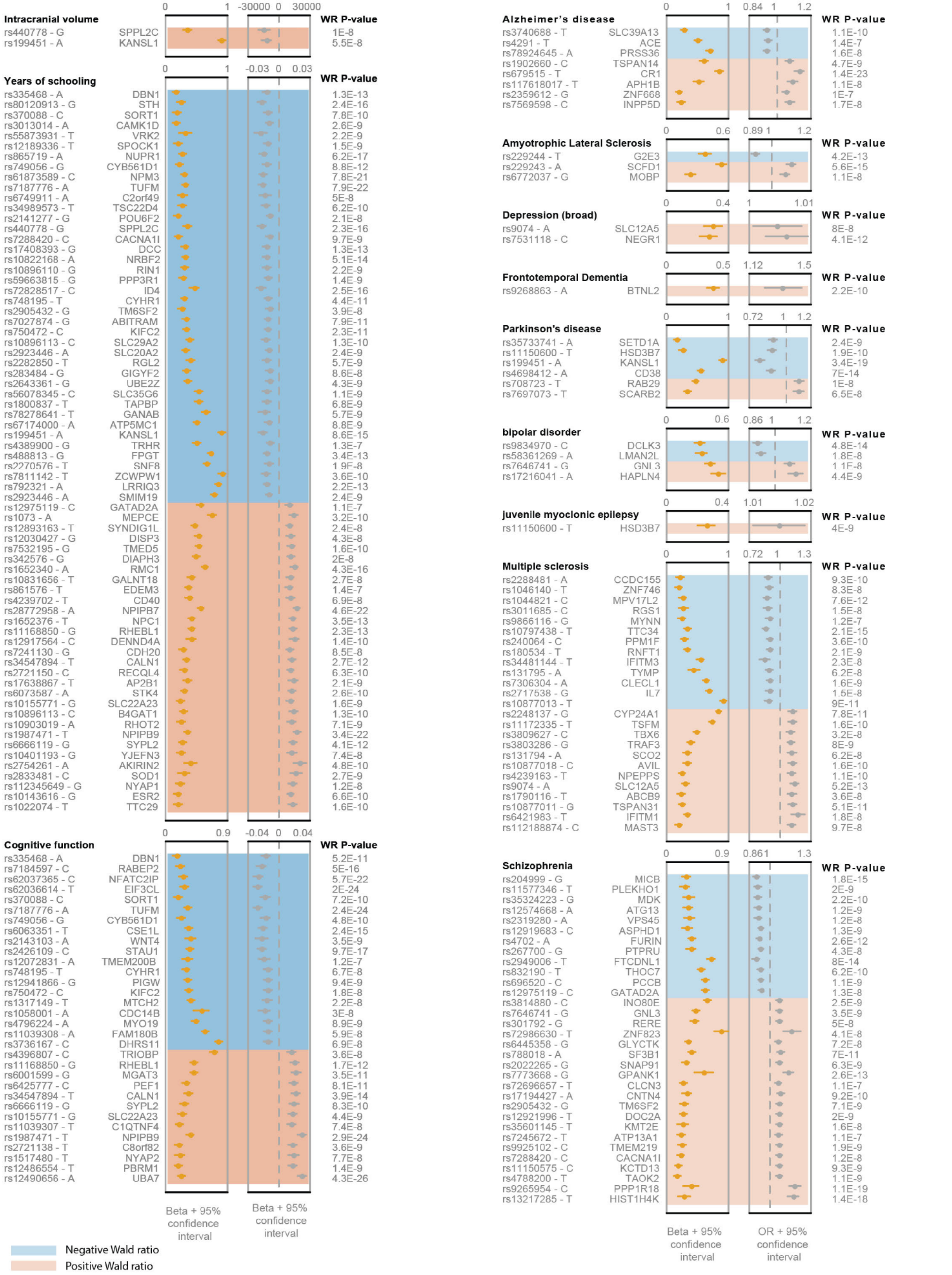

Supplementary Figure 22 - Colocalization regional plots for nine significant MR findings in Cortex-EUR that were replicated in eQTLGen with allelic discordance

Regional plots were made for nine MR findings (*DBN1* for intelligence and years of schooling, *ZNF746* and *AVIL* for multiple sclerosis, *SCFD1* for ALS, *KCTD13* for SCZ, *GATAD2A* for SCZ and years of schooling, and *ZCWPW1* for years of schooling) in Cortex-EUR (top), eQTLGen (middle) and outcome GWAS (bottom) to show colocalization. These nine findings all passed Bonferroni threshold ($p<1.43x10^{-7}$) in Cortex-EUR, with eQTL effects replicated in eQTLGen ($p<0.05$), showed colocalization for both Cortex-EUR and eQTLGen but opposite directions of effect.

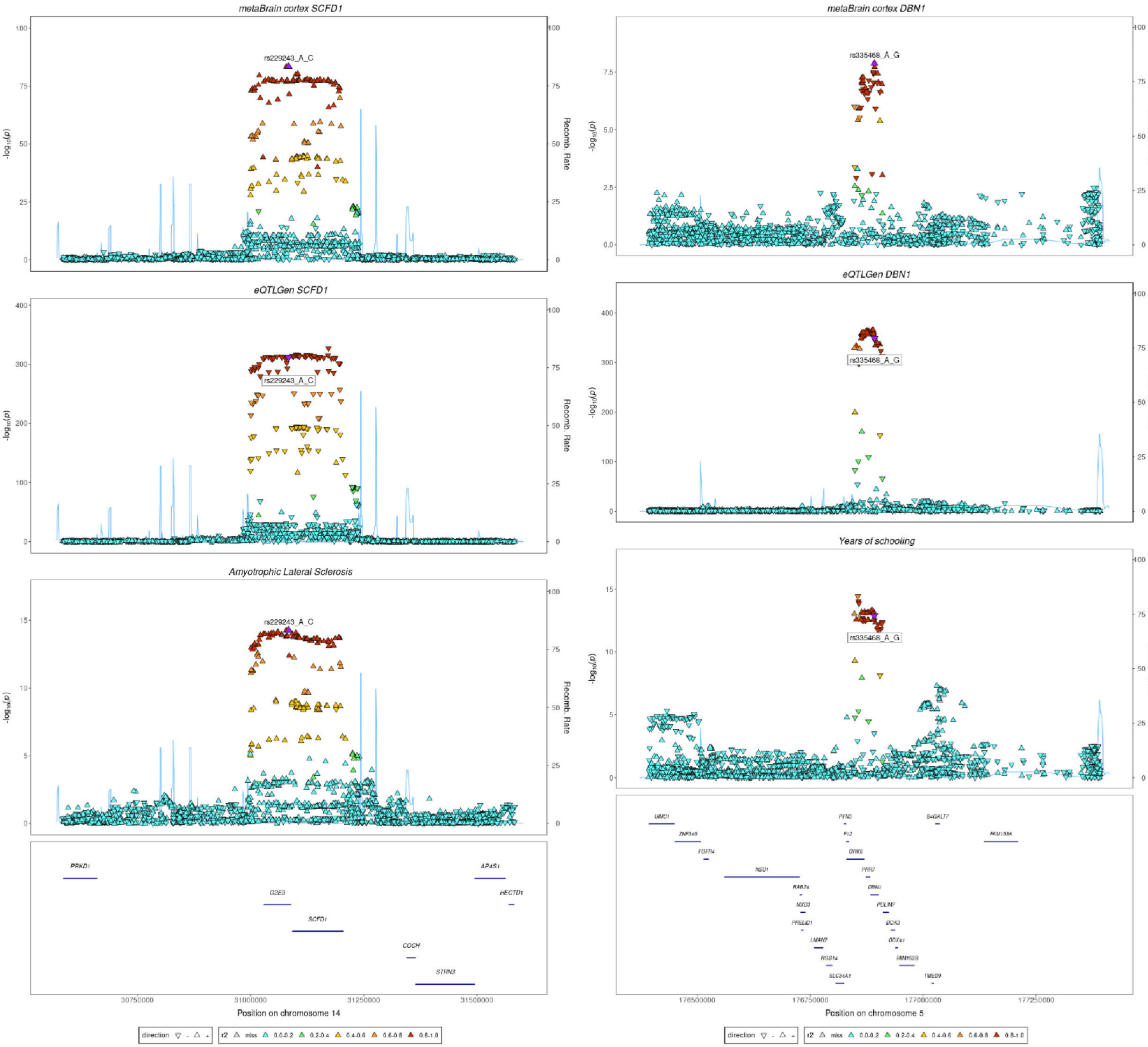

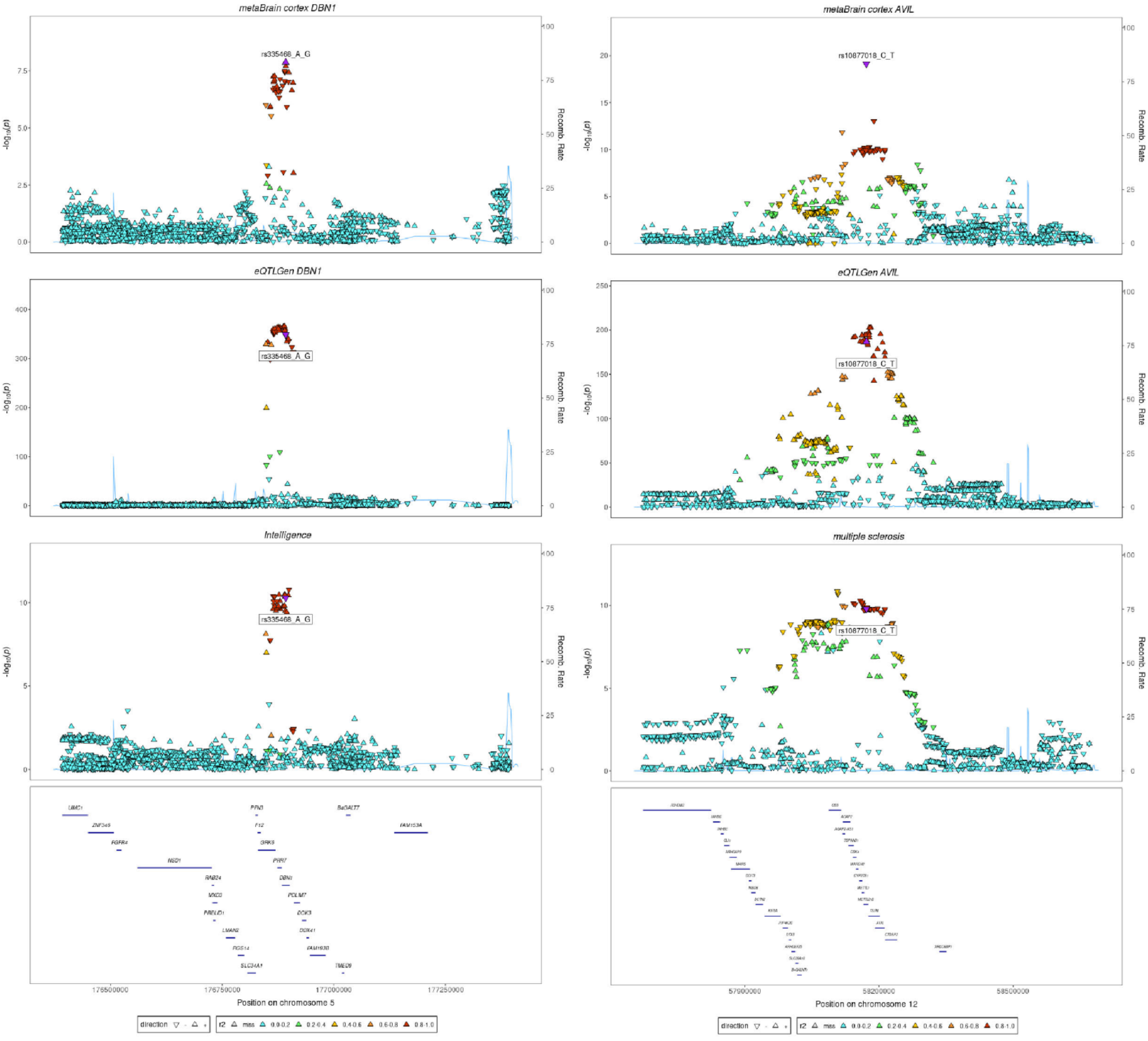

metaBrain cortex DBN1
rs335468_A_G
eQTLGen DBN1
Intelligence
-log10(p)
Recomb. Rate
Position on chromosome 5
metaBrain cortex AVIL
rs10877018_C_T
eQTLGen AVIL
multiple sclerosis
Position on chromosome 12
direction
r2
miss
0.0-0.2
0.2-0.4
0.4-0.6
0.6-0.8
0.8-1.0

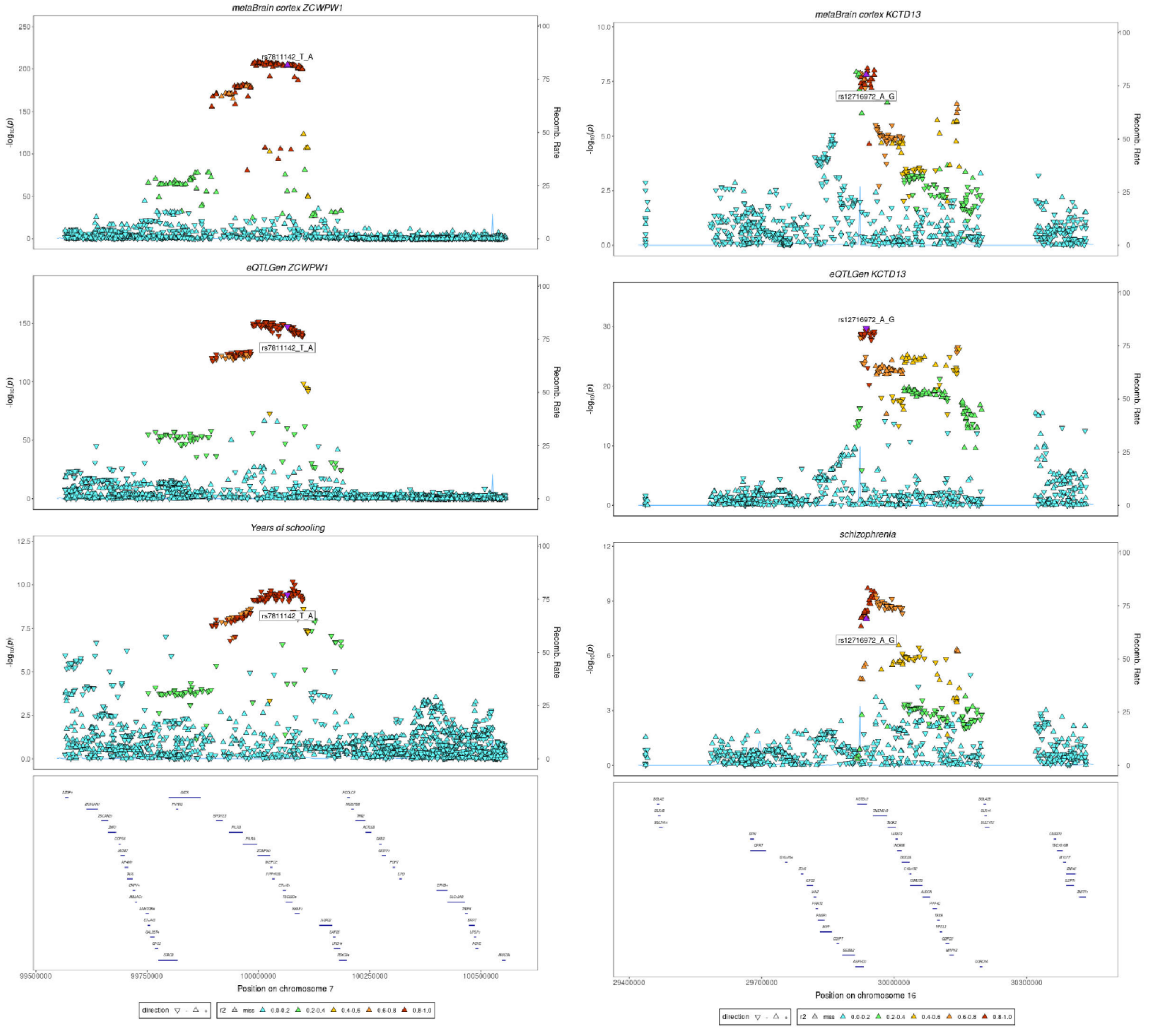

metaBrain cortex ZCWPW1
rs7811142_T_A
eQTLGen ZCWPW1
Years of schooling
-log10(p)
Recomb. Rate
Position on chromosome 7
metaBrain cortex KCTD13
rs12716972_A_G
eQTLGen KCTD13
schizophrenia
Position on chromosome 16
direction - +
r2 miss 0.0-0.2 0.2-0.4 0.4-0.6 0.6-0.8 0.8-1.0

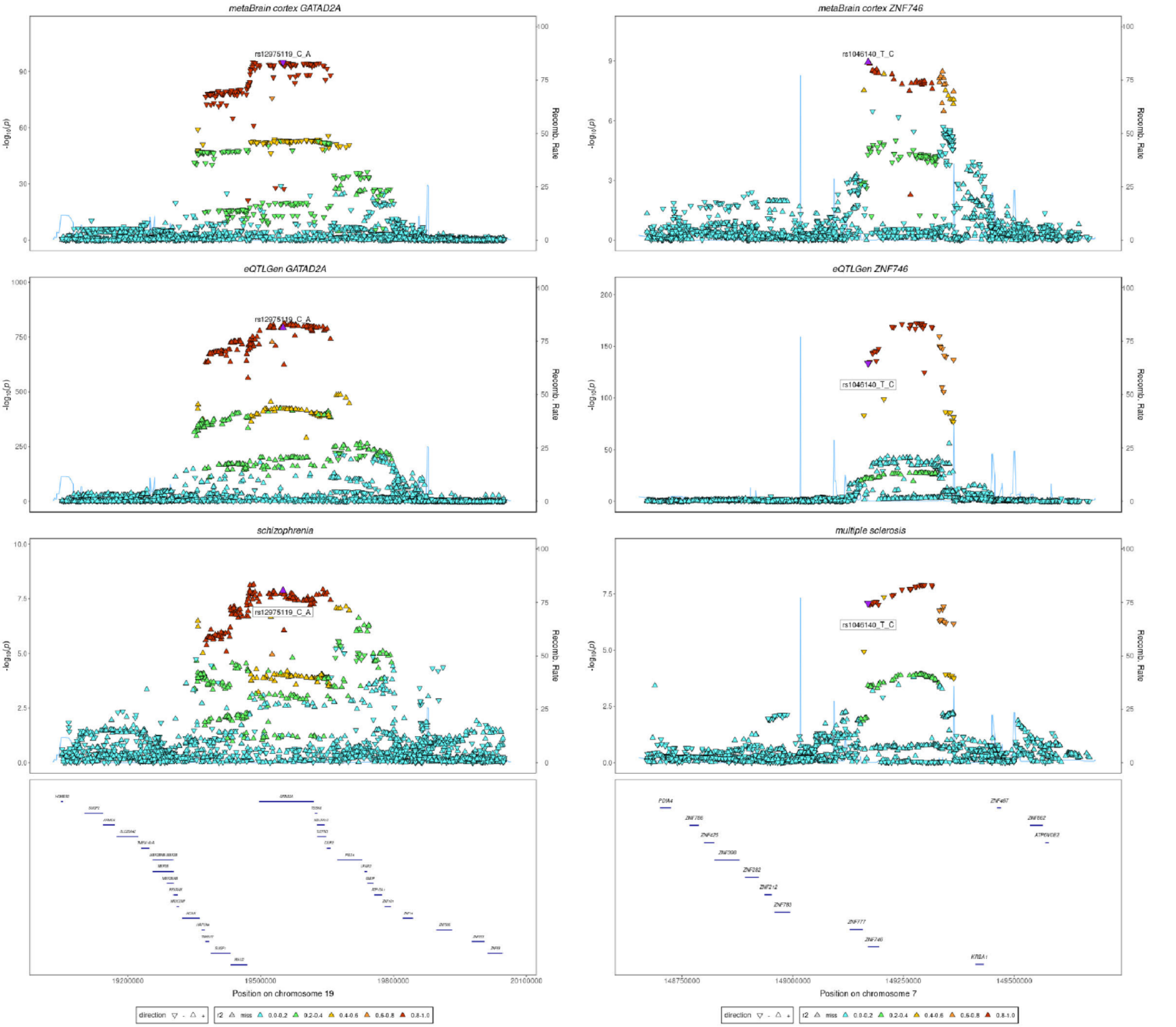
metaBrain cortex GATAD2A
rs12975119_C_A
metaBrain cortex ZNF746
rs1046140_T_C
eQTLGen GATAD2A
rs12975119_C_A
eQTLGen ZNF746
rs1046140_T_C
schizophrenia
rs12975119_C_A
multiple sclerosis
rs1046140_T_C
-log10(p)
Recomb. Rate
PDIA4
ZNF786
ZNF425
ZNF398
ZNF282
ZNF212
ZNF783
ZNF777
ZNF746
KRBA1
ZNF467
ZNF862
ATP6V0E2
19200000
19500000
19800000
20100000
Position on chromosome 19
148750000
149000000
149250000
149500000
Position on chromosome 7
direction - +
r2 miss 0.0-0.2 0.2-0.4 0.4-0.6 0.6-0.8 0.8-1.0

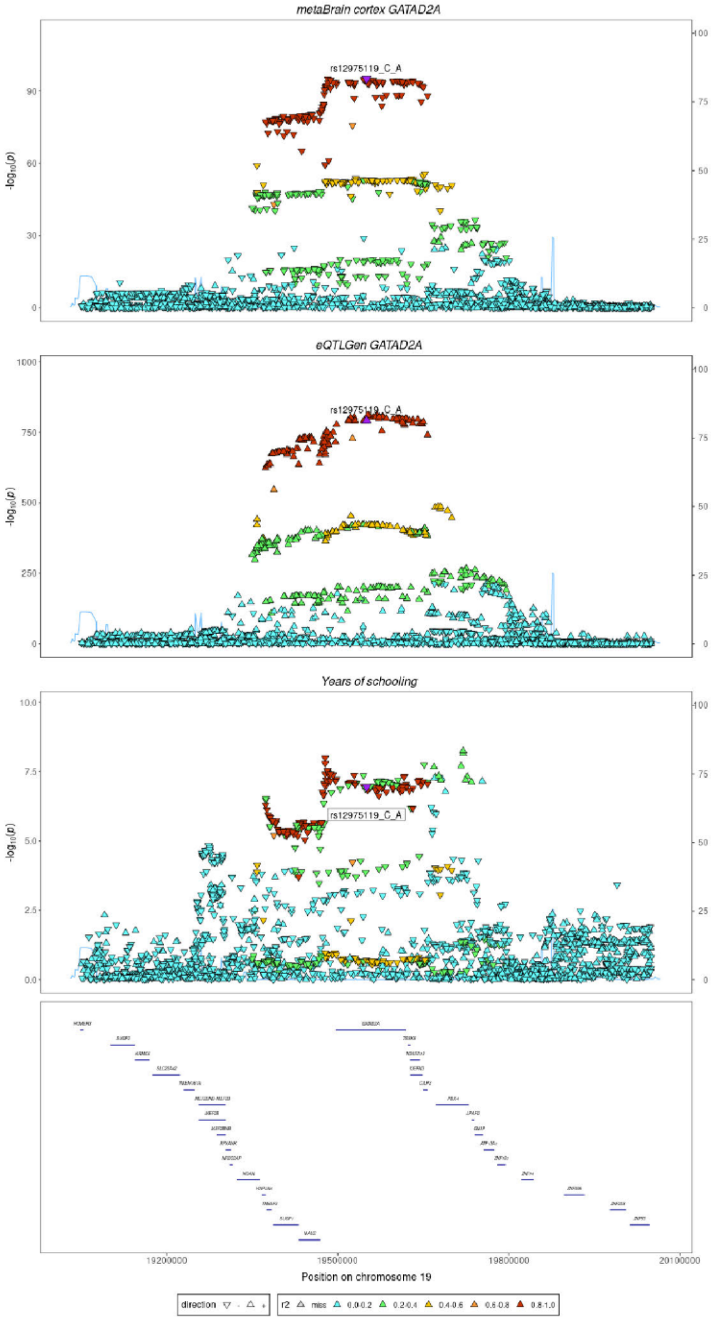

metaBrain cortex GATAD2A
eQTLGen GATAD2A
Years of schooling
rs12975119_C_A
-log10(p)
Recomb. Rate
Position on chromosome 19
19200000
19500000
19800000
20100000
direction
r2
miss
0.0-0.2
0.2-0.4
0.4-0.6
0.6-0.8
0.8-1.0

Supplementary Figure 23 - Scatterplots comparing MR effects for multiple sclerosis derived using instruments from the *MetaBrain* versus eQTLGen studies

The top panel shows the Wald ratio comparison on the same gene but with the different SNP instruments selected by each study (matching on the top Wald ratio finding if gene instrumented with multiple SNPs in the study) and the bottom panel the Wald ratio comparison between *MetaBrain* instruments and eQTLGen matching on both the same gene and SNP instrument. Genes which showed opposite direction of Wald ratio effect between *MetaBrain* and eQTLGen are colored in red and the genes with the same direction in blue.

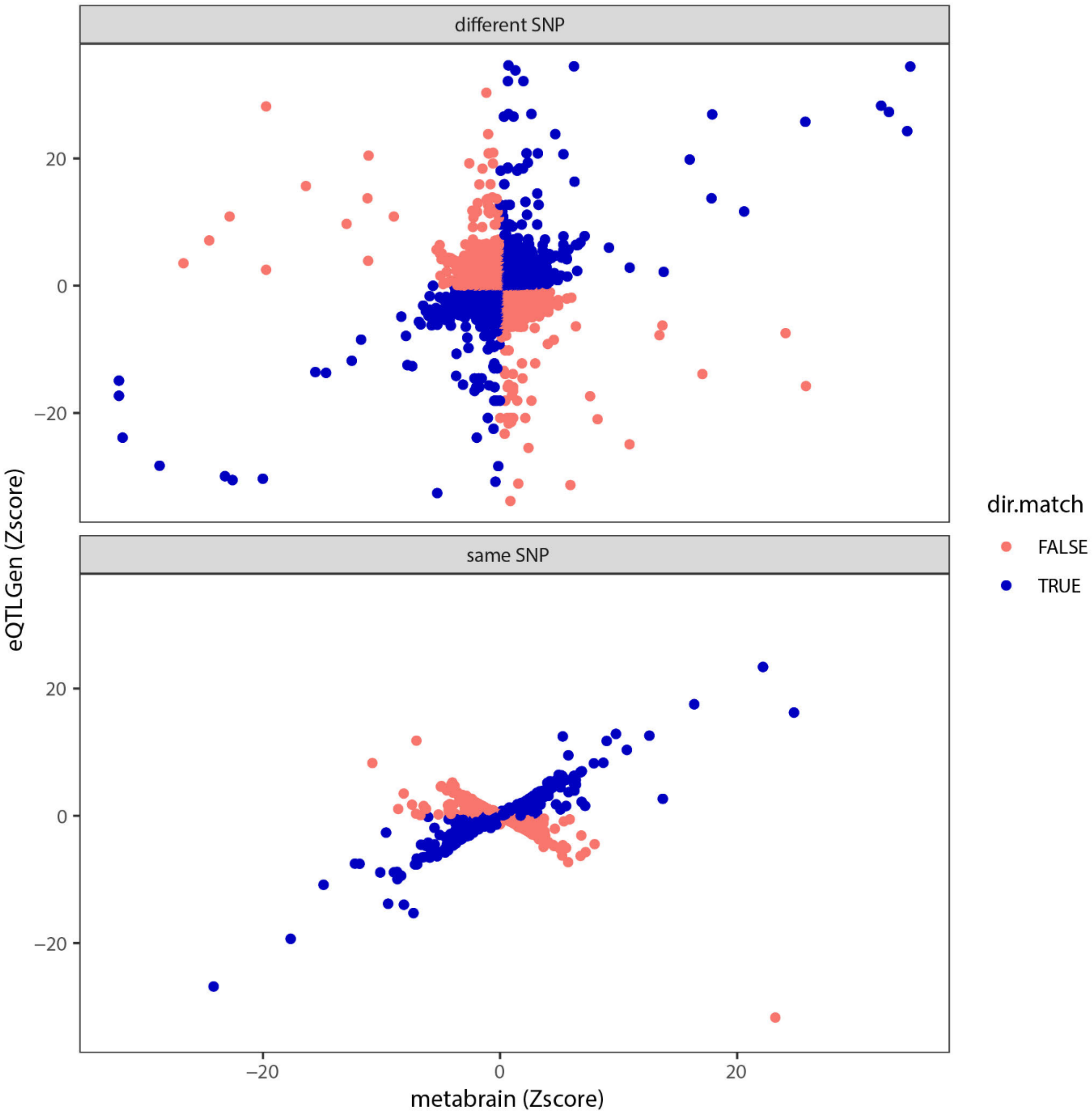

Supplementary Figure 24 - Colocalization regional plots for three suggestive MR findings for multiple sclerosis that showed opposite directions of effect between Cortex-EUR and eQTLGen

Regional plots were made for three suggestive MR findings for MS (*AVIL, KCTD13, ZNF746*) in Cortex-EUR (top), eQTLGen (middle) and MS GWAS (bottom) to show colocalization. All three were suggestive signals in Cortex-EUR as well as eQTLGen ($p<5x10^{-5}$), showed colocalization for both Cortex-EUR and eQTLGen but opposite directions of effect.

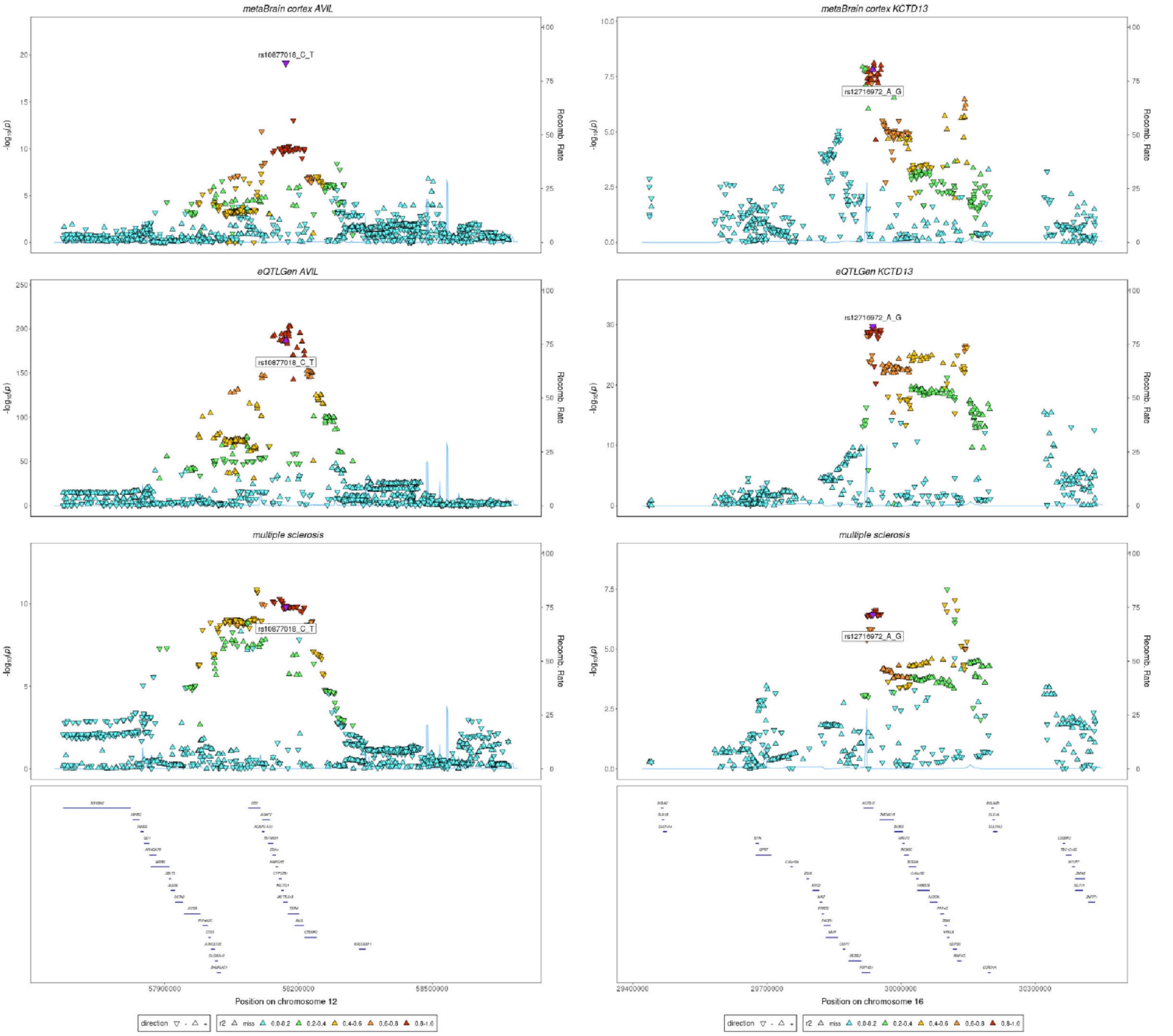

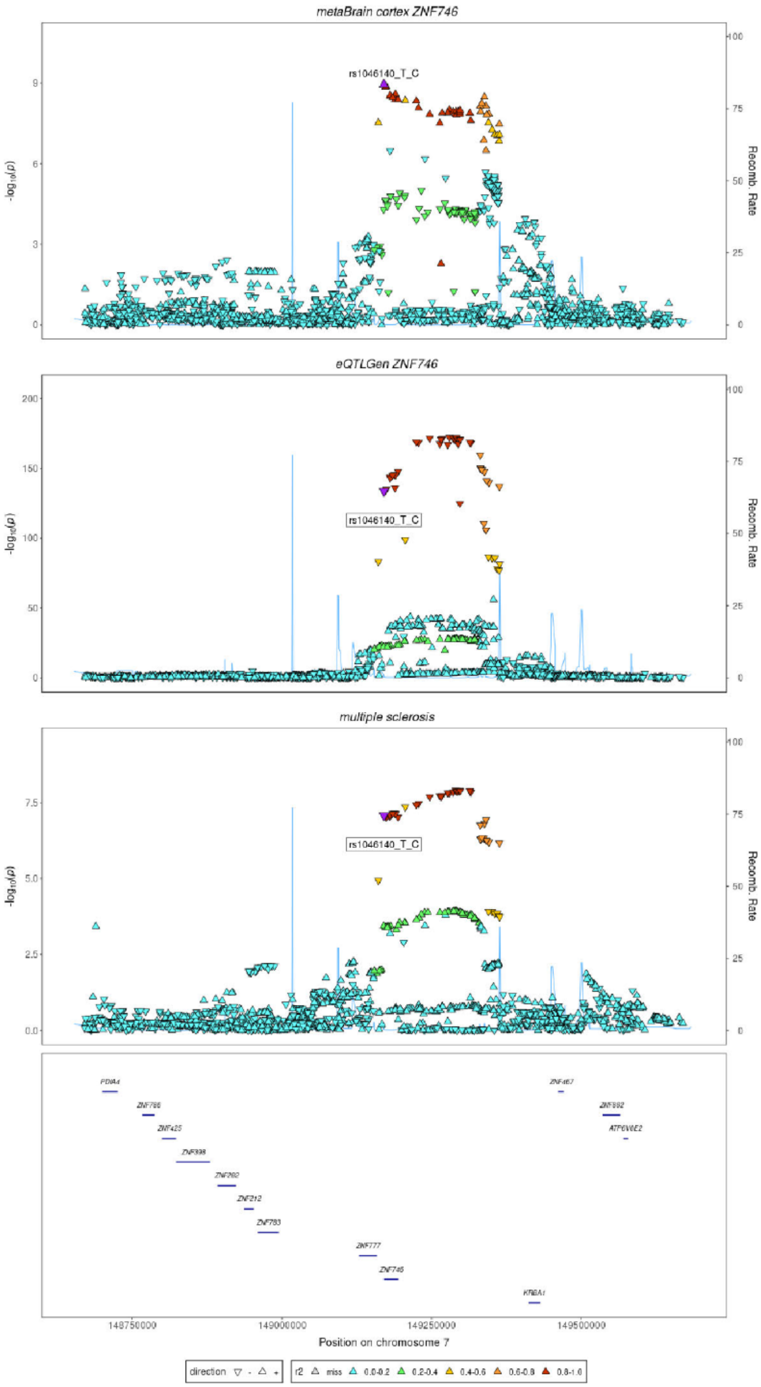
metaBrain cortex ZNF746
rs1046140_T_C
eQTLGen ZNF746
rs1046140_T_C
multiple sclerosis
rs1046140_T_C
-log10(p)
Recomb. Rate
PDIA4
ZNF786
ZNF425
ZNF398
ZNF282
ZNF212
ZNF783
ZNF777
ZNF746
KRBA1
ZNF467
ZNF862
ATP6V0E2
148750000
149000000
149250000
149500000
Position on chromosome 7
direction
r2
miss
0.0-0.2
0.2-0.4
0.4-0.6
0.6-0.8
0.8-1.0

Supplementary Figure 25 - Expression of genes with opposite effects with eQTLGen

Log10 of median expression of brain and blood tissue samples in GTEx for 5 multiple sclerosis genes for which there are no significant eQTLgen instruments, but for which we did find significant MR and colocalization in *MetaBrain*.

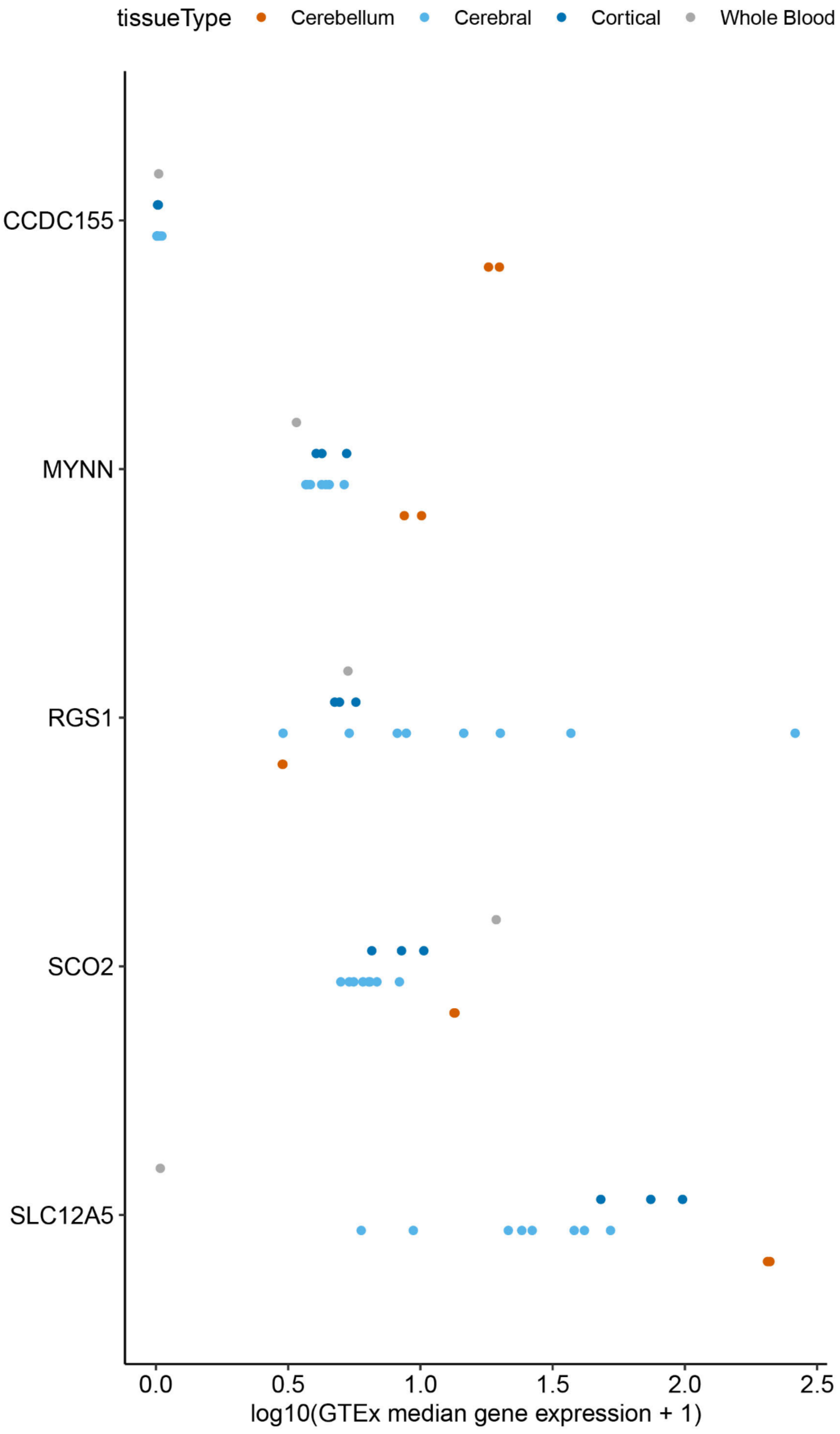

Supplementary Figure 26 - Heterogeneity of *trans*-eQTLs in Cortex

Measured by I². A high amount of heterogeneity is observed when the gene expression data is not corrected for PCs, which disappears when AMP-AD datasets are excluded. Similarly, heterogeneity decreases when the gene expression data is corrected for PCs.

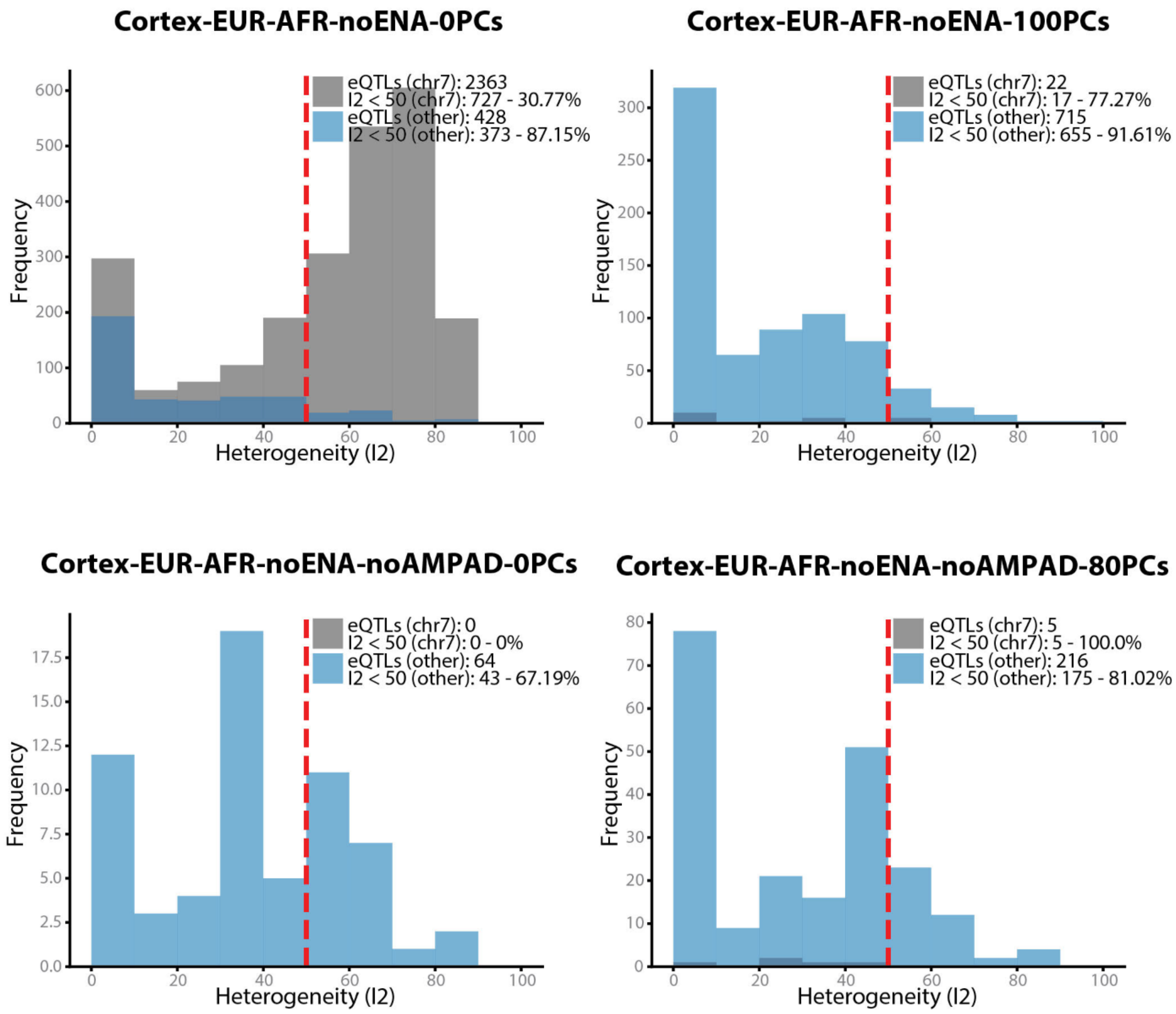

Supplementary Figure 27 - *Trans*-eQTLs in the 7p21.3 locus

(a) Dot plot showing the location of the significant *trans*-eQTLs with and without PCA correction. (b) Overview of the *TMEM106B* locus. (c) QQ-plots after correcting for 0 PCs or 100 PCs of all *trans*-eQTL associations between genes and SNPs in the 7p21.3 locus. SNPs were limited to those that had a significant *trans*-eQTL association when no PCs were removed. No cross-mapping correction applied and created using estlambda2 function in R, using permuted p-values to determine expected χ^2. (d) Correlation of *trans*-eQTL gene expression levels with *TMEM106B* and *THSD7A*. (e) Comparison of effect sizes between datasets of rs1990622-A for nearby genes *TMEM106B* and *THSD7A*, *trans*-eQTL gene *CALB2*, and predicted excitatory neuron proportion. Dots indicate beta, error bars indicate 95% confidence interval. Violin plots indicate distributions of predicted excitatory neuron cell counts per dataset with dots being the median value. Meta-analysis n=2,683. (f) Pearson correlations between *trans*-eQTL Z-scores and correlation of *trans*-eQTL genes with excitatory neuron proportions. (g) Correlation between *trans*-eQTL Z-scores observed in Alzheimer's disease cases versus non-neurological controls in the AMP-AD datasets. (h) Interaction between *CALB2 trans*-eQTL for rs1990622 and Alzheimer's disease status, using (left) all samples in Cortex-EUR with a Alzheimer's disease or non-neurological control label, (middle) limited to AMP-AD datasets, and (right) excluding AMP-AD. Boxplots show median (line in box), interquartile range (25th and 75th percentile, box), and minimum and maximum value (whiskers), excluding outliers (outliers are defined as less than Q1 – 1.5*(IQ3-IQ1) or greater than Q3 + 1.5*(IQ3-IQ1)). The x-axis shows the disease status, the y-axis shows the gene expression, each dot is a sample and is colored by the SNP genotype.

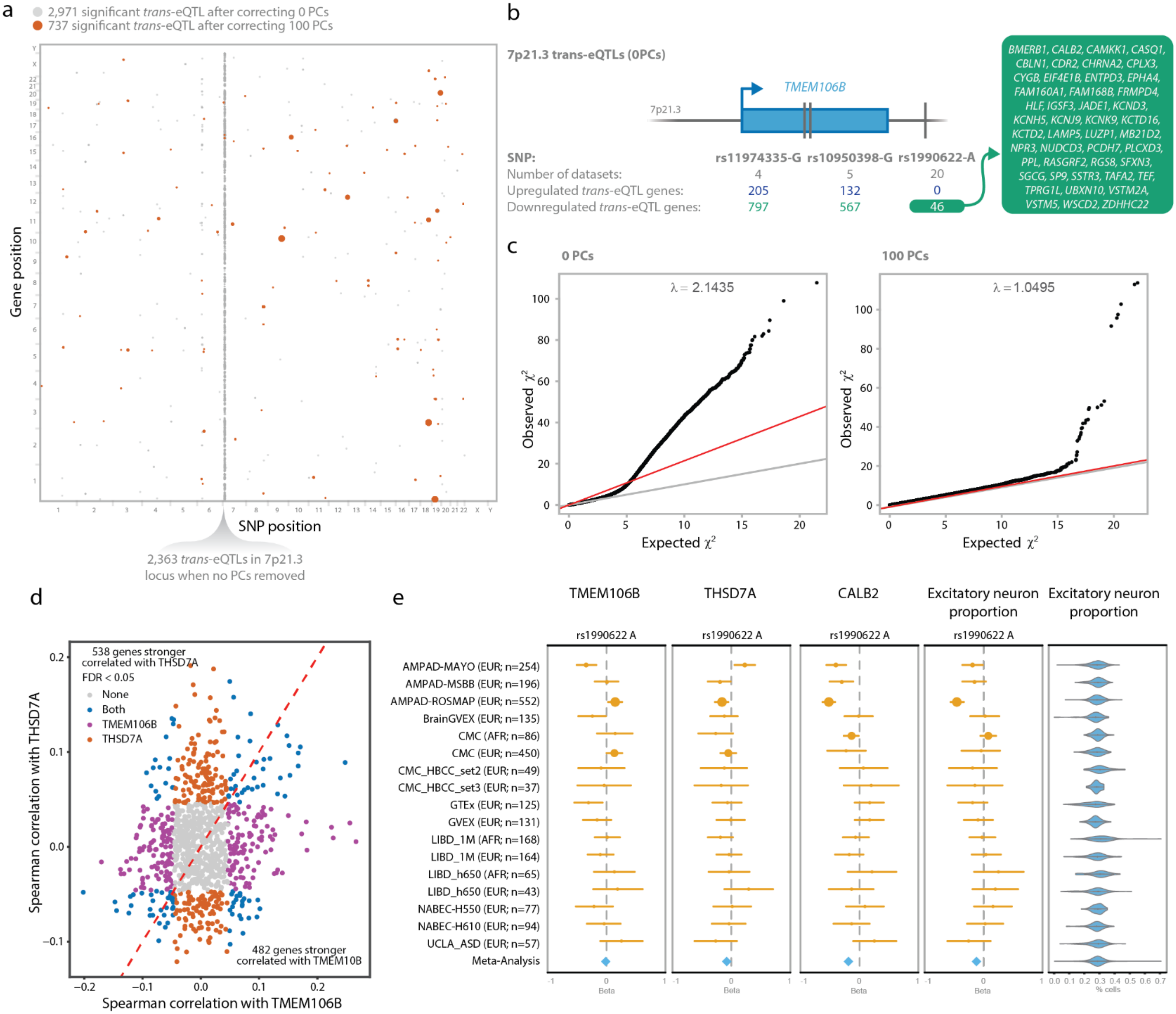

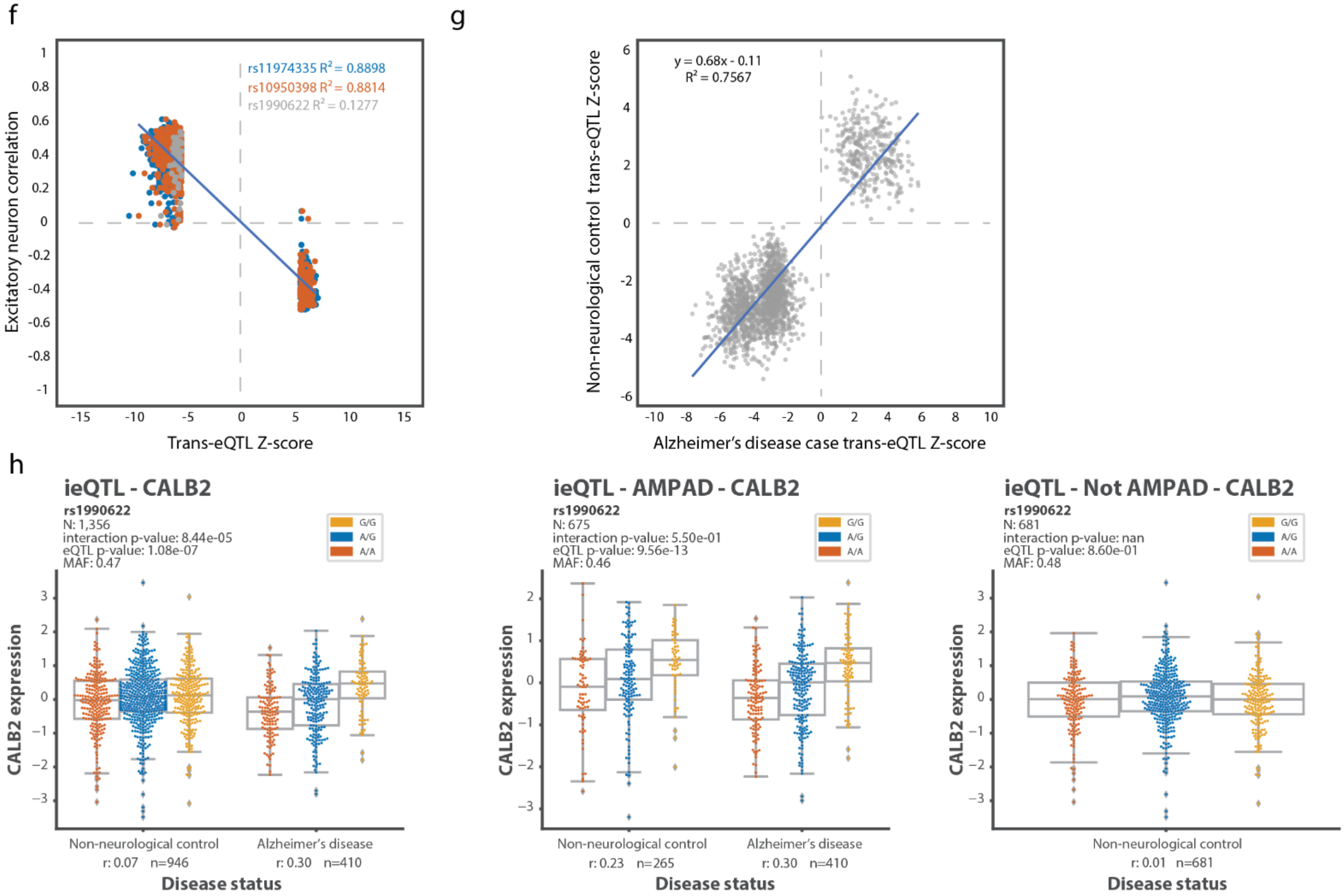

f
rs11974335 R² = 0.8898
rs10950398 R² = 0.8814
rs1990622 R² = 0.1277
Excitatory neuron correlation
Trans-eQTL Z-score
g
y = 0.68x - 0.11
R² = 0.7567
Non-neurological control trans-eQTL Z-score
Alzheimer's disease case trans-eQTL Z-score
h
ieQTL - CALB2
rs1990622
N: 1,356
interaction p-value: 8.44e-05
eQTL p-value: 1.08e-07
MAF: 0.47
G/G
A/G
A/A
CALB2 expression
Non-neurological control
r: 0.07 n=946
Alzheimer's disease
r: 0.30 n=410
Disease status
ieQTL - AMPAD - CALB2
rs1990622
N: 675
interaction p-value: 5.50e-01
eQTL p-value: 9.56e-13
MAF: 0.46
G/G
A/G
A/A
CALB2 expression
Non-neurological control
r: 0.23 n=265
Alzheimer's disease
r: 0.30 n=410
Disease status
ieQTL - Not AMPAD - CALB2
rs1990622
N: 681
interaction p-value: nan
eQTL p-value: 8.60e-01
MAF: 0.48
G/G
A/G
A/A
CALB2 expression
Non-neurological control
r: 0.01 n=681
Disease status

Supplementary Figure 28 - Forest plots for rs1990622 *trans*-eQTLs

Forest plots for each of the *trans*-eQTL genes associated with rs1990622. Each plot shows the *trans*-eQTL beta (dots) and 95% confidence interval (error bars) for each of the included datasets and the meta-analysis. Effect directions are relative to the A allele of rs1990622. Sizes of dots are relative to sample size of each dataset. *Trans*-eQTL effects are most pronounced in AMP-AD datasets. Meta analysis n=2,683.

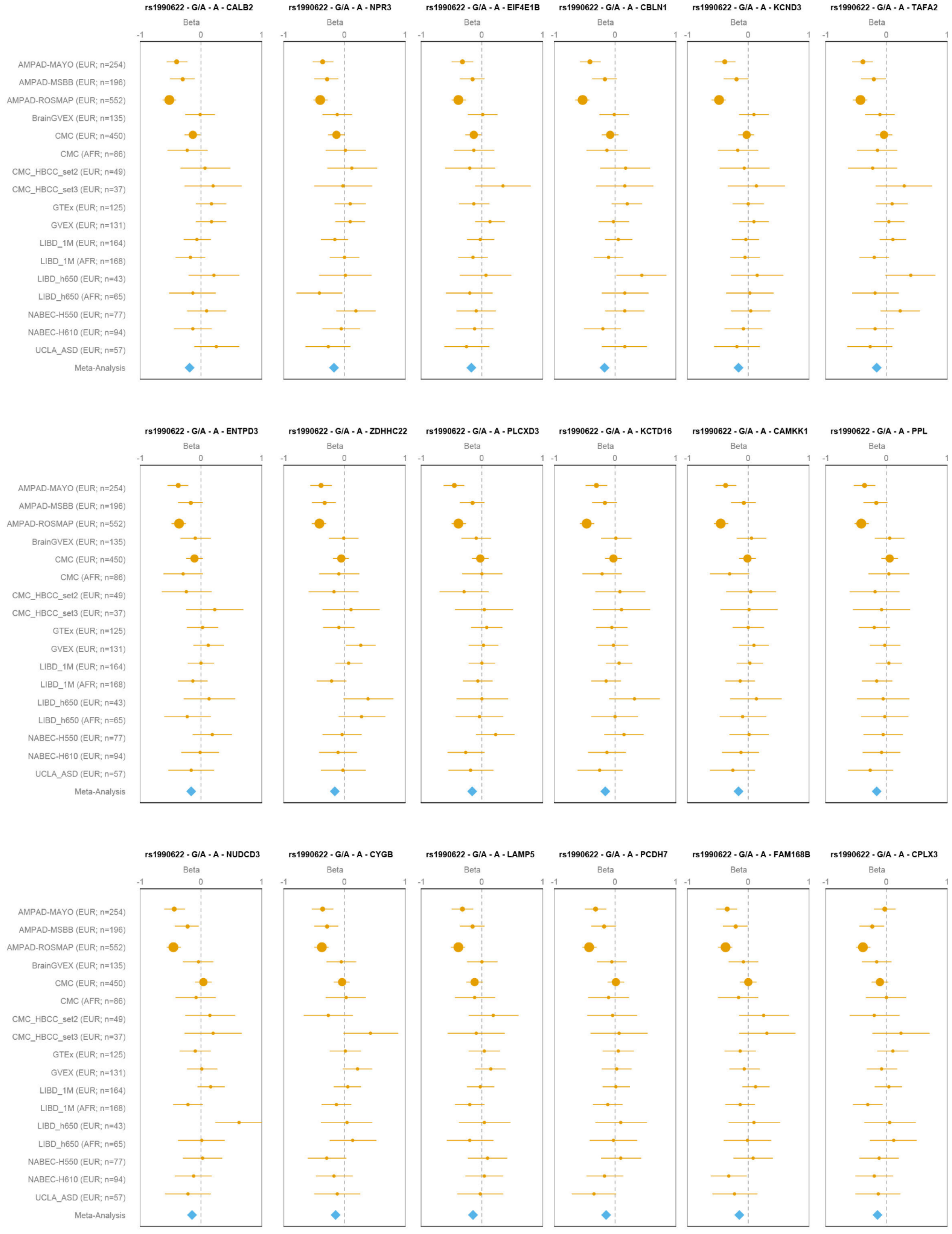

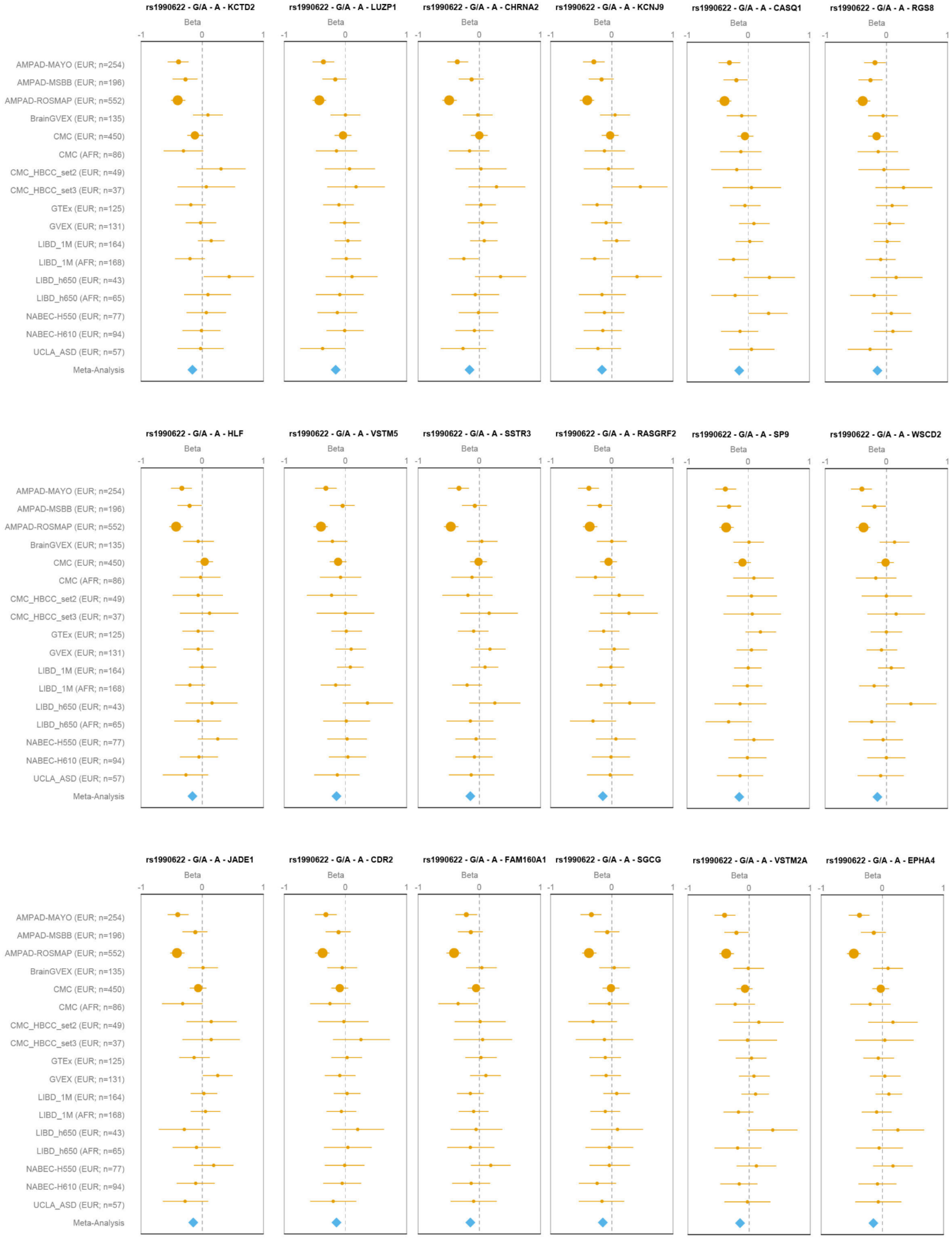

rs1990622 - G/A - A - KCTD2
rs1990622 - G/A - A - LUZP1
rs1990622 - G/A - A - CHRNA2
rs1990622 - G/A - A - KCNJ9
rs1990622 - G/A - A - CASQ1
rs1990622 - G/A - A - RGS8
rs1990622 - G/A - A - HLF
rs1990622 - G/A - A - VSTM5
rs1990622 - G/A - A - SSTR3
rs1990622 - G/A - A - RASGRF2
rs1990622 - G/A - A - SP9
rs1990622 - G/A - A - WSCD2
rs1990622 - G/A - A - JADE1
rs1990622 - G/A - A - CDR2
rs1990622 - G/A - A - FAM160A1
rs1990622 - G/A - A - SGCG
rs1990622 - G/A - A - VSTM2A
rs1990622 - G/A - A - EPHA4
Beta
-1
0
1
AMPAD-MAYO (EUR; n=254)
AMPAD-MSBB (EUR; n=196)
AMPAD-ROSMAP (EUR; n=552)
BrainGVEX (EUR; n=135)
CMC (EUR; n=450)
CMC (AFR; n=86)
CMC_HBCC_set2 (EUR; n=49)
CMC_HBCC_set3 (EUR; n=37)
GTEx (EUR; n=125)
GVEX (EUR; n=131)
LIBD_1M (EUR; n=164)
LIBD_1M (AFR; n=168)
LIBD_h650 (EUR; n=43)
LIBD_h650 (AFR; n=65)
NABEC-H550 (EUR; n=77)
NABEC-H610 (EUR; n=94)
UCLA_ASD (EUR; n=57)
Meta-Analysis

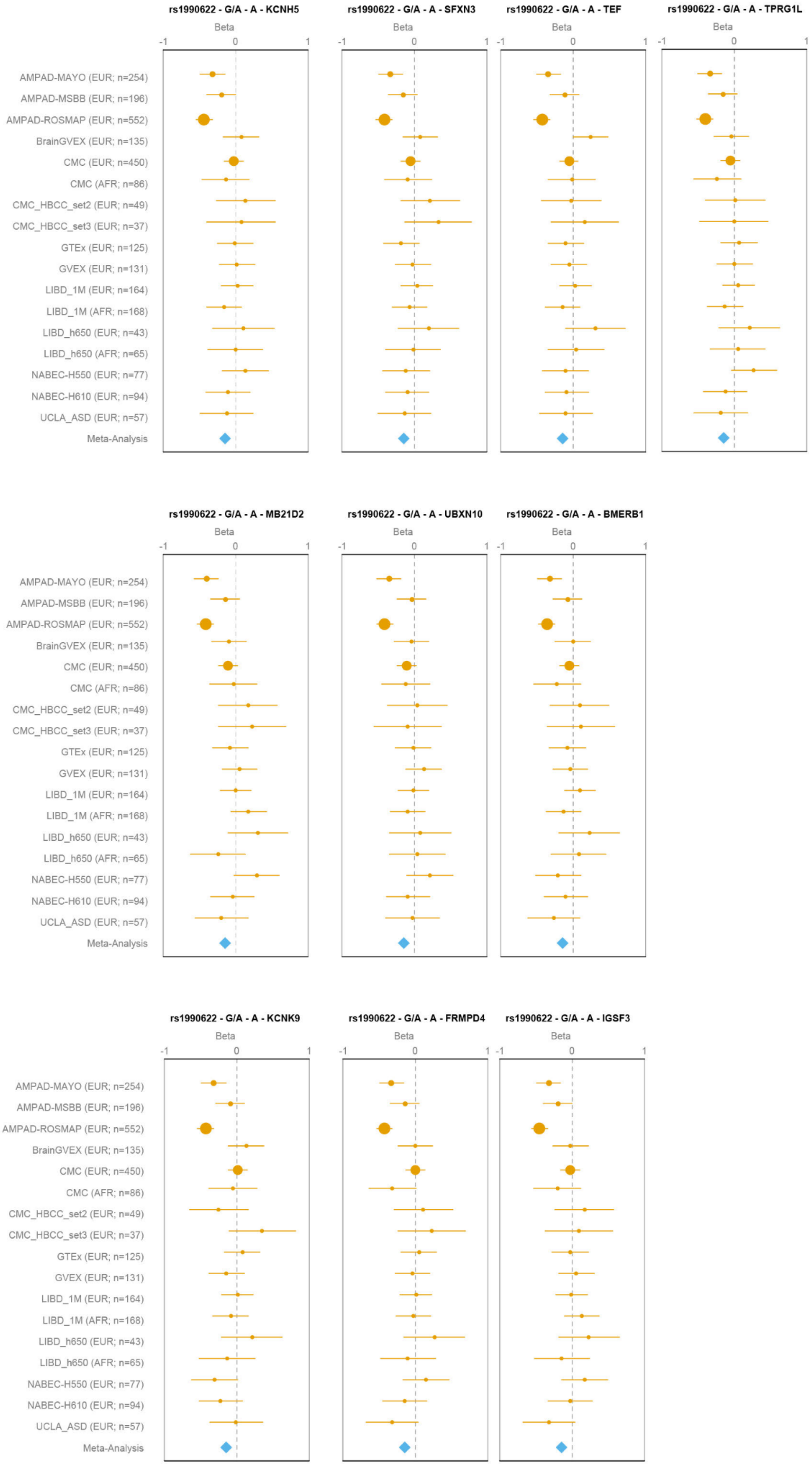

rs1990622 - G/A - A - KCNH5
rs1990622 - G/A - A - SFXN3
rs1990622 - G/A - A - TEF
rs1990622 - G/A - A - TPRG1L
Beta
-1
0
1
AMPAD-MAYO (EUR; n=254)
AMPAD-MSBB (EUR; n=196)
AMPAD-ROSMAP (EUR; n=552)
BrainGVEX (EUR; n=135)
CMC (EUR; n=450)
CMC (AFR; n=86)
CMC_HBCC_set2 (EUR; n=49)
CMC_HBCC_set3 (EUR; n=37)
GTEx (EUR; n=125)
GVEX (EUR; n=131)
LIBD_1M (EUR; n=164)
LIBD_1M (AFR; n=168)
LIBD_h650 (EUR; n=43)
LIBD_h650 (AFR; n=65)
NABEC-H550 (EUR; n=77)
NABEC-H610 (EUR; n=94)
UCLA_ASD (EUR; n=57)
Meta-Analysis
rs1990622 - G/A - A - MB21D2
rs1990622 - G/A - A - UBXN10
rs1990622 - G/A - A - BMERB1
Beta
-1
0
1
AMPAD-MAYO (EUR; n=254)
AMPAD-MSBB (EUR; n=196)
AMPAD-ROSMAP (EUR; n=552)
BrainGVEX (EUR; n=135)
CMC (EUR; n=450)
CMC (AFR; n=86)
CMC_HBCC_set2 (EUR; n=49)
CMC_HBCC_set3 (EUR; n=37)
GTEx (EUR; n=125)
GVEX (EUR; n=131)
LIBD_1M (EUR; n=164)
LIBD_1M (AFR; n=168)
LIBD_h650 (EUR; n=43)
LIBD_h650 (AFR; n=65)
NABEC-H550 (EUR; n=77)
NABEC-H610 (EUR; n=94)
UCLA_ASD (EUR; n=57)
Meta-Analysis
rs1990622 - G/A - A - KCNK9
rs1990622 - G/A - A - FRMPD4
rs1990622 - G/A - A - IGSF3
Beta
-1
0
1
AMPAD-MAYO (EUR; n=254)
AMPAD-MSBB (EUR; n=196)
AMPAD-ROSMAP (EUR; n=552)
BrainGVEX (EUR; n=135)
CMC (EUR; n=450)
CMC (AFR; n=86)
CMC_HBCC_set2 (EUR; n=49)
CMC_HBCC_set3 (EUR; n=37)
GTEx (EUR; n=125)
GVEX (EUR; n=131)
LIBD_1M (EUR; n=164)
LIBD_1M (AFR; n=168)
LIBD_h650 (EUR; n=43)
LIBD_h650 (AFR; n=65)
NABEC-H550 (EUR; n=77)
NABEC-H610 (EUR; n=94)
UCLA_ASD (EUR; n=57)
Meta-Analysis

Supplementary Figure 29 - Cell type proportions in Alzheimer's disease patients

Predicted cell count proportions for the AMP-AD samples that were used in the Cortex-EUR eQTL analysis for individuals with Alzheimer's disease and non-neurological controls. Each dot is the predicted cell proportion for one sample. Numbers under the box plots indicate the number of samples plotted. Values above the line are p-values from a two-sided t-test between groups. Boxplots show median (line in box), interquartile range (25th and 75th percentile, box), and minimum and maximum value (whiskers), excluding outliers (outliers are defined as less than Q1 – 1.5*(IQ3-IQ1) or greater than Q3 + 1.5*(IQ3-IQ1)).

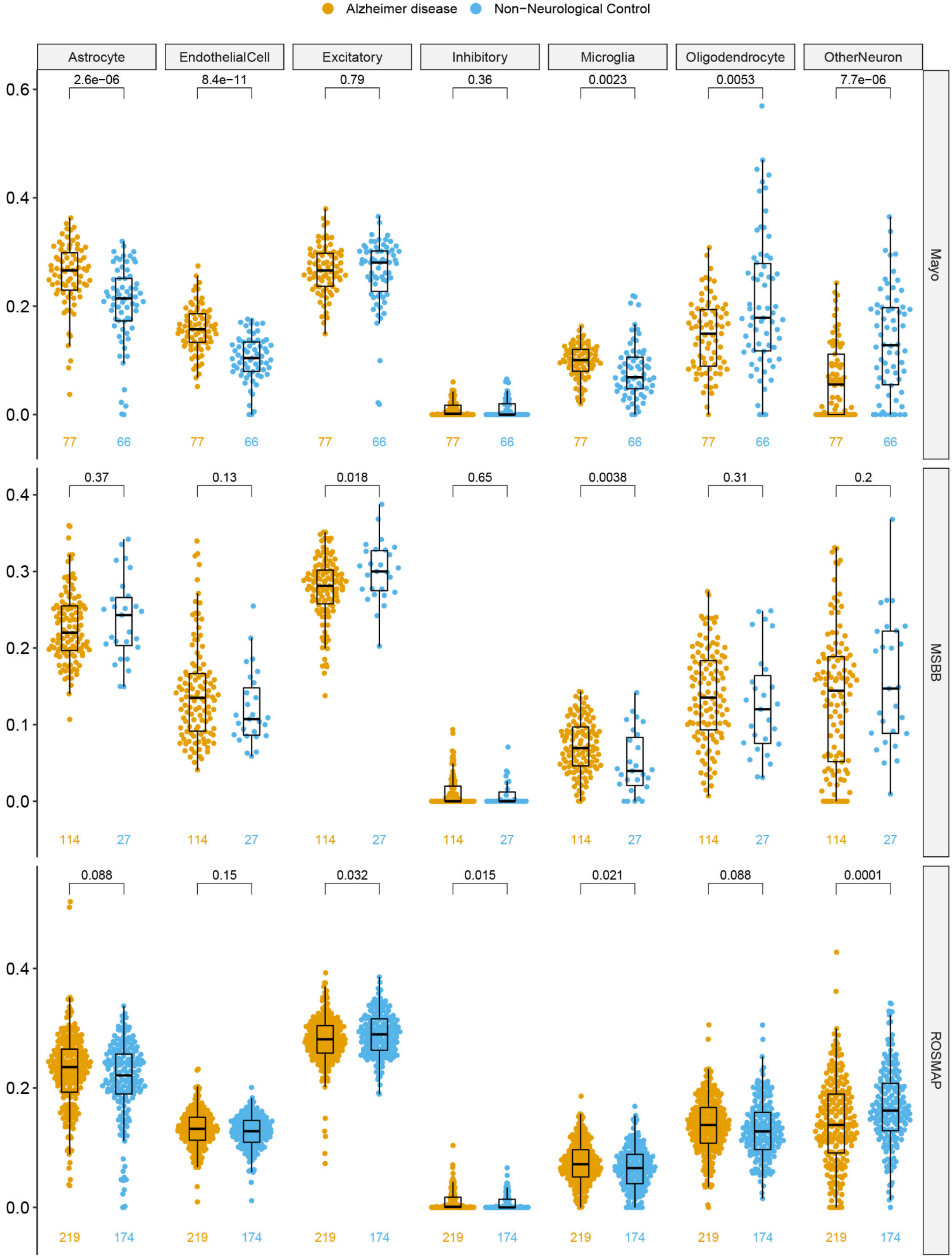

Supplementary Figure 30 - Replication of cortex *trans*-eQTLs (100 PCs) in snRNA-seq data from ROSMAP

Each figure in this plot represents a comparison between bulk RNA-seq (x-axis) and ROSMAP single-nucleus RNA-seq (y-axis). Each dot represents one *trans*-eQTL, and the legend shows the sample size, Pearson correlation coefficient, the AC, and, if applicable, the Rb and π1 statistics. Each column is a comparison between equivalent cell types in both datasets. Each row illustrates a different filtering on which eQTLs are shown. The x-axis always denotes the log interaction beta from Decon-QTL, the y-axis always denotes the log beta of the eQTL effect in the single-nucleus dataset. (a) All overlapping (i)eQTLs (b) (i)eQTLs filtered on being significant in *MetaBrain* (c) (i)eQTLs filtered on being significant in each respective dataset. Colored bands indicate 95% confidence interval around regression line.

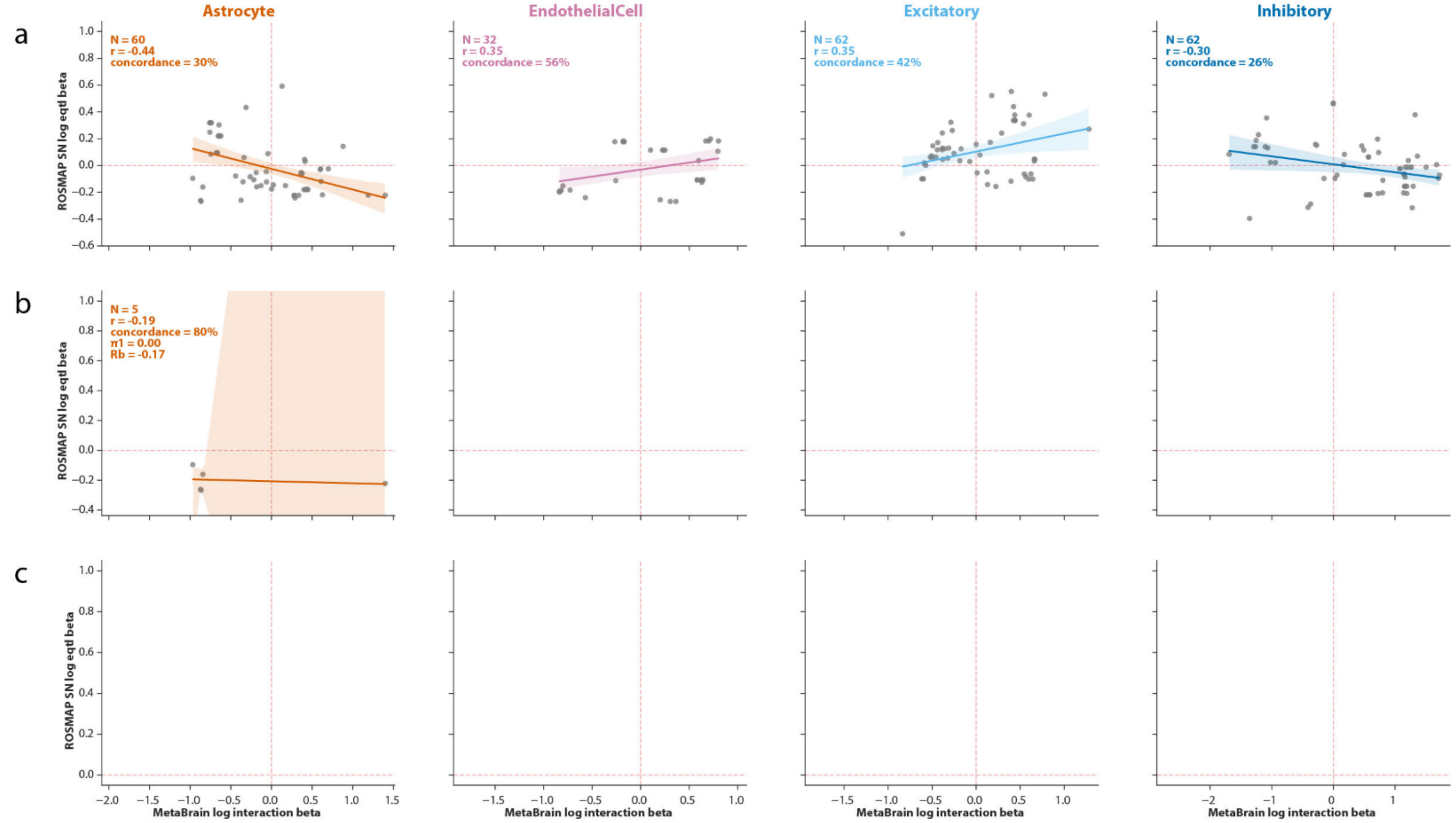

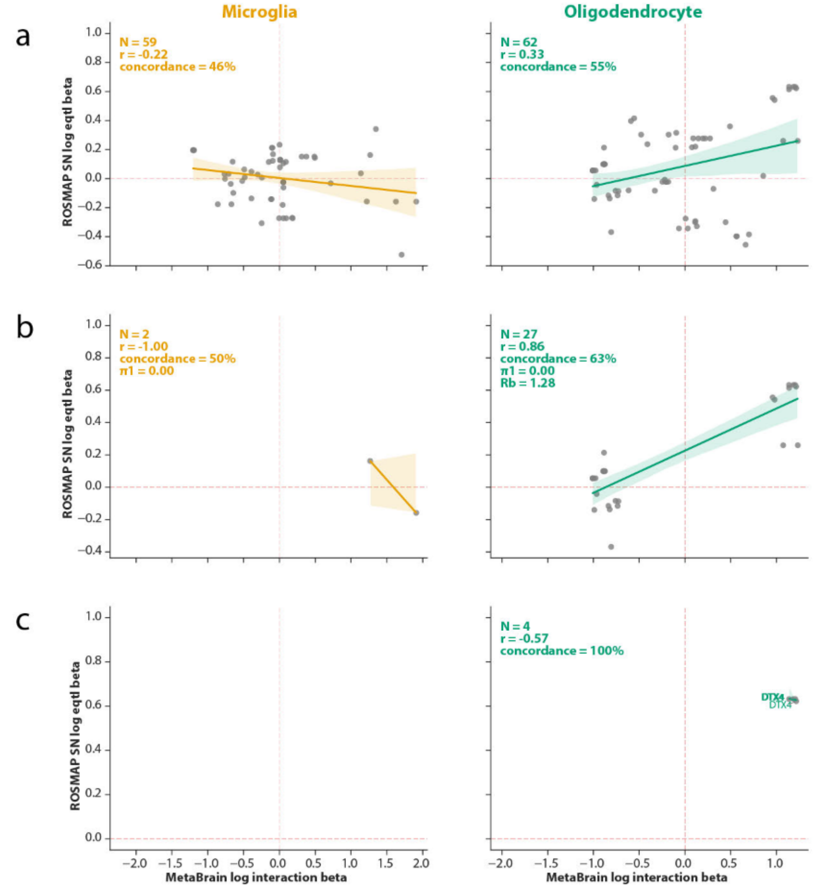

Supplementary Figure 31

(a) UMAP representation of heterogeneous multi-tissue gene network. Immune and blood cell types show increased gene expression levels for genes prioritized using *Downstreamer* for multiple sclerosis, while decreased expression is observed in brain related tissues. (b) Within *MetaBrain*, those same genes show lower expression in cortex, but higher expression in spinal cord and cerebellum.

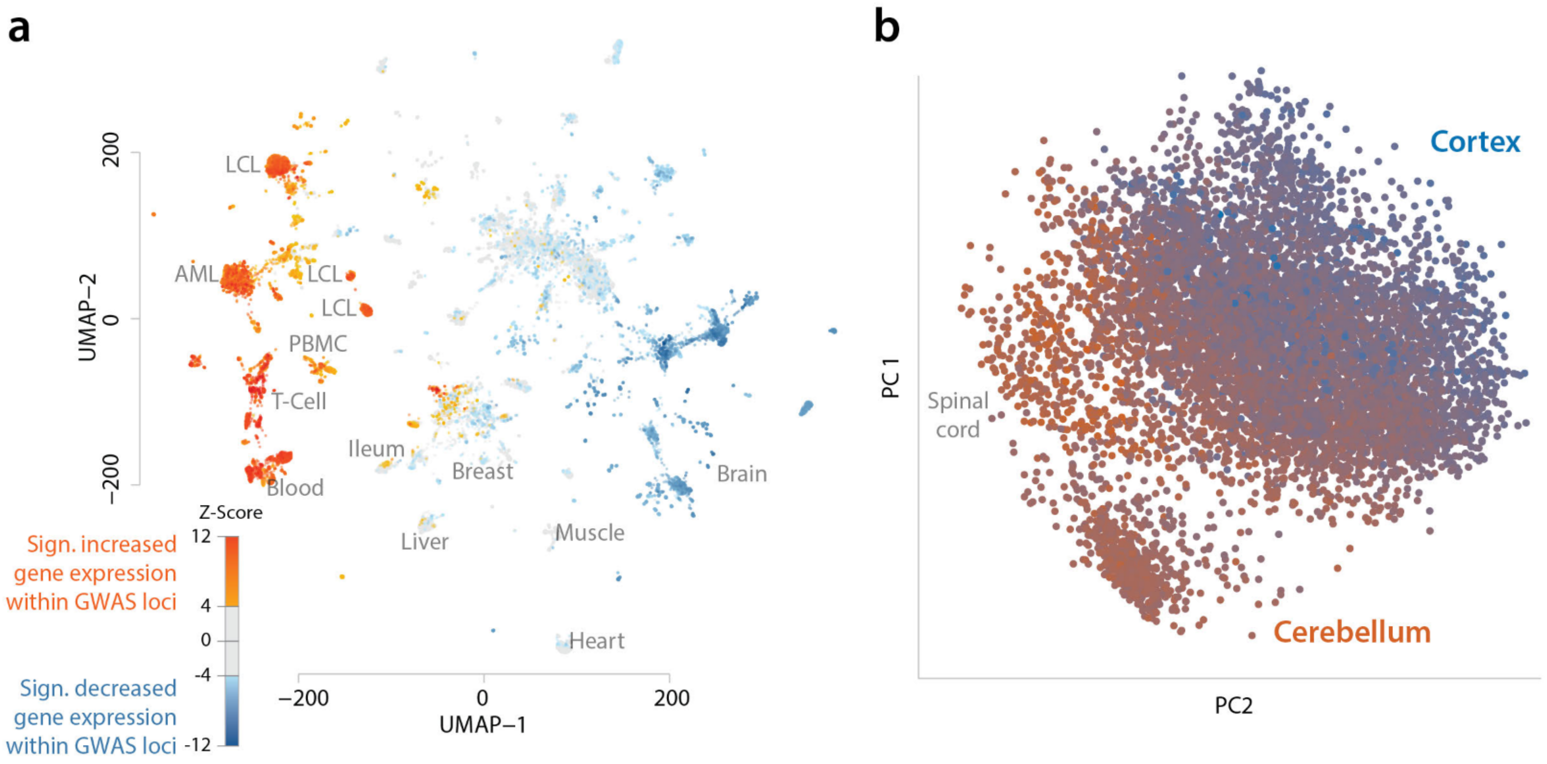

Supplementary Figure 32 - Comparison of AUC distribution for different eigenvector cut-offs

The quality of the gene network that we built for *MetaBrain* is measured by an AUC for each gene derived from a leave-one-out procedure. One of the parameters to build the network is the number of eigenvectors to use after PCA over the gene correlation matrix. Here we show for the 6 annotation categories (KEGG, REACTOME, GO Biological Process, GO Molecular Function, GO Cellular Component, and HPO) the AUC mean (dot) and standard deviation (lines) at different eigenvector cut-offs. The red dot and line indicate the eigenvector cut-off that was used for that annotation category.

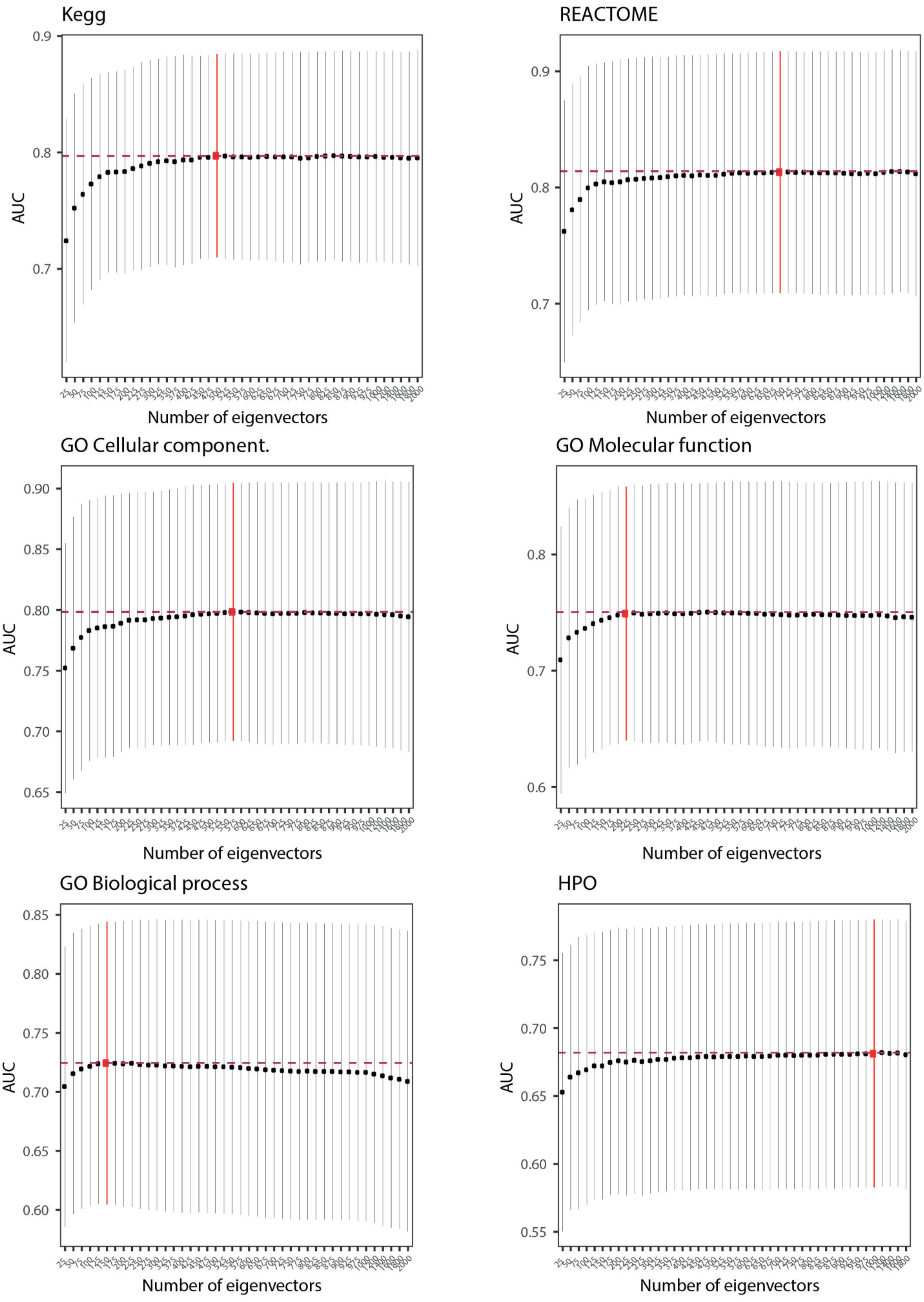

Supplementary Figure 33 - Heatmaps of the Pearson correlation of the AUC values between different eigenvector cut-offs

Correlation was calculated between the different eigenvector cutoffs for the 6 annotation categories.

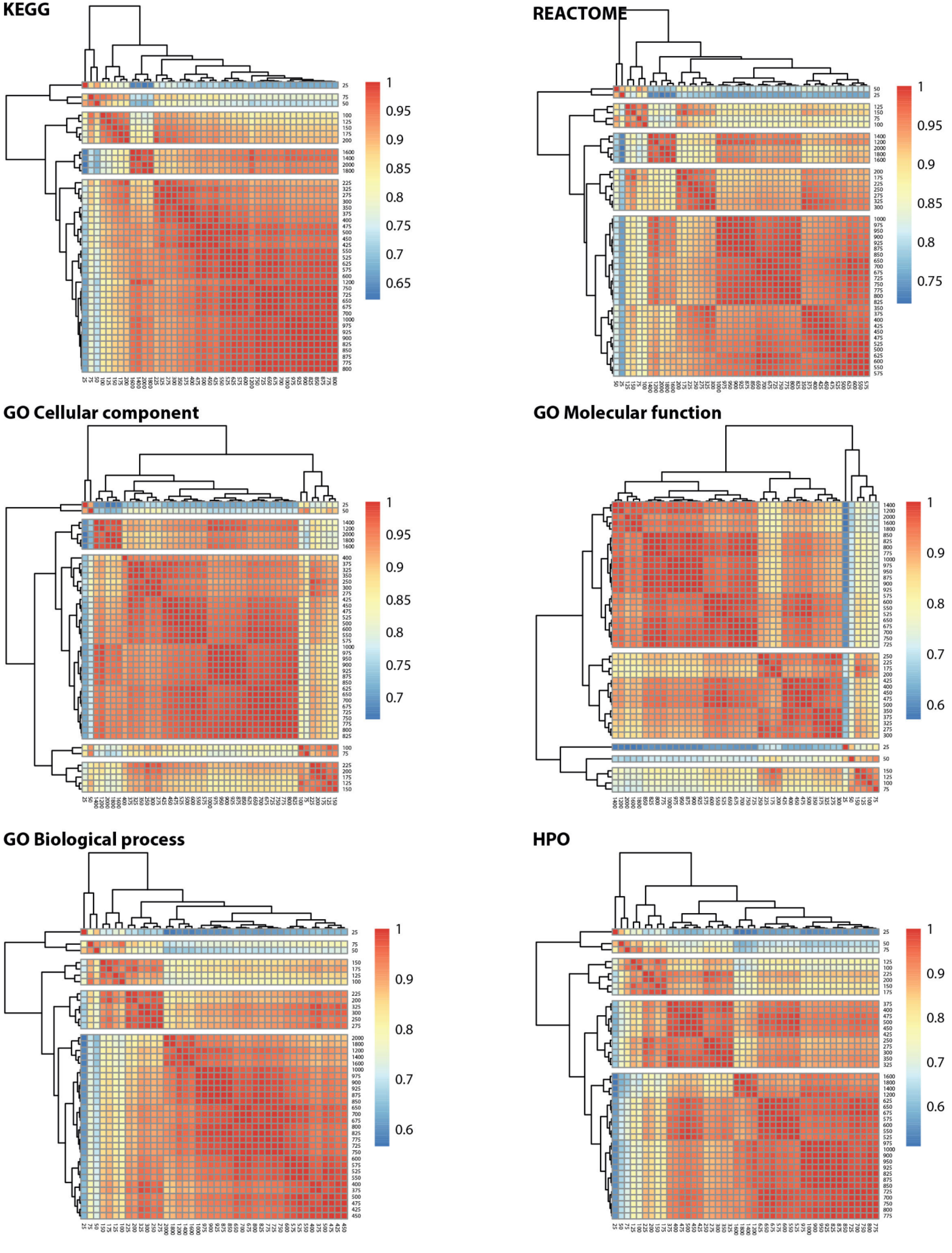

Supplementary Figure 34 - *Downstreamer* with and without including eQTL information

The *Downstreamer* analysis was repeated while explicitly including *cis*-eQTL SNPs, but this did not alter results.

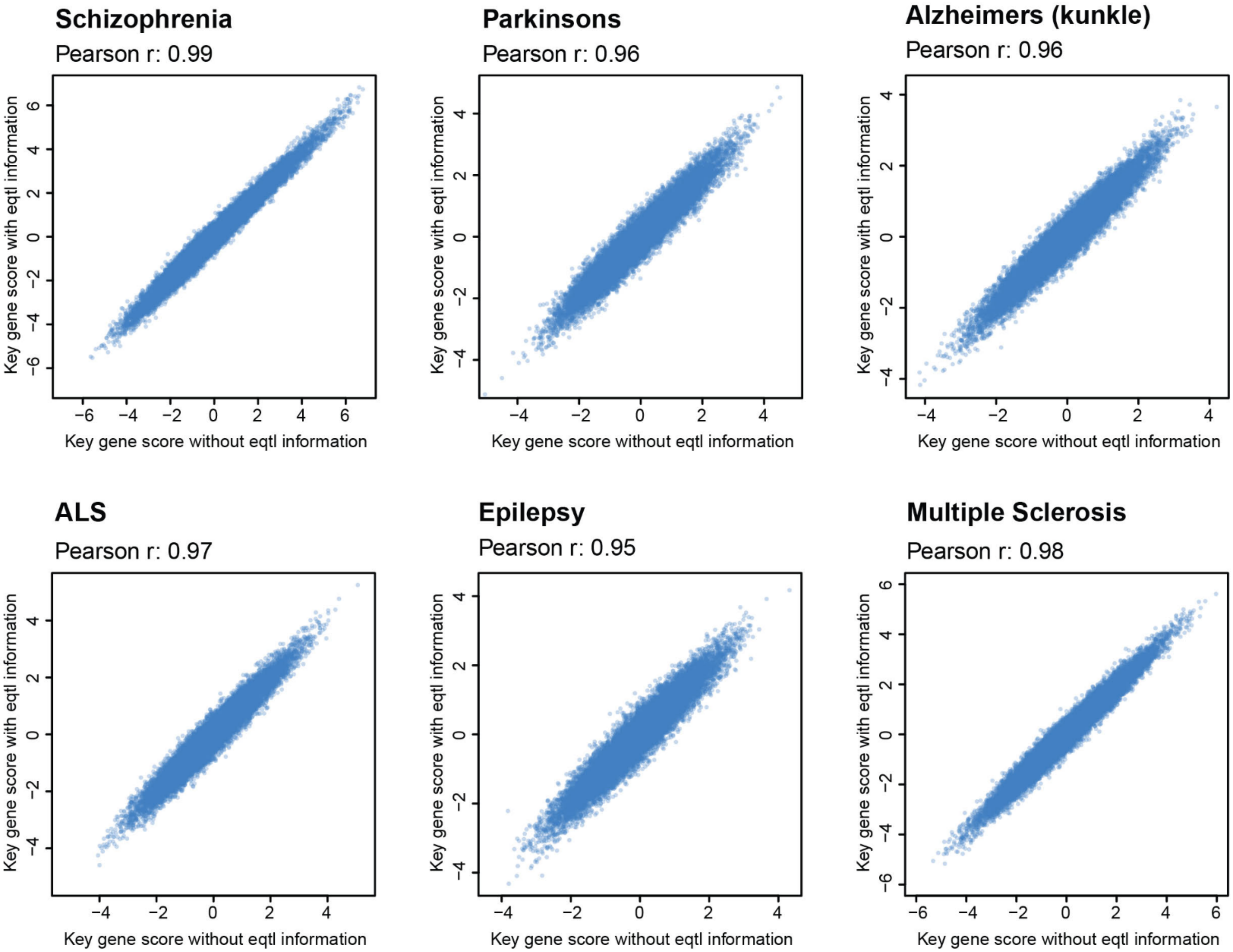

Supplementary Figure 35 - Comparison between eQTL multiple testing correction methods based on permutations

(a) -log10 p-value comparison between EMP and FastQTL shows highly similar p-values for tested eQTLs. (b) -log10 FDR and q-value comparison between EMP and FastQTL shows that FastQTL produces more significant results. eQTL mapping pipeline p-values are capped at 4.946e-324 as this is the lowest value Java can return. (c) Comparison of -log10 q-values between mbQTL and FastQTL showing highly similar q-values. (d) Zoom in shows that major differences between methods are near significance thresholds, and that FastQTL q-values are slightly higher compared to those from mbQTL. (a-d) R is the pearson correlation. P-value test statistic is based on Pearson's product moment correlation coefficient and follows a t distribution with 18,140-2 (a, b) or 18,417-2 (c,d) degrees of freedom. (e) distance to TSS for EMP, FastQTL and mbQTL, visualized by ranking each eQTL (significant and not significant) by the nominal p-value. This shows that variance in TSS increases the less significant an eQTL is. Red dotted line indicates FDR/q-value < 0.05. Blue dotted line indicates FDR/q-value < 0.01.

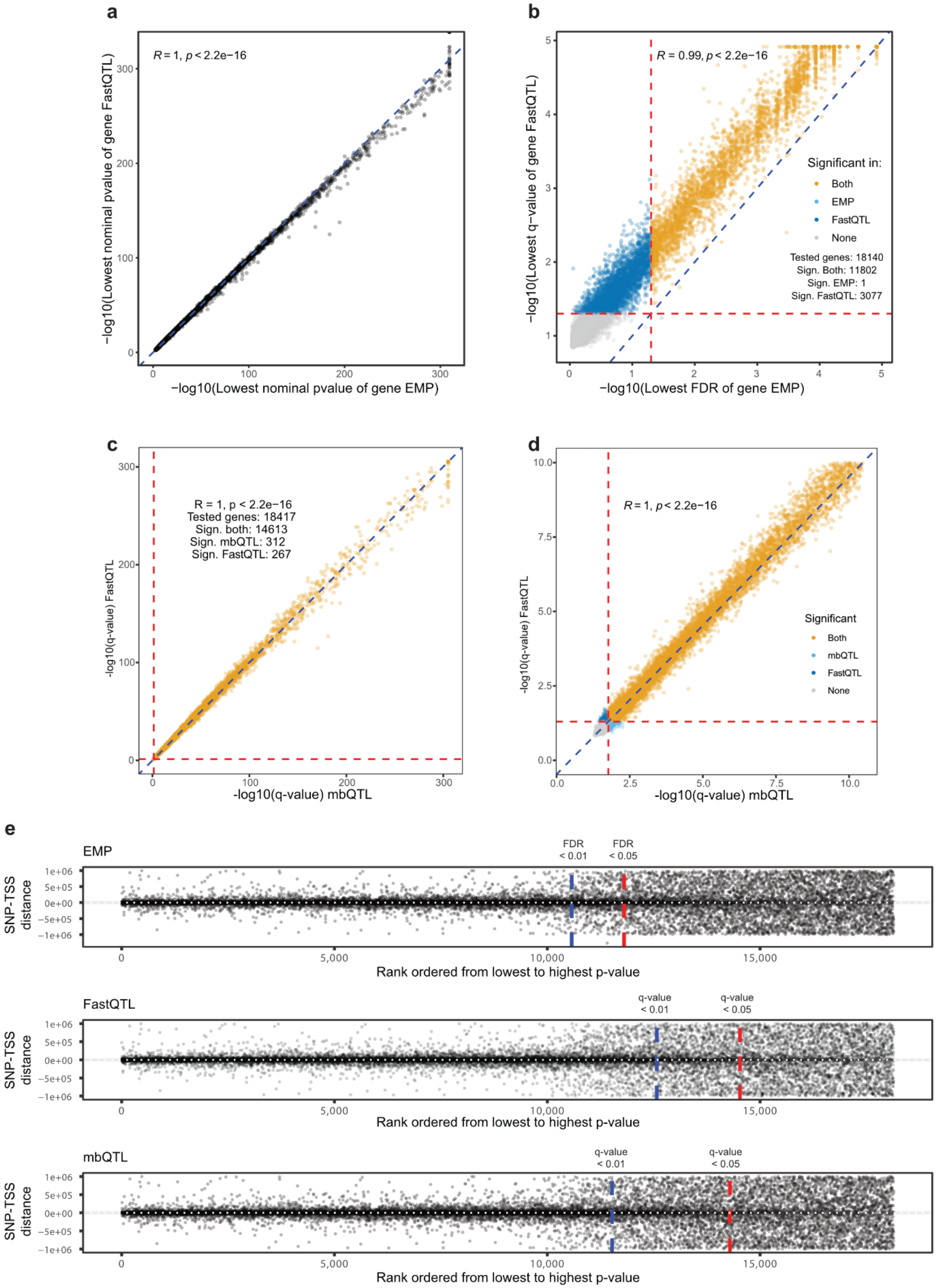

Supplementary Figure 36 - Pearson correlation heatmap of predicted cell fractions versus expression principal components

A heatmap showing the correlation between the predicted cell type proportions and TPM expression matrix principal components. The PCA components are determined over the full expression matrix (n genes = 57,886), a subset of which is directly used for the prediction of the cell type proportions. Each cell contains the Pearson correlation coefficient; blue denotes a negative correlation, red a positive correlation and white denotes no correlation.

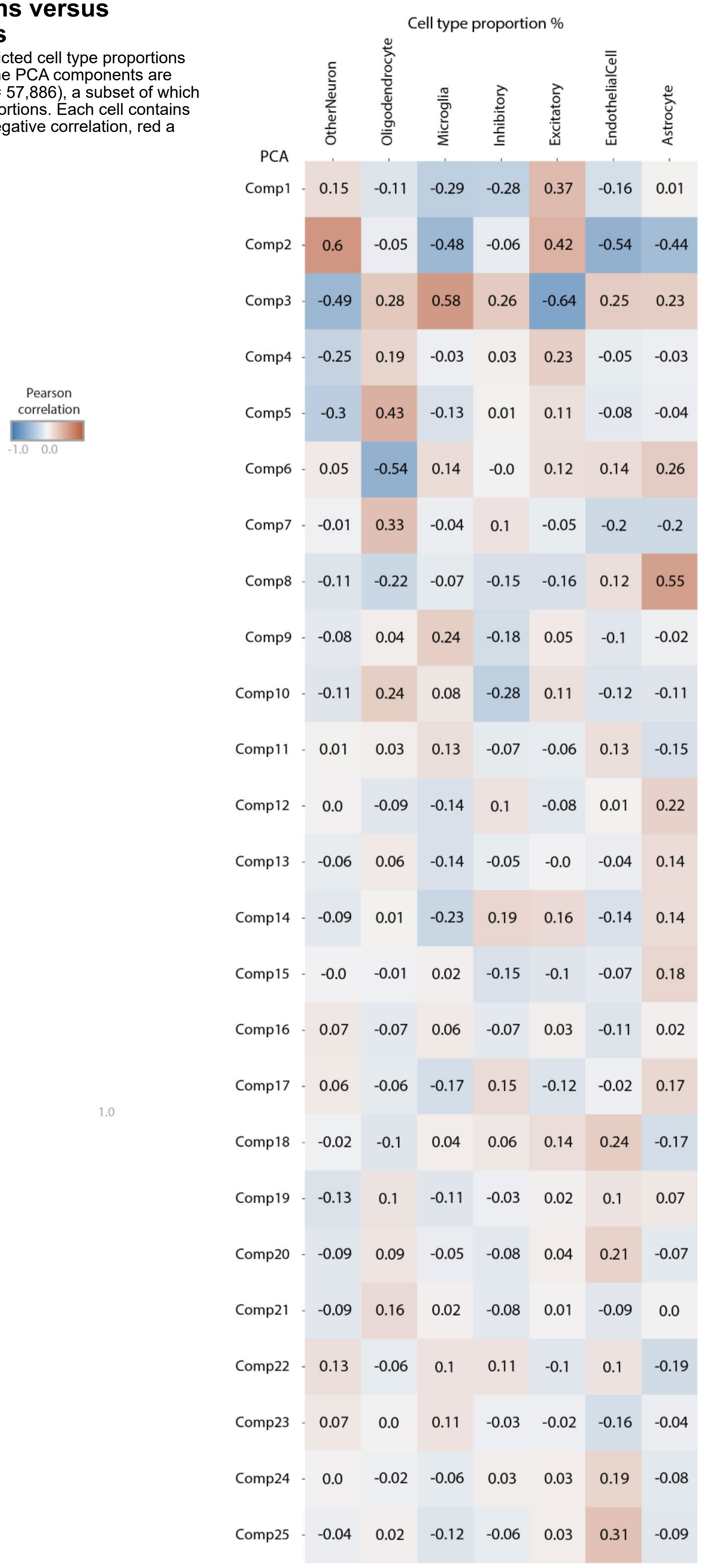

PCA	OtherNeuron	Oligodendrocyte	Microglia	Inhibitory	Excitatory	EndothelialCell	Astrocyte
Comp1	0.15	-0.11	-0.29	-0.28	0.37	-0.16	0.01
Comp2	0.6	-0.05	-0.48	-0.06	0.42	-0.54	-0.44
Comp3	-0.49	0.28	0.58	0.26	-0.64	0.25	0.23
Comp4	-0.25	0.19	-0.03	0.03	0.23	-0.05	-0.03
Comp5	-0.3	0.43	-0.13	0.01	0.11	-0.08	-0.04
Comp6	0.05	-0.54	0.14	-0.0	0.12	0.14	0.26
Comp7	-0.01	0.33	-0.04	0.1	-0.05	-0.2	-0.2
Comp8	-0.11	-0.22	-0.07	-0.15	-0.16	0.12	0.55
Comp9	-0.08	0.04	0.24	-0.18	0.05	-0.1	-0.02
Comp10	-0.11	0.24	0.08	-0.28	0.11	-0.12	-0.11
Comp11	0.01	0.03	0.13	-0.07	-0.06	0.13	-0.15
Comp12	0.0	-0.09	-0.14	0.1	-0.08	0.01	0.22
Comp13	-0.06	0.06	-0.14	-0.05	-0.0	-0.04	0.14
Comp14	-0.09	0.01	-0.23	0.19	0.16	-0.14	0.14
Comp15	-0.0	-0.01	0.02	-0.15	-0.1	-0.07	0.18
Comp16	0.07	-0.07	0.06	-0.07	0.03	-0.11	0.02
Comp17	0.06	-0.06	-0.17	0.15	-0.12	-0.02	0.17
Comp18	-0.02	-0.1	0.04	0.06	0.14	0.24	-0.17
Comp19	-0.13	0.1	-0.11	-0.03	0.02	0.1	0.07
Comp20	-0.09	0.09	-0.05	-0.08	0.04	0.21	-0.07
Comp21	-0.09	0.16	0.02	-0.08	0.01	-0.09	0.0
Comp22	0.13	-0.06	0.1	0.11	-0.1	0.1	-0.19
Comp23	0.07	0.0	0.11	-0.03	-0.02	-0.16	-0.04
Comp24	0.0	-0.02	-0.06	0.03	0.03	0.19	-0.08
Comp25	-0.04	0.02	-0.12	-0.06	0.03	0.31	-0.09

Supplementary Figure 37 - Decon-QTL nominal p-value per cell type

Decon-QTL nominal p-value distribution per cell type for all tested eQTLs.

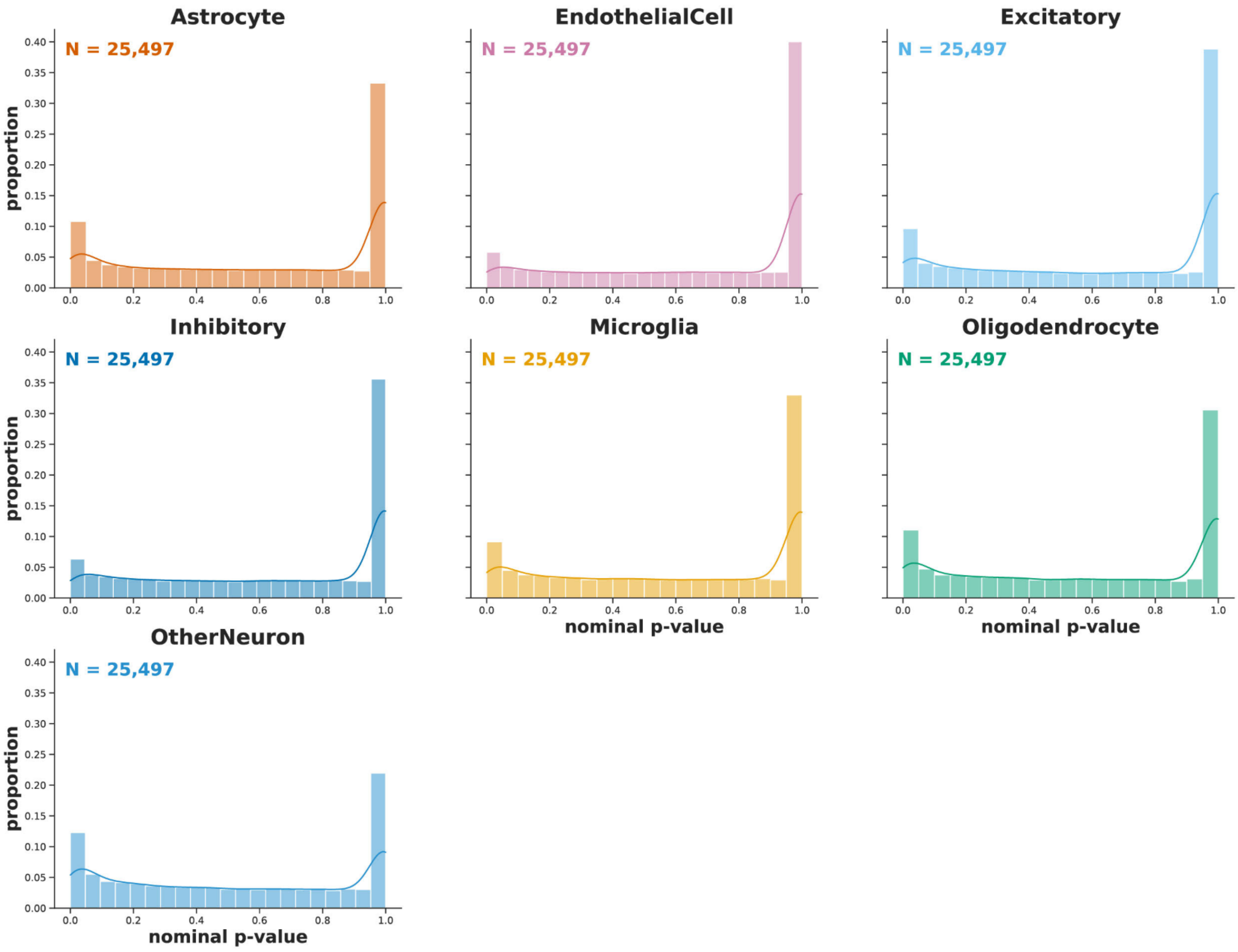

Supplementary Figure 38 - Decon-QTL multiple testing comparison

Pairwise comparison per cell type of the interaction FDR values calculated using Benjamini-Hochberg (BH; x-axis) and a permutation-based FDR (EMP; y-axis). Each point is an ieQTL and is colored as follows: green denotes significant in both analyses, blue denotes only significant on the x-axis, orange denotes only significant on the y-axis, and grey is not significant. The horizontal and vertical dashed lines show the significance threshold of FDR = 0.05. The r denotes the Spearman correlation coefficient.

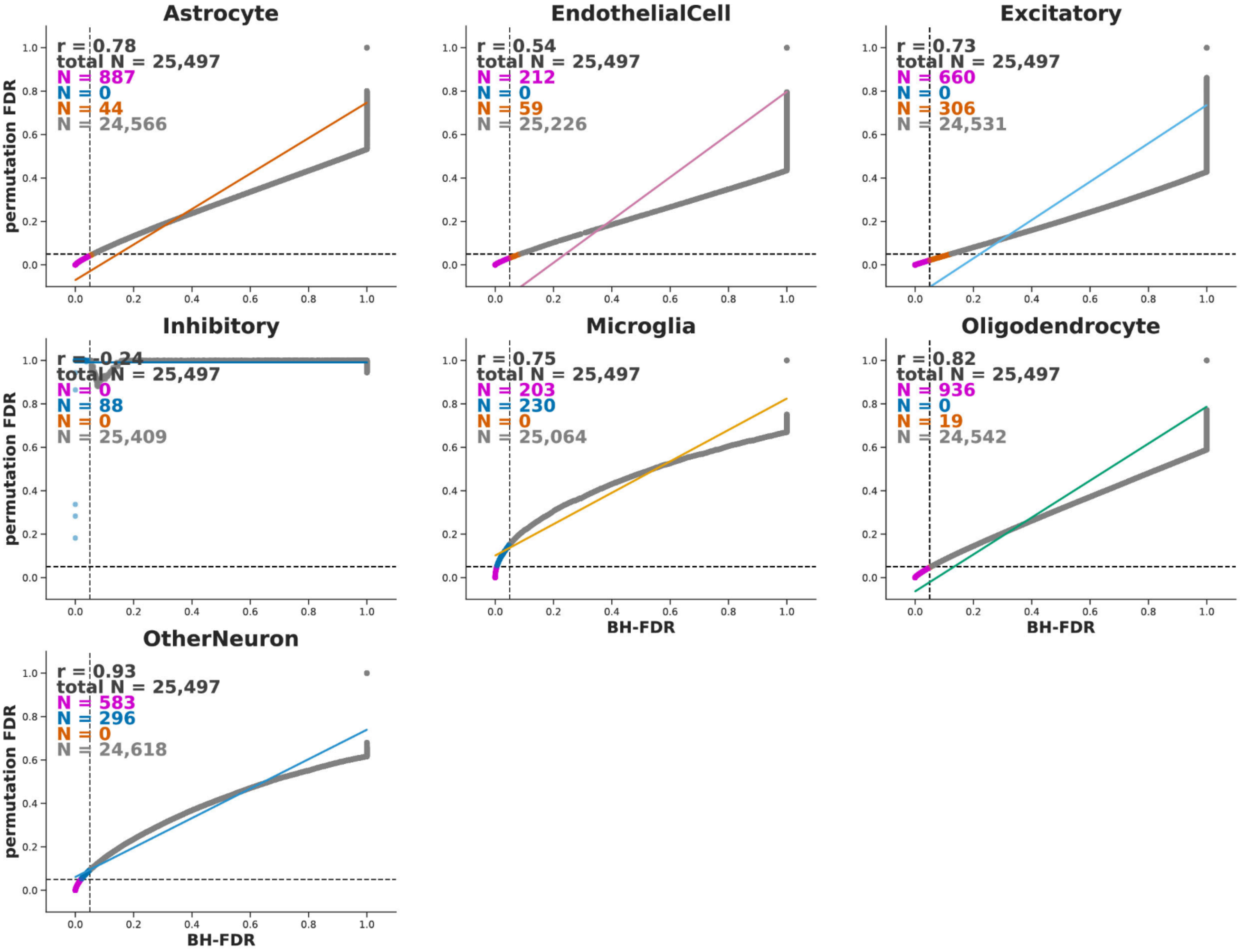

Supplementary Figure 39 - SnRNA-seq visualization by cell type

UMAP dimensionality reduction plot of 39 snRNA-seq samples from ROSMAP. Each dot represents a single cell (n=70,634). The dots are colored by their corresponding cell type: excitatory neurons (EX), oligodendrocytes (OLI), inhibitory neurons (IN), astrocytes (AST), oligodendrocyte precursor cells (OPC), microglia (MIC), pericytes (PER) and endothelial cells (END).

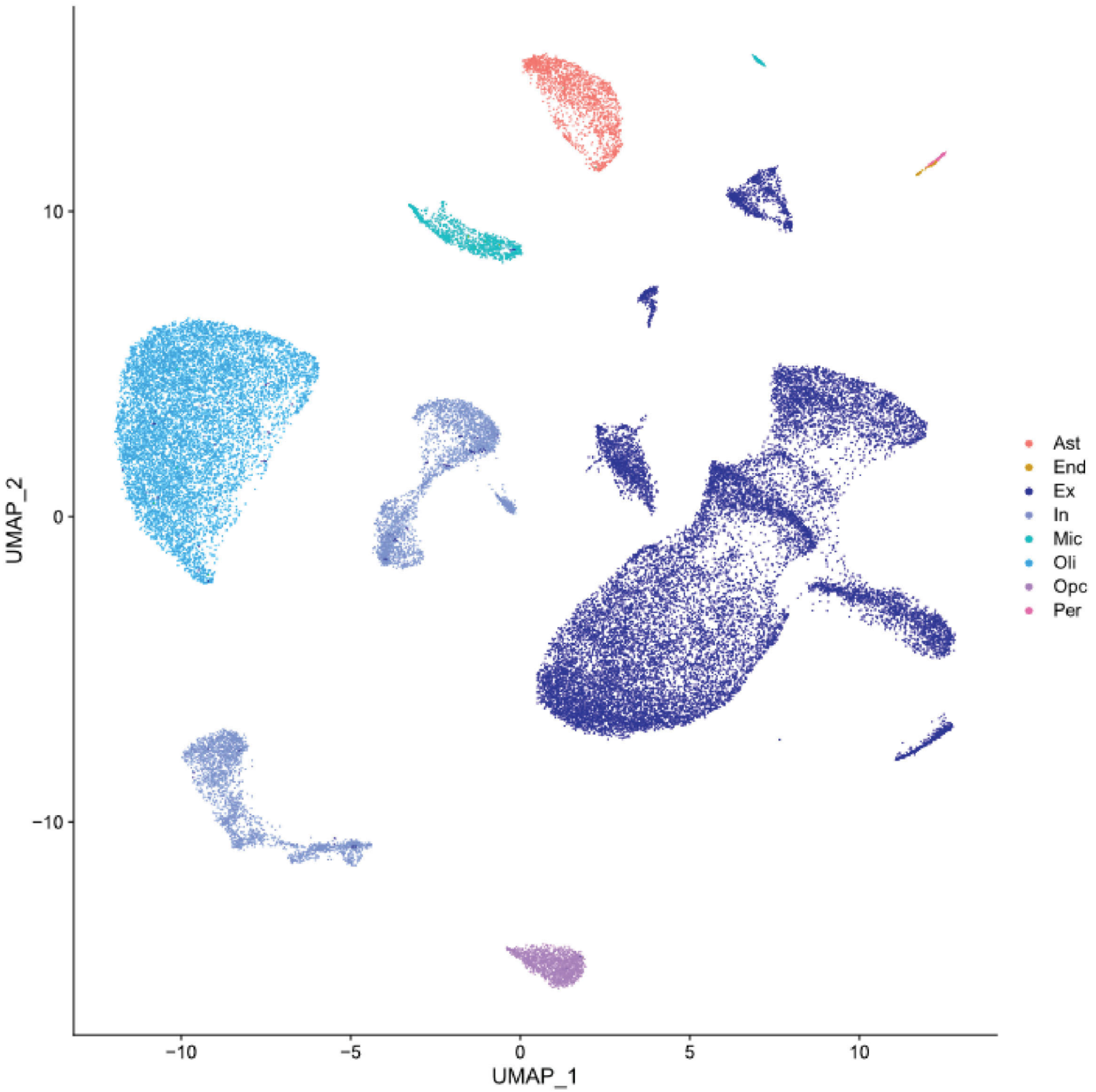

Supplementary Figure 40 - SnRNA-seq visualization by cell type

UMAP dimensionality reduction plot of 39 snRNA-seq samples from ROSMAP. Each dot represents a single cell (n=70,634). The dots are colored by their corresponding cell type subcluster: excitatory neurons (EX), oligodendrocytes (OLI), inhibitory neurons (IN), astrocytes (AST), oligodendrocyte precursor cells (OPC), microglia (MIC), pericytes (PER) and endothelial cells (END).

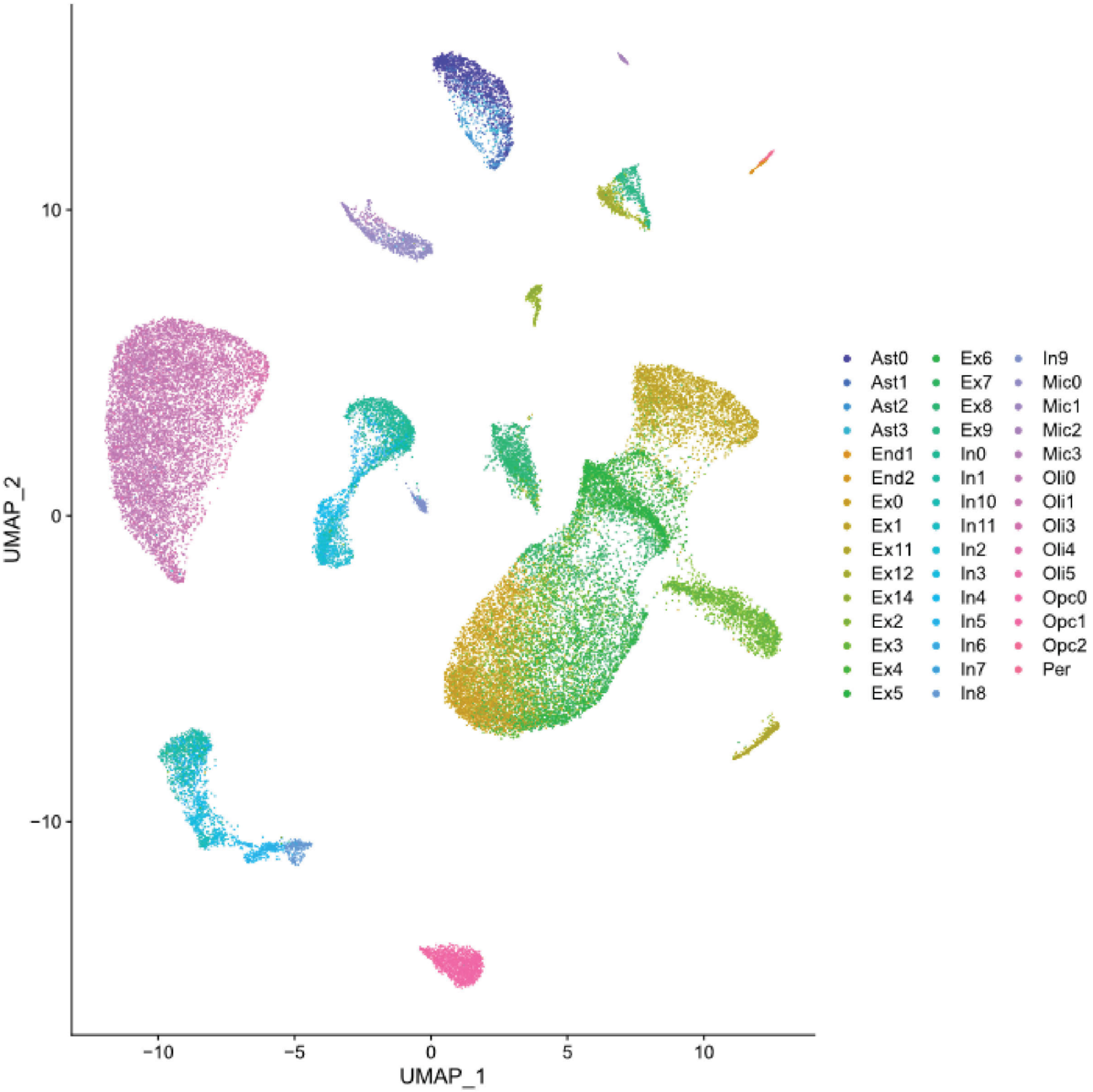

Supplement: Supplementary file 1 — Supplementary Note and Supplementary Figs. 1–40. [file 41588_2023_1300_MOESM1_ESM.pdf]
