# Supplementary material for: Brain expression quantitative trait locus and network analyses reveal downstream effects and putative drivers for brain-related diseases
Source: Nat Genet. 2023 Feb 23;55(3):377–88. doi: 10.1038/s41588-023-01300-6 (PMC10011140; doi:10.1038/s41588-023-01300-6)
Supplement: Supplementary file 2 — Reporting Summary [file 41588_2023_1300_MOESM2_ESM.pdf]

nature portfolio

Corresponding author(s): Harm-Jan Westra

Last updated by author(s): Dec 1, 2022

Reporting Summary

Nature Portfolio wishes to improve the reproducibility of the work that we publish. This form provides structure for consistency and transparency in reporting. For further information on Nature Portfolio policies, see our Editorial Policies and the Editorial Policy Checklist.

Statistics

For all statistical analyses, confirm that the following items are present in the figure legend, table legend, main text, or Methods section.

n/a	Confirmed	
☐	☒	The exact sample size (*n*) for each experimental group/condition, given as a discrete number and unit of measurement
☐	☒	A statement on whether measurements were taken from distinct samples or whether the same sample was measured repeatedly
☐	☒	The statistical test(s) used AND whether they are one- or two-sided<br>*Only common tests should be described solely by name; describe more complex techniques in the Methods section.*
☐	☒	A description of all covariates tested
☐	☒	A description of any assumptions or corrections, such as tests of normality and adjustment for multiple comparisons
☐	☒	A full description of the statistical parameters including central tendency (e.g. means) or other basic estimates (e.g. regression coefficient) AND variation (e.g. standard deviation) or associated estimates of uncertainty (e.g. confidence intervals)
☐	☒	For null hypothesis testing, the test statistic (e.g. *F*, *t*, *r*) with confidence intervals, effect sizes, degrees of freedom and *P* value noted<br>*Give P values as exact values whenever suitable.*
☐	☒	For Bayesian analysis, information on the choice of priors and Markov chain Monte Carlo settings
☒	☐	For hierarchical and complex designs, identification of the appropriate level for tests and full reporting of outcomes
☐	☒	Estimates of effect sizes (e.g. Cohen's *d*, Pearson's *r*), indicating how they were calculated

Our web collection on statistics for biologists contains articles on many of the points above.

Software and code

Policy information about availability of computer code

Data collection	A number of datasets (AMP-AD datasets, CMC, CMC_HBCC, BrainSeq, UCLA_ASD, BrainGVEx, BipSeq) were collected from Synapse.org using the Pythong synapseclient (version 1.9.2) pacakge. Other datasets (GTEx, NABEC) were acquired from dbGAP using NCBI's SRA-tools. Two datasets (TargetALS and Braineac) were made available through personal communication with investigators of those studies.
Data analysis	Alignment for eQTL analysis was performed using STAR v2.6.1c and TMM normalization was performed using edgeR v3.20.9. Alignment for creation of gene networks was performed using Kallisto v0.43.1. RNA-seq genotype calling was performed using GATK v4.0.9.1. RNA-seq QC was performed using FastQC v0.11.3, STAR metrics, and Picard tools v2.18.26. Genotype processing was performed using PLINK v2.0 and bcftools v1.7. Where applicable genotypes were harmonized using GenotypeHarmonizer (https://github.com/molgenis/systemsgenetics/tree/master/Genotype-Harmonizer), and imputed to HRC v1.1 using the Michigan imputation server (https://imputationserver.sph.umich.edu/index.html). Additional custom scripts are available at https://github.com/molgenis/metabrain. This repository includes code for prediction of cell type proportions using the NNLS implementation of SciPy v1.4.1. eQTL analysis software (v1.4.9) is available at https://github.com/molgenis/systemsgenetics/tree/master/eqtl-mapping-pipeline. and https://github.com/molgenis/systemsgenetics/tree/master/mbQTL. Mendelian randomization was performerd using the ieugwasr and TwoSampleMR R packages. Colocalization was performed using COLOC. Downstreamer is available at https://github.com/molgenis/systemsgenetics/tree/master/Downstreamer. Allelic fold-change analysis (aFC was performed using the aFC.py script in this github repository: https://github.com/secastel/aFC/. Single cell data was analyzed using Seurat v3.2.2119.

For manuscripts utilizing custom algorithms or software that are central to the research but not yet described in published literature, software must be made available to editors and reviewers. We strongly encourage code deposition in a community repository (e.g. GitHub). See the Nature Portfolio guidelines for submitting code & software for further information.

Data

Policy information about availability of data

All manuscripts must include a data availability statement. This statement should provide the following information, where applicable:

- Accession codes, unique identifiers, or web links for publicly available datasets
- A description of any restrictions on data availability
- For clinical datasets or third party data, please ensure that the statement adheres to our policy

Our study is comprised of previously published human brain eQTL datasets. The majority of these datasets are available upon request, or through online repositories after signing data acces agreements. We have listed the mode of access of each of the included datasets below. Summary statistics for the performed eQTL analyses will be available through https://www.metabrain.nl. TargetALS data was pushed directly from the NY Genome center to our sftp server.
CMC data was downloaded from https://www.synapse.org/ using synapse client (https://python-docs.synapse.org/build/html/index.html). Accession code: syn2759792
GTEx was downloaded from SRA using fastq-dump of the SRA toolkit (http://www.ncbi.nlm.nih.gov/Traces/sra/sra.cgi?cmd=show&f=software&m=software&s=software). Access has been requested and granted through dbGaP, accession code: phs000424.v7.p2.
Braineac data has been pushed to our sftp server by Biogen.
AMP-AD data has been downloaded from synapse13. Accession code: syn2580853. snRNA-seq was collected using Synapse accession code: syn18485175. IHC data: https://github.com/ellispatrick/CortexCellDeconv/tree/master/CellTypeDeconvAnalysis/Data
ENA data has been downloaded from the European Nucleotide Archive. The identifiers of the 76 included studies and 2021 brain samples are listed in Supplementary Table 31.
CMC_HBCC data was downloaded from https://www.synapse.org/ using synapse client (https://python-docs.synapse.org/build/html/index.html). Accession code: syn10623034.
BrainSeq data was downloaded from https://www.synapse.org/ using synapse client (https://python-docs.synapse.org/build/html/index.html). Accession code: syn12299750
UCLA_ASD data was downloaded from https://www.synapse.org/ using synapse client (https://python-docs.synapse.org/build/html/index.html). Accession code: syn4587609
BrainGVEx data was downloaded from https://www.synapse.org/ using synapse client (https://python-docs.synapse.org/build/html/index.html). Accession code: syn4590909
BipSeq data was downloaded from https://www.synapse.org/ using synapse client (https://python-docs.synapse.org/build/html/index.html). Accession code: syn5844980
NABEC data was downloaded from dbgap. Accession code: phs001301.v1.p1
Bryois et al. 2022. Bryois et al. 2022 eQTL summary statistics where downloaded from Zenodo on January 2022 from https://doi.org/10.5281/zenodo.5543734.
Other databaes/datasets that we have used: SkyMap (https://github.com/brianyiktaktsui/Skymap).

Human research participants

Policy information about studies involving human research participants and Sex and Gender in Research.

Reporting on sex and gender	We do not report on sex or gender.
Population characteristics	Our study includes 13 previously published brain eQTL datasets, and one dataset created from publicly available RNA-seq samples. In toal, we collected 8,613 RNA-seq samples and 6,518 genotype samples. Depending on the dataset, different clinical covariates were either available or unavailable, including age, gender and ancestry. Depending on the dataset, disease status was available (Alzheimer's disease for AMP-AD, several psychiatric diseases for Psychencode datasets, amyotrophic lateral sclerosis for TargetALS). Due to the high missingness within these clinical covariates, we did not explicitly correct for these covariates, but used principal components, which capture variation in gene expression caused by these covariates
Recruitment	Participants were not recruited by the authors of this study.
Ethics oversight	All included datasets recruited participants with informed consent, and data was collected in accordance with ethical and institutional regulations, according to their institutional review boards (IRBs). All data was anlayzed in compliance with signed data access agreements, which have been reviewed by the IRBs or data access committees of each of the included datasets.

Note that full information on the approval of the study protocol must also be provided in the manuscript.

Field-specific reporting

Please select the one below that is the best fit for your research. If you are not sure, read the appropriate sections before making your selection.

☒ Life sciences ☐ Behavioural & social sciences ☐ Ecological, evolutionary & environmental sciences

For a reference copy of the document with all sections, see nature.com/documents/nr-reporting-summary-flat.pdf

Life sciences study design

All studies must disclose on these points even when the disclosure is negative.

Sample size	Our study includes 13 previously published brain eQTL datasets, and one dataset created from publicly available RNA-seq samples. In total, we collected 8,613 RNA-seq samples and 6,518 genotype samples. We did not calculate sample size, instead we aimed to include all (at that time) available bulk brain RNA-seq+genotype samples. eQTL analysis was performed in distinct tissue groups: Basal ganglia-EUR (n=208), Cerebellum-EUR (n=492), Cortex-EUR (n=2,683), Cortex-AFR (n=319), Hippocampus-EUR (n=168) and Spinal cord-EUR (n=108). For each of these analyses, we required that each tested eQTL was present in at least 2 cohorts. As eQTL analysis was performed previously on each of the individual cohorts separately, we reasoned that this would provide sufficient statistical power for our analysis.
Data exclusions	A number of samples were excluded during the quality control steps outlined in the methods section of our manuscript (i.e. outliers in terms of alignment metrics, or outliers identified through principal component analysis). For eQTL analysis, if an individual had multiple RNA-seq measurements for the same tissue, we randomly selected a single sample for inclusion, and excluded the others. Similarly, related genotype samples were excluded from analysis. Some samples, such as those for the amygdala and hypothalamus tissues, or samples from SAS or AMR datasets ancestry were excluded from eQTL analysis because their group sizes were deemed too small (n<30), or there were less than 2 available datsets for that particular group. Group definitions and sample inclusion per group are listed in Supplementary Table 1.
Replication	All tested eQTLs were required to be present in at least 2 of the included datasets. Cis-eQTLs were replicated in GTEx tissues by excluding the GTEx cohort from the discovery analysis. We osbserved high concordance in allelic direction, correlation of genetic effect sizes, and pi1 replication rates in cortical tissues. Interaction eQTLs detected in Cortex-EUR were replicated in the Cortex-AFR dataset, a single nucleus RNA-seq dataset and using summary statistics of an independent single nucleus RNA-seq study. Trans-eQTLs were replicated in a single nucleus RNA-seq dataset. We observed high agreement for both these types of eQTLs.
Randomization	RNA-seq samples were assigned to different groups according to (predicted) brain region. The datset consisted of many different tissues from similar brain regions. These tissues were merged into larger brain tissue groups (i.e. amygdala, basal ganglia, cortex, cerebellum, hypothalamus, hippocampus, spinalcord). Tissue grouping is defined in Supplementary Table 1. Genotype samples were grouped according to ancestry, including African (AFR), European (EUR), East Asian (EAS), South Asian (SAS), and admixed American (AMR). To define eQTL datasets, tissue and ancestry groups were overlapped, but analysis was limited to groups having samples from two or more datasets. This created Basal ganglia-EUR, Cerebellum-EUR, Cortex-EUR, Cortex-AFR, Hippocampsu-EUR, and Spinalcord-EUR eQTL datasets. For trans-eQTL analysis an additional dataset was defined, by merging Cortex-EUR and Cortex-AFR, but excluding the ENA dataset. To correct for dataset or batch effects we included several covariates, including dataset indicator variables, principal components, several RNA-seq alignment QC metrics, and 4 MDS components derived from the genotype data to further account for potential population stratification. eQTL analysis tests associations etween genotype (groups) and gene expression levels. Assignment of each individual to a genotype group for a given random can be considered randomized when accounting for the mentioned covariates.
Blinding	Since our study uses previously published datasets, the authors were not involved in group allocation during data collection. Furthemore, this study does not represent a clinical trial or an interventional study, so blinding was not required. As described from above, assignment of an individual to a genotype group of a given variant can be considered random.

Behavioural & social sciences study design

All studies must disclose on these points even when the disclosure is negative.

Study description	*Briefly describe the study type including whether data are quantitative, qualitative, or mixed-methods (e.g. qualitative cross-sectional, quantitative experimental, mixed-methods case study).*
Research sample	*State the research sample (e.g. Harvard university undergraduates, villagers in rural India) and provide relevant demographic information (e.g. age, sex) and indicate whether the sample is representative. Provide a rationale for the study sample chosen. For studies involving existing datasets, please describe the dataset and source.*
Sampling strategy	*Describe the sampling procedure (e.g. random, snowball, stratified, convenience). Describe the statistical methods that were used to predetermine sample size OR if no sample-size calculation was performed, describe how sample sizes were chosen and provide a rationale for why these sample sizes are sufficient. For qualitative data, please indicate whether data saturation was considered, and what criteria were used to decide that no further sampling was needed.*
Data collection	*Provide details about the data collection procedure, including the instruments or devices used to record the data (e.g. pen and paper, computer, eye tracker, video or audio equipment) whether anyone was present besides the participant(s) and the researcher, and whether the researcher was blind to experimental condition and/or the study hypothesis during data collection.*
Timing	*Indicate the start and stop dates of data collection. If there is a gap between collection periods, state the dates for each sample cohort.*
Data exclusions	*If no data were excluded from the analyses, state so OR if data were excluded, provide the exact number of exclusions and the rationale behind them, indicating whether exclusion criteria were pre-established.*
Non-participation	*State how many participants dropped out/declined participation and the reason(s) given OR provide response rate OR state that no participants dropped out/declined participation.*

Randomization	*If participants were not allocated into experimental groups, state so OR describe how participants were allocated to groups, and if allocation was not random, describe how covariates were controlled.*

Ecological, evolutionary & environmental sciences study design

All studies must disclose on these points even when the disclosure is negative.

Study description	*Briefly describe the study. For quantitative data include treatment factors and interactions, design structure (e.g. factorial, nested, hierarchical), nature and number of experimental units and replicates.*
Research sample	*Describe the research sample (e.g. a group of tagged Passer domesticus, all Stenocereus thurberi within Organ Pipe Cactus National Monument), and provide a rationale for the sample choice. When relevant, describe the organism taxa, source, sex, age range and any manipulations. State what population the sample is meant to represent when applicable. For studies involving existing datasets, describe the data and its source.*
Sampling strategy	*Note the sampling procedure. Describe the statistical methods that were used to predetermine sample size OR if no sample-size calculation was performed, describe how sample sizes were chosen and provide a rationale for why these sample sizes are sufficient.*
Data collection	*Describe the data collection procedure, including who recorded the data and how.*
Timing and spatial scale	*Indicate the start and stop dates of data collection, noting the frequency and periodicity of sampling and providing a rationale for these choices. If there is a gap between collection periods, state the dates for each sample cohort. Specify the spatial scale from which the data are taken*
Data exclusions	*If no data were excluded from the analyses, state so OR if data were excluded, describe the exclusions and the rationale behind them, indicating whether exclusion criteria were pre-established.*
Reproducibility	*Describe the measures taken to verify the reproducibility of experimental findings. For each experiment, note whether any attempts to repeat the experiment failed OR state that all attempts to repeat the experiment were successful.*
Randomization	*Describe how samples/organisms/participants were allocated into groups. If allocation was not random, describe how covariates were controlled. If this is not relevant to your study, explain why.*
Blinding	*Describe the extent of blinding used during data acquisition and analysis. If blinding was not possible, describe why OR explain why blinding was not relevant to your study.*

Did the study involve field work? ☐ Yes ☐ No

Field work, collection and transport

Field conditions	*Describe the study conditions for field work, providing relevant parameters (e.g. temperature, rainfall).*
Location	*State the location of the sampling or experiment, providing relevant parameters (e.g. latitude and longitude, elevation, water depth).*
Access & import/export	*Describe the efforts you have made to access habitats and to collect and import/export your samples in a responsible manner and in compliance with local, national and international laws, noting any permits that were obtained (give the name of the issuing authority, the date of issue, and any identifying information).*
Disturbance	*Describe any disturbance caused by the study and how it was minimized.*

Reporting for specific materials, systems and methods

We require information from authors about some types of materials, experimental systems and methods used in many studies. Here, indicate whether each material, system or method listed is relevant to your study. If you are not sure if a list item applies to your research, read the appropriate section before selecting a response.

Materials & experimental systems

n/a	Involved in the study
☒	☐ Antibodies
☒	☐ Eukaryotic cell lines
☒	☐ Palaeontology and archaeology
☒	☐ Animals and other organisms
☒	☐ Clinical data
☒	☐ Dual use research of concern

Methods

n/a	Involved in the study
☒	☐ ChIP-seq
☒	☐ Flow cytometry
☐	☒ MRI-based neuroimaging

Antibodies

Antibodies used — *Describe all antibodies used in the study; as applicable, provide supplier name, catalog number, clone name, and lot number.*

Validation — *Describe the validation of each primary antibody for the species and application, noting any validation statements on the manufacturer's website, relevant citations, antibody profiles in online databases, or data provided in the manuscript.*

Eukaryotic cell lines

Policy information about cell lines and Sex and Gender in Research

Cell line source(s) — *State the source of each cell line used and the sex of all primary cell lines and cells derived from human participants or vertebrate models.*

Authentication — *Describe the authentication procedures for each cell line used OR declare that none of the cell lines used were authenticated.*

Mycoplasma contamination — *Confirm that all cell lines tested negative for mycoplasma contamination OR describe the results of the testing for mycoplasma contamination OR declare that the cell lines were not tested for mycoplasma contamination.*

Commonly misidentified lines (See ICLAC register) — *Name any commonly misidentified cell lines used in the study and provide a rationale for their use.*

Palaeontology and Archaeology

Specimen provenance — *Provide provenance information for specimens and describe permits that were obtained for the work (including the name of the issuing authority, the date of issue, and any identifying information). Permits should encompass collection and, where applicable, export.*

Specimen deposition — *Indicate where the specimens have been deposited to permit free access by other researchers.*

Dating methods — *If new dates are provided, describe how they were obtained (e.g. collection, storage, sample pretreatment and measurement), where they were obtained (i.e. lab name), the calibration program and the protocol for quality assurance OR state that no new dates are provided.*

☐ Tick this box to confirm that the raw and calibrated dates are available in the paper or in Supplementary Information.

Ethics oversight — *Identify the organization(s) that approved or provided guidance on the study protocol, OR state that no ethical approval or guidance was required and explain why not.*

Note that full information on the approval of the study protocol must also be provided in the manuscript.

Animals and other research organisms

Policy information about studies involving animals; ARRIVE guidelines recommended for reporting animal research, and Sex and Gender in Research

Laboratory animals — *For laboratory animals, report species, strain and age OR state that the study did not involve laboratory animals.*

Wild animals — *Provide details on animals observed in or captured in the field; report species and age where possible. Describe how animals were caught and transported and what happened to captive animals after the study (if killed, explain why and describe method; if released, say where and when) OR state that the study did not involve wild animals.*

Reporting on sex — *Indicate if findings apply to only one sex; describe whether sex was considered in study design, methods used for assigning sex. Provide data disaggregated for sex where this information has been collected in the source data as appropriate; provide overall numbers in this Reporting Summary. Please state if this information has not been collected. Report sex-based analyses where performed, justify reasons for lack of sex-based analysis.*

Field-collected samples | *For laboratory work with field-collected samples, describe all relevant parameters such as housing, maintenance, temperature, photoperiod and end-of-experiment protocol OR state that the study did not involve samples collected from the field.*

Ethics oversight | *Identify the organization(s) that approved or provided guidance on the study protocol, OR state that no ethical approval or guidance was required and explain why not.*

Note that full information on the approval of the study protocol must also be provided in the manuscript.

Clinical data

Policy information about clinical studies

All manuscripts should comply with the ICMJE guidelines for publication of clinical research and a completed CONSORT checklist must be included with all submissions.

Clinical trial registration | *Provide the trial registration number from ClinicalTrials.gov or an equivalent agency.*

Study protocol | *Note where the full trial protocol can be accessed OR if not available, explain why.*

Data collection | *Describe the settings and locales of data collection, noting the time periods of recruitment and data collection.*

Outcomes | *Describe how you pre-defined primary and secondary outcome measures and how you assessed these measures.*

Dual use research of concern

Policy information about dual use research of concern

Hazards

Could the accidental, deliberate or reckless misuse of agents or technologies generated in the work, or the application of information presented in the manuscript, pose a threat to:

No	Yes	
☐	☐	Public health
☐	☐	National security
☐	☐	Crops and/or livestock
☐	☐	Ecosystems
☐	☐	Any other significant area

Experiments of concern

Does the work involve any of these experiments of concern:

No	Yes	
☐	☐	Demonstrate how to render a vaccine ineffective
☐	☐	Confer resistance to therapeutically useful antibiotics or antiviral agents
☐	☐	Enhance the virulence of a pathogen or render a nonpathogen virulent
☐	☐	Increase transmissibility of a pathogen
☐	☐	Alter the host range of a pathogen
☐	☐	Enable evasion of diagnostic/detection modalities
☐	☐	Enable the weaponization of a biological agent or toxin
☐	☐	Any other potentially harmful combination of experiments and agents

ChIP-seq

Data deposition

☐ Confirm that both raw and final processed data have been deposited in a public database such as GEO.

☐ Confirm that you have deposited or provided access to graph files (e.g. BED files) for the called peaks.

Data access links
May remain private before publication. | *For "Initial submission" or "Revised version" documents, provide reviewer access links. For your "Final submission" document, provide a link to the deposited data.*

Files in database submission | *Provide a list of all files available in the database submission.*

Genome browser session
(e.g. UCSC)

Provide a link to an anonymized genome browser session for "Initial submission" and "Revised version" documents only, to enable peer review. Write "no longer applicable" for "Final submission" documents.

Methodology

Replicates	*Describe the experimental replicates, specifying number, type and replicate agreement.*
Sequencing depth	*Describe the sequencing depth for each experiment, providing the total number of reads, uniquely mapped reads, length of reads and whether they were paired- or single-end.*
Antibodies	*Describe the antibodies used for the ChIP-seq experiments; as applicable, provide supplier name, catalog number, clone name, and lot number.*
Peak calling parameters	*Specify the command line program and parameters used for read mapping and peak calling, including the ChIP, control and index files used.*
Data quality	*Describe the methods used to ensure data quality in full detail, including how many peaks are at FDR 5% and above 5-fold enrichment.*
Software	*Describe the software used to collect and analyze the ChIP-seq data. For custom code that has been deposited into a community repository, provide accession details.*

Flow Cytometry

Plots

Confirm that:

- [ ] The axis labels state the marker and fluorochrome used (e.g. CD4-FITC).
- [ ] The axis scales are clearly visible. Include numbers along axes only for bottom left plot of group (a 'group' is an analysis of identical markers).
- [ ] All plots are contour plots with outliers or pseudocolor plots.
- [ ] A numerical value for number of cells or percentage (with statistics) is provided.

Methodology

Sample preparation	*Describe the sample preparation, detailing the biological source of the cells and any tissue processing steps used.*
Instrument	*Identify the instrument used for data collection, specifying make and model number.*
Software	*Describe the software used to collect and analyze the flow cytometry data. For custom code that has been deposited into a community repository, provide accession details.*
Cell population abundance	*Describe the abundance of the relevant cell populations within post-sort fractions, providing details on the purity of the samples and how it was determined.*
Gating strategy	*Describe the gating strategy used for all relevant experiments, specifying the preliminary FSC/SSC gates of the starting cell population, indicating where boundaries between "positive" and "negative" staining cell populations are defined.*

- [ ] Tick this box to confirm that a figure exemplifying the gating strategy is provided in the Supplementary Information.

Magnetic resonance imaging

Experimental design

Design type	*Indicate task or resting state; event-related or block design.*
Design specifications	*Specify the number of blocks, trials or experimental units per session and/or subject, and specify the length of each trial or block (if trials are blocked) and interval between trials.*
Behavioral performance measures	*State number and/or type of variables recorded (e.g. correct button press, response time) and what statistics were used to establish that the subjects were performing the task as expected (e.g. mean, range, and/or standard deviation across subjects).*

Acquisition

Imaging type(s) | *Specify: functional, structural, diffusion, perfusion.*

Field strength | *Specify in Tesla*

Sequence & imaging parameters | *Specify the pulse sequence type (gradient echo, spin echo, etc.), imaging type (EPI, spiral, etc.), field of view, matrix size, slice thickness, orientation and TE/TR/flip angle.*

Area of acquisition | *State whether a whole brain scan was used OR define the area of acquisition, describing how the region was determined.*

Diffusion MRI ☐ Used ☐ Not used

Preprocessing

Preprocessing software | *Provide detail on software version and revision number and on specific parameters (model/functions, brain extraction, segmentation, smoothing kernel size, etc.).*

Normalization | *If data were normalized/standardized, describe the approach(es): specify linear or non-linear and define image types used for transformation OR indicate that data were not normalized and explain rationale for lack of normalization.*

Normalization template | *Describe the template used for normalization/transformation, specifying subject space or group standardized space (e.g. original Talairach, MNI305, ICBM152) OR indicate that the data were not normalized.*

Noise and artifact removal | *Describe your procedure(s) for artifact and structured noise removal, specifying motion parameters, tissue signals and physiological signals (heart rate, respiration).*

Volume censoring | *Define your software and/or method and criteria for volume censoring, and state the extent of such censoring.*

Statistical modeling & inference

Model type and settings | *Specify type (mass univariate, multivariate, RSA, predictive, etc.) and describe essential details of the model at the first and second levels (e.g. fixed, random or mixed effects; drift or auto-correlation).*

Effect(s) tested | *Define precise effect in terms of the task or stimulus conditions instead of psychological concepts and indicate whether ANOVA or factorial designs were used.*

Specify type of analysis: ☐ Whole brain ☐ ROI-based ☐ Both

Statistic type for inference (See Eklund et al. 2016) | *Specify voxel-wise or cluster-wise and report all relevant parameters for cluster-wise methods.*

Correction | *Describe the type of correction and how it is obtained for multiple comparisons (e.g. FWE, FDR, permutation or Monte Carlo).*

Models & analysis

n/a	Involved in the study
☐	☐ Functional and/or effective connectivity
☐	☐ Graph analysis
☐	☐ Multivariate modeling or predictive analysis

Functional and/or effective connectivity | *Report the measures of dependence used and the model details (e.g. Pearson correlation, partial correlation, mutual information).*

Graph analysis | *Report the dependent variable and connectivity measure, specifying weighted graph or binarized graph, subject- or group-level, and the global and/or node summaries used (e.g. clustering coefficient, efficiency, etc.).*

Multivariate modeling and predictive analysis | *Specify independent variables, features extraction and dimension reduction, model, training and evaluation metrics.*
